# Gold‐Catalyzed Atroposelective Synthesis of 1,1′‐Binaphthalene‐2,3′‐diols

**DOI:** 10.1002/anie.201915456

**Published:** 2020-01-23

**Authors:** Jianwei Zhang, Martin Simon, Christopher Golz, Manuel Alcarazo

**Affiliations:** ^1^ Institut für Organische und Biomolekulare Chemie Georg August Universität Göttingen Tammannstr 2 37077 Göttingen Germany

**Keywords:** gold catalysis, biaryls, cationic ligands, chiral phosphines, enantioselectivity

## Abstract

A highly atroposelective (up to 97 % *ee*) Au‐catalyzed synthesis of 1,1′‐binaphthalene‐2,3′‐diols is reported starting from a range of substituted benzyl alkynones. Essential for the achievement of high enantioselectivity during the key assembly of the naphto‐3‐ol unit is the use of TADDOL‐derived α‐cationic phosphonites as ancillary ligands. Preliminary results demonstrate that the transformation of the obtained binaphthyls into axially chiral monodentate phosphines is possible without degradation of enantiopurity.

## Graphical abstract

**Remote control**: Two methyl groups strategically located at the backbone of the imidazolium component of a gold‐based catalyst are essential to deliver the desired binaphthol compounds in up to 97 % *ee*.

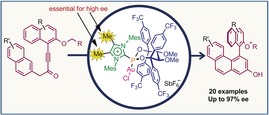

The presence of substituents at the *ortho* positions flanking the aryl–aryl bond in biaryl compounds restricts and may even cancel the rotation around that bond, rendering molecules that contain such architectures axially chiral.^1^ Far from being just a curiosity, this type of isomerism, specifically called atropoisomerism, is ubiquitous in natural products,^2^ pharmaceuticals,^3^ organocatalysts,^4^ and ligands for transition metals^5^ (Figure 1 a). This feature has stimulated the development of a number of atroposelective routes targeting the preparation of such motives in enantiopure form.^6^ Arguably, one of the most commonly found axially chiral architectures is the one derived from 1,1′‐naphthyl‐2,2′‐diol (BINOL), which has been recognized as a “privileged structure” in the area of enantioselective catalysis because of its ability to promote high enantioinduction in mechanistically non‐related asymmetric processes.^7^ BINOL itself, and related *C_2_*‐symmetrical 1,1′‐biaryl‐2,2′‐diols, are often prepared by oxidative coupling of the corresponding phenols; a process that takes place with very high regioselectivity and can be carried out on a large scale.^8^ However, the asymmetric synthesis of other isomers of BINOL, in particular those that are non‐*C_2_*‐symmetric, is more challenging because in addition to enantiodiscrimination, regio‐, and/or diasteroselection need to be simultaneously controlled and optimized.^9^ For example, the formal displacement of one of the −OH units in the original BINOL scaffold to the adjacent C‐position renders 1,1′‐naphthyl‐2,3′‐diol, which is the basic structural motive of denthyrsinone. Only one enantioselective synthesis, recently reported by Tanaka and co‐workers, is available for this scaffold.^10^ These authors were able to construct the 3‐naphthol unit through a Au‐catalyzed atroposelective hydroarylation of an alkynone moiety strategically located in position 1‐ of the 2‐naphthol fragment (Figure 1 b). The transformation proceeds with good to excellent yields, but only moderate enantioselectivities (up to 67 % *ee*) were obtained despite the exhaustive inspection of a complete series of non‐structurally related chiral diphosphine ligands.

**Figure 1:**
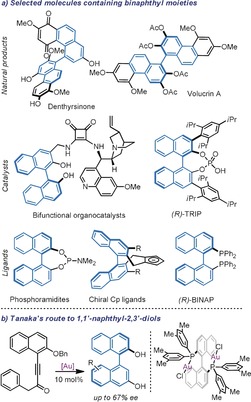
a) Axially chiral natural products, catalysts, and ligands. b) Available route for the preparation of 1,1′‐naphthyl‐2,3′‐diols.

Very recently, we reported the synthesis of TADDOL‐derived α‐cationic phosphonites and their application as ancillary ligands in the Au‐catalyzed hydroarylation of dyines into [6]helicenes.^11^, ^12^ In these ligands the chiral information is provided by an easily available and well precedented TADDOL‐derived moiety, whose modular synthesis allows an easy modification of their structures upon demand. Moreover, the positively charged heterocyclic substituent directly attached to the phosphorus center provides enhanced Lewis acidity at the metal center if compared with traditional catalysts. This feature results in higher catalytic activity and the possibility to work at lower temperatures.^13^ Given these precedents, we hypothesized that even if the use of α‐cationic ligands for the induction of atroposelectivity has not been studied, these ligands might also be beneficial for the enantioselective preparation of 1,1′‐naphthyl‐2,3′‐diols. Our advances in this direction are reported herein.

We initially studied the performance of catalysts **5 a**–**c** employing Tanaka's model substrates **1 a**. From this set of experiments, 4‐(trifluoromethyl)phenyl groups on the TADDOL moiety were identified to be essential to promote enantiomeric excess (Table 1, Entries 1–3); hence, that substituent was fixed and a subsequent optimization focused on the evaluation of the impact of the outer TADDOL backbone. Interestingly, we found that flexible dimethyl ether groups were superior to a cyclic acetonide to achieve enantiodiscrimination (Table 1, Entry 5).^14^

**Table 1:** Ligand and substrate optimization. 
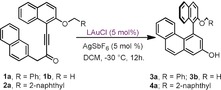

Entry	Substrate	Catalyst	Yield [%]^[a]^	*ee* [%]^[b]^
1	**1 a**	**5 a**	90	(+) 2
2	**1 a**	**5 b**	93	0
3	**1 a**	**5 c**	91	(+) 30
4	**1 a**	**6 a**	85	(−) 38
5	**1 a**	**6 b**	83	(−) 50
6	**1 a**	**6 c**	90	(−) 95
7	**1 a**	**6 d**	86	(−) 88
8	**2 a**	**6 c**	90	(−) 97
9	**2 a**	**6 d**	87	(−) 95
10	**1 b**	**6 c**	81	(−) 64

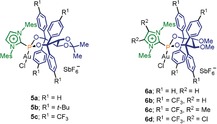

All reactions were conducted on a 0.25 mmol scale in DCM (0.05 m). [a] Yields are that of isolated product; [b] *ee* values were determined by chiral HPLC.

Next, the influence of substitution at the backbone of the imidazolium ring was examined. To our surprise, we found that the mere presence of substituents in that region translates into higher levels of enantioinduction, and is independent of the electronic nature of the group incorporated (Me− or Cl−) as can be seen in Table 1, Entries 6 and 7. Careful scrutiny of the X‐ray structures of **5 c** and **6 c**, an overlay is shown in Figure 2, showed that the geometries adopted by both frameworks are very similar. However, it worth mentioning that the methyl or chloride groups at the imidazolium unit slightly push the vicinal mesityl components towards the inner cavity of the ligand, narrowing the chiral pocket around the Au atom. One *p*‐(CF_3_)Ph moiety suffers an identical effect on introduction of the methyl groups.^15^ The increment of steric pressure is confirmed by comparison of the percent buried volumes (%*V*_Bur_; **5 c**, 46.1 % and **6 c**, 47.8 %), and the topographic steric maps for both ligands, depicted in Figure 2.^16^ Hence, the ultimate reason for such a beneficial effect still remains obscure, but we believe that it is the tighter puckering of the ligand around the metal atom that causes the improved enantioinduction.

**Figure 2:**
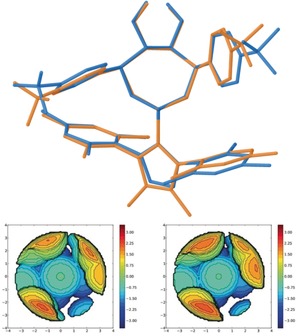
Overlay of the X‐ray structures of **5 c** (blue) and **6 c** (orange) seen from the Cl‐Au‐P axes; only the inward orientated *p*‐(CF_3_)Ph groups are shown for clarity. Steric maps for **5 c** (left) and **6 c** (right); %*V*_Bur_; **5 c**, 46.1 % and **6 c**, 47.8 %.

Finally, given the highly aromatic nature of both the substrate and the catalyst, and the complementary electronic properties of their substituents, it was rationalized that attractive π‐stacking interactions surely play a fundamental role in achieving enantiodiscrimination in this process.^17^ With that in mind, we exchanged the benzyl group in **1 a** with a more π‐extended 2‐naphthalenemethyl component (**2 a**). Our intention was to reinforce the attractive interaction between the ligand and substrate substituents and thus, reduce the conformational degrees of freedom in the selectivity determining the transition state of the catalytic cycle. We were glad to see that this assumption seems to be correct. In fact, both precatalysts **6 c** and **6 d**, were able to achieve higher enantiomeric excess values after this subtle change (Table 1, Entries 8 and 9). In contrast, the substitution of the benzyl group in **1 a** by a methyl group in **1 b** leads to a severe drop in the enantioselectivity (Table 1, Entry 10). These results, therefore, identified **6 c** as the optimal catalyst, and the 2‐naphthalenemethyl unit as the preferred protecting group for the naphthol moiety.

With the optimal conditions in hand, the scope of the cyclization was evaluated by using alkynones **2 a**–**u** (Figure 3), which contain diverse substitution patterns, including additional halogen, arene, and alkyl substituents at diverse positions of the original substrate (see the Supporting Information for the preparation of ynones **2 a**–**u**). Gratifyingly, high chemical yields were obtained for the desired hydroarylation process, and the high enantioinduction imparted by **6 c** was consistently maintained throughout the series. It is also worth noting that, a priori, two positions (1‐ and 3‐) of the naphthalene substituent in **2 a**–**t** might attack the activated alkyne, affording two regioisomeric binaphthols; however, only the shown binaphthols **4 a**–**t** were observed, despite being the more sterically demanding regioisomers. Low *ee* values were observed for substrate **2 u**, containing a benzyl alkynone instead of 2‐(methyl)naphthyl alkynone substituent; this result again corroborates the positive influence of expanded π‐systems in the enantioselectivity of the cyclization when using our catalytic system.

**Figure 3:**
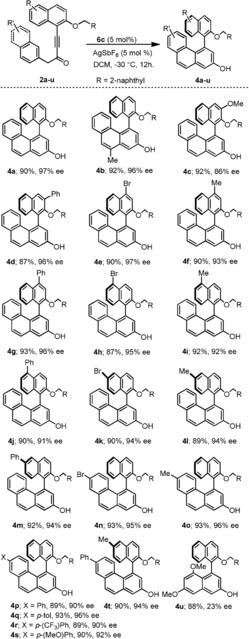
Substrate scope. All reactions were conducted on a 0.25 mmol scale in DCM (0.05 m). Yields are that of isolated product and *ee* values were determined by chiral HPLC.

To demonstrate the utility of the products obtained, we decided to further elaborate **4 a** into an axially chiral monodentate phosphine. Thus, methylation of the free alcohol followed by hydrogenolysis of the 2‐naphthyl group afforded, in 82 % yield (two steps), alcohol **8**, which was subsequently transformed into the corresponding triflate **9** by treatment with trifluoromethanesulfonic anhydride. This compound was then submitted to palladium‐catalyzed phosphinylation under already reported conditions to render the expected phosphine oxide **10** in 78 % yield.^18^ Reduction of **10** to the desired phosphine **11** was achieved by employing a HSiCl_3_/Et_3_N mixture in boiling toluene. Importantly, analysis by chiral HPLC of compounds **7**–**11** revealed no erosion of the enantiomeric purity along the complete sequence. Finally, reaction of **11** with (Me_2_S)AuCl afforded Au complex **12** (Scheme 1).

**Scheme 1:**
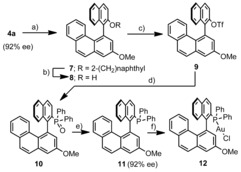
Transformation of **4 a** into a new axially chiral phosphine. Reagents and conditions: a) MeI, K_2_CO_3_, CH_3_CN, 93 %; b) Pd/C, H_2_, EtOH, reflux, 88 %; c) Tf_2_O, Et_3_N, 60 %; d) Ph_2_P(O)H, Pd(OAc)_2_ (10 mol %), dppb (12 mol %), DMSO, 110 °C, 78 %; e) HSiCl_3_/NEt_3_ (5:7 equiv), 100 °C, 18 h, 87 %; f) (Me_2_S)AuCl, DCM, 0 °C→r.t. (90 %).

Monocrystals of **12** were obtained by slow diffusion of diethylether into a saturated dichloromethane solution of the title compound, and its structure was determined by X‐ray diffraction. This analysis allowed the assignment of the absolute configuration of **12**, and by extension that of **4 a**–**t**, to be *M* (Figure 4).

**Figure 4:**
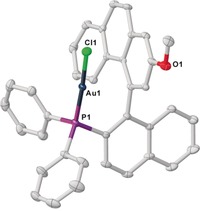
Molecular structure of compound **12** in the solid state. Anisotropic displacement shown at 50 % probability level and hydrogen atoms omitted for clarity.^19^

In conclusion, a highly atroposelective synthesis of 1,1′‐binaphthalene‐2,3′‐diols is reported via Au‐catalyzed intramolecular hydroarylation of appropriately designed alkynones. The use of 3,4‐disubstituted imidazolium units directly attached to the phosphorus atom of the ancillary ligand, and 2‐naphthalenemethyl moieties as protecting groups at the naphthol substrates **2 a**–**t** were found to be essential to achieve high enantioselectivities. The possibility to transform the 1′‐binaphthalene‐2,3′‐diols described into new chiral phosphines might inspire the development of new asymmetric Au‐catalyzed transformations. Explorative studies in this direction are currently in progress in our laboratory.

## Conflict of interest

The authors declare no conflict of interest.

## Supporting information

As a service to our authors and readers, this journal provides supporting information supplied by the authors. Such materials are peer reviewed and may be re‐organized for online delivery, but are not copy‐edited or typeset. Technical support issues arising from supporting information (other than missing files) should be addressed to the authors.

Supplementary
