# Supplementary material for: Gold‐Catalyzed Atroposelective Synthesis of 1,1′‐Binaphthalene‐2,3′‐diols
Source: Angew Chem Int Ed Engl. 2020 Jan 23;59(14):5647–50. doi: 10.1002/anie.201915456 (PMC7155015; doi:10.1002/anie.201915456)
Supplement: Supplementary file 1 — Supplementary [file ANIE-59-5647-s001.pdf]

Angewandte Chemie
Eine Zeitschrift der Gesellschaft Deutscher Chemiker

Supporting Information

Gold-Catalyzed Atroposelective Synthesis of 1,1′-Binaphthalene-2,3′-diols

*Jianwei Zhang, Martin Simon, Christopher Golz, and Manuel Alcarazo**

anie_201915456_sm_miscellaneous_information.pdf

Contents

1. General information 2
2. Optimization of reaction conditions 3
3. General procedure for the synthesis of alkynones 6
4. General procedure for the Au-catalyzed hydroarylation of alkynones 21
5. Transformation of **4**a into **12** 39
6. Crystal structure of **12** 43
7. References 44
8. NMR spectra 45
9. HPLC spectra 99

1. General information

Unless otherwise noted, all experiments were carried out under an atmosphere of nitrogen using standard Schlenk techniques or in a nitrogen-filled glovebox. All NMR spectra were recorded on Bruker AV600, and AV400; ^{1}H and ^{13}C chemical shifts (δ) are given in ppm relative to TMS, using the solvent signals as references and converting the chemical shifts to the TMS scale. ^{31}P and ^{19}F chemical shifts (δ) are given in ppm relative to H_3PO_4 and $CFCl_3$ respectively (external standard). Coupling constants (J) are given in Hz. All high resolution mass spectra were obtained on Finnigan MAT 95 (70 eV, EI), Finnigan LCQ (ESI) and APEX IV 7T FTICR, Bruker Daltonic (HRMS). For thin layer chromatography (TLC), Merck silica gel 60 F254 TLC plates were used, and compounds were visualized with a UV light at 254 nm. Chiral HPLC: the enantiomeric excesses of the products in catalysis reactions was determined using either a Shimadzu Nexera-*i* LC 2040 3D with integrated downstream UV/Vis PDA detector. Optical rotations were measured using Perkin Elmer 343 or Jasco P-2000 polarimeters at the stated temperature under a Na/Hg lamp, λ = 589 nm (c in g/100 ml). Dry and degassed solvents were obtained by distillation over the appropriate drying agents and stored under nitrogen. Alternatively, dry solvents were obtained using an MBraun MB-SPS-800 solvent purification system (tetrahydrofuran, diethyl ether, toluene, pentane, dichloromethane, acetonitrile). All other chemicals were used as received from Aldrich or Acros without further purification. The catalysts were prepared according to the published method.[1] All substrates of alkynones were synthesized according to modified literature method.[2,3]

2. Optimization of reaction conditions

Table S1: Preliminary Experiments[a]

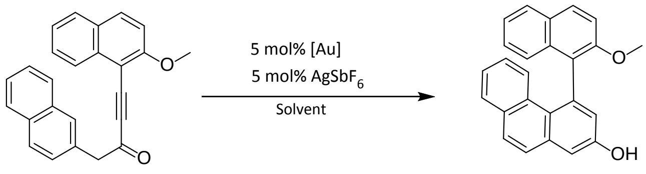

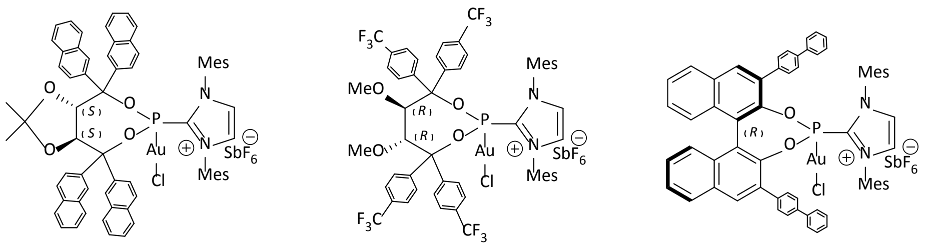

5d 6b 6e

Entry	Solvent	[Au]	T (°C)	yield (%)[b]	*ee* (%)[c]
1	DCE	**5d**	40	90	-11
2	DCE	**6b**	40	87	19
3	DCE	**6e**	40	75	11
4[d]	DCM	**5d**	-20	85	-20
5[d]	DCM	**6b**	-20	80	41
6[d]	DCM	**6e**	-20	67	24

[a] Reaction conditions: substrate (0.025 mmol) in solvent (0.5 mL), [Au]-catalyst (5 mol%), $AgSbF_6$ (5 mol%), stirred for 6 h. [b] Yield of isolated product. [c] The enantiomeric excesses were determined by chiral HPLC. [d] stirred for 12 h.

***Table S2*: Evaluation of Different Catalysts[a]**

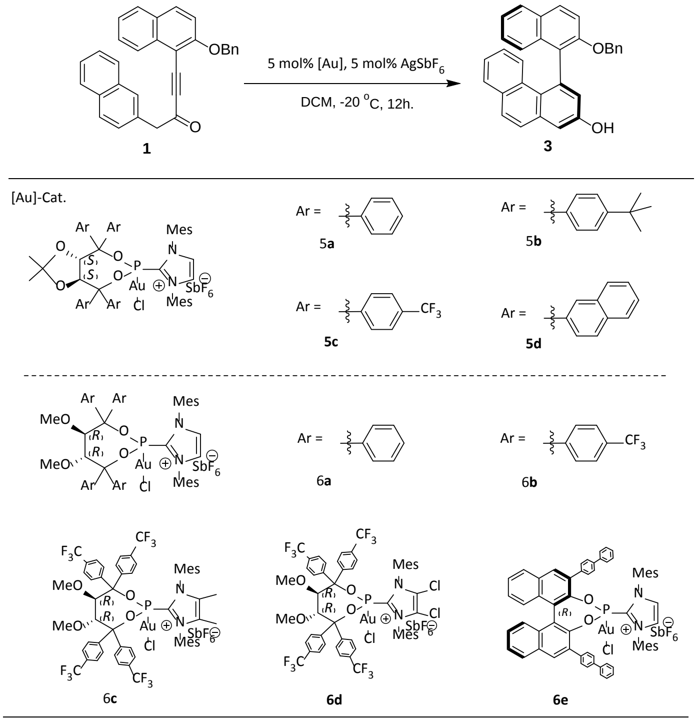

Entry	[Au]-Cat.	Solvent	Conv.[%] [b]	Yield [%][c]	*ee* [%][c]
1	none	DCM	0	0	-
2	**5a**	DCM	> 95	90	-2
3	**5b**	DCM	> 95	93	0
4	**5c**	DCM	> 95	91	-30
5	**5d**	DCM	> 95	87	-3
6	**6a**	DCM	> 95	85	38
7	**6b**	DCM	> 95	83	50
**8**	**6c**	**DCM**	**> 95**	**90**	**95**
9	**6d**	DCM	> 95	86	88
10	**6e**	DCM	> 95	90	0

[a] Reaction conditions: substrate (0.025 mmol) in DCM (0.5 mL), [Au]-catalyst (5.0 mol%), $AgSbF_6$ (5.0 mol%), stirred at -20 °C for 12 h. [b] The conversions were determined by ^{1}H-NMR spectroscopy of the crude reaction mixture. [c] Yield of isolated product. [d] The enantiomeric excesses were determined by HPLC with a chiral column.

***Table S3*: Evaluation of Different Protecting Groups**[a]

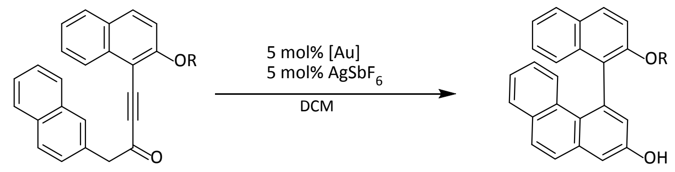

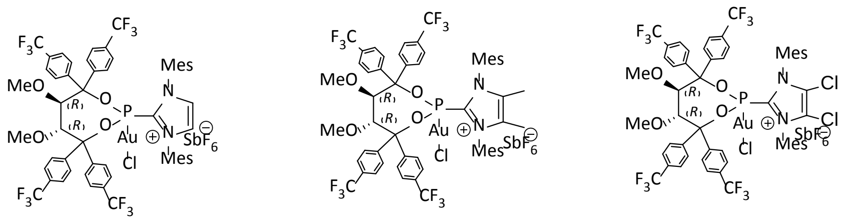

Entry	R	[Au]	T (°C)	Conv. (%)[b]	Yield (%)[c]	*ee* (%)[d]
1	CH_3	**6b**	-20	>95	90	41
2	benzyl	**6b**	-20	>95	83	50
3	2-naphthylmethyl	**6b**	-20	>95	92	57
4	benzyl	**6c**	-20	>95	90	95
**5**[e]	**2-naphthylmethyl**	**6c**	**-30**	**>95**	**90**	**97**
6[e]	2-naphthylmethyl	**6d**	-30	>95	87	95

[a] Reaction conditions: substrate (0.025 mmol) in DCM (0.5 mL), [Au]-catalyst (5.0 mol%), $AgSbF_6$ (5.0 mol%), stirred for 12 h. [b] The conversions were determined by ^{1}H-NMR spectroscopy of the crude reaction mixture. [c] Yield of isolated product. [d] The enantiomeric excesses were determined by HPLC with a chiral column. [e] stirred for 18 h.

3. General procedure for the synthesis of alkynones

General procedure for the synthesis of **2a-b** and **2e-t** [2]

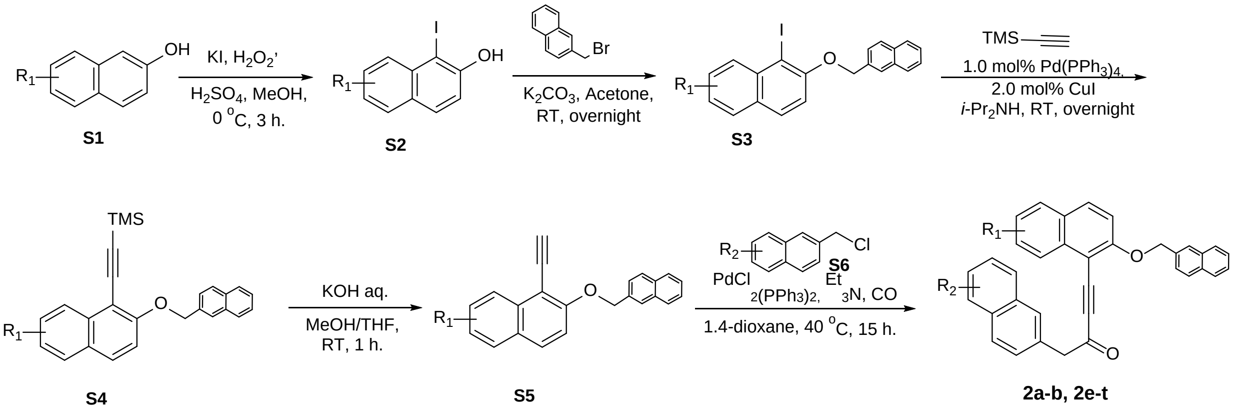

2-naphthol **S1** (20.6 mmol, 1.00 eq.) and KI (20.6 mmol, 1.00 eq.) were added to a solution of conc. H_2SO_4 (1.60 mL, 30.9 mmol, 1.50 eq.) in MeOH (100 mL) at 0 °C. After this, 35% aq. H_2O_2 solution (41.2 mmol, 2.00 eq.) was added and the mixture stirred for 80 min. After this, the reaction mixture was poured into CH_2Cl_2 (100 mL) and subsequently washed with 0.1 M $NaHSO_3$ solution (100 mL) and H_2O (100 mL). The organic phase was dried over $MgSO_4$ and the solvent evaporated. Finally, the crude product was submitted to flash column chromatography (SiO2, hexanes/EtOAc 50:1) to yield the pure products **S2**.

K_2CO_3 (3.11 g, 22.5 mmol, 1.50 eq.) was added to a solution of 1-iodonaphthalenes **S2** (15 mmol, 1.00 eq.) and 2-naphtylmethyl bromide (22.5 mmol, 1.50 eq.) in acetone (100 mL). The resulting mixture was stirred at room temperature for 13 h. After filtration, the solvents were concentrated and the residue was purified by a silica gel column chromatography (hexane/acetone = 50:1, v/v) to give products **S3**.

$Pd(PPh_3)_4$ (0.13 mmol, 0.01 eq.) was added to a solution of **S3** (13.3 mmol, 1.00 eq.) and trimethylsilyl acetylene (19.9 mmol, 1.50 eq.) in *i*-Pr_2NH (80 mL). The mixture thus obtained

was stirred for 5 min, and finally CuI (0.26 mmol, 0.02 eq.) was added. The resulting mixture was stirred at room temperature for 15 h and subsequently concentrated to afford the corresponding crude **S4**, which was used for the next step without further purification.

A solution of KOH (ca. 0.1 g) in distilled water (5 mL) was added to a solution of crude **S4** in MeOH (60 mL) and THF (15 mL). The mixture obtained was stirred at RT for 30 min. and subsequently diluted with water (30 mL) and extracted with ether. After separation of the phases, the organic one was washed with brine, dried over Na_2SO_4, and concentrated. The residue obtained was purified by silica gel column chromatography (hexane:acetone = 30:1, v/v) to afford the desired 1-ethynyl-2-(naphthalen-2-ylmethoxy)naphthalene **S5** as a yellow solid.

Finally, $PdCl_2(PPh_3)_2$ (0.13 mmol, 0.05 eq.), the desired 2-(chloromethyl)naphthalene **S6** (2.6 mmol, 1.00 eq.), **S5** (2.9 mmol, 1.10 eq.) and Et_3N (3.9 mmol, 1.50 eq.), were suspended in 1,4-dioxane (10 mL) under a N_2 atmosphere. The flask was purged with CO (1 atm) three times, and heated to 40 °C for 15 h. After cooling the reaction mixture to room temperature, the remaining CO was vented, the reaction was quenched with water and finally extracted with CH_2Cl_2. The organic layer was washed with brine, dried over Na_2SO_4, and concentrated in vacuo affording a residue that was purified by silica gel column chromatography (CH_2Cl_2/MeOH = 150:1, v/v) to give the alkynone products **2a-b** and **2e-t**.

Characterization data of **2a-b** and **2e-t**.

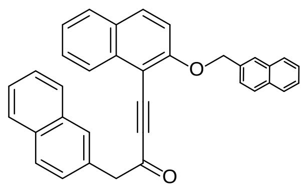

2a

Yellow solid; **^{1}H NMR** (400 MHz, $CDCl_3$): δ (ppm) 7.96 (s, 1H), 7.90 – 7.85 (m, 2H), 7.85 – 7.74 (m, 7H), 7.69 (d, *J* = 8.1 Hz, 1H), 7.66 – 7.61 (m, 1H), 7.55 (dd, *J* = 8.5, 1.7 Hz, 1H), 7.50 – 7.45 (m, 5H), 7.33 – 7.27 (m, 1H), 7.22 (d, *J* = 9.2 Hz, 1H), 7.11 – 7.07 (m, 1H), 5.44 (s, 2H), 4.16 (s, 2H); **^{13}C NMR** (101 MHz, $CDCl_3$): δ (ppm) 185.4, 161.2, 134.9, 134.0, 133.8, 133.5, 133.4, 133.2, 132.8, 131.4, 129.1, 128.6, 128.6, 128.5, 128.3, 128.2, 128.2, 128.1, 127.9, 127.9, 126.5, 126.3, 126.3, 126.0, 126.0, 125.0, 124.9, 124.7, 114.2, 103.9, 98.0, 89.1, 71.4, 52.5. **IR** (neat) 439, 475, 549, 667, 736, 751, 761, 773, 812, 824, 856, 949, 1019, 1044, 1137, 1149, 1227, 1277, 1650, 2177 cm^{-1}. **HRMS-ESI** exact mass calcd. for $C_{35}H_{24}O_2Na$ ($[M+Na]^+$) requires m/z 499.1669, found m/z 499.1656.

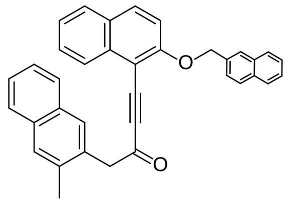

2b

Yellow solid; **^{1}H NMR** (400 MHz, $CDCl_3$): δ (ppm) 7.99 (s, 1H), 7.96 – 7.90 (m, 1H), 7.90 – 7.83 (m, 3H), 7.80 (dd, *J* = 8.7, 3.0 Hz, 3H), 7.74 – 7.62 (m, 2H), 7.60 – 7.36 (m, 6H), 7.32 – 7.26 (m, 1H), 7.21 (d, *J* = 9.1 Hz, 1H), 6.91 (dd, *J* = 8.5, 6.9 Hz, 1H), 5.42 (s, 2H), 4.20 (s, 2H), 2.54 (s, 3H); **^{13}C NMR** (101 MHz, $CDCl_3$): δ (ppm) 185.7, 161.3, 135.7, 134.9, 134.0, 133.5, 133.5, 133.4, 133.2, 132.5, 131.7, 130.2, 128.7, 128.6, 128.4, 128.2, 128.2, 128.2, 127.8, 127.6, 127.2, 126.5, 126.2, 126.1, 125.9, 125.5, 124.9, 124.8, 124.6, 114.1, 103.9, 97.9, 88.8, 71.3, 50.7, 20.5. **IR** (neat) 421, 439, 449, 560, 570, 675, 761, 775, 805, 888, 1048, 1077, 1122, 1148, 1217, 1229, 1276, 1349, 1375, 1508, 1642, 1739, 2175 cm^{-1}. **HRMS-ESI** exact mass calcd. for $C_{36}H_{26}O_2Na$ ($[M+Na]^+$) requires m/z 513.1825, found

m/z 513.1817.

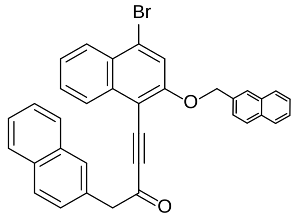

2e

Yellow solid; **^{1}H NMR** (400 MHz, $CDCl_3$): δ (ppm) 8.08 (d, *J* = 8.5 Hz, 1H), 7.97 (s, 1H), 7.91 – 7.76 (m, 7H), 7.61 – 7.59 (m, 2H), 7.55 (dd, *J* = 8.5, 1.7 Hz, 1H), 7.51 – 7.43 (m, 5H), 7.40 – 7.36 (m, 1H), 7.09 – 7.05 (m, 1H), 5.41 (s, 2H), 4.13 (s, 2H); **^{13}C NMR** (101 MHz, $CDCl_3$): δ (ppm) 185.3, 160.2, 135.2, 133.8, 133.5, 133.4, 133.3, 132.8, 131.2, 129.1, 129.0, 128.7, 128.7, 128.5, 128.3, 128.0, 127.9, 127.9, 127.6, 127.5, 126.6, 126.4, 126.4, 126.2, 126.1, 125.4, 124.7, 118.7, 104.2, 98.5, 88.2, 71.7, 52.5. **IR** (neat) 413, 445, 617, 679, 776, 801, 812, 822, 834, 902, 1048, 1089, 1227, 1257, 1274, 1325, 1497, 1646 cm^{-1}. **HRMS-ESI** exact mass calcd. for $C_{35}H_{23}BrO_2Na$ ($[M+Na]^+$) requires m/z 577.0774, found m/z 577.0760.

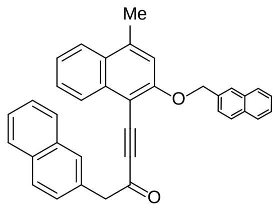

2f

White solid; **^{1}H NMR** (400 MHz, $CDCl_3$): δ (ppm) 7.98 (s, 1H), 7.93 – 7.70 (m, 8H), 7.68 (d, *J* = 8.3 Hz, 1H), 7.57 (dd, *J* = 8.5, 1.7 Hz, 1H), 7.50 – 7.44 (m, 5H), 7.50 – 7.44 (m, 5H), 7.35 – 7.31 (m, 1H), 7.11 – 7.07 (m, 2H), 5.43 (s, 2H), 4.15 (s, 2H), 2.64 (s, 3H); **^{13}C NMR** (101 MHz, $CDCl_3$): δ (ppm) 185.3, 161.0, 141.5, 135.0, 134.2, 133.8, 133.5, 133.2, 132.8, 131.5, 129.0, 128.6, 128.6, 128.2, 128.1, 128.0, 127.9, 127.9, 126.5, 126.3, 126.2, 126.0, 126.0, 125.6, 124.8, 124.5, 115.3, 102.1, 98.1, 89.9, 52.5, 20.5. **IR** (neat) 418, 619, 681, 812, 836, 860, 901, 1049, 1095, 1130, 1214, 1234, 1264, 1275, 1340, 1353, 1508, 1641, 2164 cm^{-1}. **HRMS-ESI** exact mass calcd. for $C_{36}H_{26}O_2Na$ ($[M+Na]^+$) requires m/z 513.1825, found m/z 513.1815.

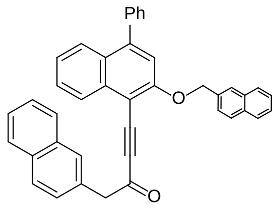

2g

Yellow solid; **^{1}H NMR** (400 MHz, $CDCl_3$): δ (ppm) 8.04 (s, 1H), 7.95 – 7.93 (m, 1H), 7.91 – 7.81 (m, 6H), 7.79 – 7.72 (m, 2H), 7.61 (dd, *J* = 8.5, 1.8 Hz, 1H), 7.55 – 7.48 (m, 8H), 7.41 (dt, *J* = 7.1, 2.0 Hz, 2H), 7.30 – 7.26 (m, 1H), 7.15 – 7.11 (m, 1H), 5.48 (s, 2H), 4.21 (s, 2H); **^{13}C NMR** (101 MHz, $CDCl_3$): δ (ppm) 185.3, 160.5, 146.1, 139.7, 135.4, 133.9, 133.8, 133.5, 133.2, 132.8, 131.4, 129.8, 129.0, 128.6, 128.6, 128.2, 128.1, 128.1, 127.9, 127.9, 127.9, 127.1, 126.7, 126.5, 126.3, 126.3, 126.2, 126.0, 125.4, 124.9, 124.9, 115.3, 103.4, 98.4, 89.3, 71.4, 52.5. **IR** (neat) 420, 437, 451, 617, 639, 689, 858, 1026, 1046, 1089, 1229, 1153, 1215, 1342, 1359, 1560, 1575, 1650, 2172 cm^{-1}. **HRMS-ESI** exact mass calcd. for $C_{41}H_{28}O_2Na$ ($[M+Na]^+$) requires m/z 575.1982, found m/z 575.1966.

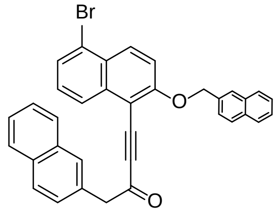

2h

Yellow solid; **^{1}H NMR** (400 MHz, $CDCl_3$): δ (ppm) 8.21 (d, *J* = 9.4 Hz, 1H), 7.95 (d, *J* = 1.6 Hz, 1H), 7.91 – 7.72 (m, 7H), 7.58 – 7.42 (m, 8H), 7.29 (d, *J* = 9.4 Hz, 1H), 6.81 (dd, *J* = 8.5, 7.4 Hz, 1H), 5.44 (s, 2H), 4.15 (s, 2H); **^{13}C NMR** (101 MHz, $CDCl_3$): δ (ppm) 185.3, 161.5, 136.2, 133.8, 133.7, 133.4, 133.3, 132.8, 132.6, 131.3, 129.1, 128.9, 128.7, 128.7, 128.5, 128.2, 128.0, 127.9, 127.9, 127.0, 126.6, 126.4, 126.4, 126.1, 126.1, 124.9, 124.6, 123.2, 115.1, 104.3, 98.1, 88.3, 71.3, 52.5. **IR** (neat) 420, 572, 668, 688, 707, 759, 776, 794, 814, 824, 857, 1052, 1086, 1124, 1144, 1233, 1259, 1271, 1650, 2176 cm^{-1}. **HRMS-ESI** exact mass calcd. for $C_{35}H_{23}BrO_2Na$ ($[M+Na]^+$) requires m/z 577.0774, found m/z 577.0755.

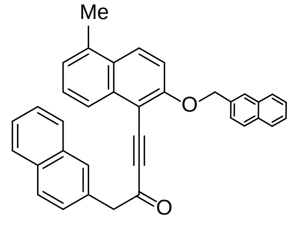

2i

White solid; **^{1}H NMR** (400 MHz, $CDCl_3$): δ (ppm) 7.97 (dt, *J* = 10.9, 1.2 Hz, 2H), 7.91 – 7.68 (m, 7H), 7.55 (dd, *J* = 8.5, 1.8 Hz, 2H), 7.52 – 7.40 (m, 5H), 7.22 (dt, *J* = 9.4, 1.2 Hz, 1H), 7.12 (dt, *J* = 6.9, 1.1 Hz, 1H), 6.99 (dd, *J* = 8.5, 7.0 Hz, 1H), 5.43 (s, 2H), 4.16 (s, 2H), 2.58 (s, 3H). **^{13}C NMR** (101 MHz, $CDCl_3$): δ (ppm) 185.4, 160.9, 135.3, 135.0, 134.1, 133.8, 133.5, 133.2, 132.8, 131.4, 129.7, 129.0, 128.6, 128.6, 128.2, 128.1, 128.1, 127.9, 127.9, 127.7, 126.5, 126.3, 126.3, 126.0, 126.0, 125.8, 124.7, 123.4, 113.6, 104.4, 98.0, 89.4, 71.3, 52.6, 19.4. **IR** (neat) 413, 441, 666, 775, 802, 855, 950, 1055, 1093, 1117, 1143, 1171, 1237, 1265, 1497, 1506, 1575, 1588, 1652, 2183 cm^{-1}. **HRMS-ESI** exact mass calcd. for $C_{36}H_{26}O_2Na$ ($[M+Na]^+$) requires m/z 513.1825, found m/z 513.1814.

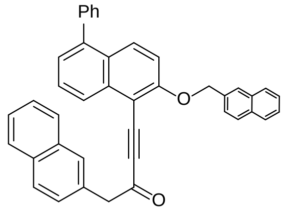

2j

Yellow solid; **^{1}H NMR** (400 MHz, $CDCl_3$): δ (ppm) 7.94 (d, *J* = 1.6 Hz, 1H), 7.91 – 7.73 (m, 8H), 7.67 (dt, *J* = 8.5, 1.0 Hz, 1H), 7.54 (dd, *J* = 8.5, 1.8 Hz, 1H), 7.51 – 7.38 (m, 8H), 7.36 (dd, *J* = 7.8, 1.8 Hz, 2H), 7.23 (dd, *J* = 7.1, 1.2 Hz, 1H), 7.17 – 7.01 (m, 2H), 5.42 (s, 2H), 4.18 (s, 2H). **^{13}C NMR** (101 MHz, $CDCl_3$): δ (ppm) 185.4, 161.0, 140.9, 140.2, 135.4, 134.0, 133.8, 133.5, 133.2, 132.8, 131.8, 131.4, 130.1, 129.1, 128.7, 128.6, 128.4, 128.2, 128.1, 128.0, 127.9, 127.9, 127.8, 127.6, 126.7, 126.5, 126.4, 126.3, 126.1, 126.0, 126.0, 124.7, 124.6, 113.9, 104.0, 98.1, 89.4, 71.2, 52.6. **IR** (neat) 413, 539, 547, 571, 603, 626, 667, 776, 857, 1018, 1044, 1057, 1086, 1114, 1126, 1136, 1233, 1257, 1275, 1507, 1634, 1645 cm^{-1}. **HRMS-ESI** exact mass calcd. for $C_{41}H_{28}O_2Na$ ($[M+Na]^+$) requires m/z 575.1982, found m/z

575.1963.

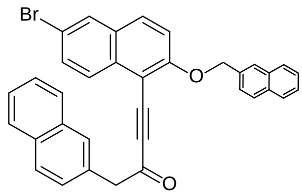

2k

Yellow solid; **^{1}H NMR** (400 MHz, $CDCl_3$): δ (ppm) 7.94 (d, *J* = 1.6 Hz, 1H), 7.91 – 7.74 (m, 8H), 7.68 (d, *J* = 9.2 Hz, 1H), 7.56 – 7.42 (m, 6H), 7.36 (d, *J* = 8.9 Hz, 1H), 7.22 (d, *J* = 9.2 Hz, 1H), 7.01 (dd, *J* = 8.9, 2.0 Hz, 1H), 5.41 (s, 2H), 4.14 (s, 2H); **^{13}C NMR** (101 MHz, $CDCl_3$): δ (ppm) 185.3, 161.2, 133.8, 133.4, 133.4, 133.2, 132.8, 132.2, 131.4, 131.3, 130.1, 129.4, 129.1, 128.7, 128.7, 128.2, 128.0, 127.9, 127.9, 127.9, 126.7, 126.6, 126.5, 126.4, 126.2, 126.0, 124.7, 118.7, 115.2, 104.2, 98.0, 88.2, 71.4, 52.5. **IR** (neat) 439, 476, 655, 671, 739, 760, 775, 824, 857, 882, 953, 1038, 1066, 1144, 1144, 1162, 1228, 1272, 1327, 1495, 1580, 1646, 2185 cm^{-1}. **HRMS-ESI** exact mass calcd. for $C_{35}H_{23}BrO_2Na$ ($[M+Na]^+$) requires m/z 577.0774, found m/z 577.0757.

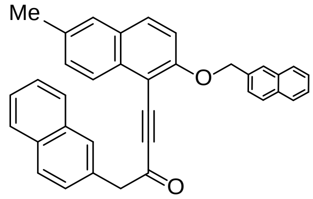

2l

Light yellow solid; **^{1}H NMR** (400 MHz, $CDCl_3$): δ (ppm) 7.96 (s, 1H), 7.91 – 7.75 (m, 7H), 7.71 (d, *J* = 9.1 Hz, 1H), 7.61 – 7.51 (m, 2H), 7.50 – 7.45 (m, 6H), 7.17 (d, *J* = 9.1 Hz, 1H), 6.95 (dd, *J* = 8.5, 1.6 Hz, 1H), 5.39 (s, 2H), 4.16 (s, 2H), 2.41 (s, 3H); **^{13}C NMR** (101 MHz, $CDCl_3$): δ (ppm) 185.3, 160.6, 134.5, 134.2, 133.8, 133.5, 133.2, 133.0, 132.8, 131.3, 130.5, 129.0, 128.7, 128.6, 128.2, 128.0, 127.9, 127.9, 127.2, 126.5, 126.3, 126.3, 126.0, 126.0, 124.8, 124.8, 114.2, 103.8, 97.9, 89.4, 71.4, 52.5, 21.4. **IR** (neat) 440, 476, 544, 667, 676, 687, 738, 757, 775, 823, 856, 883, 1035, 1044, 1144, 1229, 1278, 1647, 2180 cm^{-1}. **HRMS-ESI** exact mass calcd. for $C_{36}H_{26}O_2Na$ ($[M+Na]^+$) requires m/z 513.1825, found m/z 513.1813.

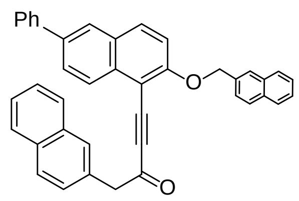

2m

Yellow solid; ^{1}**H NMR** (600 MHz, $CDCl_3$, 323 K): δ (ppm) 8.01 – 7.95 (m, 1H), 7.93 – 7.78 (m, 9H), 7.67 – 7.56 (m, 4H), 7.52 – 7.47 (m, 7H), 7.43 – 7.34 (m, 1H), 7.30 (dd, *J* = 8.7, 1.9 Hz, 1H), 7.28 (s, 2H), 5.47 (s, 2H), 4.18 (s, 2H); ^{13}**C NMR** (151 MHz, $CDCl_3$, 323 K): δ (ppm) 185.2, 161.3, 140.7, 137.8, 134.2, 134.2, 134.0, 133.6, 133.6, 133.4, 133.0, 131.6, 129.2, 129.0, 129.0, 128.7, 128.7, 128.3, 128.2, 128.1, 128.0, 127.9, 127.9, 127.6, 127.4, 126.5, 126.4, 126.4, 126.2, 126.1, 126.0, 125.6, 124.9, 115.0, 104.3, 98.1, 89.0, 71.8, 52.6. **IR** (neat) 434, 475, 546, 601, 669, 688, 741, 756, 775, 802, 824, 836, 1055, 1145, 1232, 1272, 1287, 1496, 1654, 2181 cm^{-1}. **HRMS-ESI** exact mass calcd. for $C_{41}H_{28}O_2Na$ ($[M+Na]^+$) requires m/z 575.1982, found m/z 575.1966.

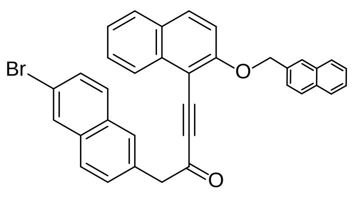

2n

White solid; ^{1}**H NMR** (400 MHz, $CDCl_3$): δ (ppm) 7.93 (d, *J* = 9.5 Hz, 2H), 7.84 (dd, *J* = 11.1, 6.9 Hz, 4H), 7.73 (q, *J* = 8.3 Hz, 3H), 7.65 (d, *J* = 8.4 Hz, 1H), 7.62 – 7.53 (m, 2H), 7.53 – 7.42 (m, 4H), 7.36 – 7.32 (m, 1H), 7.23 (td, *J* = 7.9, 7.0, 1.3 Hz, 2H), 5.43 (s, 2H), 4.13 (s, 2H); ^{13}**C NMR** (101 MHz, $CDCl_3$): δ (ppm) 184.9, 161.3, 134.9, 134.0, 133.8, 133.6, 133.4, 133.2, 132.1, 131.9, 129.9, 129.7, 129.5, 129.1, 128.9, 128.7, 128.5, 128.4, 128.3, 128.2, 127.9, 127.6, 126.6, 126.4, 126.0, 125.0, 124.9, 124.7, 119.9, 114.1, 103.8, 98.0, 89.3, 71.4, 52.4. **IR** (neat) 440, 475, 550, 674, 742, 768, 803, 826, 855, 882, 1049, 1137, 1147, 1229, 1276, 1506, 1587, 1652, 2178 cm^{-1}. **HRMS-ESI** exact mass calcd. for $C_{35}H_{23}BrO_2Na$ ($[M+Na]^+$) requires m/z 577.0774, found m/z 577.0756.

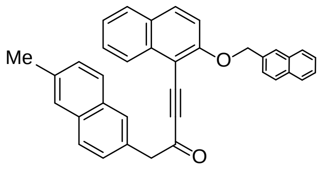

2o

White solid; **^{1}H NMR** (400 MHz, $CDCl_3$): δ (ppm) 7.94 (s, 1H), 7.90 – 7.73 (m, 6H), 7.74 – 7.63 (m, 4H), 7.59 – 7.51 (m, 2H), 7.51 – 7.46 (m, 2H), 7.43 (dd, *J* = 8.3, 1.8 Hz, 1H), 7.32 – 7.27 (m, 2H), 7.21 (d, *J* = 9.1 Hz, 1H), 7.16 – 7.13 (m, 1H), 5.43 (s, 2H), 4.14 (s, 2H), 2.48 (s, 3H); **^{13}C NMR** (101 MHz, $CDCl_3$): δ (ppm) 185.5, 161.2, 135.7, 134.9, 134.1, 133.5, 133.4, 133.2, 133.0, 132.0, 130.4, 128.8, 128.6, 128.6, 128.5, 128.3, 128.2, 128.1, 128.0, 127.9, 127.7, 126.8, 126.5, 126.3, 126.0, 125.0, 124.9, 124.7, 114.2, 104.0, 98.1, 89.0, 71.4, 52.5, 21.8. **IR** (neat) 421, 441, 472, 553, 666, 711, 740, 749, 771, 802, 815, 825, 852, 884, 1048, 1136, 1147, 1219, 1227, 1276, 1506, 1652, 2180 cm^{-1}. **HRMS-ESI** exact mass calcd. for $C_{36}H_{26}O_2Na$ ($[M+Na]^+$) requires m/z 513.1825, found m/z 513.1814.

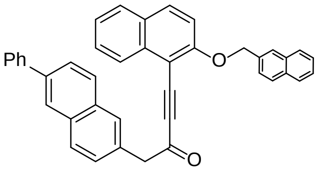

2p

Yellow solid; **^{1}H NMR** (400 MHz, $CDCl_3$): δ (ppm) 7.98 – 7.94 (m, 2H), 7.91 – 7.77 (m, 7H), 7.73 – 7.66 (m, 5H), 7.54 (dd, *J* = 8.4, 1.8 Hz, 1H), 7.51 – 7.43 (m, 5H), 7.43 – 7.34 (m, 1H), 7.31 – 7.27 (m, 1H), 7.21 (d, *J* = 9.2 Hz, 1H), 7.13 – 7.17 (m, 1H), 5.43 (s, 2H), 4.17 (s, 2H). **^{13}C NMR** (101 MHz, $CDCl_3$): δ (ppm) 185.3, 161.2, 141.2, 138.7, 134.9, 134.1, 133.5, 133.4, 133.2, 133.0, 132.9, 131.5, 129.0, 128.9, 128.8, 128.6, 128.5, 128.5, 128.5, 128.3, 128.2, 127.9, 127.5, 127.5, 126.5, 126.3, 126.1, 126.0, 125.8, 125.0, 124.9, 124.7, 114.2, 103.9, 98.1, 89.2, 71.4, 52.6. **IR** (neat) 413, 440, 473, 517, 549, 623, 651, 672, 696, 741, 751, 814, 826, 855, 1048, 1137, 1147, 1229, 1277, 1506, 1652, 2180 cm^{-1}. **HRMS-ESI** exact mass calcd. for $C_{41}H_{28}O_2Na$ ($[M+Na]^+$) requires m/z 575.1982, found m/z 575.1975.

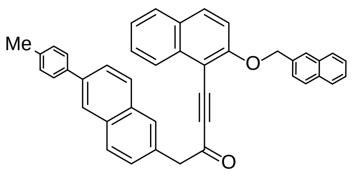

2q

Light yellow solid; **^{1}H NMR** (400 MHz, $CDCl_3$): δ (ppm) 8.00 – 7.91 (m, 2H), 7.90 – 7.84 (m, 2H), 7.82 (d, *J* = 7.8 Hz, 5H), 7.78 (s, 1H), 7.74 – 7.64 (m, 3H), 7.61 – 7.56 (m, 2H), 7.54 (dd, *J* = 8.5, 1.8 Hz, 1H), 7.51 – 7.43 (m, 3H), 7.34 – 7.27 (m, 3H), 7.20 (d, *J* = 8.8 Hz, 1H), 7. 16 – 7.14 (m, 1H), 5.41 (s, 2H), 4.17 (s, 2H), 2.44 (s, 3H); **^{13}C NMR** (101 MHz, $CDCl_3$): δ (ppm) 185.4, 161.2, 138.7, 138.2, 137.3, 134.9, 134.0, 133.5, 133.4, 133.2, 133.1, 132.8, 131.3, 129.7, 128.8, 128.8, 128.6, 128.5, 128.4, 128.3, 128.2, 127.9, 127.3, 126.5, 126.3, 126.0, 126.0, 125.4, 125.0, 124.9, 124.7, 114.1, 103.9, 98.1, 89.2, 71.3, 52.5, 21.3. **IR** (neat) 412, 439, 475, 660, 688, 741, 750, 776, 802, 811, 825, 1048, 1137, 1147, 1226, 1254, 1278, 1497, 1506, 1656, 2181 cm^{-1}. **HRMS-ESI** exact mass calcd. for $C_{42}H_{30}O_2Na$ ($[M+Na]^+$) requires m/z 589.2138, found m/z 589.2126.

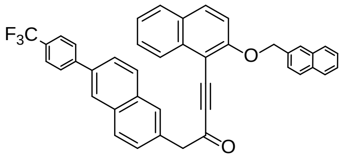

2r

Yellow solid; **^{1}H NMR** (400 MHz, $CDCl_3$): δ (ppm) 7.96 (d, *J* = 1.8 Hz, 1H), 7.92 (d, *J* = 1.6 Hz, 1H), 7.89 – 7.77 (m, 8H), 7.77 – 7.63 (m, 7H), 7.54 – 7.50 (m, 2H), 7.49 – 7.43 (m, 2H), 7.31 – 7.28 (m, 1H), 7.24 – 7.15 (m, 2H), 5.42 (s, 2H), 4.18 (s, 2H); **^{13}C NMR** (101 MHz, $CDCl_3$): δ (ppm) 185.1, 161.2, 144.6, 137.2, 134.9, 134.0, 133.5, 133.4, 133.3, 133.2, 132.9, 132.1, 129.7, 129.4, 129.0, 128.8, 128.8, 128.8, 128.6, 128.5, 128.3, 128.3, 128.2, 127.9, 127.7, 126.5, 126.3, 126.3, 126.0, 125.9, 125.9, 125.8, 125.6, 125.0, 124.9, 124.7, 114.1, 103.9, 98.1, 89.2, 71.3, 52.5. **^{19}F NMR** (376 MHz, $CDCl_3$) δ (ppm) -62.4. **IR** (neat) 419, 445, 476, 549, 600, 726, 744, 777, 808, 827, 849, 861, 896, 1012, 1025, 1054,

1072, 1108, 1154, 1227, 1254, 1275, 1331, 1656, 2175. cm^{-1} **HRMS-ESI** exact mass calcd. for $C_{42}H_{27}F_3O_2Na$ ($[M+Na]^+$) requires m/z 643.1855, found m/z 643.1840.

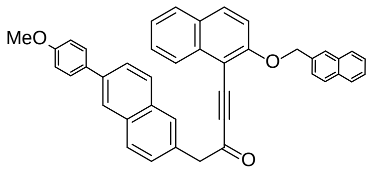

2s

White solid; ^{1}**H NMR** (400 MHz, $CDCl_3$): δ (ppm) 7.98 – 7.90 (m, 2H), 7.90 – 7.77 (m, 7H), 7.69 (td, *J* = 8.8, 8.4, 1.4 Hz, 3H), 7.65 – 7.58 (m, 2H), 7.53 (dd, *J* = 8.5, 1.7 Hz, 1H), 7.51 – 7.42 (m, 3H), 7.33 – 7.27 (m, 1H), 7.21 (d, *J* = 9.2 Hz, 1H), 7. 17 – 7.17 (m, 1H), 7.05 – 6.95 (m, 2H), 5.42 (s, 2H), 4.16 (s, 2H), 3.88 (s, 3H); ^{13}**C NMR** (101 MHz, $CDCl_3$): δ (ppm) 185.4, 161.2, 159.4, 138.3, 134.9, 134.1, 133.7, 133.5, 133.4, 133.2, 133.1, 132.6, 131.2, 128.8, 128.8, 128.6, 128.5, 128.5, 128.4, 128.3, 128.2, 127.9, 126.5, 126.3, 126.0, 125.9, 125.0, 124.9, 124.7, 114.5, 114.2, 103.9, 98.1, 89.1, 71.3, 55.5, 52.6. **IR** (neat) 411, 420, 439, 475, 741, 752, 812, 825, 1048, 1139, 1148, 1218, 1227, 1278, 1364, 1507, 1654, 1738, 2181, 2359 cm^{-1}. **HRMS-ESI** exact mass calcd. for $C_{42}H_{30}O_3Na$ ($[M+Na]^+$) requires m/z 605.2087, found m/z 605.2075.

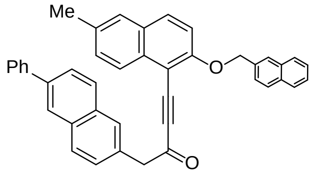

2t

White solid; ^{1}**H NMR** (400 MHz, $CDCl_3$): δ (ppm) 8.00 – 7.91 (m, 2H), 7.91 – 7.78 (m, 6H), 7.78 – 7.63 (m, 4H), 7.54 (dd, *J* = 8.5, 1.3 Hz, 2H), 7.51 – 7.43 (m, 6H), 7.43 – 7.33 (m, 1H), 7.17 (d, *J* = 9.1 Hz, 1H), 6.93 (dd, *J* = 8.6, 1.7 Hz, 1H), 5.40 (s, 2H), 4.16 (s, 2H), 2.37 (s, 3H); ^{13}**C NMR** (101 MHz, $CDCl_3$): δ (ppm) 185.4, 160.7, 141.2, 138.8, 134.5, 134.2, 133.5, 133.2, 133.1, 133.0, 133.0, 132.8, 131.5, 130.5, 129.0, 128.9, 128.8, 128.7, 128.6, 128.6, 128.5, 128.2, 127.9, 127.5, 127.5, 127.2, 126.5, 126.3, 126.1, 126.0, 125.8, 124.8,

124.8, 114.2, 103.8, 97.9, 89.5, 71.4, 52.5, 21.5. **IR** (neat) 418, 440, 544, 676, 694, 750, 764, 855, 881, 1041, 1044, 1228, 1279, 1333, 1373, 1497, 1591, 1738, 2182 cm^{-1}. **HRMS-ESI** exact mass calcd. for $C_{42}H_{31}O_2Na$ ($[M+H]^+$) requires m/z 567.2319, found m/z 567.2302.

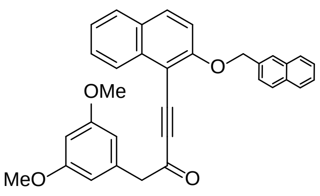

2u

Yellow solid; **^{1}H NMR** (400 MHz, $CDCl_3$): δ (ppm) 8.00 – 7.91 (m, 2H), 7.91 – 7.80 (m, 4H), 7.74 (d, *J* = 8.1 Hz, 1H), 7.58 (dd, *J* = 8.5, 1.7 Hz, 1H), 7.55 – 7.43 (m, 3H), 7.41 – 7.37 (m, 1H), 7.24 (s, 1H), 6.55 (d, *J* = 2.2 Hz, 2H), 6.40 (s, 1H), 5.51 (s, 2H), 3.95 (s, 2H), 3.72 (s, 6H); **^{13}C NMR** (101 MHz, $CDCl_3$): δ (ppm) 185.0, 161.2, 161.2, 135.7, 135.0, 134.1, 133.5, 133.4, 133.2, 128.6, 128.6, 128.4, 128.4, 128.2, 127.9, 126.5, 126.3, 126.0, 125.1, 125.0, 124.7, 114.3, 108.1, 104.1, 99.7, 97.9, 88.7, 71.4, 55.4, 52.7. **IR** (neat) 478, 592, 664, 741, 773, 801, 812, 1046, 1064, 1083, 1122, 1148, 1203, 1234, 1274, 1592, 1635. **HRMS-ESI** exact mass calcd. for $C_{36}H_{27}O_4$ ($[M+H]^+$) requires m/z 487.1904, found m/z 487.1892.

General procedure for the synthesis of **2c** and **2d**[3]

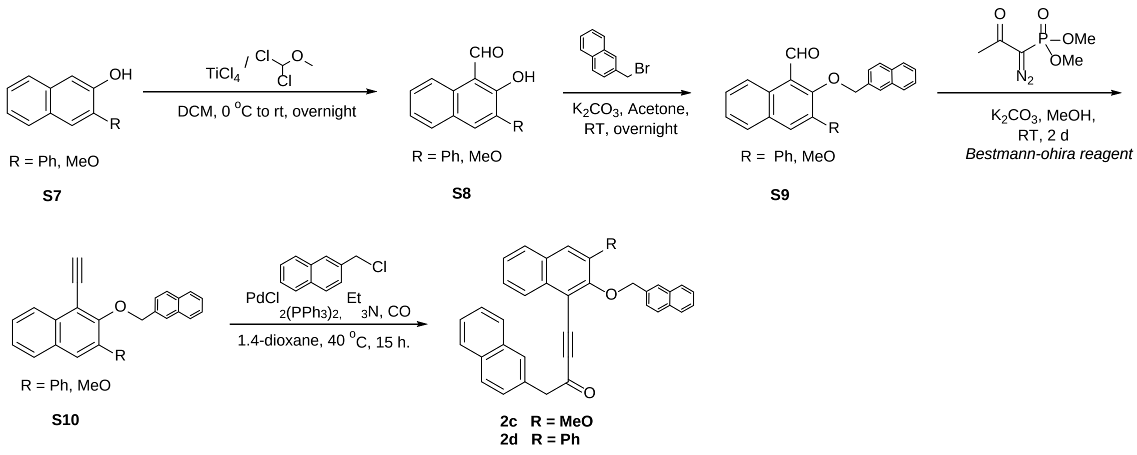

A solution of $TiCl_4$ (15.4 mmol, 2.0 eq.) and dichloromethyl methylether (7.7 mmol, 1.0 eq.) in anhydrous CH_2Cl_2 (10mL) was stirred at 0 °C for 15 min. After this, a solution of the corresponding substituted naphthol **S7** (7.7 mmol, 1.0 eq.) in CH_2Cl_2 (20 mL) was added dropwise, and the reaction allowed to warm to room temperature. The mixture obtained was stirred overnight and subsequently, it was quenched by adding 1 N HCl (10 mL). The aqueous layer was extracted with CH_2Cl_2 (3 × 10 mL), and the combined organic phases dried with Na_2SO_4, and evaporated to afford a residue which was further purified by column chromatography (hexanes/acetone = 10:1, v/v) to yield the corresponding aldehydes **S8**.

After this, K_2CO_3 (455 mg, 3.3 mmol, 1.10 eq.) was added to a solution of **S8** (3.0 mmol, 1.00 eq.) and 2-naphtylmethyl bromide (3.3 mmol, 1.10 eq.) in acetone (30 mL) and the resulting mixture stirred at room temperature for 13 h . Filtration of the reaction mixture produced a transparent organic phase that was concentrated in vacuo. The residue thus obtained was purified by a silica gel column chromatography (hexane/acetone = 40:1, v/v) affording **S9**.

To a flame dried, argon purged round bottom flask was added the aldehyde **S9** (0.7 mmol,

1.00 eq.) dissolved in anhydrous methanol (10 mL) and K_2CO_3 (1.4 mmol, 2.00 eq.). Then, Bestmann-Ohira reagent (0.84 mmol, 1.20 eq.) was added slowly via a syringe and the reaction mixture thus obtained was stirred at room temperature for 2 days. After that, the solvent was removed under reduced pressure, water (10 mL) was added and the suspension obtained was extracted with ethyl acetate (15 mL) three times. Concentration of the organic phases was under reduced pressure afforded a residue, which was purified by a silica gel flash column chromatography (hexane/acetone = 20:1, v/v) to obtain the desired product **S10**.

Finally, $PdCl_2(PPh_3)_2$ (0.13 mmol, 0.05 eq.), 2-(chloromethyl)naphthalene (0.56 mmol, 1.00 eq.), **S10** (0.62 mmol, 1.10 eq.) and Et_3N (0.84 mmol, 1.50 eq.) were suspended in 1,4-dioxane (5 mL) under a N_2 atmosphere, the flask purged with CO (1 atm) three times, and heated to 40 °C for 15 h. After this the flask was allowed to cool to room temperature and the remaining CO was vented. The reaction was quenched with water and extracted with CH_2Cl_2. After washing the organic layer with brine, it was dried over Na_2SO_4 and concentrated. The residue was purified by a silica gel column chromatography (CH_2Cl_2/MeOH = 150:1, v/v) to give the desired alkynone products **2c** and **2d**.

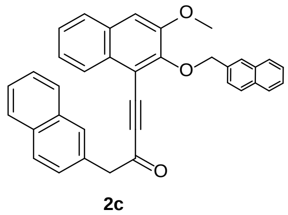

2c

White solid; **^{1}H NMR** (400 MHz, $CDCl_3$): δ (ppm) 7.98 (d, *J* = 1.6 Hz, 1H), 7.90 – 7.74 (m, 6H), 7.72 (d, *J* = 1.7 Hz, 1H), 7.68 (dd, *J* = 8.4, 1.7 Hz, 1H), 7.64 (d, *J* = 8.1 Hz, 1H), 7.50 – 7.46 (m, 5H), 7.39 – 7.34 (m, 1H), 7.24 (s, 1H), 6.96 – 6.91 (m, 1H), 5.38 (s, 2H), 4.02 (s, 2H), 3.98 (s, 3H). **^{13}C NMR** (101 MHz, $CDCl_3$): δ (ppm) 185.1, 153.4, 151.8, 134.5, 133.7, 133.4, 133.4, 132.8, 131.0, 130.6, 129.3, 129.0, 128.6, 128.3, 128.3, 127.9, 127.9, 127.8,

127.7, 126.8, 126.6, 126.4, 126.2, 126.2, 126.1, 125.5, 125.2, 111.1, 110.7, 96.7, 88.1, 76.4, 56.0, 52.4. **IR** (neat) 4768, 664, 745, 829, 853, 950, 1001, 1012, 1151, 1165, 1203, 1215, 1227, 1255, 1339, 1366, 1425, 1456, 1650, 2181 cm^{-1}. **HRMS-ESI** exact mass calcd. for $C_{26}H_{36}O_3Na$ ([M+Na]$^+$) requires m/z 529.1774, found m/z 529.1761.

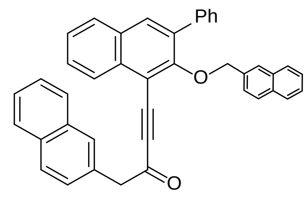

2d

White solid; **^{1}H NMR** (400 MHz, $CDCl_3$): δ (ppm) 7.89 (s, 1H), 7.87 – 7.75 (m, 7H), 7.73 (d, *J* = 8.4 Hz, 1H), 7.67 – 7.58 (m, 3H), 7.54 (d, *J* = 8.4 Hz, 1H), 7.52 – 7.34 (m, 9H), 7.32 – 7.26 (m, 1H), 7.05 – 7.03 (m, 1H), 4.90 (s, 2H), 4.09 (s, 2H); **^{13}C NMR** (101 MHz, $CDCl_3$): δ (ppm) 185.1, 159.9, 137.5, 135.4, 134.0, 133.8, 133.8, 133.7, 133.3, 132.8, 131.1, 130.3, 129.7, 129.1, 128.7, 128.6, 128.3, 128.3, 128.1, 128.1, 128.0, 128.0, 128.0, 127.9, 127.8, 127.8, 126.7, 126.4, 126.3, 126.2, 126.1, 125.2, 110.4, 110.1, 97.1, 88.6, 52.4. **IR** (neat) 420, 478, 517, 667, 696, 751, 828, 991, 1202, 1216, 1227, 1365, 1426, 1457, 1490, 1507, 1649, 2181, 2342, 2359 cm^{-1}. **HRMS-ESI** exact mass calcd. for $C_{41}H_{28}O_2Na$ ([M+Na]$^+$) requires m/z 575.1982, found m/z 575.1964.

4. General procedure for gold-catalyzed atroposelective intramolecular hydroarylation of alkynones

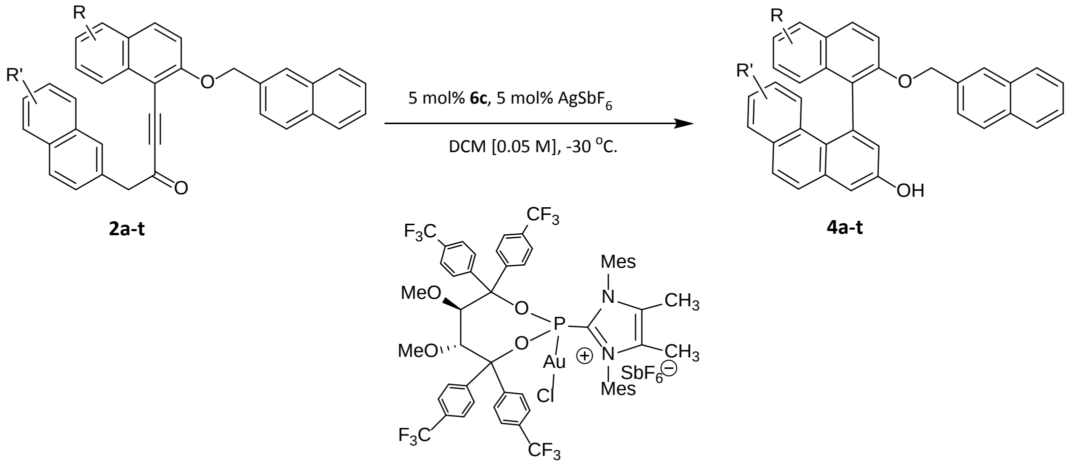

To a dried Schlenk flask equipped with magnetic stirrer substrate **2** (1.0 equiv.) and gold precatalyst **6c** (5 mol%) were added. A septum was fitted to the flask and the content was dried under high vacuum for 30 minutes. Then dichloromethane (0.05M) was added via syringe and the reaction mixture was allowed to stir at room temperature for 2 minutes before transferring it to a pre-cooled isopropanol bath at -30 °C. After this, a solution of $AgSbF_6$ (5 mol%, 0.05M solution in dichloromethane) was added through the septum, the septum was exchanged for a greased glass stopper and the reaction mixture was allowed to stir at -30 °C for the indicated time. Once the starting material was consumed, the reaction mixture was filtered through a short pad of silica eluting with dichloromethane, and the solvent was removed in vacuo. The obtained residue was purified by column chromatography on silica gel (*n*-hexanes/acetone =7:1, v/v) to afford the desired product **4a-t**.

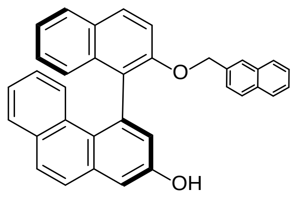

4a

Yellow solid; isolated yield 90%, 97% ee. $[\alpha]_D^{20}$ = -64.1 (c = 1.1, $CHCl_3$); **^{1}H NMR** (400 MHz, $CDCl_3$): δ (ppm) 7.97 (d, J = 9.0 Hz, 1H), 7.89 (d, J = 8.2 Hz, 1H), 7.77-7.67 (m, 4H), 7.53 (q, J = 7.4, 6.7 Hz, 3H), 7.47-7.34 (m, 6H), 7.28 (dd, J = 12.7, 3.9 Hz, 3H), 7.19 (s, 1H), 6.94 (d, J = 2.7 Hz, 1H), 6.88 (t, J = 8.1 Hz, 2H), 5.16 (d, J = 12.6 Hz, 1H), 5.02 (d, J = 12.5 Hz, 1H), 4.94 (s, 1H); **^{13}C NMR** (101MHz, $CDCl_3$): δ (ppm) 153.2, 152.5, 136.4, 135.5, 134.6, 133.4, 133.2, 132.9, 132.3, 131.3, 129.9, 129.5, 128.6, 128.3, 128.0, 127.9, 127.6, 127.3, 126.9, 126.1, 126.0, 125.9, 125.8, 125.7, 125.6, 125.2, 125.0, 124.9, 124.3, 121.6, 116.2, 112.6, 71.3. . **IR** (neat) 413, 473, 504, 580, 720, 745, 809, 857, 996, 1070, 1146, 1174, 1220, 1267, 1327, 1600 cm^{-1}. **HRMS-ESI** exact mass calcd. for $C_{35}H_{24}O_2Na$ ($[M+Na]^+$) requires m/z 499.1669, found m/z 499.1665.

The enantiomeric excess was determined by HPLC on Chiralcel IC-3 column (hexane : isopropanol = 95 : 5, flowing rate = 1.0 mL/min, 30 ^oC, UV detection at λ = 254 nm), t_{R1} = 9.0 min (minor), t_{R2} = 10.1 min (major).

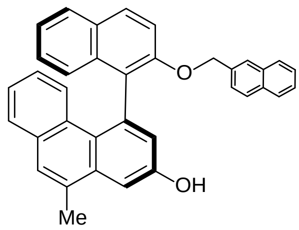

4b

White solid; isolated yield 92%, 96% ee. $[\alpha]_D^{20}$ = -59 (c = 1.1, $CHCl_3$); **^{1}H NMR** (400 MHz, $CDCl_3$): δ (ppm) 7.95 (d, J = 8.9 Hz, 1H), 7.88 (d, J = 8.2 Hz, 1H), 7.80 – 7.63 (m, 2H), 7.63 – 7.30 (m, 11H), 7.26 – 7.19 (m, 2H), 6.95 (d, J = 2.9 Hz, 1H), 6.91 – 6.73 (m, 2H), 5.14 (d, J = 12.7 Hz, 1H), 5.00 (d, J = 12.5 Hz, 1H), 4.94 (s, 1H), 2.75 (s, 3H); **^{13}C NMR** (101MHz, $CDCl_3$): δ (ppm) 153.2, 152.4, 136.7, 135.6, 134.7, 133.3, 133.2, 132.8, 132.1, 131.9, 130.6, 129.9, 129.4, 129.0, 128.5, 128.0, 127.9, 127.9, 127.6, 126.9, 126.0, 125.9,

125.7, 125.7, 125.6, 125.3, 125.3, 125.2, 124.9, 124.3, 121.1, 116.2, 109.0, 71.2, 21.0. **IR** (neat) 418, 450, 473, 519, 578, 729, 746, 809, 855, 885, 951, 1003, 1048, 1183, 1222, 1267, 1328, 1599 cm^{-1}. **HRMS-ESI** exact mass calcd. for $C_{36}H_{26}O_2$ ($[M+H]^+$) requires m/z 490.1927, found m/z 490.1925.

The enantiomeric excess was determined by HPLC on Chiralcel IA-3 column (hexane : isopropanol = 80 : 20, flowing rate = 1.0 mL/min, 25 ^{o}C, UV detection at λ = 254 nm), t_{R1} = 5.2 min (major), t_{R2} = 9.0 min (minor).

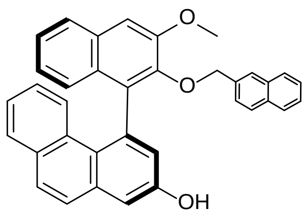

4c

Yellow solid; isolated yield 92%, 86% ee. $[\alpha]_D^{20}$ = -104 (*c* = 1.6, $CHCl_3$); **^{1}H NMR** (400 MHz, $CDCl_3$): δ (ppm) 7.84 (d, *J* = 8.3 Hz, 1H), 7.76 – 7.60 (m, 4H), 7.50 (q, *J* = 9.2 Hz, 3H), 7.43 – 7.34 (m, 4H), 7.34 – 7.27 (m, 2H), 7.14 – 7.10 (m, 1H), 7.05 (s, 1H), 6.95 – 6.81 (m, 2H), 6.76 (dd, *J* = 8.4, 1.6 Hz, 1H), 5.02 (d, *J* = 11.1 Hz, 1H), 4.75 (s, 1H), 4.72 (d, *J* = 11.1 Hz, 1H), 4.10 (s, 3H); **^{13}C NMR** (101MHz, $CDCl_3$): δ (ppm) 153.0, 153.0, 144.8, 135.8, 135.5, 134.9, 134.7, 133.1, 132.9, 132.3, 131.9, 130.9, 128.7, 128.4, 128.3, 128.1, 127.6, 127.5, 127.1, 126.8, 126.7, 126.1, 125.9, 125.8, 125.8, 125.8, 125.2, 124.8, 124.6, 121.5, 112.8, 107.3, 74.5, 55.9. **IR** (neat) 413, 433, 444, 472, 503, 580, 630, 721, 743, 812, 857, 997, 1045, 1113, 1150, 1174, 1194, 1245, 1423, 1459, 1599 cm^{-1}. **HRMS-ESI** exact mass calcd. for $C_{36}H_{26}O_3$ ($[M+H]^+$) requires m/z 506.1876, found m/z 499.1872.

The enantiomeric excess was determined by HPLC on Chiralcel IA-3 column (hexane : isopropanol = 70 : 30, flowing rate = 1.0 mL/min, 25 ^{o}C, UV detection at λ = 254 nm), t_{R1} = 4.1 min (minor), t_{R2} = 13.1 min (major).

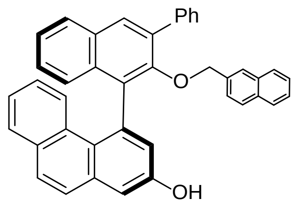

4d

White solid; isolated yield 87%, 97% ee. $[\alpha]_D^{20}$ = -195 (*c* = 1.2, $CHCl_3$); **^{1}H NMR** (400 MHz, $CDCl_3$): δ (ppm) 8.06 (s, 1H), 7.97 (d, *J* = 8.3 Hz, 1H), 7.84 – 7.53 (m, 7H), 7.51 – 7.26 (m, 12H), 7.02 (t, *J* = 2.1 Hz, 1H), 6.96 (dd, *J* = 8.8, 7.0 Hz, 1H), 6.62 (s, 1H), 6.35 (dd, *J* = 8.5, 1.9 Hz, 1H), 4.90 (s, 1H), 4.31 (d, *J* = 10.5 Hz, 1H), 4.22 (d, *J* = 10.5 Hz, 1H); **^{13}C NMR** (101MHz, $CDCl_3$): δ (ppm) 153.1, 151.1, 138.9, 136.3, 136.0, 135.6, 134.2, 134.1, 133.0, 132.8, 132.8, 132.4, 131.4, 131.1, 130.4, 129.7, 128.8, 128.5, 128.5, 128.2, 128.0, 127.6, 127.5, 127.5, 127.2, 127.2, 126.8, 126.1, 126.1, 125.9, 125.8, 125.7, 125.7, 125.3, 124.9, 121.7, 112.8, 74.4. **IR** (neat) 410, 434, 472, 504, 556, 580, 634, 699, 718, 746, 812, 855, 893, 994, 1149, 1189, 1255, 1560 cm^{-1}. **HRMS-ESI** exact mass calcd. for $C_{41}H_{28}O_2Na$ ($[M+Na]^+$) requires m/z 575.1982, found m/z 575.1982.

The enantiomeric excess was determined by HPLC on Chiralcel IC-3 column (hexane : isopropanol = 98 : 2, flowing rate = 1.0 mL/min, 25 °C, UV detection at λ = 254 nm), t_{R1} = 6.6 min (minor), t_{R2} = 8.7 min (major).

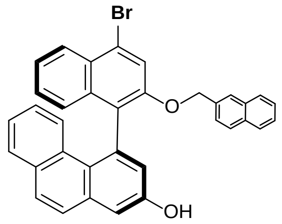

4e

White solid; isolated yield 90%, 97% ee. $[\alpha]_D^{20}$ = -88 (*c* = 1.7, $CHCl_3$); **^{1}H NMR** (400 MHz, $CDCl_3$): δ (ppm) 8.33 – 8.21 (m, 1H), 7.82 (s, 1H), 7.79 – 7.65 (m, 4H), 7.61 – 7.43 (m, 6H), 7.40 (dt, *J* = 6.5, 3.5 Hz, 2H), 7.36 – 7.27 (m, 3H), 7.15 (s, 1H), 6.98 – 6.86 (m, 2H), 6.82 (d, *J* = 8.5 Hz, 1H), 5.14 (d, *J* = 12.3 Hz, 1H), 5.00 (d, *J* = 12.2 Hz, 1H), 4.85 (s, 1H); **^{13}C NMR** (101MHz, $CDCl_3$): δ (ppm) 153.1, 152.2, 135.5, 135.5, 134.0, 134.0, 133.1, 132.9, 132.3, 131.1, 128.9, 128.7, 128.5, 128.5, 128.1, 128.0, 127.7, 127.7, 127.3, 127.2, 126.3, 126.1, 126.0, 125.9, 125.8, 125.4, 125.4, 124.9, 124.8, 123.4, 121.6, 120.5, 112.9, 71.6. **IR**

(neat) 473, 506, 580, 719, 744, 813, 855, 912, 995, 1158, 1174, 1219, 1261, 1312, 1372, 1579, 1600, 1739 cm^{-1}. **HRMS-ESI** exact mass calcd. for $C_{35}H_{23}BrO_2$ ($[M+H]^+$) requires m/z 554.0876, found m/z 554.0870.

The enantiomeric excess was determined by HPLC on Chiralcel IA-3 column (hexane : isopropanol = 80 : 20, flowing rate = 1.0 mL/min, 25 ^oC, UV detection at λ = 254 nm), t_{R1} = 4.5 min (minor), t_{R2} = 14.4 min (major).

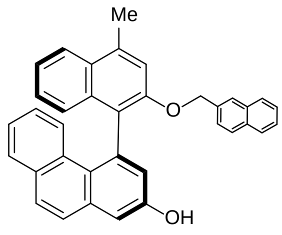

4f

White solid; isolated yield 90%, 93% ee. $[\alpha]_D^{20}$ = -105 (*c* = 0.8, $CHCl_3$); **^{1}H NMR** (400 MHz, $CDCl_3$): δ (ppm) 8.05 (d, *J* = 8.5 Hz, 1H), 7.76 (dd, *J* = 7.7, 1.5 Hz, 1H), 7.67 (dt, *J* = 25.5, 8.6 Hz, 4H), 7.57 – 7.44 (m, 3H), 7.44 – 7.35 (m, 3H), 7.35 – 7.25 (m, 4H), 7.16 (s, 1H), 6.94 – 6.80 (m, 3H), 5.14 (d, *J* = 12.4 Hz, 1H), 4.99 (d, *J* = 12.4 Hz, 1H), 4.89 (s, 1H), 2.83 (s, 3H); **^{13}C NMR** (101MHz, $CDCl_3$): δ (ppm) 153.1, 152.0, 136.6, 136.3, 135.5, 134.7, 133.5, 133.2, 132.9, 132.3, 131.4, 129.2, 128.6, 128.2, 128.0, 127.9, 127.6, 127.3, 126.9, 126.7, 126.4, 126.1, 126.0, 125.9, 125.6, 125.2, 125.1, 125.0, 124.3, 124.2, 121.9, 117.3, 112.5, 71.4, 20.2. **IR** (neat) 412, 422, 474, 510, 720, 746, 812, 857, 996, 1123, 1175, 1200, 1217, 1228, 1337, 1365, 1597, 1739 cm^{-1}. **HRMS-ESI** exact mass calcd. for $C_{36}H_{26}O_2$ ($[M+H]^+$) requires m/z 490.1927, found m/z 490.1926.

The enantiomeric excess was determined by HPLC on Chiralcel IA-3 column (hexane : isopropanol = 80 : 20, flowing rate = 1.0 mL/min, 25 ^oC, UV detection at λ = 254 nm), t_{R1} = 6.5 min (major), t_{R2} = 12.5 min (minor).

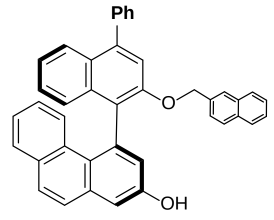

4g

White solid; isolated yield 93%, 96% ee. $[\alpha]_D^{20}$ = -97 (c = 1.7, $CHCl_3$); **^{1}H NMR** (400 MHz, $CDCl_3$): δ (ppm) 7.95 (d, J = 8.3 Hz, 1H), 7.82 – 7.66 (m, 5H), 7.65 – 7.47 (m, 8H), 7.45 (s, 1H), 7.43 – 7.35 (m, 2H), 7.35 – 7.27 (m, 4H), 7.15 (s, 1H), 7.00 (d, J = 2.7 Hz, 1H), 6.97 – 6.88 (m, 1H), 6.83 (dd, J = 8.4, 1.6 Hz, 1H), 5.17 (d, J = 12.2 Hz, 1H), 5.02 (d, J = 12.3 Hz, 1H), 4.86 (s, 1H); **^{13}C NMR** (101MHz, $CDCl_3$): δ (ppm) 153.2, 151.9, 142.0, 140.7, 136.4, 135.5, 134.5, 133.8, 133.2, 132.9, 132.3, 131.4, 130.4, 128.7, 128.6, 128.3, 128.2, 128.0, 127.9, 127.7, 127.6, 127.3, 126.9, 126.3, 126.2, 126.0, 125.9, 125.6, 125.3, 125.1, 125.1, 124.4, 121.8, 117.3, 112.6, 71.4. **IR** (neat) 403, 429, 443, 473, 582, 702, 721, 745, 811, 857, 996, 1149, 1174, 1197, 1335, 1600 cm^{-1}. **HRMS-ESI** exact mass calcd. for $C_{41}H_{28}O_2$ ($[M+H]^+$) requires m/z 552.2084, found m/z 552.2084.

The enantiomeric excess was determined by HPLC on Chiralcel IA-3 column (hexane : isopropanol = 80 : 20, flowing rate = 1.0 mL/min, 25 ^oC, UV detection at λ = 254 nm), t_{R1} = 5.3 min (major), t_{R2} = 8.6 min (minor).

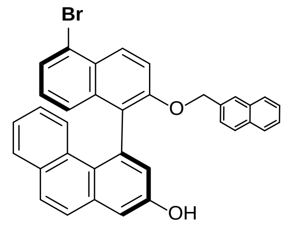

4h

White solid; isolated yield 87%, 95% ee. $[\alpha]_D^{20}$ = -131 (c = 1.0, $CHCl_3$); **^{1}H NMR** (400 MHz, $CDCl_3$): δ (ppm) δ 8.39 (d, J = 9.4 Hz, 1H), 7.81 – 7.60 (m, 5H), 7.60 – 7.43 (m, 4H), 7.43 – 7.34 (m, 3H), 7.34 – 7.27 (m, 2H), 7.20 (s, 1H), 7.06 (dd, J = 8.5, 7.4 Hz, 1H), 6.92 – 6.85 (m, 3H), 5.19 (d, J = 12.6 Hz, 1H), 5.06 (d, J = 12.6 Hz, 1H), 4.93 (s, 1H); **^{13}C NMR** (101MHz, $CDCl_3$): δ (ppm) 153.2, 153.1, 135.9, 135.5, 134.6, 134.3, 133.1, 132.9, 132.3, 131.2, 128.9, 128.8, 128.7, 128.4, 128.4, 128.2, 128.0, 127.7, 127.3, 127.2, 126.3, 126.1, 126.0, 125.9, 125.8, 125.4, 124.8, 124.8, 123.0, 121.6, 117.0, 112.8, 71.2. **IR** (neat) 408, 474,

580, 687, 723, 744, 803, 856, 996, 1043, 1118, 1175, 1198, 1228, 1262, 1600, 1611 cm^{-1}. **HRMS-ESI** exact mass calcd. for $C_{35}H_{23}BrO_2$ ($[M+H]^+$) requires m/z 554.0876, found m/z 554.0872.

The enantiomeric excess was determined by HPLC on Chiralcel IC-3 column (hexane : isopropanol = 98 : 2, flowing rate = 1.0 mL/min, 30 °C, UV detection at λ = 254 nm), t_{R1} = 15.4 min (minor), t_{R2} = 17.6 min (major).

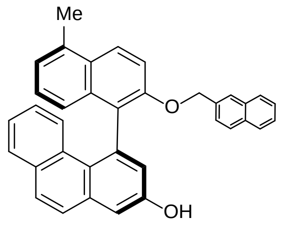

4i

White solid; isolated yield 92%, 92% ee. $[\alpha]_D^{20}$ = -125 (*c* = 2.2, $CHCl_3$); **^{1}H NMR** (400 MHz, $CDCl_3$): δ (ppm) 8.12 (d, *J* = 9.3 Hz, 1H), 7.77 (dd, *J* = 7.9, 1.5 Hz, 1H), 7.73 – 7.66 (m, 2H), 7.61 (d, *J* = 8.8 Hz, 1H), 7.57 – 7.43 (m, 4H), 7.43 – 7.36 (m, 2H), 7.30 (td, *J* = 8.1, 3.6 Hz, 2H), 7.23 – 7.06 (m, 4H), 6.95 – 6.77 (m, 3H), 5.16 (d, *J* = 12.4 Hz, 1H), 5.01 (d, *J* = 12.4 Hz, 1H), 4.95 (s, 1H), 2.77 (s, 3H); **^{13}C NMR** (101MHz, $CDCl_3$): δ (ppm) 153.2, 152.3, 136.7, 135.4, 134.6, 134.4, 133.6, 133.2, 132.9, 132.3, 131.4, 129.1, 128.6, 128.2, 128.0, 127.9, 127.7, 127.3, 126.7, 126.1, 126.0, 125.9, 125.7, 125.6, 125.3, 125.2, 125.0, 124.9, 124.3, 121.7, 115.6, 112.5, 71.3, 19.9. **IR** (neat) 422, 433, 474, 504, 518, 527, 745, 806, 857, 1178, 1217, 1228, 1365, 1452, 1598, 1739 cm^{-1}. **HRMS-ESI** exact mass calcd. for $C_{36}H_{26}O_2$ ($[M+H]^+$) requires m/z 490.1927, found m/z 490.1926.

The enantiomeric excess was determined by HPLC on Chiralcel IC-3 column (hexane : isopropanol = 97 : 3, flowing rate = 1.0 mL/min, 30 °C, UV detection at λ = 254 nm), t_{R1} = 16.1 min (minor), t_{R2} = 24.8 min (major).

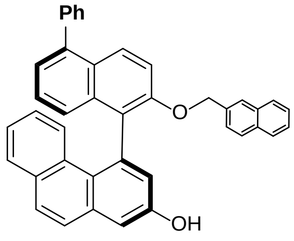

4j

Yellow solid; isolated yield 90%, 90% ee. $[\alpha]_D^{20}$ = -118 (*c* = 1.0, $CHCl_3$); **^{1}H NMR** (400 MHz, $CDCl_3$): δ (ppm) 8.02 (d, *J* = 9.3 Hz, 1H), 7.79 (dd, *J* = 7.9, 1.3 Hz, 1H), 7.76 – 7.67 (m, 2H), 7.67 – 7.42 (m, 10H), 7.38 (dd, *J* = 9.5, 5.8 Hz, 3H), 7.35 – 7.27 (m, 3H), 7.23 (t, *J* = 2.0 Hz, 1H), 7.17 (s, 1H), 7.01 – 6.89 (m, 2H), 6.85 (dd, *J* = 8.4, 1.6 Hz, 1H), 5.15 (d, *J* = 12.6 Hz, 1H), 5.01 (d, *J* = 12.6 Hz, 1H), 4.93 (s, 1H); **^{13}C NMR** (101MHz, $CDCl_3$): δ (ppm) 153.2, 152.3, 141.1, 140.4, 136.6, 135.5, 134.6, 133.8, 133.2, 132.9, 132.3, 131.4, 130.3, 128.7, 128.6, 128.4, 128.3, 128.0, 128.0, 128.0, 127.8, 127.7, 127.4, 127.3, 126.5, 126.2, 126.0, 125.9, 125.9, 125.6, 125.5, 125.4, 125.3, 124.9, 121.8, 115.8, 112.6, 71.2. **IR** (neat) 422, 434, 474, 504, 528, 702, 745, 808, 856, 1174, 1217, 1227, 1365, 1452, 1600, 1739. **HRMS-ESI** exact mass calcd. for $C_{41}H_{28}O_2$ ($[M+H]^+$) requires m/z 552.2084, found m/z 552.2082.

The enantiomeric excess was determined by HPLC on Chiralcel IC-3 column (hexane : isopropanol = 98 : 2, flowing rate = 1.0 mL/min, 30 ^oC, UV detection at λ = 254 nm), t_{R1} = 18.6 min (major), t_{R2} = 21.8 min (minor).

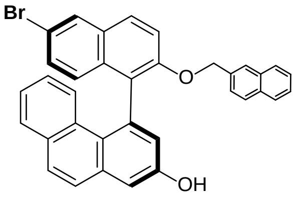

4k

White solid; isolated yield 87%, 97% ee. $[\alpha]_D^{20}$ = -53.8 (*c* = 1.0, $CHCl_3$); **^{1}H NMR** (400 MHz, $CDCl_3$): δ (ppm) 7.85 (d, *J* = 9.0 Hz, 1H), 7.78 (d, *J* = 7.8 Hz, 1H), 7.74 – 7.66 (m, 2H), 7.62 (d, *J* = 8.8 Hz, 1H), 7.56 – 7.43 (m, 4H), 7.40 (dd, *J* = 6.3, 3.2 Hz, 2H), 7.30 (d, *J* = 13.0 Hz, 3H), 7.24 – 7.12 (m, 2H), 6.94 – 6.78 (m, 3H), 5.15 (d, *J* = 12.5 Hz, 1H), 5.06 (s, 1H), 5.01 (d, *J* = 12.5 Hz, 1H); **^{13}C NMR** (101MHz, $CDCl_3$): δ (ppm) 153.2, 152.7, 135.6,

135.5, 134.3, 133.1, 132.9, 132.3, 131.9, 131.1, 130.8, 130.2, 130.0, 128.8, 128.7, 128.6, 128.4, 128.0, 127.7, 127.5, 127.3, 126.2, 126.1, 126.0, 125.9, 125.3, 124.9, 124.7, 121.6, 118.2, 117.1, 112.8, 71.3. **IR** (neat) 418, 428, 444, 473, 498, 576, 718, 744, 812, 868, 900, 1068, 1175, 1264, 1324, 1493, 1583, 1600 cm^{-1}. **HRMS-ESI** exact mass calcd. for $C_{35}H_{23}BrO_2Na$ ($[M+Na]^+$) requires m/z 577.0774, found m/z 577.0754.

The enantiomeric excess was determined by HPLC on Chiralcel OD-3 column (hexane : isopropanol = 90 : 10, flowing rate = 1.1 mL/min, 30 °C, UV detection at λ = 254 nm), t_{R1} = 7.0 min (major), t_{R2} = 9.6 min (minor).

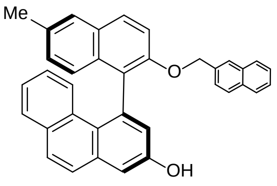

4l

White solid; isolated yield 90%, 94% ee. $[\alpha]_D^{20}$ = -88.0 (*c* = 1.4, $CHCl_3$); **^{1}H NMR** (400 MHz, $CDCl_3$): δ (ppm) 7.87 (d, *J* = 9.0 Hz, 1H), 7.78 (dd, *J* = 8.0, 1.5 Hz, 1H), 7.75 – 7.62 (m, 3H), 7.62 – 7.47 (m, 4H), 7.47 – 7.28 (m, 5H), 7.13 (s, 1H), 7.09 (dd, *J* = 8.7, 1.8 Hz, 1H), 7.05 – 6.97 (m, 1H), 6.94 – 6.87 (m, 2H), 6.85 (dd, *J* = 8.5, 1.7 Hz, 1H), 5.13 (d, *J* = 12.2 Hz, 1H), 4.99 (s, 1H), 4.95 (d, *J* = 12.2 Hz, 1H), 2.46 (s, 3H); **^{13}C NMR** (101MHz, $CDCl_3$): δ (ppm) 153.1, 151.8, 136.3, 135.4, 134.5, 133.9, 133.2, 132.9, 132.3, 131.5, 131.4, 130.2, 129.2, 128.8, 128.7, 128.6, 128.1, 128.0, 127.9, 127.6, 127.3, 127.0, 126.1, 126.0, 126.0, 125.9, 125.6, 125.6, 125.2, 125.2, 124.8, 121.7, 116.3, 112.5, 71.6, 21.6. **IR** (neat) 423, 433, 473, 503, 529, 579, 617, 674, 703, 719, 744, 817, 856, 995, 1054, 1175, 1219, 1269, 1332, 1454, 1598 cm^{-1}. **HRMS-ESI** exact mass calcd. for $C_{36}H_{26}O_2Na$ ($[M+Na]^+$) requires m/z 513.1825, found m/z 513.1814.

The enantiomeric excess was determined by HPLC on Chiralcel IC-3 column (hexane :

isopropanol = 95 : 5, flowing rate = 1.0 mL/min, 30 °C, UV detection at λ = 254 nm), t_{R1} = 9.1 min (minor), t_{R2} = 11.3 min (major).

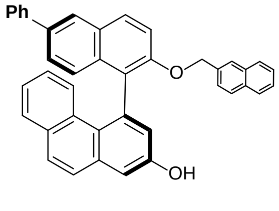

4m

Yellow solid; isolated yield 85%, 93% ee. $[\alpha]_D^{20}$ = -67.0 (*c* = 1.2, $CHCl_3$); **^{1}H NMR** (400 MHz, $CDCl_3$): δ (ppm) 8.09 (s, 1H), 8.01 (d, *J* = 9.0 Hz, 1H), 7.79 (d, *J* = 7.9 Hz, 1H), 7.75 – 7.58 (m, 6H), 7.55 – 7.29 (m, 11H), 7.22 – 7.14 (m, 2H), 6.99 – 6.90 (m, 2H), 6.86 (d, *J* = 8.4 Hz, 1H), 5.15 (d, *J* = 12.5 Hz, 1H), 5.11 (s, 1H), 5.01 (d, *J* = 12.4 Hz, 1H); **^{13}C NMR** (101MHz, $CDCl_3$): δ (ppm) 153.2, 152.6, 141.0, 136.9, 136.2, 135.5, 134.5, 133.2, 132.9, 132.5, 132.3, 131.3, 130.1, 129.8, 129.0, 128.7, 128.6, 128.2, 128.0, 128.0, 127.7, 127.3, 126.6, 126.3, 126.1, 126.0, 125.9, 125.9, 125.6, 125.3, 125.0, 124.8, 121.7, 116.5, 112.7, 71.4. **IR** (neat) 418, 426, 473, 504, 580, 696, 719, 744, 811, 856, 995, 1175, 1222, 1270, 1493, 1593. **HRMS-ESI** exact mass calcd. for $C_{41}H_{28}O_2Na$ ($[M+Na]^+$) requires m/z 575.1982, found m/z 575.1965.

The enantiomeric excess was determined by HPLC on Chiralcel IC-3 column (hexane : isopropanol = 92 : 8, flowing rate = 1.0 mL/min, 30 °C, UV detection at λ = 254 nm), t_{R1} = 6.3 min (minor), t_{R2} = 13.9 min (major).

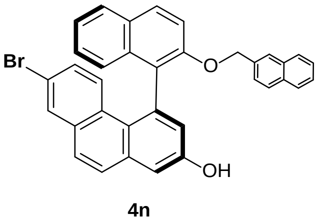

4n

White solid; isolated yield 92%, 94% ee. $[\alpha]_D^{20}$ = -90 (*c* = 0.8, $CHCl_3$); **^{1}H NMR** (400 MHz, $CDCl_3$): δ (ppm) 7.96 (d, *J* = 9.0 Hz, 1H), 7.92 – 7.84 (m, 2H), 7.76 – 7.68 (m, 2H), 7.60 (d, *J* = 8.9 Hz, 1H), 7.51 (dd, *J* = 15.7, 6.9 Hz, 2H), 7.46 (s, 1H), 7.45 – 7.31 (m, 7H), 7.21 (s, 1H), 6.96 (d, *J* = 2.8 Hz, 1H), 6.89 (td, *J* = 9.2, 2.0 Hz, 2H), 5.18 (d, *J* = 12.7 Hz,

1H), 5.07 (d, J = 12.7 Hz, 1H), 4.95 (s, 1H); **^{13}C NMR** (101MHz, $CDCl_3$): δ (ppm) 153.5, 152.4, 136.4, 135.4, 134.5, 133.8, 133.2, 133.1, 132.9, 130.6, 129.9, 129.8, 129.0, 128.5, 128.1, 128.0, 128.0, 127.9, 127.7, 127.3, 127.2, 127.1, 126.2, 126.0, 125.8, 125.4, 124.8, 124.6, 124.4, 122.0, 119.2, 116.0, 112.7, 71.2. **IR** (neat) 433, 473, 504, 716, 747, 779, 804, 861, 877, 994, 1174, 1217, 1268, 1370, 1452, 1605, 1738, 2918 cm^{-1}. **HRMS-ESI** exact mass calcd. for $C_{35}H_{23}BrO_2$ ($[M+H]^+$) requires m/z 554.0876, found m/z 554.0873.

The enantiomeric excess was determined by HPLC on Chiralcel IC-3 column (hexane : isopropanol = 97 : 3, flowing rate = 1.0 mL/min, 25 ^{o}C, UV detection at λ = 254 nm), t_{R1} = 14.3 min (minor), t_{R2} = 16.4 min (major).

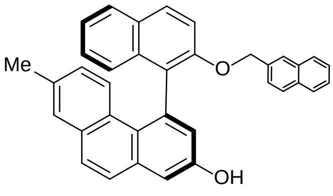

4o

White solid; isolated yield 89%, 94% ee. $[\alpha]_D^{20}$ = -131 (c = 1.1, $CHCl_3$); **^{1}H NMR** (400 MHz, $CDCl_3$): δ (ppm) 7.95 (d, J = 9.0 Hz, 1H), 7.88 (d, J = 8.1 Hz, 1H), 7.74 – 7.57 (m, 4H), 7.57 – 7.31 (m, 11H), 7.25 – 7.14 (m, 3H), 6.92 (d, J = 2.8 Hz, 1H), 6.89 (dd, J = 8.4, 1.8 Hz, 1H), 6.70 (dd, J = 8.8, 2.1 Hz, 1H), 5.16 (d, J = 12.5 Hz, 1H), 5.03 (d, J = 12.5 Hz, 1H), 4.87 (s, 1H), 2.34 (s, 3H); **^{13}C NMR** (101MHz, $CDCl_3$): δ (ppm) 152.9, 152.4, 136.0, 135.1, 134.7, 134.6, 133.4, 133.2, 132.9, 132.5, 129.9, 129.5, 129.1, 128.7, 128.3, 128.1, 128.0, 128.0, 127.9, 127.9, 127.6, 127.3, 126.9, 126.0, 126.0, 125.9, 125.9, 125.7, 125.4, 125.0, 124.9, 124.3, 121.5, 116.2, 112.6, 71.3, 21.1. **IR** (neat) 404, 429, 473, 503, 535, 718, 746, 783, 808, 856, 885, 998, 1146, 1158, 1174, 1222, 1266, 1591, 1602 cm^{-1}. **HRMS-ESI** exact mass calcd. for $C_{36}H_{26}O_2Na$ ($[M+Na]^+$) requires m/z 513.1825, found m/z 513.1816.

The enantiomeric excess was determined by HPLC on Chiralcel IA-3 column (hexane : isopropanol = 70 : 30, flowing rate = 1.0 mL/min, 25 ^{o}C, UV detection at λ = 254 nm), t_{R1} = 5.5 min (major), t_{R2} = 8.2 min (minor).

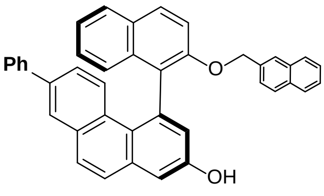

4p

Yellow solid; isolated yield 93%, 95% ee. $[\alpha]_D^{20}$ = -110 (*c* = 1.2, $CHCl_3$); **^{1}H NMR** (400 MHz, $CDCl_3$): δ (ppm) 8.04 – 7.93 (m, 2H), 7.93 – 7.84 (m, 1H), 7.84 – 7.70 (m, 2H), 7.66 (dd, *J* = 8.1, 3.2 Hz, 1H), 7.62 – 7.50 (m, 4H), 7.49 – 7.45 (m, 3H), 7.41 – 7.26 (m, 9H), 7.19 – 7.03 (m, 1H), 6.97 (q, *J* = 3.3, 2.9 Hz, 1H), 6.93 – 6.83 (m, 1H), 5.24 – 5.13 (m, 1H), 5.13 – 5.02 (m, 1H), 4.89 (s, 1H); **^{13}C NMR** (101MHz, $CDCl_3$): δ (ppm) 153.3, 152.5, 140.6, 137.5, 136.5, 135.6, 134.7, 133.4, 133.2, 132.9, 132.7, 130.5, 129.8, 129.6, 128.8, 128.6, 128.4, 128.1, 128.0, 128.0, 127.7, 127.6, 127.3, 127.2, 127.0, 126.4, 126.1, 126.0, 125.9, 125.8, 125.6, 125.2, 124.9, 124.8, 124.4, 121.7, 116.1, 112.7, 71.2. **IR** (neat) 414, 472, 501, 675, 696, 721, 756, 811, 856, 890, 996, 1052, 1173, 1220, 1265, 1328, 1459, 1600. **HRMS-ESI** exact mass calcd. for $C_{41}H_{28}O_2$ ($[M+H]^+$) requires m/z 552.2084, found m/z 552.2084.

The enantiomeric excess was determined by HPLC on Chiralcel IA-3 column (hexane : isopropanol = 75 : 25, flowing rate = 1.0 mL/min, 25 ^{o}C, UV detection at λ = 254 nm), t_{R1} = 6.1 min (major), t_{R2} = 9.1 min (minor).

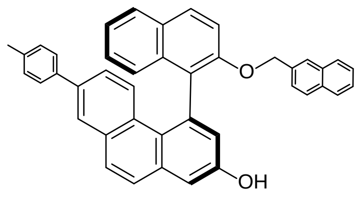

4q

White solid; isolated yield 93%, 96% ee. $[\alpha]_D^{20}$ = -111 (*c* = 1.2, $CHCl_3$); **^{1}H NMR** (400 MHz, $CDCl_3$): δ (ppm) δ 8.01 – 7.93 (m, 2H), 7.89 (d, *J* = 8.1 Hz, 1H), 7.77 (d, *J* = 8.8 Hz, 1H), 7.71 – 7.61 (m, 2H), 7.61 – 7.41 (m, 7H), 7.39 – 7.30 (m, 3H), 7.29 – 7.17 (m, 6H), 7.13 (dd, *J* = 9.0, 2.1 Hz, 1H), 6.95 (d, *J* = 2.9 Hz, 1H), 6.89 (dd, *J* = 8.4, 1.7 Hz, 1H), 5.18 (d, *J* = 12.6 Hz, 1H), 5.06 (d, *J* = 12.6 Hz, 1H), 4.90 (s, 1H), 2.37 (s, 3H); **^{13}C NMR** (101MHz, $CDCl_3$): δ (ppm) 153.2, 152.5, 137.7, 137.4, 137.0, 136.3, 135.5, 134.6, 133.4, 133.2, 132.9, 132.7, 130.3, 129.8, 129.6, 128.5, 128.5, 128.0, 128.0, 127.6, 127.0, 127.0, 126.1, 126.0, 126.0, 125.9, 125.9, 125.7, 125.1, 125.0, 124.8, 124.4, 121.7, 116.1, 112.7, 71.3, 21.2. **IR** (neat) 403, 418, 473, 502, 520, 722, 747, 785, 813, 856, 891, 996, 1052, 1147, 1174, 1221, 1265, 1601 cm^{-1}. **HRMS-ESI** exact mass calcd. for $C_{42}H_{30}O_2Na$ ($[M+Na]^+$) requires m/z 589.2138, found m/z 589.2129.

The enantiomeric excess was determined by HPLC on Chiralcel IA-3 column (hexane : isopropanol = 80 : 20, flowing rate = 1.0 mL/min, 25 ^oC, UV detection at λ = 254 nm), t_{R1} = 9.1 min (major), t_{R2} = 13.2 min (minor).

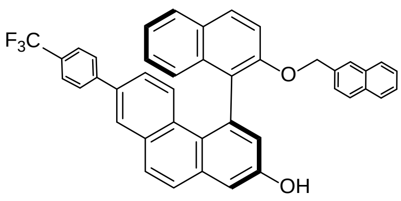

4r

Yellow solid; isolated yield 89%, 90% ee. $[\alpha]_D^{20}$ = -68 (*c* = 1.5, $CHCl_3$); **^{1}H NMR** (400 MHz, $CDCl_3$): δ (ppm) 7.98 (dd, *J* = 5.7, 3.5 Hz, 2H), 7.90 (d, *J* = 8.1 Hz, 1H), 7.78 (d, *J* = 2.7 Hz, 2H), 7.71 – 7.59 (m, 5H), 7.57 (d, *J* = 9.0 Hz, 1H), 7.51 – 7.41 (m, 4H), 7.39 – 7.27 (m, 5H), 7.23 (d, *J* = 6.0 Hz, 1H), 7.09 (dd, *J* = 9.0, 2.2 Hz, 1H), 6.98 (d, *J* = 2.8 Hz, 1H), 6.89 (dd, *J* = 8.4, 1.7 Hz, 1H), 5.19 (d, *J* = 12.7 Hz, 1H), 5.09

(d, *J* = 12.7 Hz, 1H); **^{13}C NMR** (101MHz, $CDCl_3$): δ (ppm) 153.5, 152.5, 144.1, 136.6, 135.8, 135.8, 134.6, 133.4, 133.1, 132.8, 132.6, 131.1, 129.8, 129.7, 128.4, 128.3, 128.1, 128.0, 128.0, 127.9, 127.6, 127.4, 127.1, 126.8, 126.3, 126.1, 125.9, 125.8, 125.8, 125.8, 125.7, 125.7, 125.5, 125.0, 124.9, 124.6, 124.4, 121.9, 116.1, 112.7, 71.2. **^{19}F NMR** (376 MHz, $CDCl_3$) δ -62.4. **IR** (neat) 474, 503, 610, 722, 747, 789, 814, 849, 893, 1014, 1070, 1119, 1165, 1217, 1266, 1600 cm^{-1}. **HRMS-ESI** exact mass calcd. for $C_{42}H_{27}O_2F_3Na$ ($[M+Na]^+$) requires m/z 643.1855, found m/z 643.1847.

The enantiomeric excess was determined by HPLC on Chiralcel IB-3 column (hexane : isopropanol = 90 : 10, flowing rate = 1.0 mL/min, 25 ^{o}C, UV detection at λ = 254 nm), t_{R1} = 9.4 min (major), t_{R2} = 11.3 min (minor).

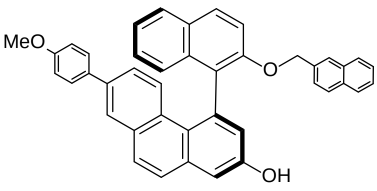

4s

White solid; isolated yield 90%, 91% ee. $[\alpha]_D^{20}$ = -57 (*c* = 2.2, $CHCl_3$); **^{1}H NMR** (400 MHz, $CDCl_3$): δ (ppm) 7.97 (s, 1H), 7.96 – 7.91 (m, 1H), 7.91 – 7.84 (m, 1H), 7.76 (d, *J* = 8.8 Hz, 1H), 7.73 – 7.62 (m, 2H), 7.52 (dd, *J* = 8.6, 5.6 Hz, 4H), 7.45 (dt, *J* = 8.8, 2.2 Hz, 3H), 7.40 – 7.26 (m, 5H), 7.23 (d, *J* = 6.6 Hz, 1H), 7.09 (dd, *J* = 9.0, 2.2 Hz, 1H), 6.99 – 6.81 (m, 4H), 5.17 (d, *J* = 12.8 Hz, 1H), 5.06 (d, *J* = 12.8 Hz, 1H), 4.94 (s, 1H), 3.83 (s, 3H); **^{13}C NMR** (101MHz, $CDCl_3$): δ (ppm) 159.2, 153.2, 152.5, 137.1, 136.3, 135.4, 134.7, 133.4, 133.1, 133.1, 132.8, 132.7, 130.0, 129.8, 129.5, 128.5, 128.5, 128.2, 128.0, 127.9, 127.6, 127.0, 126.0, 126.0, 125.9, 125.8, 125.8, 125.7, 124.9, 124.9, 124.8, 124.3, 121.6, 116.1, 114.3, 112.7, 71.2, 55.5. **IR** (neat) 417, 472, 507, 529, 650, 720, 746, 787, 812, 856, 996, 1018, 1052, 1146, 1173, 1220, 1245, 1458, 1508 . cm^{-1} **HRMS-ESI** exact mass

calcd. for $C_{42}H_{30}O_3Na$ ($[M+Na]^+$) requires m/z 605.2087, found m/z 605.2075.

The enantiomeric excess was determined by HPLC on Chiralcel IC-3 column (hexane : isopropanol = 90 : 10, flowing rate = 1.0 mL/min, 25 °C, UV detection at λ = 254 nm), t_{R1} = 4.7 min (minor), t_{R2} = 5.4 min (major).

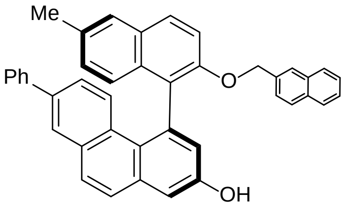

4t

White solid; isolated yield 90%, 94% ee. $[\alpha]_D^{20}$ = -27 (*c* = 1.2, $CHCl_3$); **^{1}H NMR** (400 MHz, $CDCl_3$): δ (ppm) 7.99 (d, *J* = 2.1 Hz, 1H), 7.88 (d, *J* = 9.0 Hz, 1H), 7.77 (d, *J* = 8.8 Hz, 1H), 7.72 – 7.63 (m, 3H), 7.58 (t, *J* = 8.4 Hz, 3H), 7.51 (d, *J* = 8.5 Hz, 1H), 7.49 – 7.27 (m, 9H), 7.21 (s, 1H), 7.16 – 7.09 (m, 2H), 6.94 (d, *J* = 2.8 Hz, 1H), 6.88 (dd, *J* = 8.5, 1.7 Hz, 1H), 5.15 (d, *J* = 12.6 Hz, 1H), 5.04 (d, *J* = 12.7 Hz, 1H), 4.96 (s, 1H), 2.46 (s, 3H); **^{13}C NMR** (101MHz, $CDCl_3$): δ (ppm) 153.2, 151.9, 140.6, 137.4, 136.6, 135.5, 134.7, 133.9, 133.1, 132.8, 132.7, 131.6, 130.5, 130.1, 129.3, 128.8, 128.5, 128.4, 128.0, 127.9, 127.7, 127.6, 127.3, 127.2, 127.0, 126.4, 126.1, 126.0, 125.9, 125.9, 125.5, 125.2, 125.0, 124.8, 121.7, 116.3, 112.6, 71.3, 21.6. **IR** (neat) 411, 472, 499, 520, 675, 696, 720, 757, 819, 857, 888, 995, 1054, 1172, 1219, 1268, 1332, 1459, 1599. **HRMS-ESI** exact mass calcd. for $C_{42}H_{30}O_2$ ($[M+H]^+$) requires m/z 566.2240, found m/z 566.2235.

The enantiomeric excess was determined by HPLC on Chiralcel IC-3 column (hexane : isopropanol = 75 : 25, flowing rate = 1.0 mL/min, 30 °C, UV detection at λ = 254 nm), t_{R1} = 5.5 min (minor), t_{R2} = 6.6 min (major).

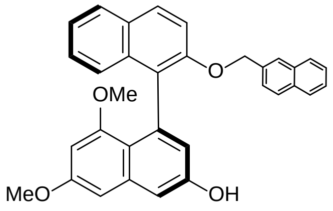

4u

White solid; isolated yield 89%, 23% ee. $[\alpha]_D^{20}$ = +6.6 (*c* = 0.5, $CHCl_3$); **^{1}H NMR** (400 MHz, $CDCl_3$): δ (ppm) 7.80 (dd, *J* = 8.6, 2.5 Hz, 2H), 7.74 (dd, *J* = 6.1, 3.4 Hz, 1H), 7.65 (d, *J* = 8.5 Hz, 1H), 7.60 (dd, *J* = 6.1, 3.4 Hz, 1H), 7.46 – 7.35 (m, 4H), 7.31 – 7.22 (m, 3H), 7.14 (dd, *J* = 6.9, 2.1 Hz, 2H), 6.73 (d, *J* = 2.3 Hz, 1H), 6.67 (d, *J* = 2.4 Hz, 1H), 6.18 (d, *J* = 2.4 Hz, 1H), 5.25 – 5.12 (m, 2H), 4.83 (s, 1H), 3.91 (s, 3H), 3.04 (s, 3H); **^{13}C NMR** (101MHz, $CDCl_3$): δ (ppm) 158.7, 158.5, 153.7, 152.0, 138.3, 135.2, 135.1, 134.0, 133.3, 132.9, 129.8, 129.1, 128.1, 127.9, 127.9, 127.7, 127.6, 126.0, 126.0, 125.9, 125.2, 123.5, 118.7, 117.0, 116.2, 109.3, 98.2, 97.1, 71.8, 55.4. **IR** (neat) 747, 813, 855, 1157, 1205, 1352, 1507, 1618, 1739, 2360. **HRMS-ESI** exact mass calcd. for $C_{36}H_{27}O_4$ ($[M+H]^+$) requires m/z 487.1904, found m/z 487.1896.

The ee was determined by HPLC on Chiralcel IC-3 column (hexane : isopropanol = 90 : 10, flowing rate = 1.0 mL/min, 30 ^{o}C, UV detection at λ = 254 nm), t_{R1} = 8.4 min (major), t_{R2} = 9.6 min (minor).

5. Transformation of 4a into 12.[4]

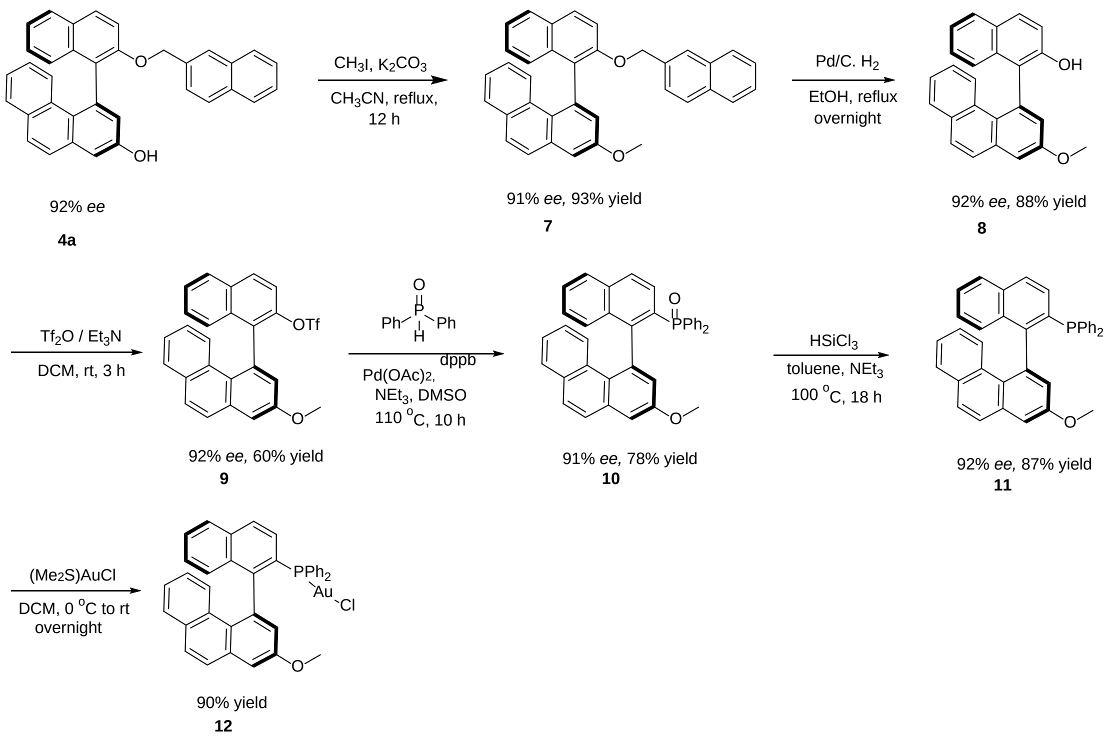

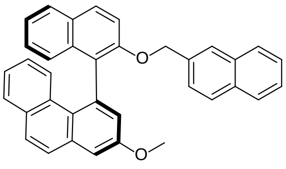

7

4a (69 mg, 0.14 mmol) and K_2CO_3 (60 mg, 0.43 mmol) were dissolved in 10 mL of acetonitrile. Then, MeI (27 uL, 0.43 mmol) was added and the reaction heated to 80°C in a sealed flask for 12 hours. After this time, the reaction mixture was cooled and 5 mL of water were added. The desired product was then extracted with ethylacetate and the organic layer was dried over anhydrous Mg_2SO_4 and evaporated under reduced pressure. Compound **7** (64 mg, 93%) was obtained as a light yellow oil. 91% *ee*. $[\alpha]_D^{20}$ = -173 (*c* = 1.2, $CHCl_3$); **^{1}H NMR** (400 MHz, $CDCl_3$): δ (ppm) 7.96 (d, *J* = 9.0 Hz, 1H), 7.93 – 7.86 (m, 1H), 7.79 (dt, *J* = 15.2, 8.8 Hz, 3H), 7.73 – 7.64 (m, 1H), 7.61 – 7.48 (m, 3H), 7.48 – 7.32 (m, 6H), 7.32 – 7.25 (m, 2H), 7.20 (s, 1H), 7.13 (d, *J* = 2.9 Hz, 1H), 6.94 – 6.82 (m, 2H), 5.16 (d, *J* = 12.7 Hz, 1H), 5.04 (d, *J* = 12.7

Hz, 1H), 3.95 (s, 3H); ^{13}**C NMR** (101 MHz, $CDCl_3$): δ (ppm) 157.3, 152.6, 136.1, 135.4, 134.8, 133.4, 133.2, 132.8, 132.3, 131.4, 129.8, 129.5, 128.7, 128.6, 128.2, 128.0, 128.0, 127.9, 127.6, 127.6, 126.9, 126.0, 126.0, 125.8, 125.8, 125.6, 125.2, 124.9, 124.8, 124.2, 121.7, 116.0, 109.7, 71.1, 55.5. **IR** (neat) 696, 719, 741, 805, 853, 1016, 1042, 1057, 1173, 1217, 1227, 1265, 1372, 1455, 1507, 1595, 1739 cm^{-1}. **HRMS-ESI** exact mass calcd. for $C_{36}H_{27}O_2$ ([M+H]+) requires m/z 491.2006, found m/z 491.1991. The enantiomeric excess was determined by HPLC on Chiralcel IA-3 column (hexane : isopropanol = 97 : 3, flowing rate = 1.0 mL/min, 25 ^{o}C, UV detection at λ = 254 nm), t_{R1} = 4.5 min (major), t_{R2} = 5.6 min (minor).

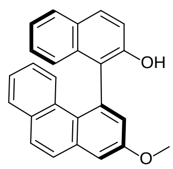

8

To a stirred solution of compound **7** (64 mg, 0.13 mmol) in EtOH (10 mL), 10% Pd/C (15 mg) was added and H_2 (1 atm) was bubbled through the reaction mixture. After stirring the reaction for 12h under H_2 atmosphere, the reaction was filtered through celite, all solvents was evaporated under reduced pressure and the remaining oil purified by column chromatography on silica gel (4% Acetone/hexanes as eluent). Compound **8** (40 mg, 88% yield) was obtained as a yellow foam. 92% *ee*. $[\alpha]_D^{20}$ = +76 (*c* = 0.8, $CHCl_3$). ^{1}**H NMR** (400 MHz, $CDCl_3$): δ (ppm) 7.95 (d, *J* = 8.9 Hz, 1H), 7.89 (dt, *J* = 8.3, 1.0 Hz, 1H), 7.78 (d, *J* = 2.9 Hz, 3H), 7.58 (d, *J* = 8.7 Hz, 1H), 7.46 (dd, *J* = 2.8, 0.8 Hz, 1H), 7.38 – 7.28 (m, 3H), 7.23 (dt, *J* = 5.9, 1.2 Hz, 2H), 7.18 (d, *J* = 2.8 Hz, 1H), 7.00 – 6.89 (m, 1H), 4.94 (s, 1H), 3.98 (d, *J* = 1.9 Hz, 3H); ^{13}**C NMR** (101 MHz, $CDCl_3$): δ (ppm) 157.8, 149.8, 136.1, 133.0, 132.9, 132.3, 130.6, 130.0, 129.5, 129.0, 128.7, 128.2, 127.3, 127.0, 126.7, 125.7, 125.1, 125.0, 124.5, 123.8, 123.5, 122.4, 118.0, 111.2, 55.6. **IR** (neat)

697, 719, 811, 854, 1030, 1057, 1136, 1158, 1171, 1199, 1216, 1230, 1256, 1340, 1381, 1454, 1595, 1738 cm^{-1}. **HRMS-ESI** exact mass calcd. for $C_{25}H_{19}O_2$ ($[M+H]^+$) requires m/z 351.1380, found m/z 351.1373. The enantiomeric excess was determined by HPLC on Chiralcel IG-K column (hexane : isopropanol = 90 : 10, flowing rate = 0.4 mL/min, 25 °C, UV detection at λ = 254 nm), t_{R1} = 4.2 min (major), t_{R2} = 5.3 min (minor).

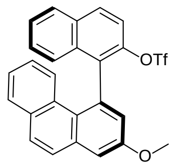

9

Trifluoromethanesulfonic anhydride (25 µL, 0.15 mmol) was added dropwise at 0 °C to a stirred dichloromethane (4.0 mL) solution of **8** (40 mg, 0.11 mmol) and pyridine (18 uL, 0.22 mmol). The mixture was allowed to warm to rt and stirred for additional 3 h. Then, a 10% aqueous HCl solution was added, and the quenched mixture was extracted by dichloromethane. The organic phases were washed with saturated $NaHCO_3$, dried over Na_2SO_4, and concentrated under a vacuum. Column chromatography on SiO_2 (eluent, 2% Acetone/hexanes) allowed the obtention of the desired triflate **9** as a yellow oil (32 mg, 60% yield). 92% *ee*. $[\alpha]_D^{20}$ = +21 (*c* = 1.0, $CHCl_3$); **^{1}H NMR** (400 MHz, $CDCl_3$): δ (ppm) 8.10 (d, *J* = 9.1 Hz, 1H), 8.01 (dd, *J* = 8.3, 1.1 Hz, 1H), 7.83 – 7.71 (m, 3H), 7.62 – 7.50 (m, 3H), 7.47 (d, *J* = 2.8 Hz, 1H), 7.38 – 7.29 (m, 2H), 7.18 – 7.15 (m, 2H), 6.91 – 6.87 (m, 1H), 3.99 (s, 3H); **^{13}C NMR** (101 MHz, $CDCl_3$): δ (ppm) 157.0, 144.0, 135.5, 134.8, 133.1, 132.9, 132.4, 131.8, 130.5, 130.3, 128.9, 128.6, 128.3, 128.1, 127.5, 127.4, 127.1, 126.1, 125.4, 125.2, 124.2, 121.8, 120.1, 119.9, 116.7, 111.2, 55.7. **^{19}F NMR** (376 MHz, $CDCl_3$) δ (ppm) -74.6. **IR** (neat) 768, 781, 855, 895, 1029, 1112, 1273, 1304, 1323, 1343, 1380, 1456, 1507, 1738 cm^{-1}. **HRMS-ESI** exact mass calcd. for $C_{26}H_{18}F_3O_4S$ ([M+H]+) requires m/z 483.0872, found m/z 483.0869. The enantiomeric excess was determined by

HPLC on Chiralcel IG-K column (hexane : isopropanol = 95 : 5, flowing rate = 0.4 mL/min, 25 °C, UV detection at λ = 254 nm), t_{R1} = 2.0 min (major), t_{R2} = 3.3 min (minor).

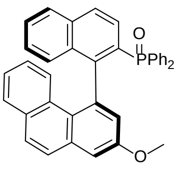

10

Triflate **9** (32 mg, 0.066 mmol, 91% ee) was dissolved in DMSO (4 mL, pre-deoxygenized by ultrasonic) and $Ph_2P(O)H$ (54 mg, 0.27 mmol), $Pd(OAc)_2$ (1.5 mg, 0.0066 mmol), and dppb (3.4 mg, 0.008 mmol) were added to the solution. Then, NEt_3 (33 µL) was added and the reaction mixture was heated to 110 °C for 10 h. After completion of the reaction, dichloromethane was added and the reaction quenched by hydrochloric acid (1 M). The resulting mixture was extracted with dichloromethane and the organic layer successively washed by saturated $NaHCO_3$ aqueous solution and saturated NaCl aqueous solution, and finally dried with anhydrous Na_2SO_4. Solvents were removed in vacuo and the remaining oil purified by flash column chromatography (eluent: hexanes/Acetone = 3:1, v/v) to afford compound **10** as a white solid (27 mg, 78% yield). 91% *ee*. $[\alpha]_D^{20}$ = -23 (*c* = 1.0, $CHCl_3$); **^{1}H NMR** (400 MHz, $CDCl_3$): δ (ppm) 8.13 – 8.00 (m, 2H), 7.94 (d, *J* = 8.2 Hz, 1H), 7.66 (dd, *J* = 7.9, 1.4 Hz, 1H), 7.56 – 7.37 (m, 5H), 7.26 – 7.20 (m, 3H), 7.18 – 7.11 (m, 4H), 7.10 (d, *J* = 2.9 Hz, 1H), 7.06 (d, *J* = 8.7 Hz, 1H), 7.04 – 6.98 (m, 3H), 6.87 – 6.75 (m, 3H), 3.82 (s, 3H); **^{13}C NMR** (101 MHz, $CDCl_3$): δ (ppm) 156.5, 147.9, 147.8, 136.6, 136.6, 135.0, 135.0, 134.8, 133.1, 132.9, 132.8, 132.7, 132.3, 132.1, 132.0, 131.9, 131.2, 131.1, 131.0, 131.0, 130.7, 130.7, 129.5, 129.4, 128.9, 128.6, 128.4, 128.1, 128.1, 128.1, 128.0, 127.9, 127.7, 127.5, 127.5, 127.4, 127.4, 127.3, 127.1, 126.2, 125.7, 124.9, 124.1, 121.2, 121.2, 112.0, 55.6. **^{31}P NMR** (162 MHz, $CDCl_3$) δ (ppm) 28.3. **IR** (neat) 719, 746, 865, 1061, 1111, 1157, 1204, 1217, 1365, 1395,

1437, 1457, 1599, 1738, 2342, 2359 cm^{-1}. **HRMS-ESI** exact mass calcd. for $C_{37}H_{28}O_2P$ ($[M+H]^+$) requires m/z 534.1743, found m/z 534.1743. The enantiomeric excess was determined by HPLC on Chiralcel IA-3 column (hexane : isopropanol = 70 : 30, flowing rate = 1.0 mL/min, 25 ^oC, UV detection at λ = 254 nm), t_{R1} = 6.0 min (major), t_{R2} = 7.3 min (minor).

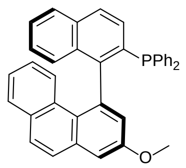

11

To a dried Schlenk flask charged with the compound **10** (27 mg, 0.05 mmol) in dry toluene (100 mL), Et_3N (35.7 mg, 49 µL, 0.35 mmol) and $HSiCl_3$ (34.5 mg, 25 µL, 0.25 mmol) were added successively under argon at 0 ^oC and the resulting mixture stirred at 100 ^oC for 18 h. After this, the reaction was diluted with Et_2O (10 mL), quenched with saturated Na_2CO_3 solution and the mixture filtered by a short Celite column. Concentration of the ethereal phase afforded an oil that was purified by flash chromatography on silica gel (hexanes/Acetone = 30:1, v/v) to afford t **11** as a white solid (23 mg, 87% yield). 92% *ee*. $[\alpha]_D^{20}$ = -78 (*c* = 0.6, $CHCl_3$); **^{1}H NMR** (400 MHz, $CDCl_3$): δ (ppm) 7.92 (t, *J* = 8.6 Hz, 2H), 7.84 – 7.70 (m, 3H), 7.47 (ddd, *J* = 8.2, 6.7, 1.3 Hz, 1H), 7.40 (td, *J* = 4.2, 2.8 Hz, 2H), 7.35 (d, *J* = 8.7 Hz, 1H), 7.30 (q, *J* = 7.4, 6.1 Hz, 5H), 7.26 – 7.10 (m, 6H), 6.98 (td, *J* = 7.7, 7.2, 1.5 Hz, 2H), 6.89 (ddd, *J* = 8.6, 6.9, 1.5 Hz, 1H), 6.73 (d, *J* = 2.8 Hz, 1H), 3.59 (s, 3H); **^{13}C NMR** (101 MHz, $CDCl_3$): δ (ppm) 156.5, 149.6, 149.3, 139.0, 138.9, 138.2, 138.1, 137.4, 137.3, 135.2, 134.1, 134.0, 133.9, 133.6, 133.5, 133.5, 133.3, 132.5, 132.4, 131.1, 130.8, 130.7, 128.7, 128.5, 128.5, 128.4, 128.3, 128.2, 128.2, 128.0, 127.6, 127.1, 127.0, 127.0, 126.8, 126.2, 126.0, 125.0, 124.1, 124.1, 120.8, 120.8, 111.4, 55.2. **^{31}P NMR** (162 MHz, $CDCl_3$) δ (ppm) -13.4. **IR** (neat) 423, 433, 487, 502, 515, 670, 694, 741,

811, 868, 1200, 1216, 1228, 1342, 1364, 1739, 2359 cm^{-1}. **HRMS-ESI** exact mass calcd. for $C_{37}H_{28}OP$ ([M+H]+) requires m/z 519.1872, found m/z 519.1864. The enantiomeric excess was determined by HPLC on Chiralcel IA-3 column (hexane : isopropanol = 95 : 5, flowing rate = 1.0 mL/min, 25 °C, UV detection at λ = 254 nm), t_{R1} = 3.1 min (major), t_{R2} = 4.1 min (minor).

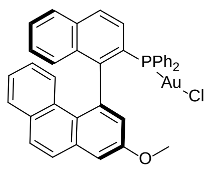

12

Compound **11** (23 mg, 0.044 mmol) and AuCl·S(Me)$_2$ (13 mg, 0.044 mmol) were stirred in dichloromethane (3 mL) overnight at rt. After this, all volatiles were removed under reduced pressure, and the residue was purified by silica gel flash column chromatography to afford gold-complex **12** (29.7 mg) in 90% yield as a white solid. Crystals of complex **12** were grown from in a saturated solution of EtOAc/pentane. **^{1}H NMR** (400 MHz, $CDCl_3$): δ (ppm) 8.05 – 7.90 (m, 2H), 7.78 (d, *J* = 8.8 Hz, 1H), 7.74 – 7.64 (m, 2H), 7.62 – 7.52 (m, 1H), 7.50 – 7.29 (m, 8H), 7.28 – 7.14 (m, 5H), 7.10 (td, *J* = 7.7, 2.6 Hz, 2H), 6.94 (d, *J* = 8.6 Hz, 1H), 6.80 – 6.67 (m, 2H), 3.80 (s, 3H); **^{13}C NMR** (101 MHz, $CDCl_3$): δ (ppm) δ 156.9, 149.9, 149.8, 136.2, 136.1, 135.9, 134.7, 134.6, 134.4, 134.3, 134.2, 134.1, 133.2, 133.1, 132.6, 131.6, 131.6, 131.3, 131.2, 130.5, 130.5, 129.9, 129.2, 129.1, 129.1, 129.0, 129.0, 128.8, 128.7, 128.6, 128.6, 128.5, 128.3, 128.1, 127.8, 127.8, 127.6, 127.6, 125.9, 125.3, 125.1, 124.4, 124.4, 123.8, 121.5, 111.8, 55.4. **^{31}P NMR** (162 MHz, $CDCl_3$) δ (ppm) 25.8. **IR** (neat) 420, 495, 520, 549, 657, 669, 691, 741, 754, 820, 1061, 1217, 1227, 1342, 1365, 1437, 1738, 2359 cm^{-1}. **HRMS-ESI** exact mass calcd. for $C_{37}H_{27}AuClOP$ ([M+Na]+) requires m/z 773.1046, found m/z 773.1027.

6. Crystal Structure of 12

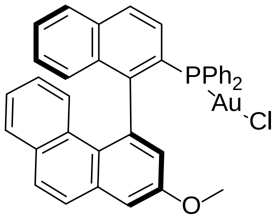

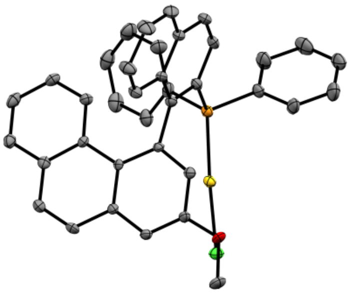

12

Basic information pertaining to crystal parameters and structure refinement is summarized in Table 1.

Table 1. Crystal data and structure refinement for **12**.

Identification code	**12**
Empirical formula	$C_{37}H_{27}AuClOP$
Formula weight	750.97
Temperature/K	100.0
Crystal system	monoclinic
Space group	$P2_1$
a/Å	10.3807(6)
b/Å	17.0354(10)
c/Å	16.6056(10)
α/°	90
β/°	90.131(2)
γ/°	90
Volume/$Å^3$	2936.5(3)
Z	4
$\rho_{calc}g/cm^3$	1.699
μ/mm^{-1}	5.185
F(000)	1472.0
Crystal size/mm^3	0.31 × 0.11 × 0.086
Radiation	MoKα (λ = 0.71073)
2Θ range for data collection/°	3.924 to 69.564
Index ranges	$-15 \le h \le 16$, $-26 \le k \le 27$, $-26 \le l \le 26$
Reflections collected	168361
Independent reflections	22935 [R_{int} = 0.0423, R_{sigma} = 0.0310]
Data/restraints/parameters	22935/4/761
Goodness-of-fit on F^2	1.200
Final R indexes [I>=2σ (I)]	R_1 = 0.0339, wR_2 = 0.0679

Final R indexes [all data]	R_1 = 0.0373, wR_2 = 0.0696
Largest diff. peak/hole / e Å^{-3}	2.50/-4.05
Flack parameter	0.0000(14)

7. References

[1] L. D. M. Nicholls, M. Marx, T. Hartung, E. G. -Fernάndez, C. Golz, M. Alcarazo, *ACS Catal.* **2018**, *8*, 6079-6085.

[2] (a) L. F. Tietze, M. A. Düfert, T. Hungerland, K. Oum, T. Lenzer, *Chem. Eur. J.* **2011**, 17, 8452-8461. (b) G. Nishida, K. Noguchi, M. Hirano, K. Tanaka, *Angew. Chem. Int. Ed*, **2007**, *46*, 3951-3954. (c) M. Satoh, Y. Shibata, Y. Kimura, K. Tanaka, *Eur. J. Org. Chem.* **2016**, 4465-4469.

[3] (a) S. S. Mahajan, M. Scian, S. Sripathy, J. Posakony, U. Lao, T. K. Loe, V. Leko, A. Thalhofer, A. D. Schuler, A. Bedalov, J. A. Simon, *J. Med. Chem.* **2014**, *57*, 3283-3294. (b) D. Li, Y. Wei, I. Marek, X. Tang, M. Shi, *Chem. Soc.*, **2015**, *6*, 5519-5525.

[4] (a) G. Cruciani, N. Milani, P. Benedetti, S. Lepri, L. Cesarini, M. Baroni, F. Spyrakis, S. Tortorella, E. Mosconi, L. Goracci, *J. Med. Chem.* **2018**, *61*, 360-371. (b) T. Hayashi, S. Hirate, K. Kitayama, H. Tsuji, A. Torii, Y. Uozumi, *Chem. Lett.* **2000**, 29, 1272-1273. (c) L. Zheng, L. Li, K. Yang, Z. Zheng, X. Xiao, L. Xu, *Tetrahedron.* **2013**, *69*, 8777-8784.

8. NMR spectra

Compound 2a

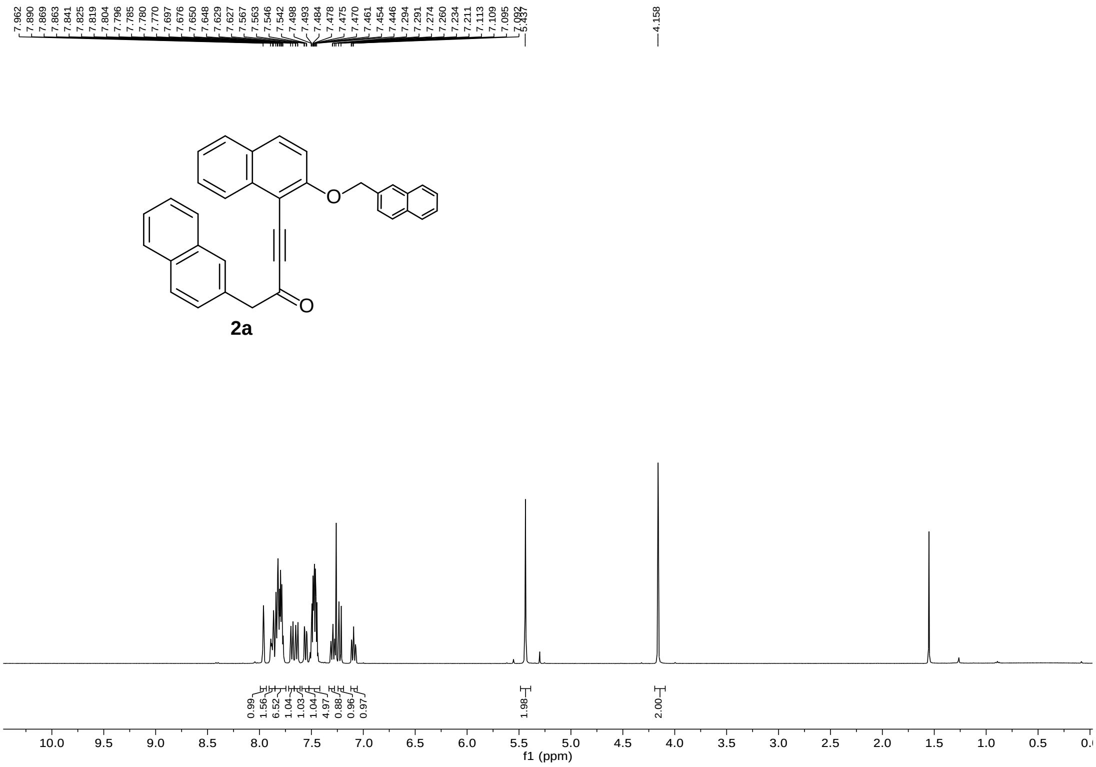

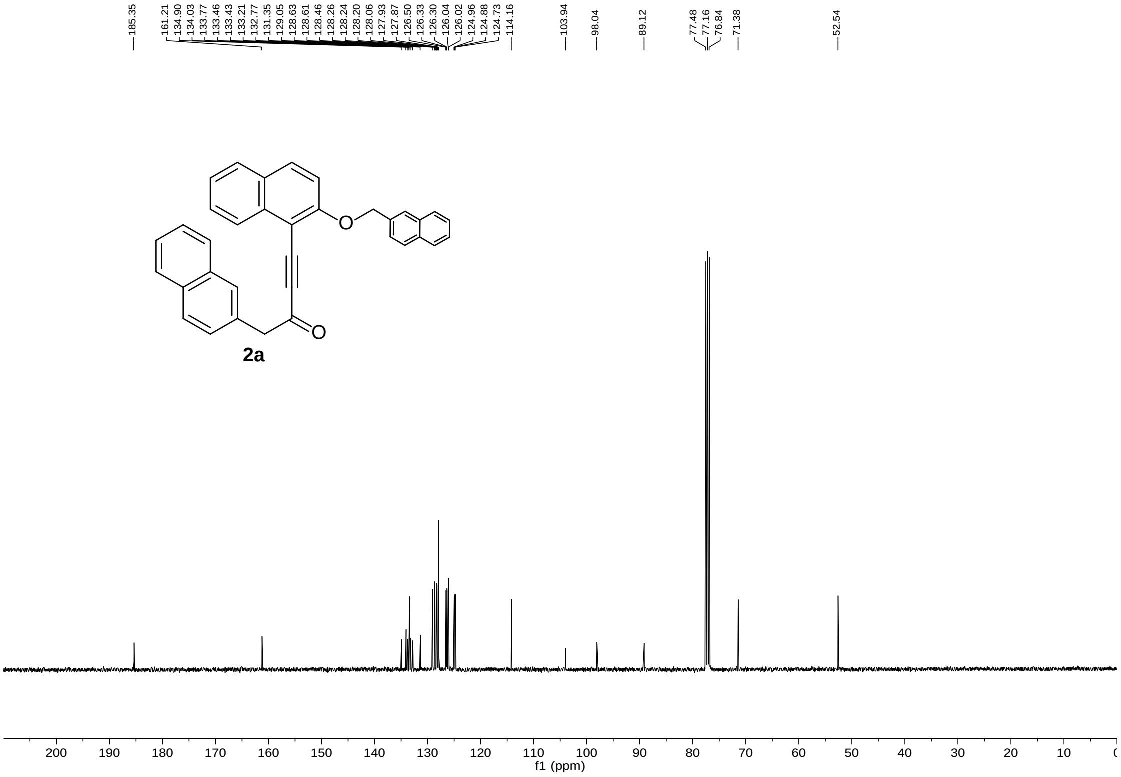

Compound 2b

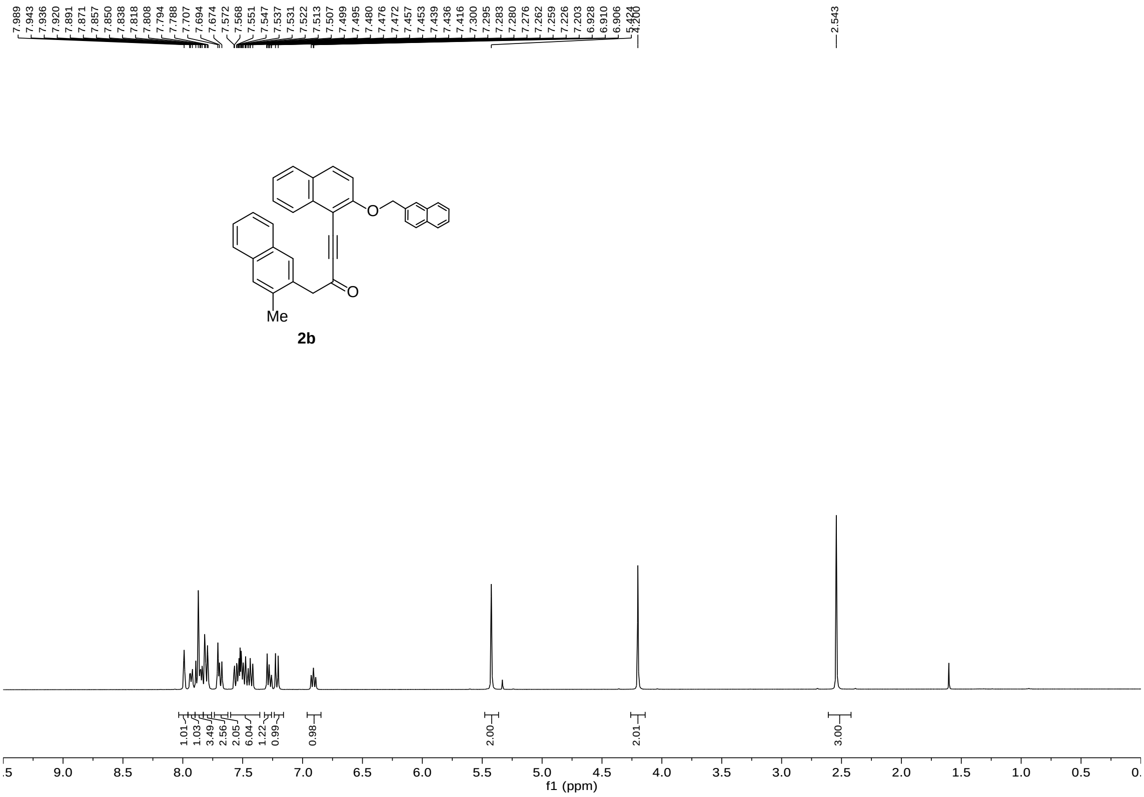

7.989 7.943 7.936 7.920 7.891 7.871 7.857 7.850 7.838 7.818 7.808 7.794 7.788 7.707 7.694 7.674 7.572 7.568 7.551 7.547 7.537 7.531 7.522 7.513 7.507 7.499 7.495 7.480 7.476 7.472 7.457 7.453 7.439 7.436 7.416 7.300 7.295 7.283 7.280 7.276 7.262 7.259 7.226 7.203 6.928 6.910 6.906 4.200
2.543
O
O
Me
2b
1.01 1.03 3.49 2.56 2.05 6.04 1.22 0.99 0.98 2.00 2.01 3.00
9.0 8.5 8.0 7.5 7.0 6.5 6.0 5.5 5.0 4.5 4.0 3.5 3.0 2.5 2.0 1.5 1.0 0.5
f1 (ppm)

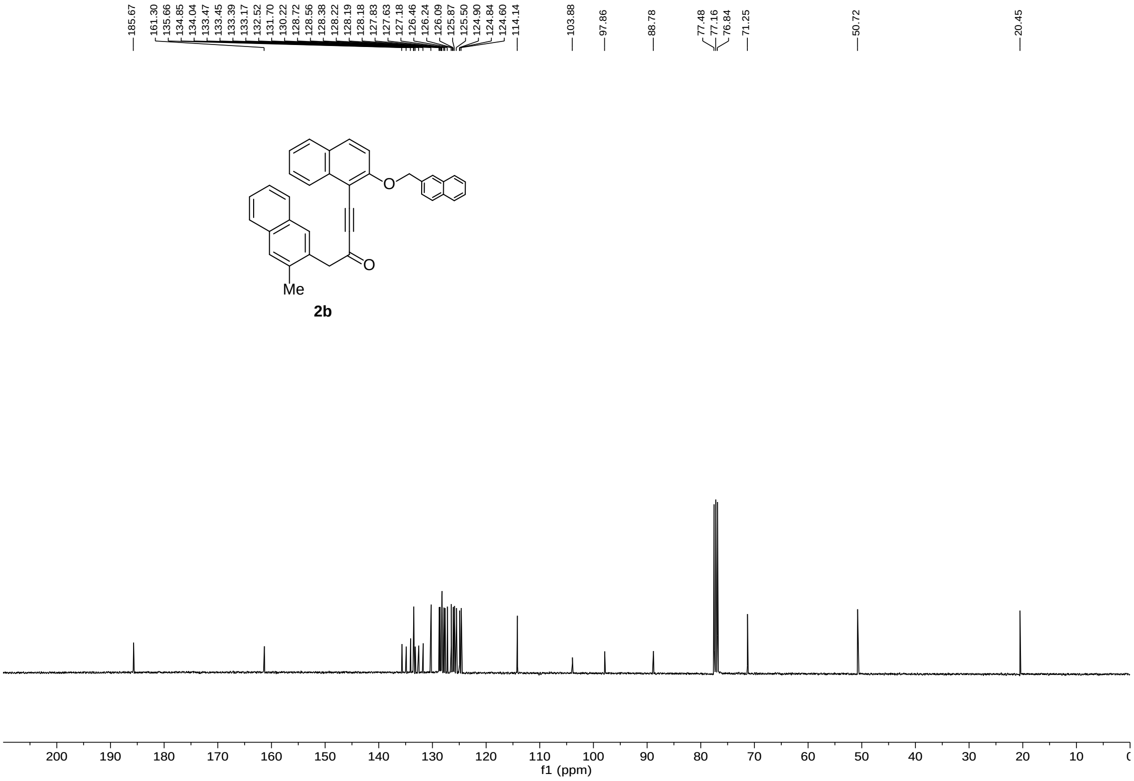

185.67 161.30 135.66 134.85 134.04 133.47 133.45 133.39 133.17 131.70 130.22 128.72 128.56 128.38 128.22 128.19 128.18 127.83 127.63 127.18 126.46 126.24 126.09 125.87 125.50 124.90 124.84 124.60 114.14
103.88
97.86
88.78
77.48 77.16 76.84
71.25
50.72
20.45
O
O
Me
2b
200 190 180 170 160 150 140 130 120 110 100 90 80 70 60 50 40 30 20 10
f1 (ppm)

Compound 2c

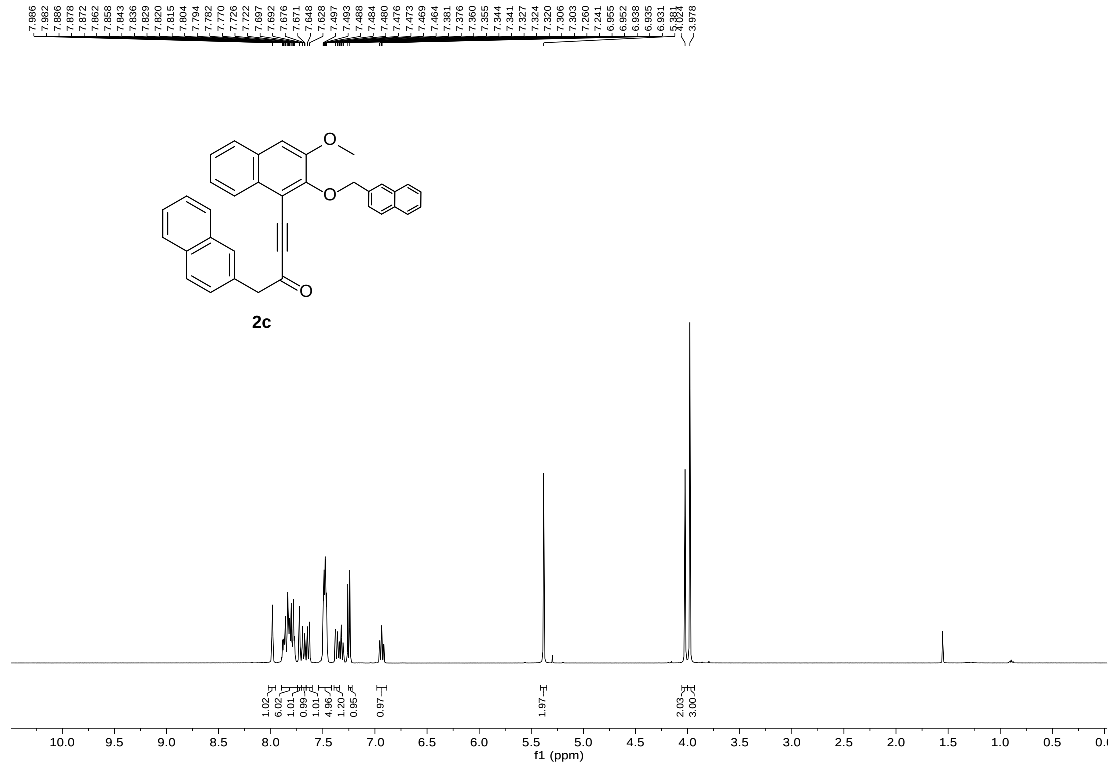
7.986
7.982
7.886
7.878
7.872
7.862
7.858
7.843
7.836
7.829
7.820
7.815
7.804
7.794
7.782
7.770
7.726
7.722
7.697
7.692
7.676
7.671
7.648
7.628
7.497
7.493
7.488
7.484
7.480
7.476
7.473
7.469
7.464
7.381
7.376
7.360
7.355
7.344
7.341
7.327
7.324
7.320
7.306
7.303
7.260
7.241
6.955
6.952
6.938
6.935
6.931
5.381
4.024
3.978
2c
1.02
6.02
1.01
0.99
1.01
4.96
1.20
0.95
0.97
1.97
2.03
3.00
f1 (ppm)

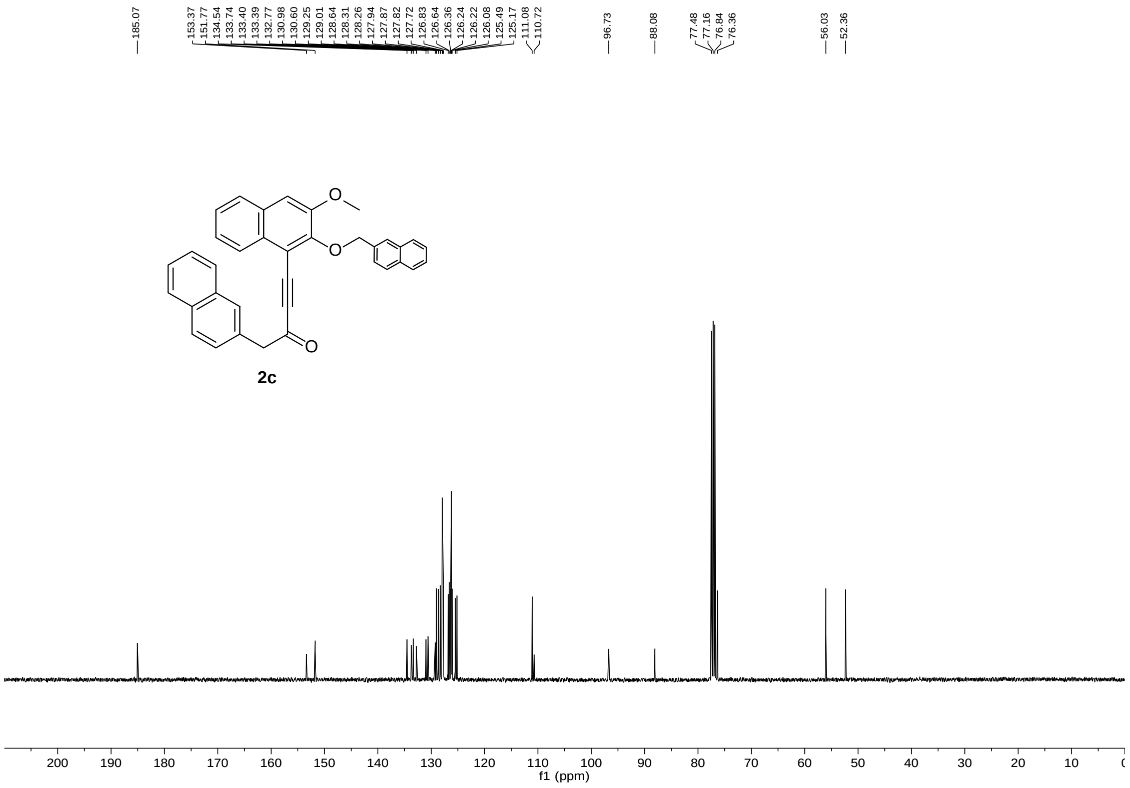
185.07
153.37
151.77
134.54
133.74
133.40
133.39
132.77
130.98
130.60
129.25
129.01
128.64
128.31
128.26
127.94
127.87
127.82
127.72
126.83
126.64
126.36
126.24
126.22
126.08
125.49
125.17
111.08
110.72
96.73
88.08
77.48
77.16
76.84
76.36
56.03
52.36
2c
f1 (ppm)

Compound 2d

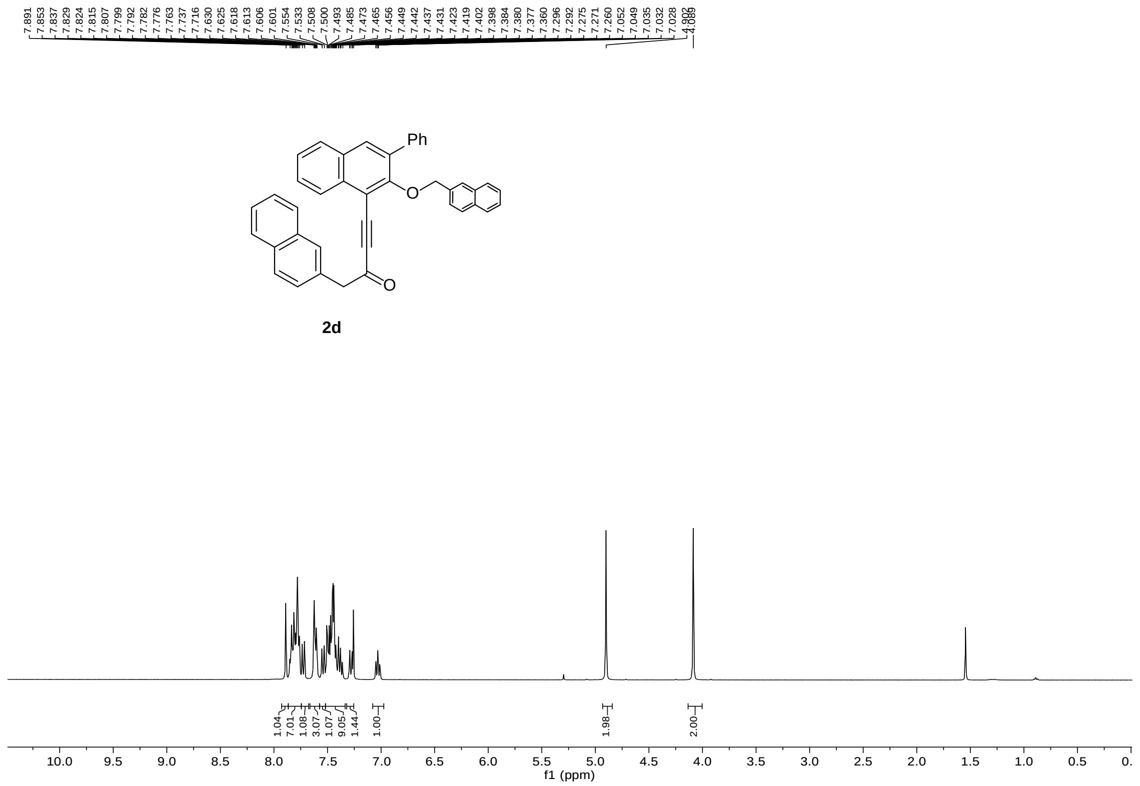

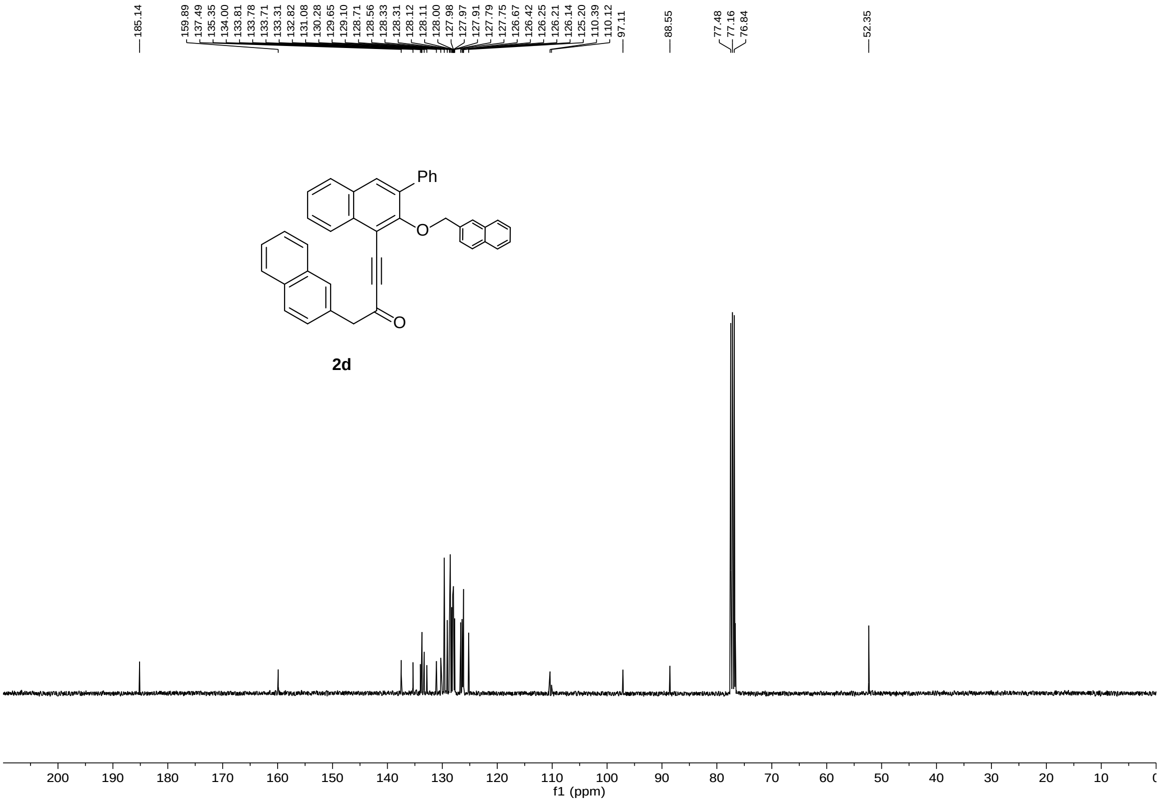

Compound 2e

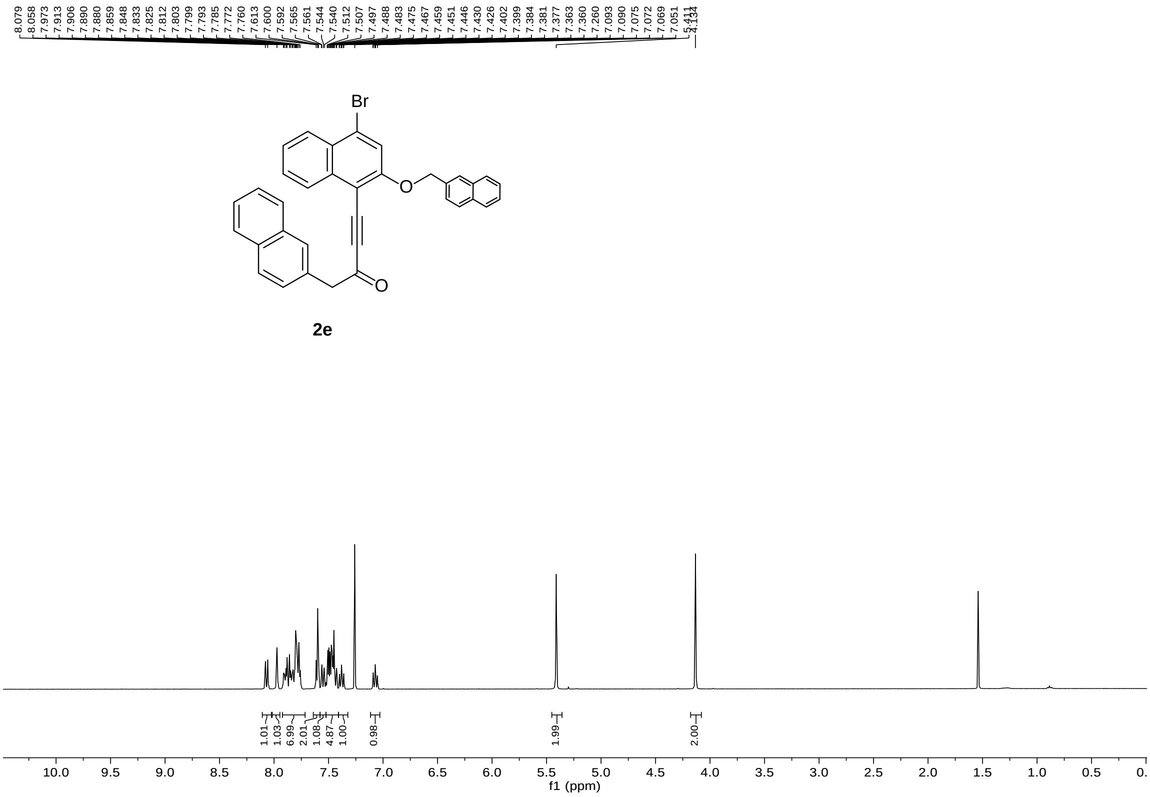
8.079 8.058 7.973 7.913 7.906 7.890 7.880 7.859 7.848 7.833 7.825 7.812 7.803 7.799 7.793 7.785 7.772 7.760 7.613 7.600 7.592 7.565 7.561 7.544 7.540 7.512 7.507 7.497 7.488 7.483 7.475 7.467 7.459 7.451 7.446 7.430 7.426 7.402 7.399 7.384 7.381 7.377 7.363 7.360 7.260 7.093 7.090 7.075 7.072 7.069 7.051 5.411 4.134
Br
O
O
2e
1.01 1.03 6.99 2.01 1.08 4.87 1.00 0.98 1.99 2.00
10.0 9.5 9.0 8.5 8.0 7.5 7.0 6.5 6.0 5.5 5.0 4.5 4.0 3.5 3.0 2.5 2.0 1.5 1.0 0.5 0.
f1 (ppm)

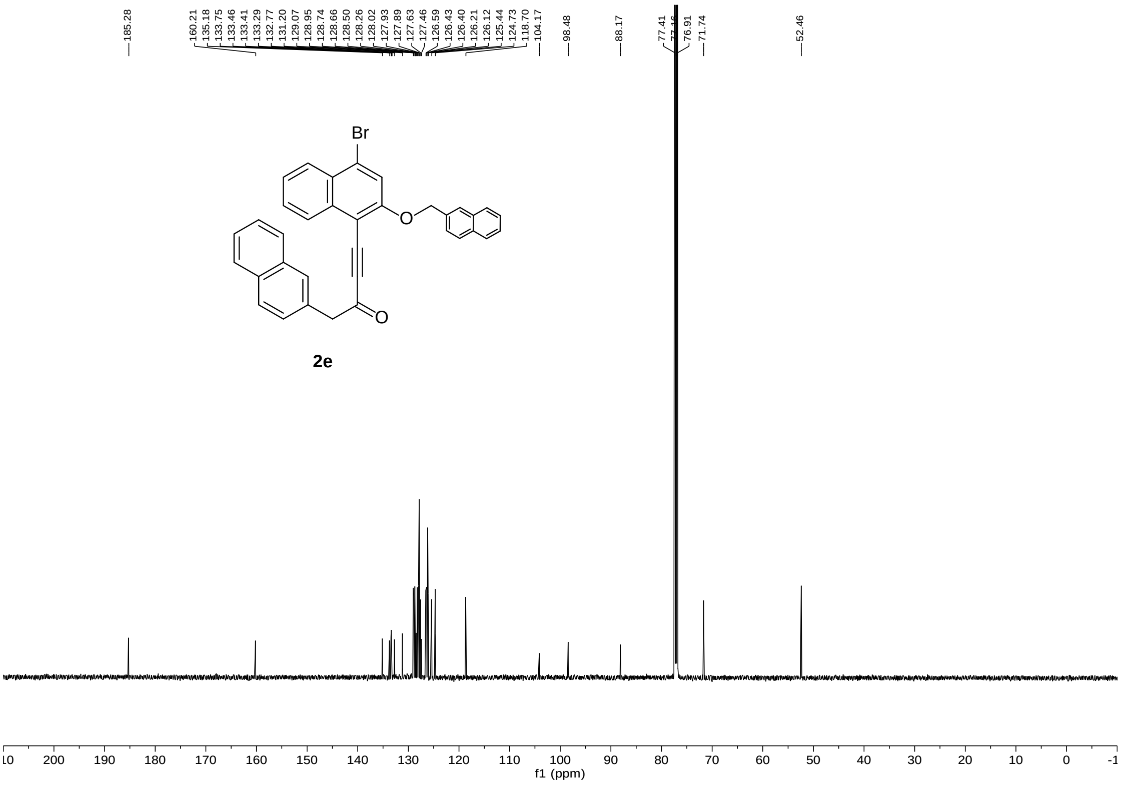
185.28
160.21 135.18 133.75 133.46 133.41 133.29 132.77 131.20 129.07 128.95 128.74 128.66 128.50 128.26 128.02 127.93 127.89 127.63 127.46 126.59 126.43 126.40 126.21 126.12 125.44 124.73 118.70 104.17
98.48
88.17
77.41 76.91 71.74
52.46
Br
O
O
2e
200 190 180 170 160 150 140 130 120 110 100 90 80 70 60 50 40 30 20 10 0 -1
f1 (ppm)

Compound 2f

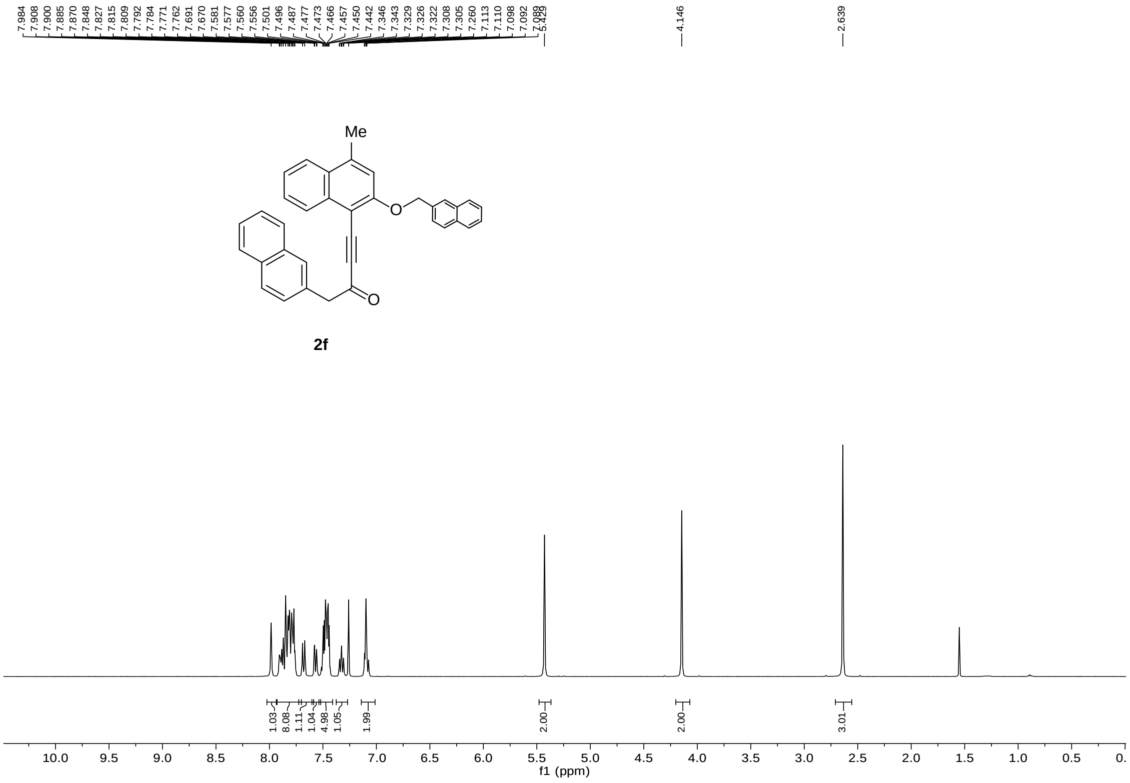

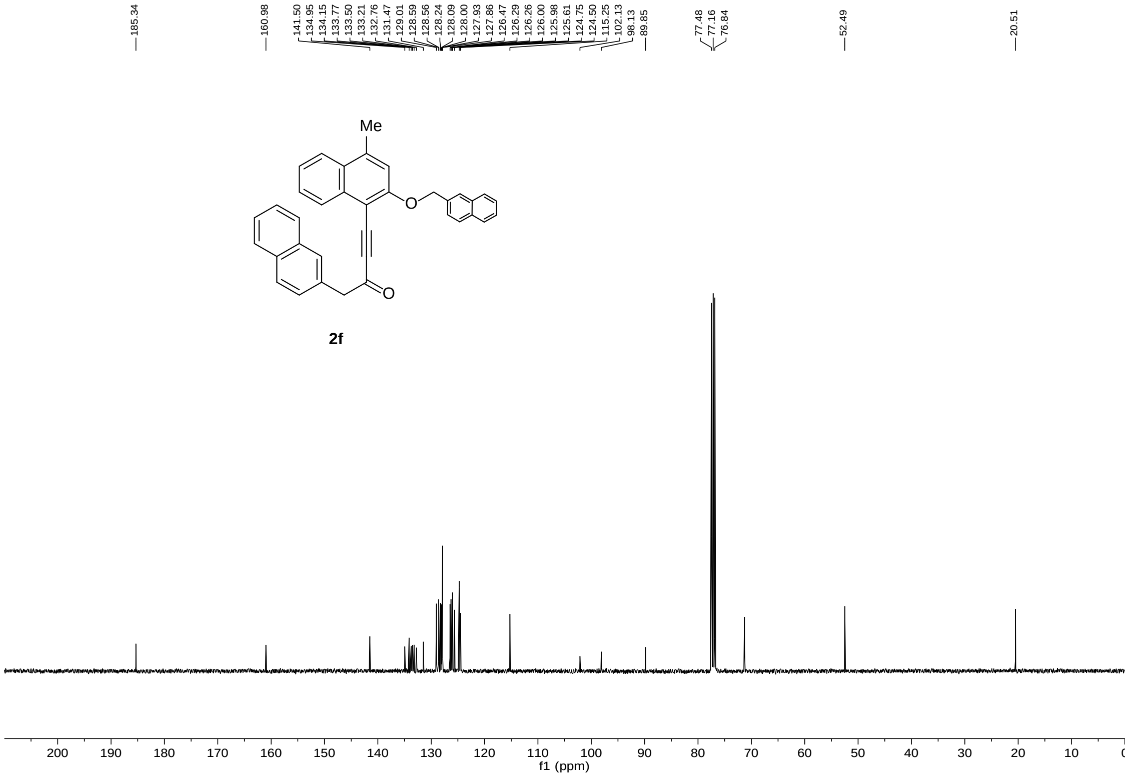

Compound 2g

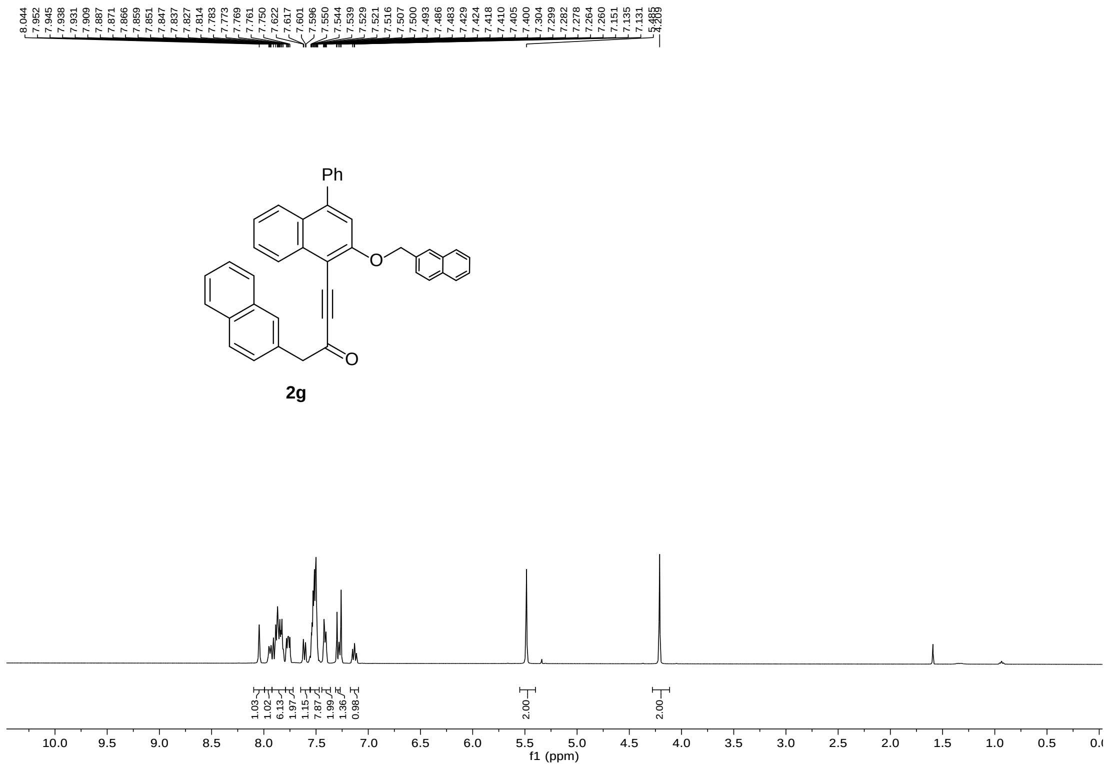
Ph
O
2g
1.03
1.02
6.13
1.97
1.15
7.87
1.99
1.36
0.98
2.00
2.00
f1 (ppm)

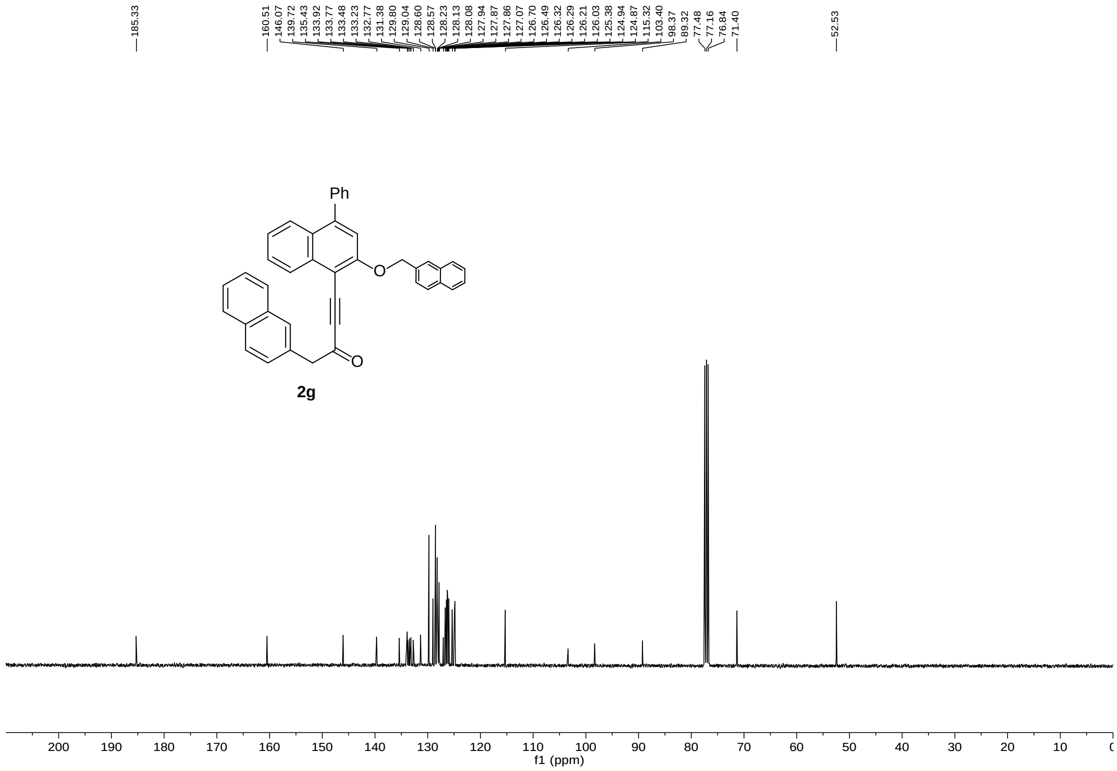
185.33
160.51
146.07
139.72
135.43
133.92
133.77
133.48
133.23
132.77
131.38
129.80
129.04
128.60
128.57
128.23
128.13
128.08
127.94
127.87
127.86
127.07
126.70
126.49
126.32
126.29
126.21
126.03
125.38
124.94
124.87
115.32
103.40
98.37
89.32
77.48
77.16
76.84
71.40
52.53
Ph
O
2g
f1 (ppm)

Compound 2h

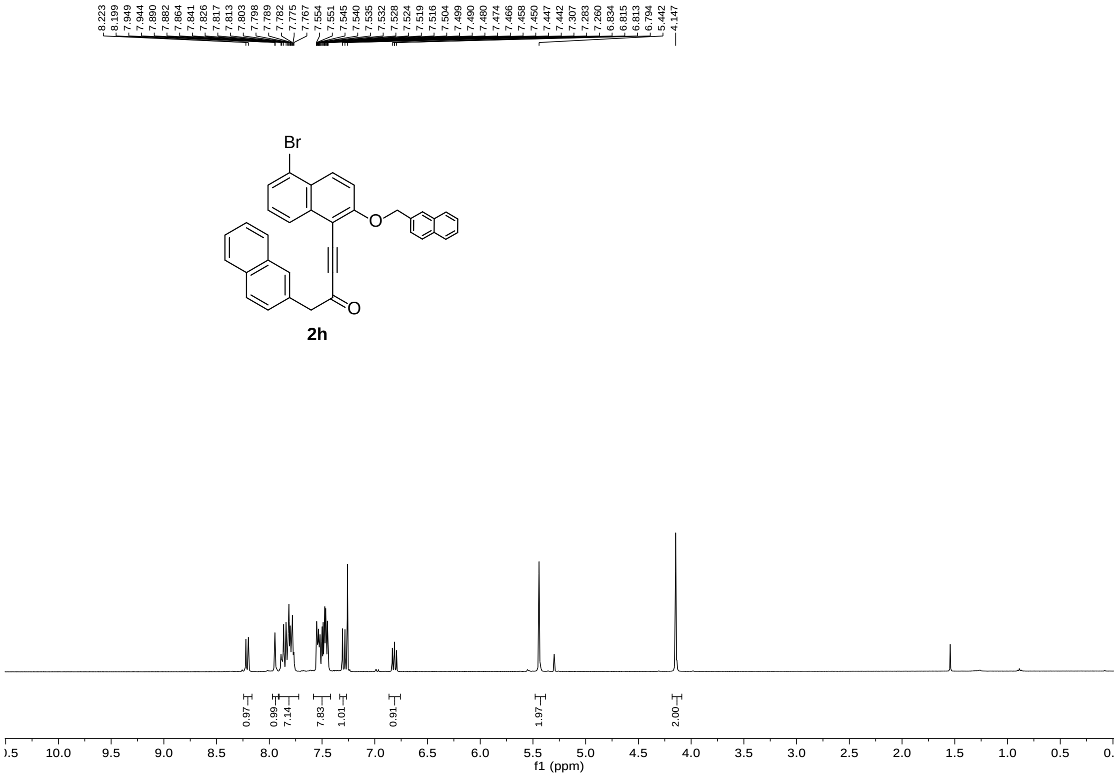
8.223
8.199
7.949
7.944
7.890
7.882
7.864
7.841
7.826
7.817
7.813
7.803
7.798
7.789
7.782
7.775
7.767
7.554
7.551
7.545
7.540
7.535
7.532
7.528
7.524
7.519
7.516
7.504
7.499
7.490
7.480
7.474
7.466
7.458
7.450
7.447
7.442
7.307
7.283
7.260
6.834
6.815
6.813
6.794
5.442
4.147
Br
O
O
2h
0.97
0.99
7.14
7.83
1.01
0.91
1.97
2.00
f1 (ppm)

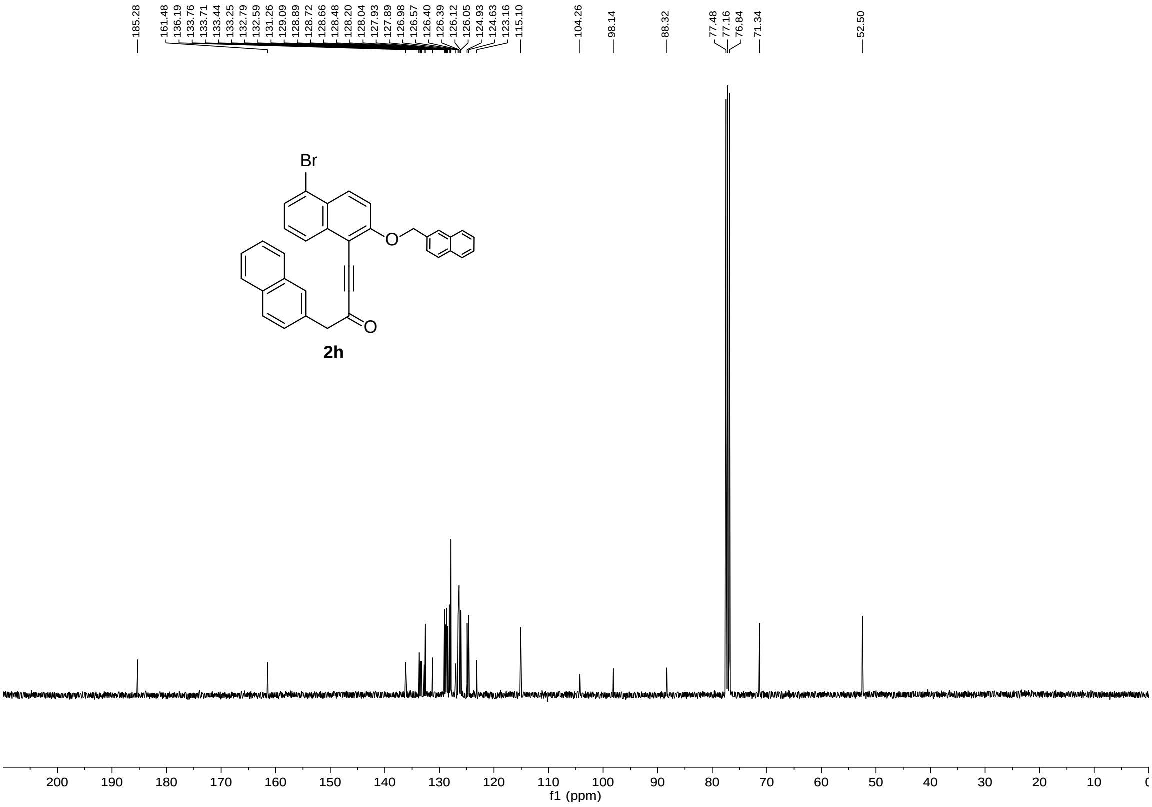
185.28
161.48
136.19
133.76
133.71
133.44
133.25
132.79
132.59
131.26
129.09
128.89
128.72
128.66
128.48
128.20
128.04
127.93
127.89
126.98
126.57
126.40
126.39
126.12
126.05
124.93
124.63
123.16
115.10
104.26
98.14
88.32
77.48
77.16
76.84
71.34
52.50
Br
O
O
2h
f1 (ppm)

Compound 2i

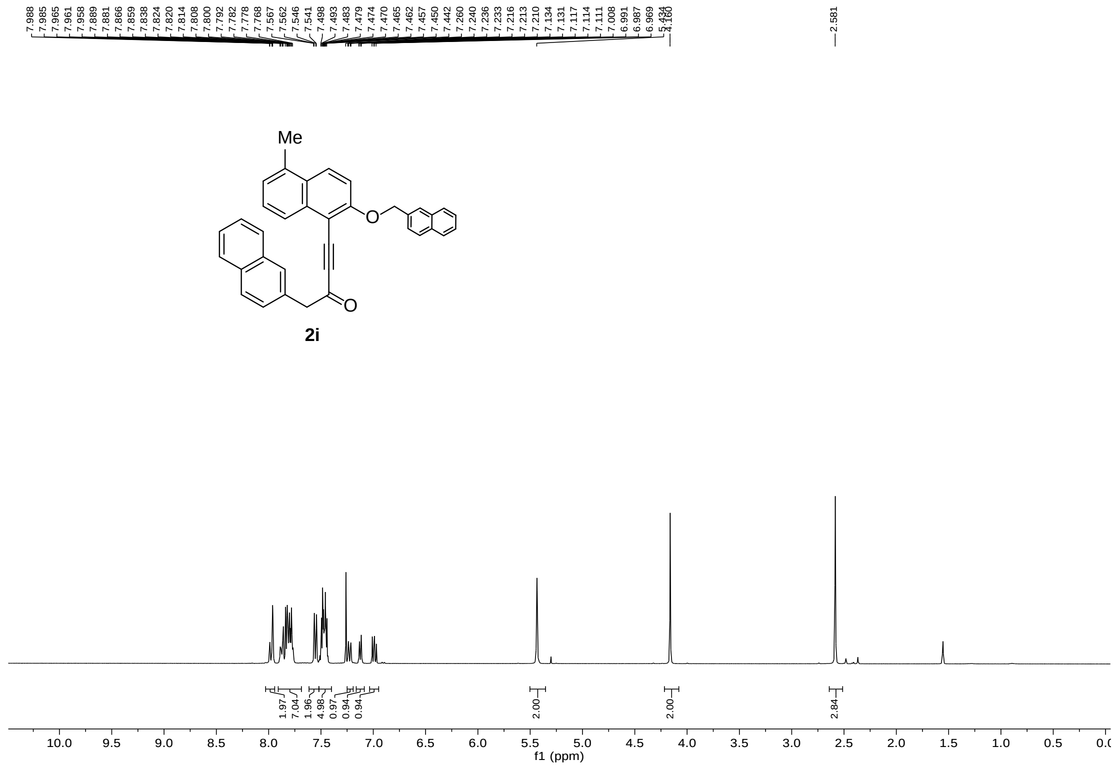

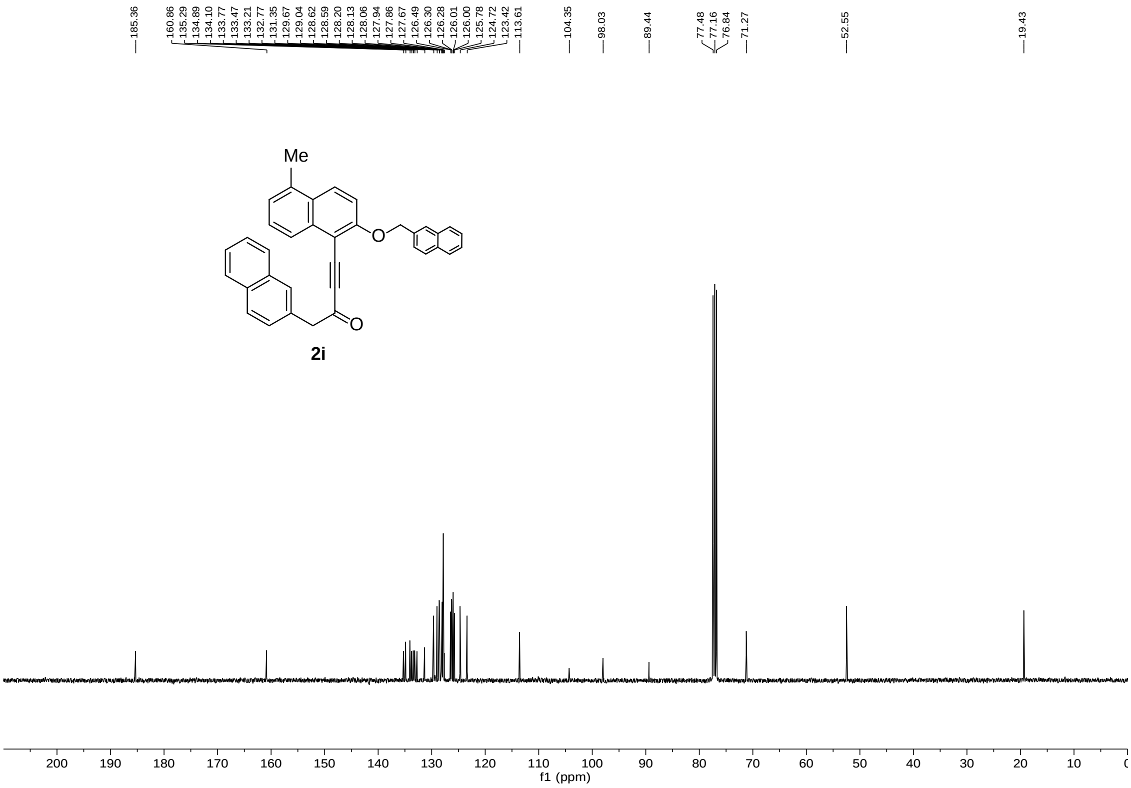

Compound 2j

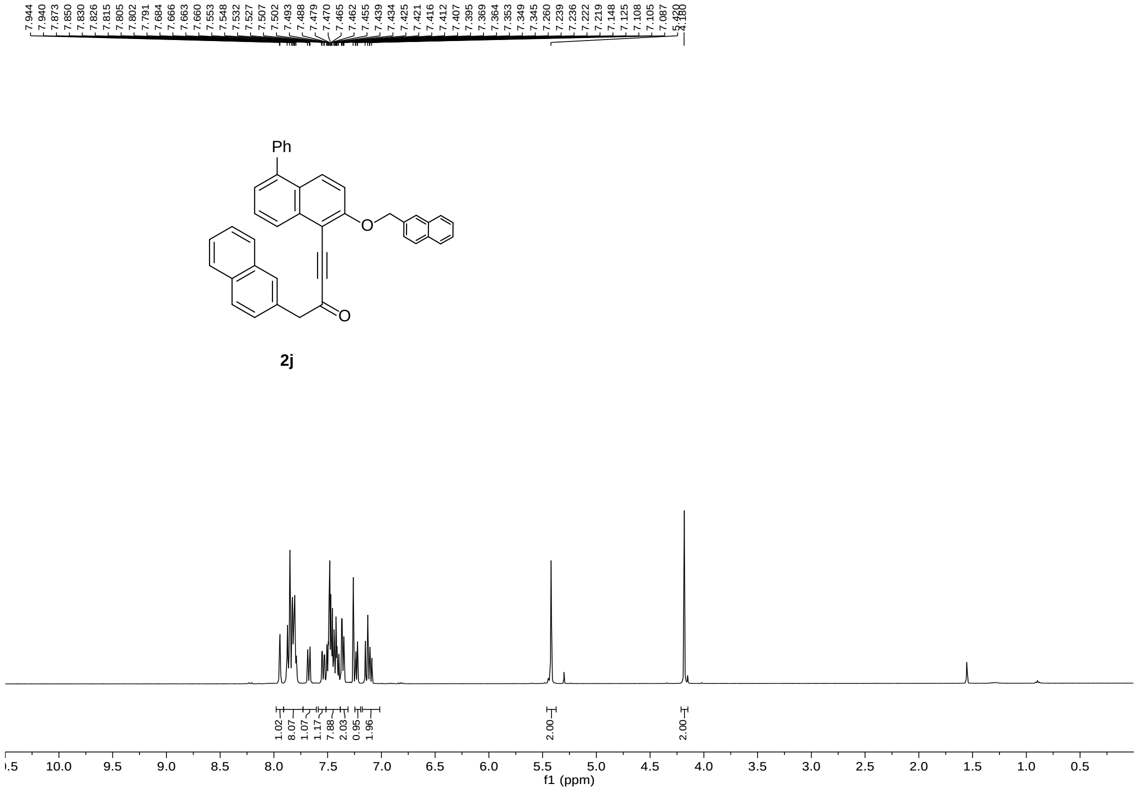

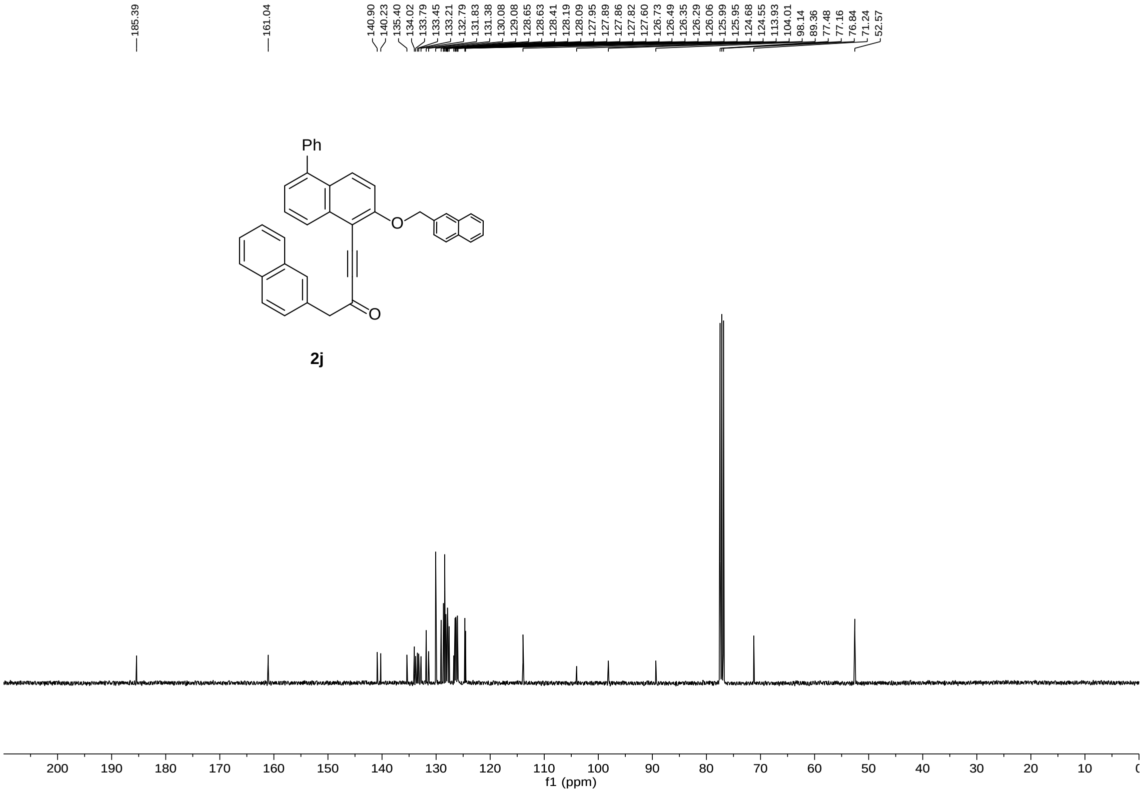

Compound 2k

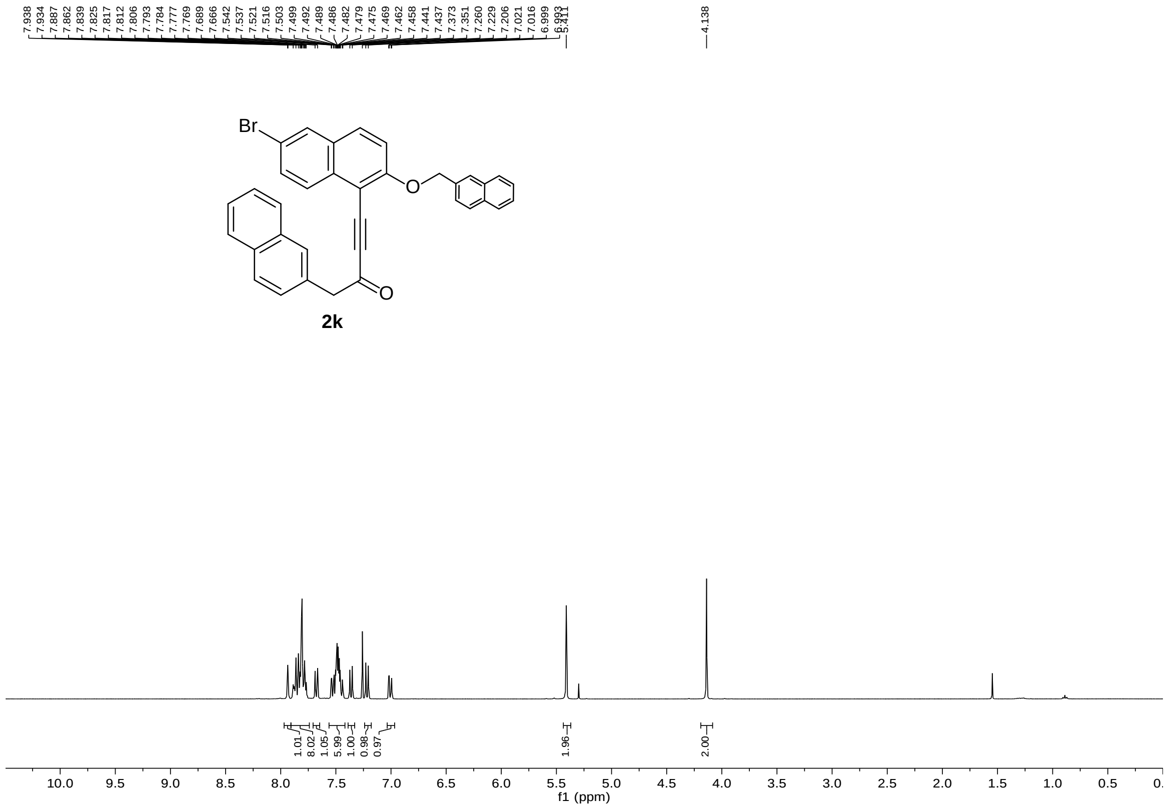

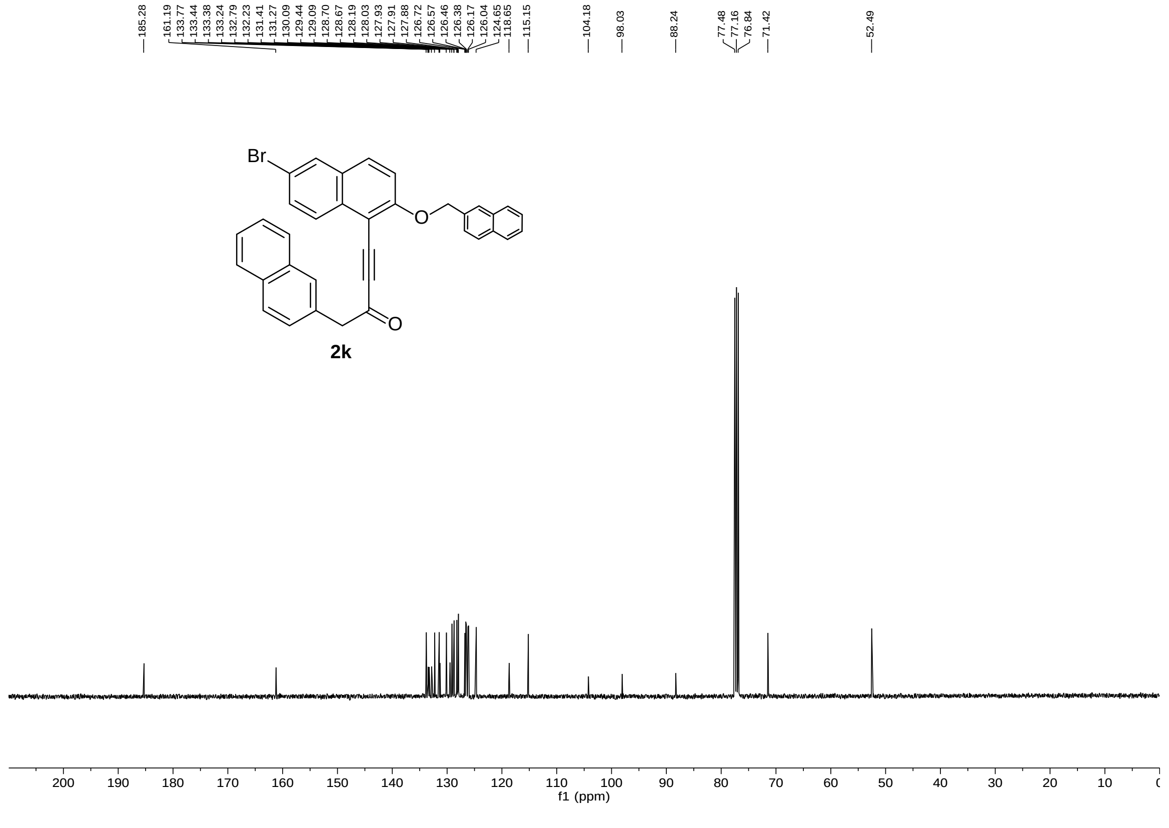

Compound 2l

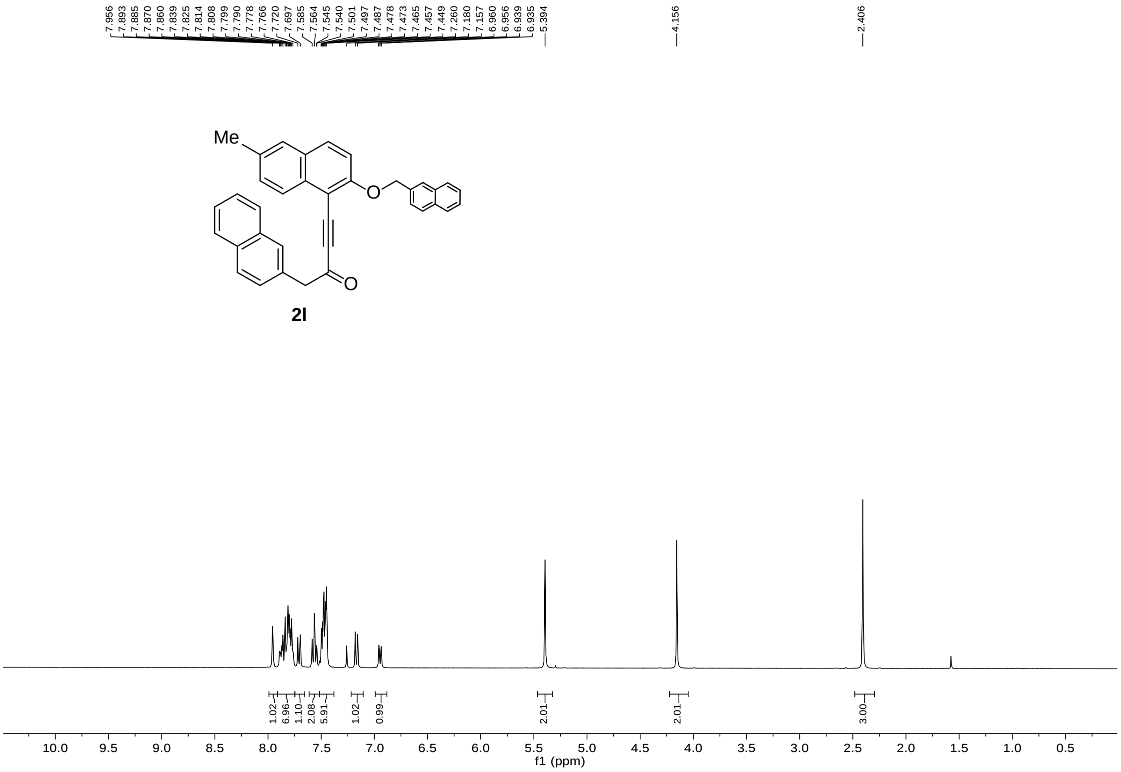
7.956 7.893 7.885 7.870 7.860 7.839 7.825 7.814 7.808 7.799 7.790 7.778 7.766 7.720 7.697 7.585 7.564 7.545 7.540 7.501 7.497 7.487 7.478 7.473 7.465 7.457 7.449 7.260 7.180 7.157 6.960 6.956 6.939 6.935 5.394
4.156
2.406
Me
O
O
2l
1.02 6.96 1.10 2.08 5.91 1.02 0.99 2.01 2.01 3.00
10.0 9.5 9.0 8.5 8.0 7.5 7.0 6.5 6.0 5.5 5.0 4.5 4.0 3.5 3.0 2.5 2.0 1.5 1.0 0.5
f1 (ppm)

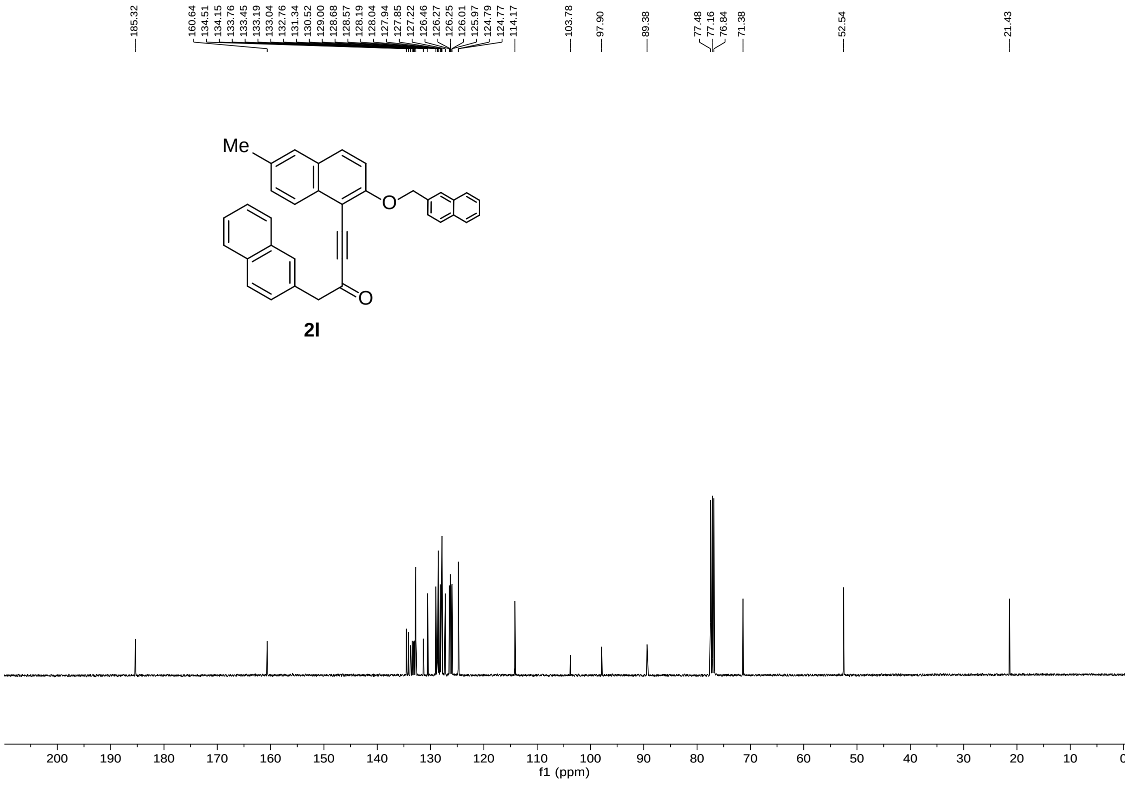
185.32
160.64 134.51 134.15 133.76 133.45 133.19 133.04 132.76 131.34 130.52 129.00 128.68 128.57 128.19 128.04 127.94 127.85 127.22 126.46 126.27 126.25 126.01 125.97 124.79 124.77 114.17
103.78
97.90
89.38
77.48 77.16 76.84
71.38
52.54
21.43
Me
O
O
2l
200 190 180 170 160 150 140 130 120 110 100 90 80 70 60 50 40 30 20 10 0
f1 (ppm)

Compound 2m

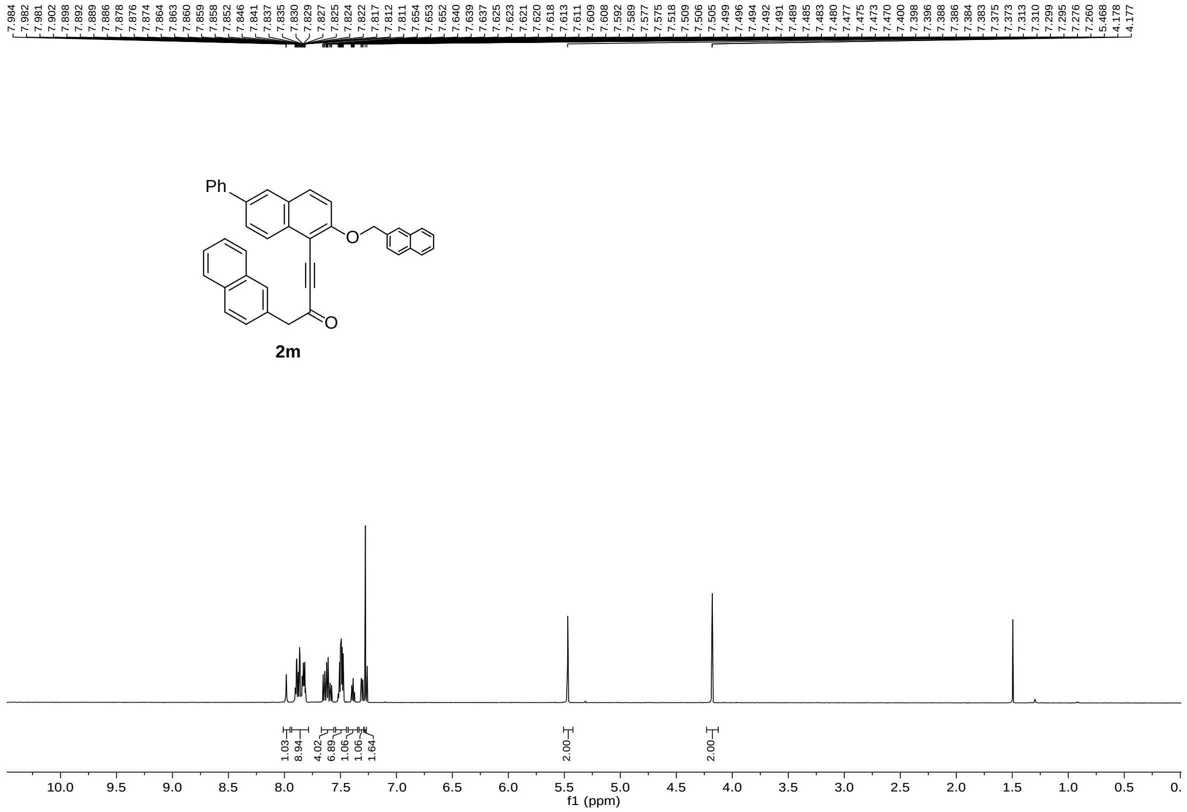

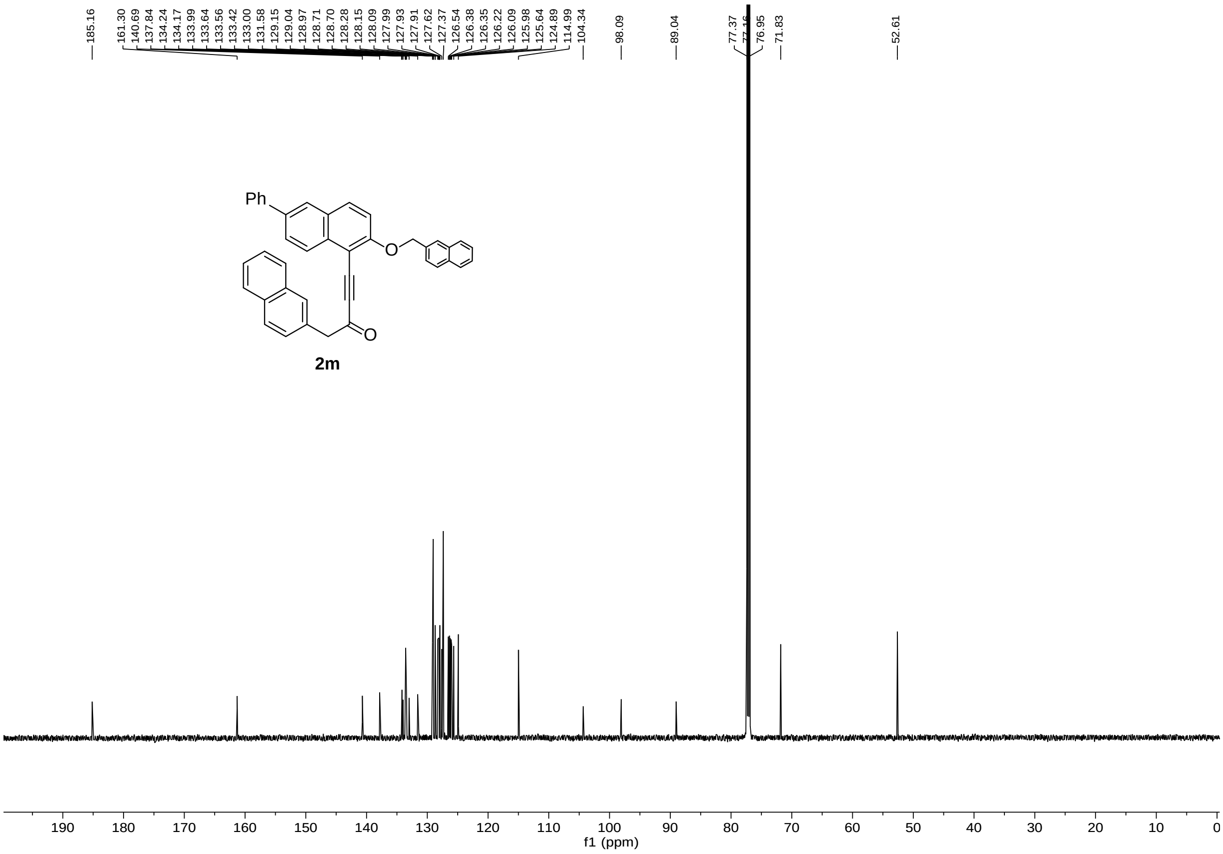

Compound 2n

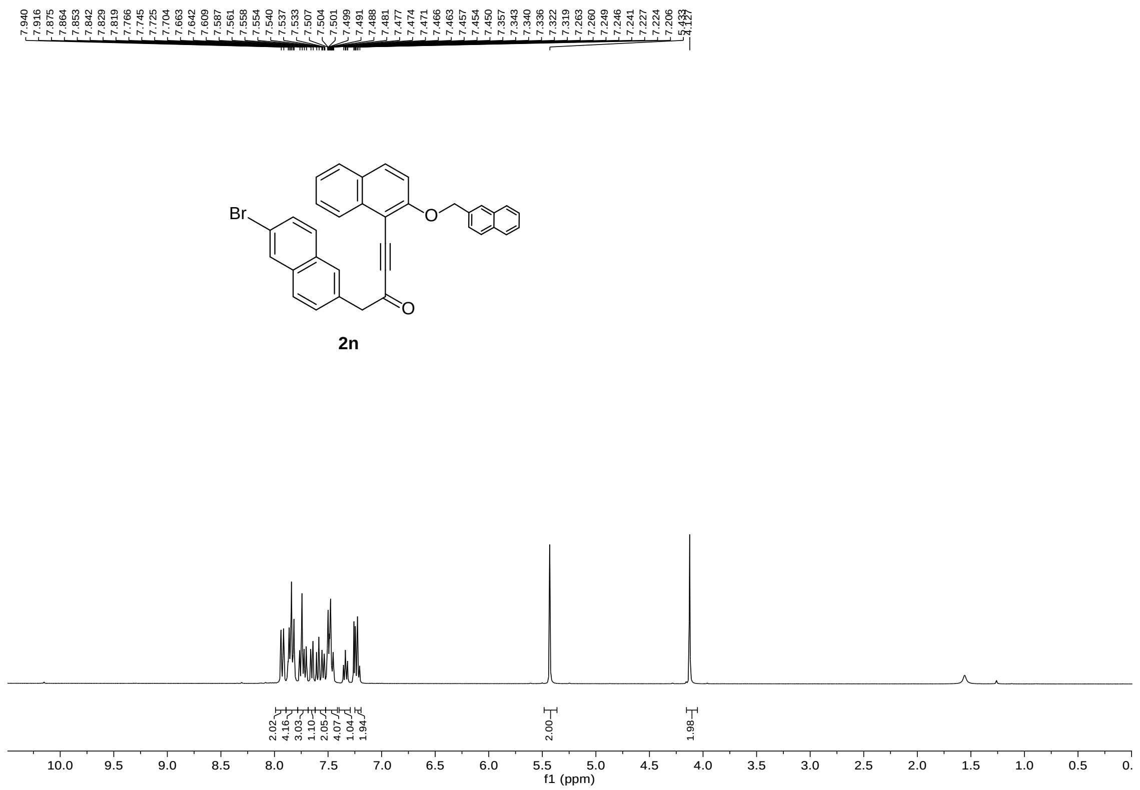

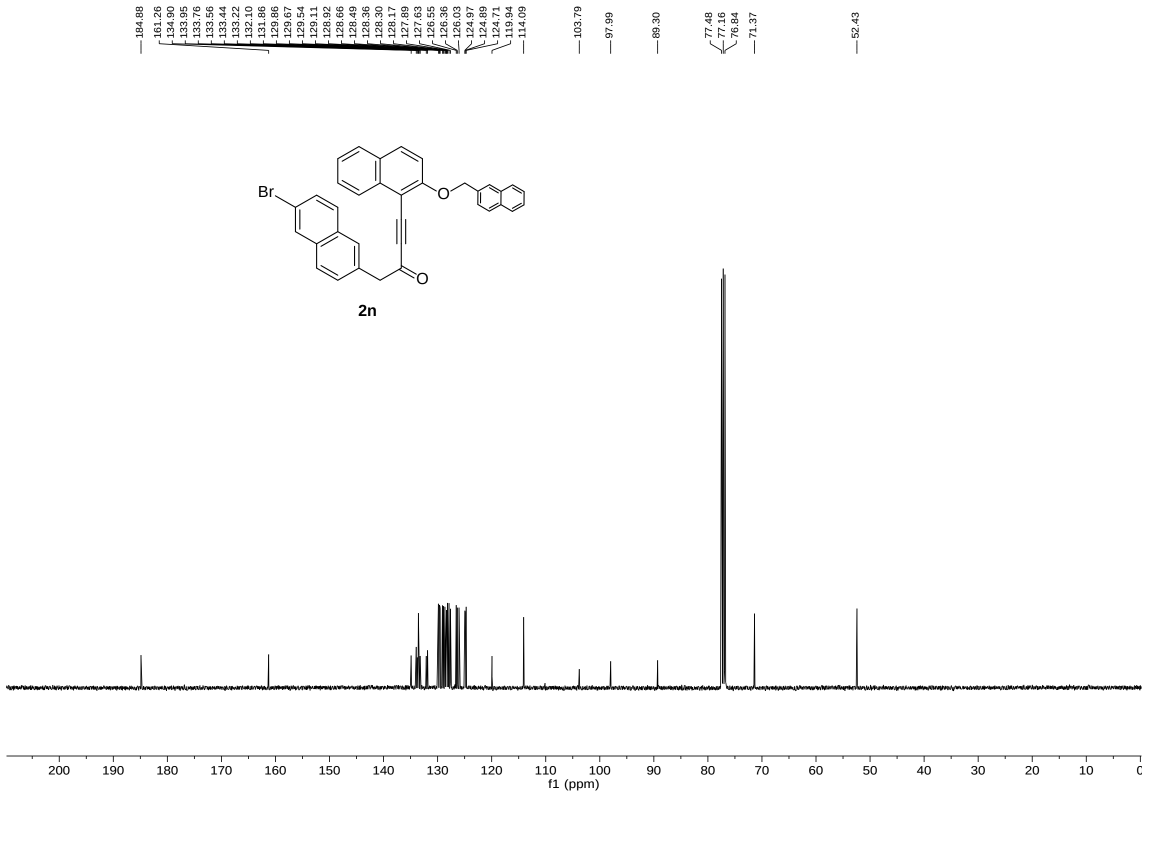

Compound 2o

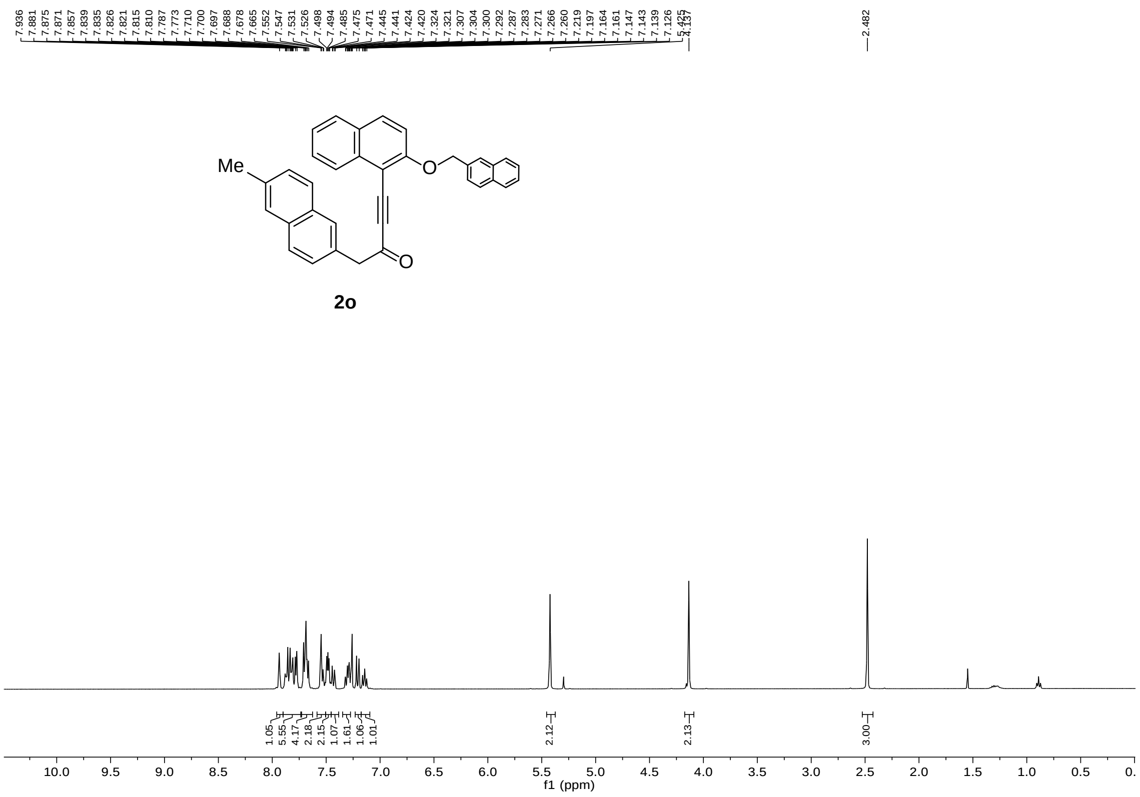

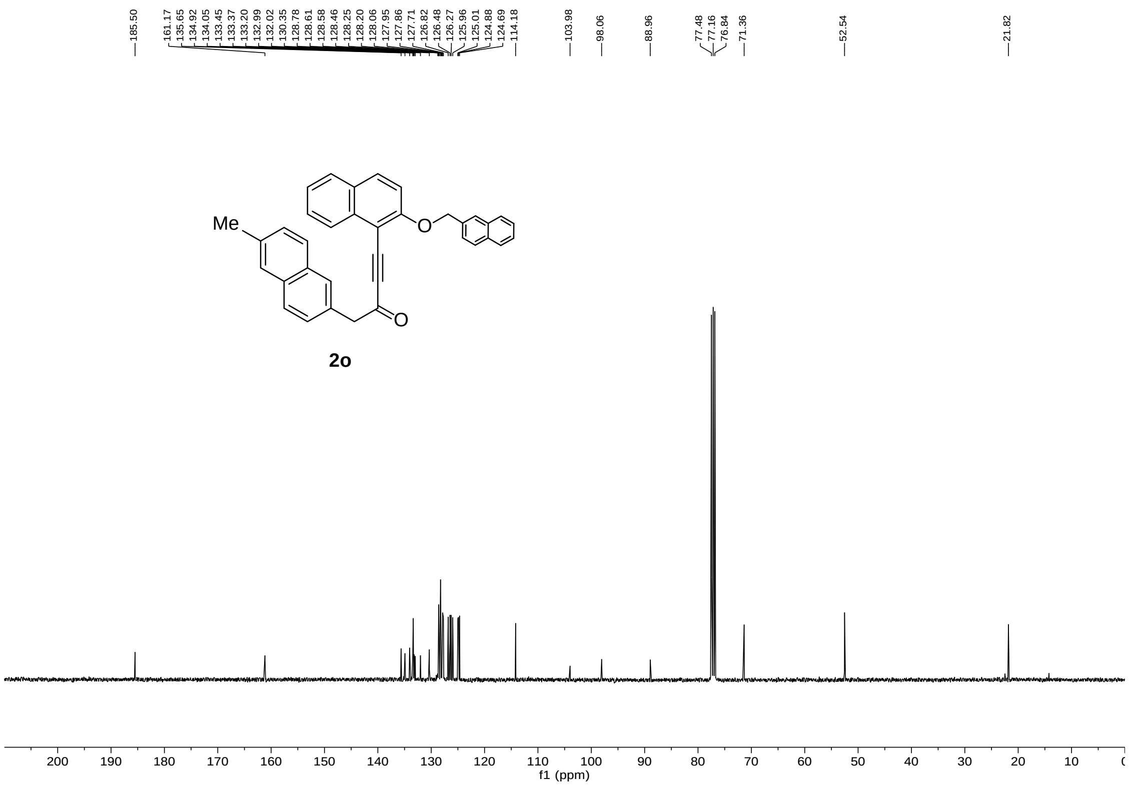

Compound 2p

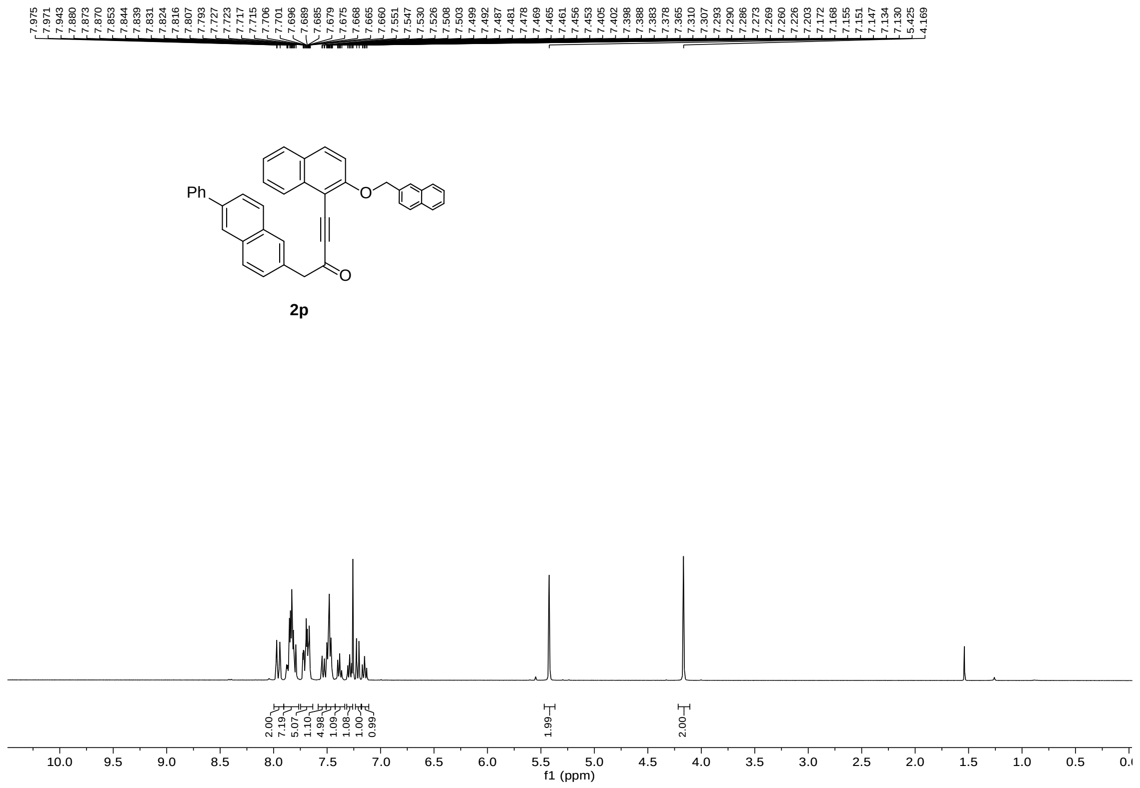
7.975
7.971
7.943
7.880
7.873
7.870
7.853
7.844
7.839
7.831
7.824
7.816
7.807
7.793
7.727
7.723
7.717
7.715
7.706
7.701
7.696
7.689
7.685
7.679
7.675
7.668
7.665
7.660
7.551
7.547
7.530
7.526
7.508
7.503
7.499
7.492
7.487
7.481
7.478
7.469
7.465
7.461
7.456
7.453
7.405
7.402
7.398
7.388
7.383
7.378
7.365
7.310
7.307
7.293
7.290
7.286
7.273
7.269
7.260
7.226
7.203
7.172
7.168
7.155
7.151
7.147
7.134
7.130
5.425
4.169
Ph
O
O
2p
2.00
7.19
5.07
1.10
4.98
1.09
1.08
1.00
0.99
1.99
2.00
f1 (ppm)

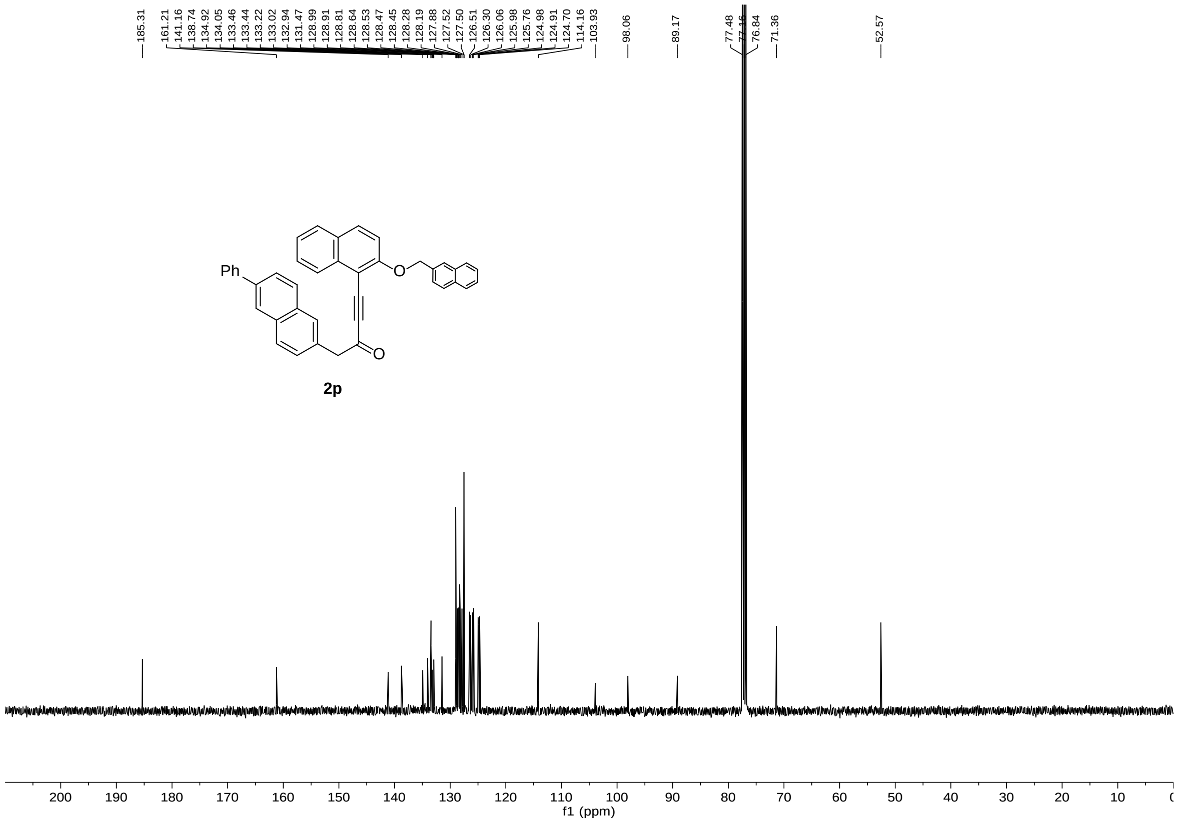
185.31
161.21
141.16
138.74
134.92
134.05
133.46
133.44
133.22
133.02
132.94
131.47
128.99
128.91
128.81
128.64
128.53
128.47
128.45
128.28
128.19
127.88
127.52
127.50
126.51
126.30
126.06
125.98
125.76
124.98
124.91
124.70
114.16
103.93
98.06
89.17
77.48
77.16
76.84
71.36
52.57
Ph
O
O
2p
f1 (ppm)

Compound 2q

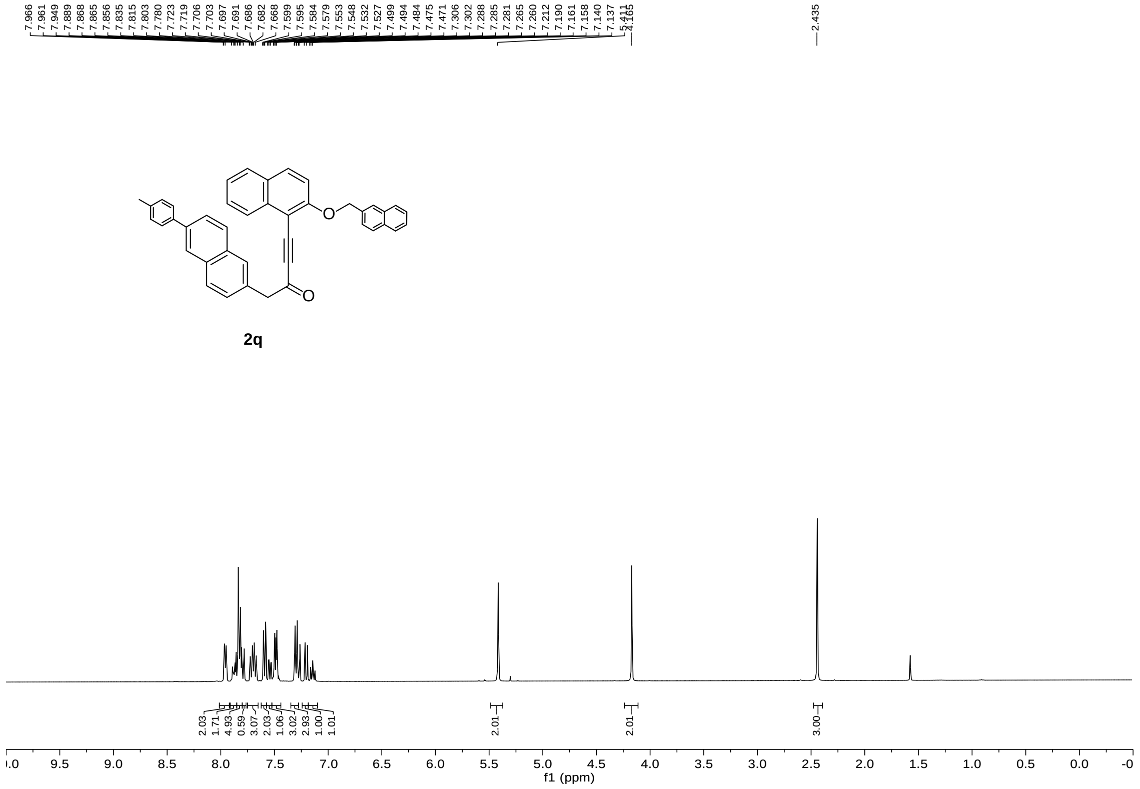

7.966 7.961 7.949 7.889 7.868 7.865 7.856 7.835 7.815 7.803 7.780 7.723 7.719 7.706 7.703 7.697 7.691 7.686 7.682 7.668 7.599 7.595 7.584 7.579 7.553 7.548 7.532 7.527 7.499 7.494 7.484 7.475 7.471 7.306 7.302 7.288 7.285 7.281 7.265 7.260 7.212 7.190 7.161 7.158 7.140 7.137 5.411 4.165
2.435
O
O
2q
2.03 1.71 4.93 0.59 3.07 2.03 1.06 3.02 2.93 1.00 1.01
2.01
2.01
3.00
9.5 9.0 8.5 8.0 7.5 7.0 6.5 6.0 5.5 5.0 4.5 4.0 3.5 3.0 2.5 2.0 1.5 1.0 0.5 0.0
f1 (ppm)

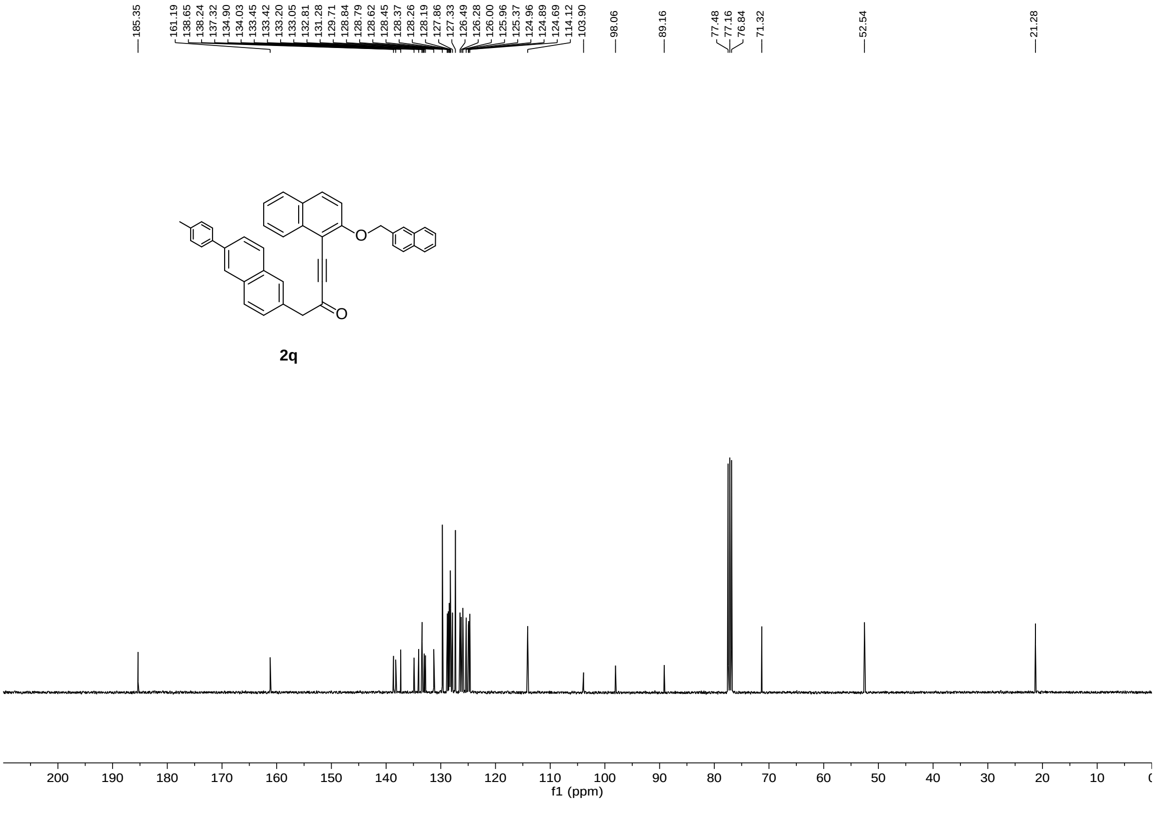

185.35
161.19 138.65 138.24 137.32 134.90 134.03 133.45 133.42 133.20 133.05 132.81 131.28 129.71 128.84 128.79 128.62 128.45 128.37 128.26 128.19 127.86 127.33 126.49 126.28 126.00 125.96 125.37 124.96 124.89 124.69 114.12 103.90
98.06
89.16
77.48 77.16 76.84
71.32
52.54
21.28
O
O
2q
200 190 180 170 160 150 140 130 120 110 100 90 80 70 60 50 40 30 20 10
f1 (ppm)

Compound 2r

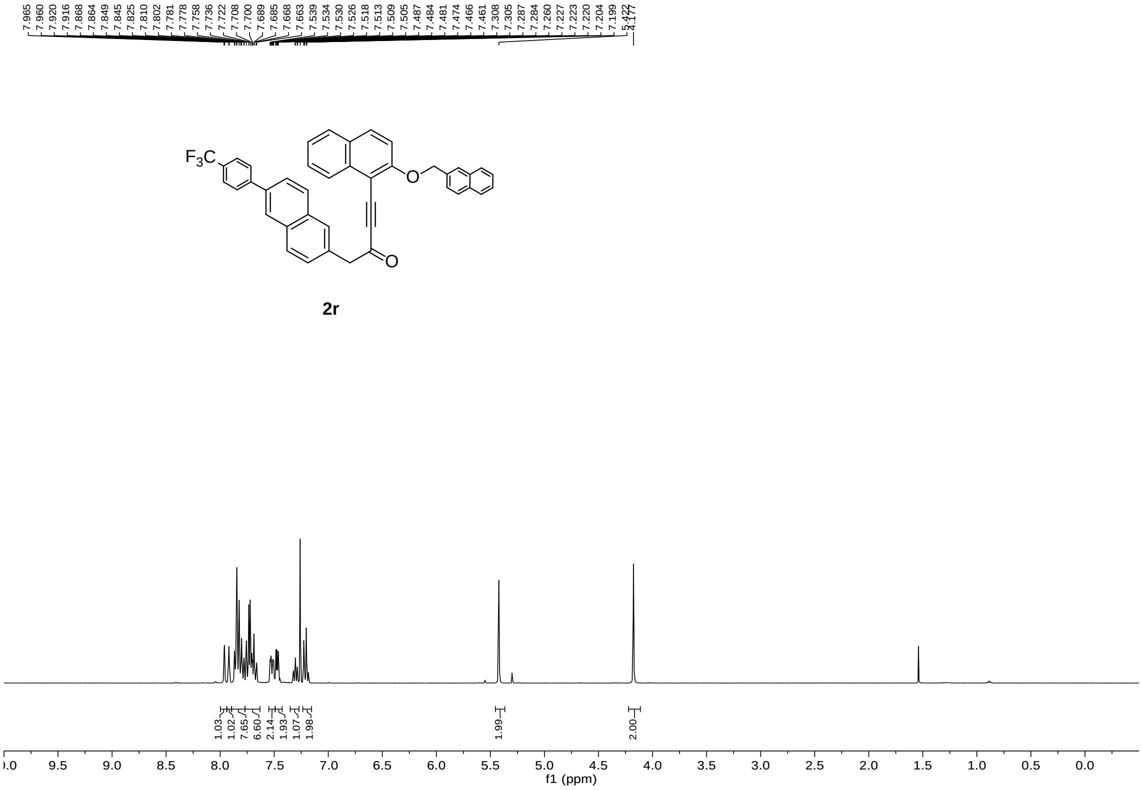

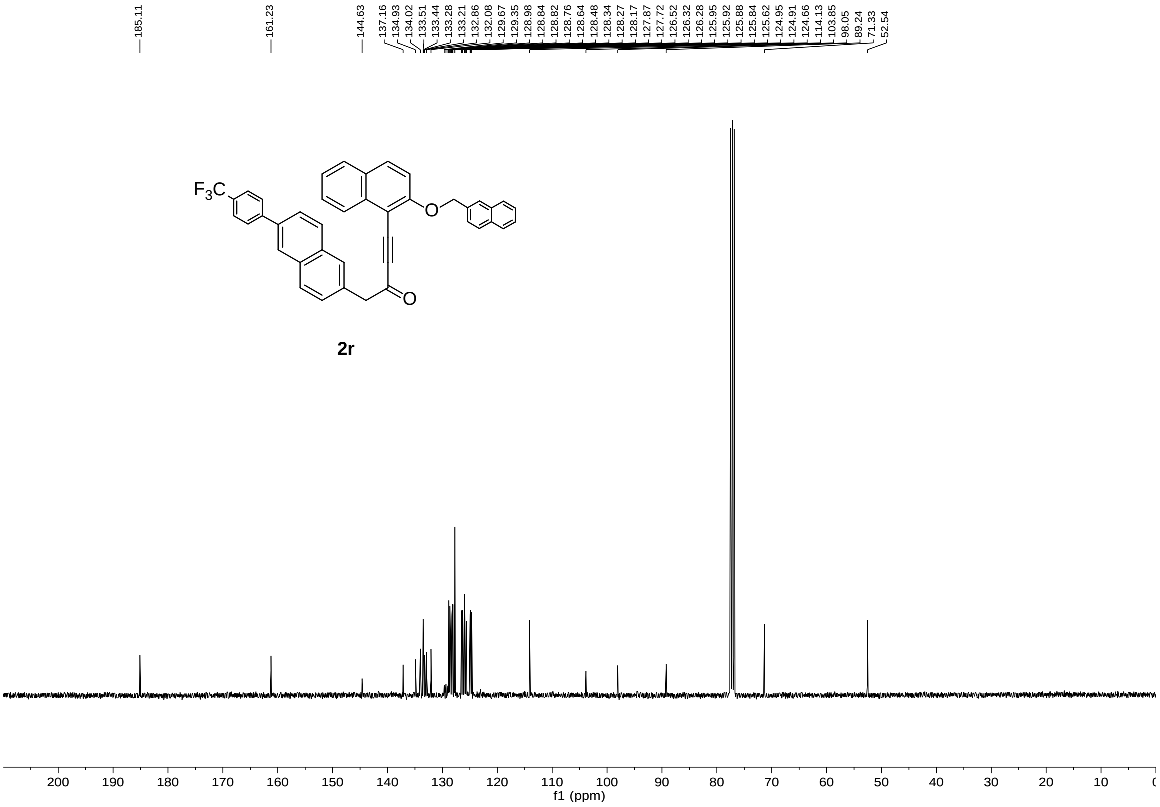

-62.37

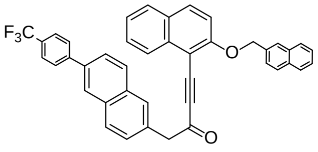
F3C
O
O

2r

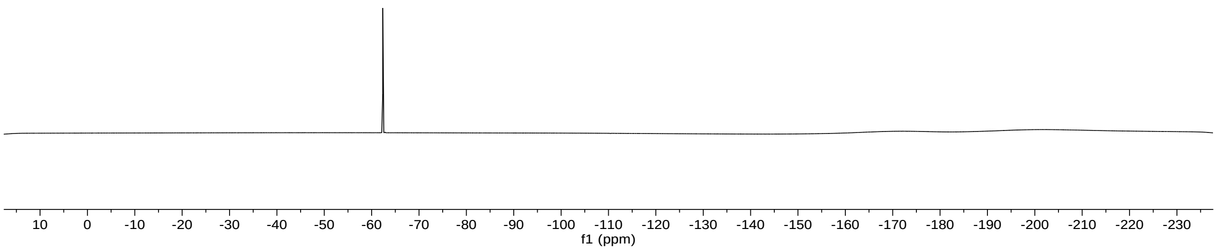
10 0 -10 -20 -30 -40 -50 -60 -70 -80 -90 -100 -110 -120 -130 -140 -150 -160 -170 -180 -190 -200 -210 -220 -230
f1 (ppm)

Compound 2s

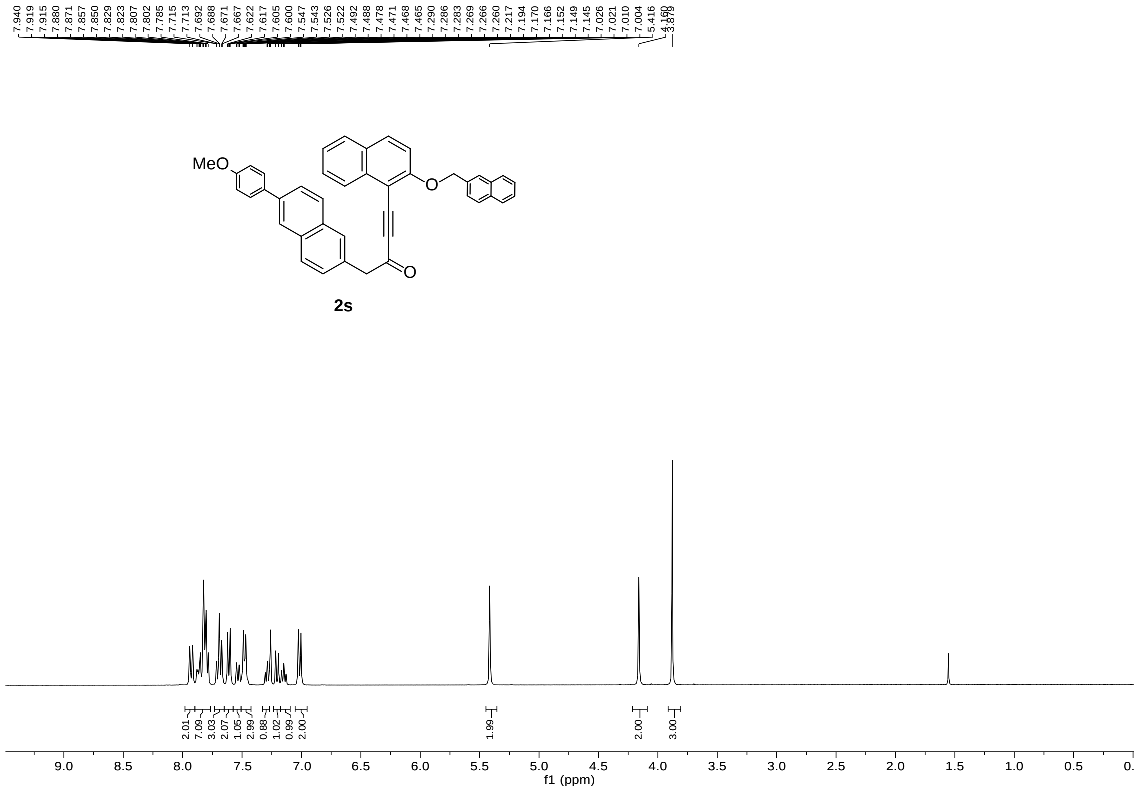
7.940 7.919 7.915 7.880 7.871 7.857 7.850 7.829 7.823 7.807 7.802 7.785 7.715 7.713 7.692 7.688 7.671 7.667 7.622 7.617 7.605 7.600 7.547 7.543 7.526 7.522 7.492 7.488 7.478 7.471 7.468 7.465 7.290 7.286 7.283 7.269 7.266 7.260 7.217 7.194 7.170 7.166 7.152 7.149 7.145 7.026 7.021 7.010 7.004 5.416 4.160 3.879
MeO
O
O
2s
2.01 7.09 3.03 2.07 1.05 2.99 0.88 1.02 0.99 2.00 1.99 2.00 3.00
9.0 8.5 8.0 7.5 7.0 6.5 6.0 5.5 5.0 4.5 4.0 3.5 3.0 2.5 2.0 1.5 1.0 0.5 0.
f1 (ppm)

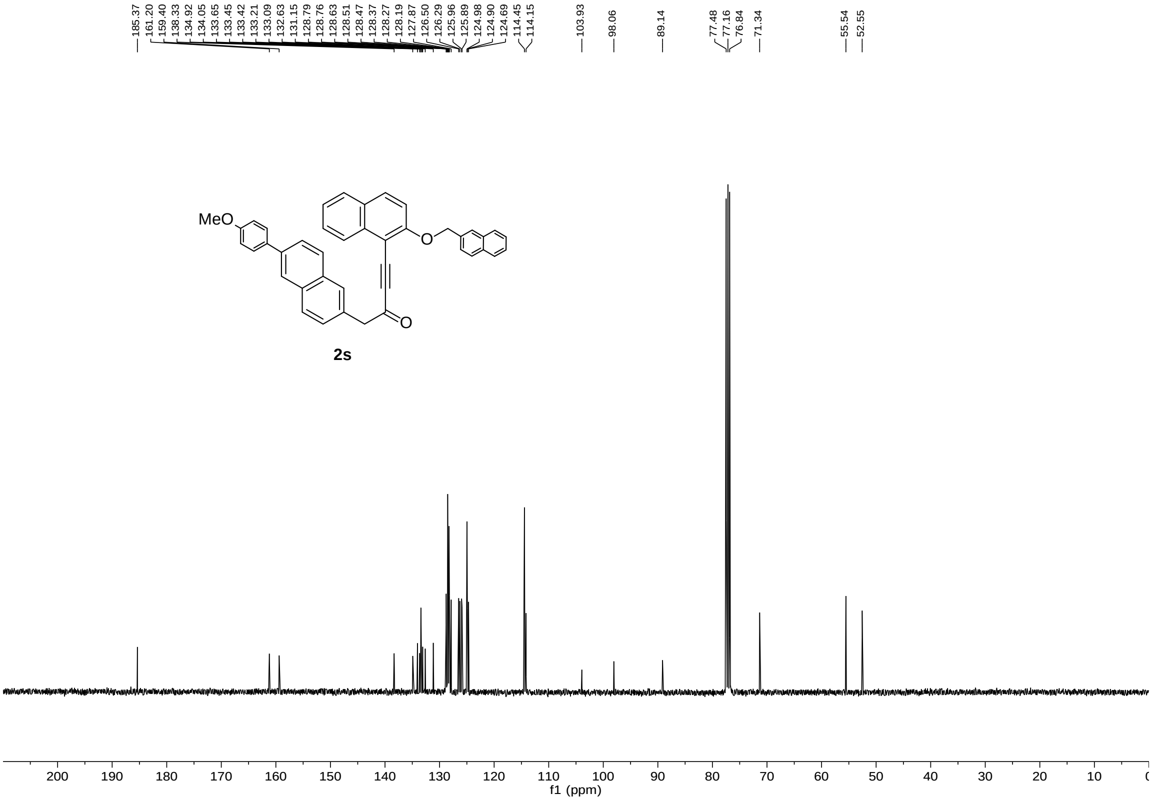
185.37 161.20 159.40 138.33 134.92 134.05 133.65 133.45 133.42 133.21 133.09 132.63 131.15 128.79 128.76 128.63 128.51 128.47 128.37 128.27 128.19 127.87 126.50 126.29 125.96 125.89 124.98 124.90 124.69 114.45 114.15 103.93 98.06 89.14 77.48 77.16 76.84 71.34 55.54 52.55
MeO
O
O
2s
200 190 180 170 160 150 140 130 120 110 100 90 80 70 60 50 40 30 20 10
f1 (ppm)

Compound 2t

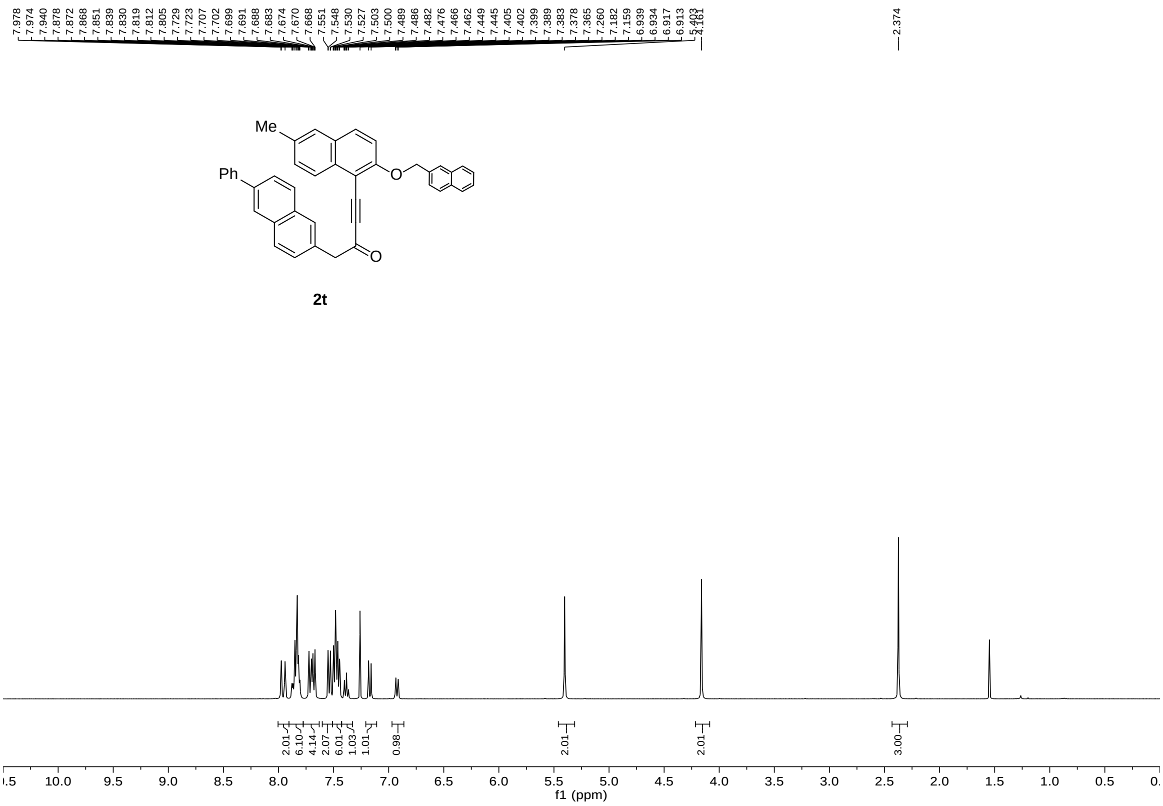
7.978
7.974
7.940
7.878
7.872
7.868
7.851
7.839
7.830
7.819
7.812
7.805
7.729
7.723
7.707
7.702
7.699
7.691
7.688
7.683
7.674
7.670
7.668
7.551
7.548
7.530
7.527
7.503
7.500
7.489
7.486
7.482
7.476
7.466
7.462
7.449
7.445
7.405
7.402
7.399
7.389
7.383
7.378
7.365
7.260
7.182
7.159
6.939
6.934
6.917
6.913
5.403
4.161
2.374
Me
Ph
O
O
2t
2.01
6.10
4.14
2.07
6.01
1.03
1.01
0.98
2.01
2.01
3.00
f1 (ppm)

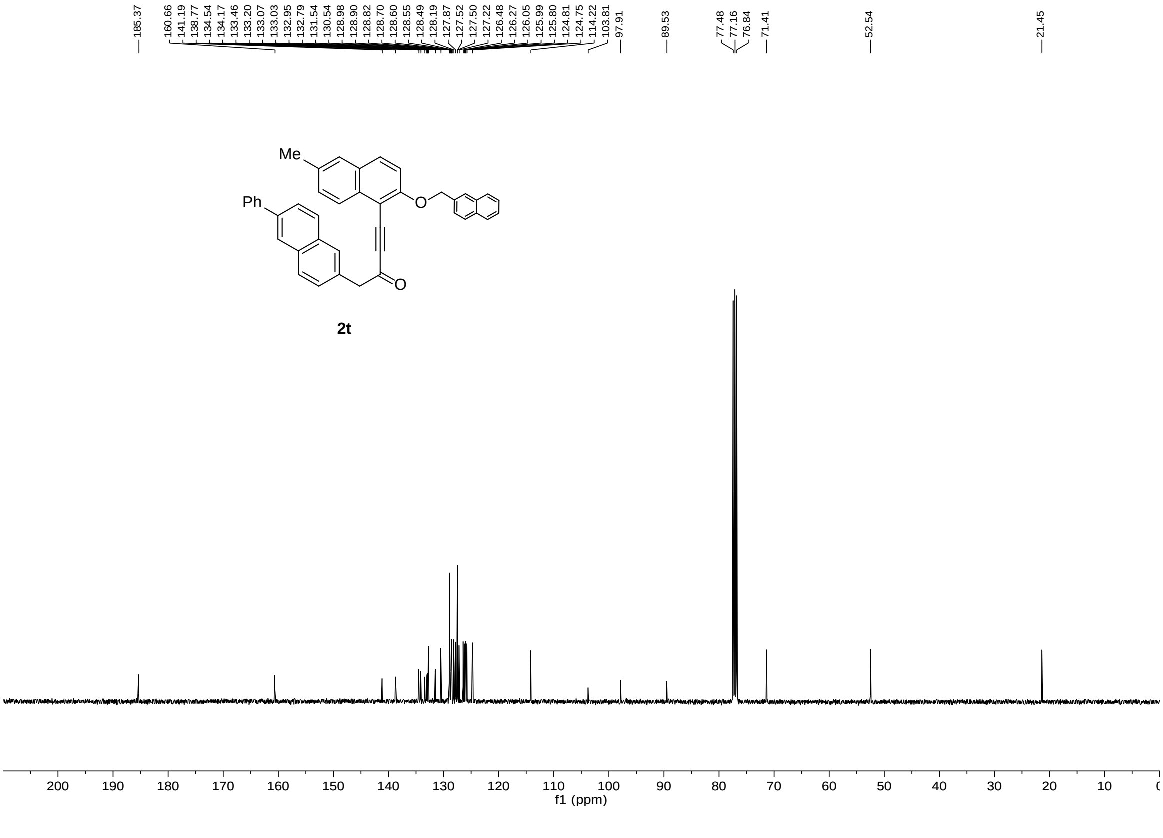
185.37
160.66
141.19
138.77
134.54
134.17
133.46
133.20
133.07
133.03
132.95
132.79
131.54
130.54
128.98
128.90
128.82
128.70
128.60
128.55
128.49
128.19
127.87
127.52
127.50
127.22
126.48
126.27
126.05
125.99
125.80
124.81
124.75
114.22
103.81
97.91
89.53
77.48
77.16
76.84
71.41
52.54
21.45
Me
Ph
O
O
2t
f1 (ppm)

Compound 2u

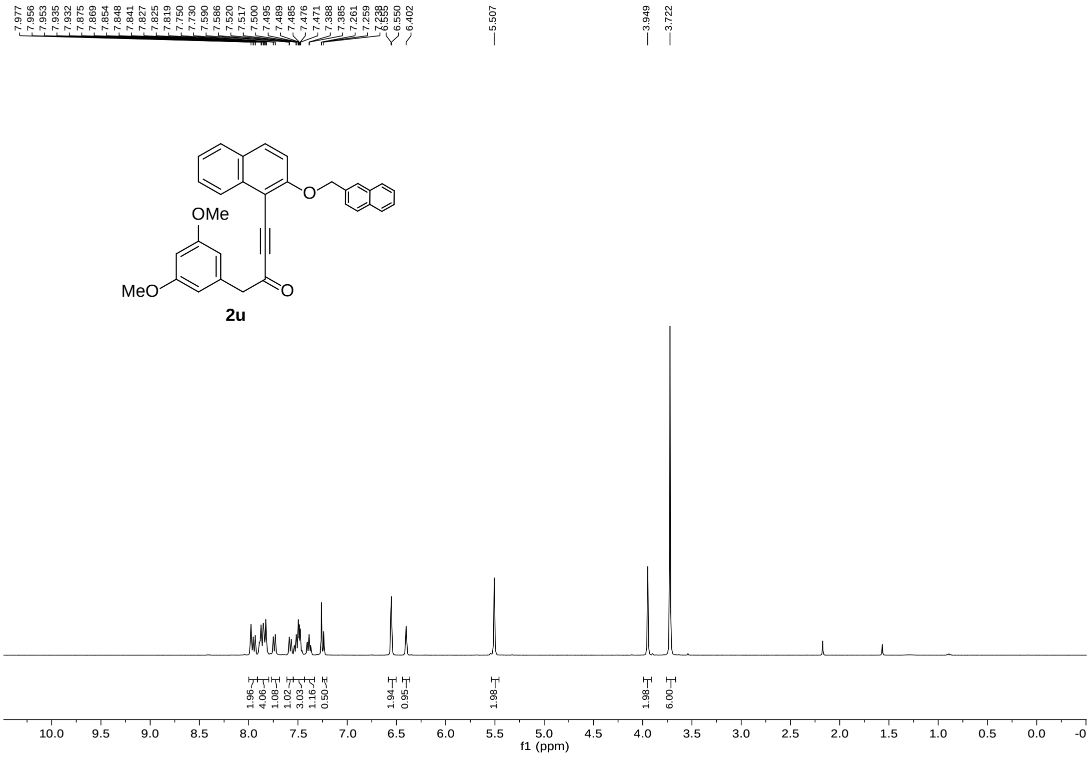

7.977
7.956
7.953
7.935
7.932
7.875
7.869
7.854
7.848
7.841
7.827
7.825
7.819
7.750
7.730
7.590
7.586
7.520
7.517
7.500
7.495
7.489
7.485
7.476
7.471
7.388
7.385
7.261
7.259
7.238
6.555
6.550
6.402
5.507
3.949
3.722
OMe
O
MeO
O
2u
1.96
4.06
1.08
1.02
3.03
1.16
0.50
1.94
0.95
1.98
1.98
6.00
10.0 9.5 9.0 8.5 8.0 7.5 7.0 6.5 6.0 5.5 5.0 4.5 4.0 3.5 3.0 2.5 2.0 1.5 1.0 0.5 0.0 -0
f1 (ppm)

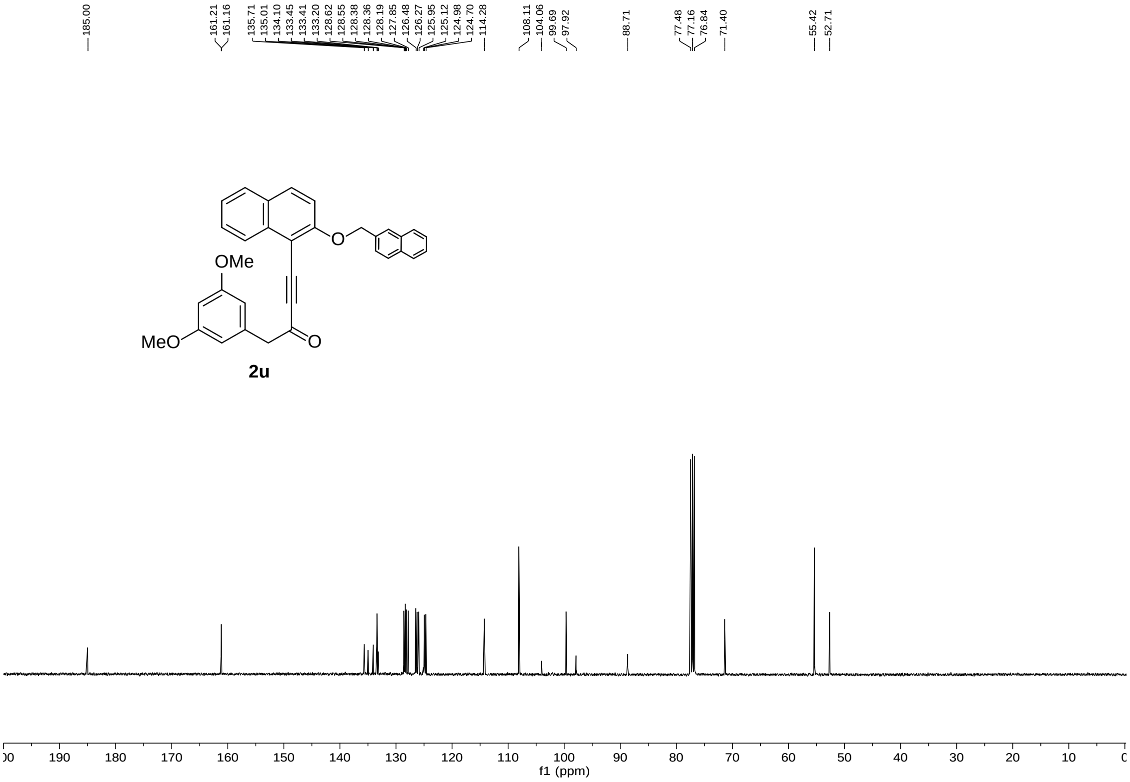

185.00
161.21
161.16
135.71
135.01
134.10
133.45
133.41
133.20
128.62
128.55
128.38
128.36
128.19
127.85
126.48
126.27
125.95
125.12
124.98
124.70
114.28
108.11
104.06
99.69
97.92
88.71
77.48
77.16
76.84
71.40
55.42
52.71
OMe
O
MeO
O
2u
190 180 170 160 150 140 130 120 110 100 90 80 70 60 50 40 30 20 10
f1 (ppm)

Compound 4a

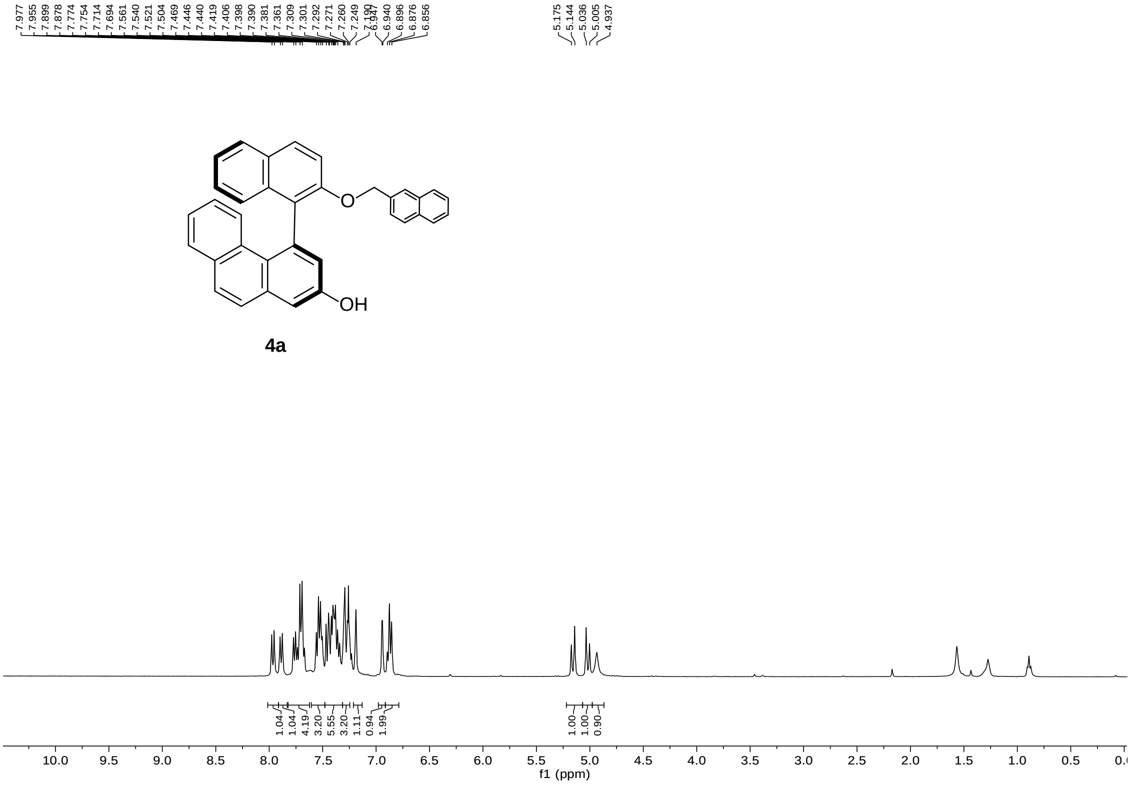

7.977
7.955
7.899
7.878
7.774
7.754
7.714
7.694
7.561
7.540
7.521
7.504
7.469
7.446
7.440
7.419
7.406
7.398
7.390
7.381
7.361
7.309
7.301
7.292
7.271
7.260
7.249
6.940
6.896
6.876
6.856
5.175
5.144
5.036
5.005
4.937
O
OH
4a
1.04
1.04
4.19
3.20
5.55
3.20
1.11
0.94
1.99
1.00
1.00
0.90
f1 (ppm)

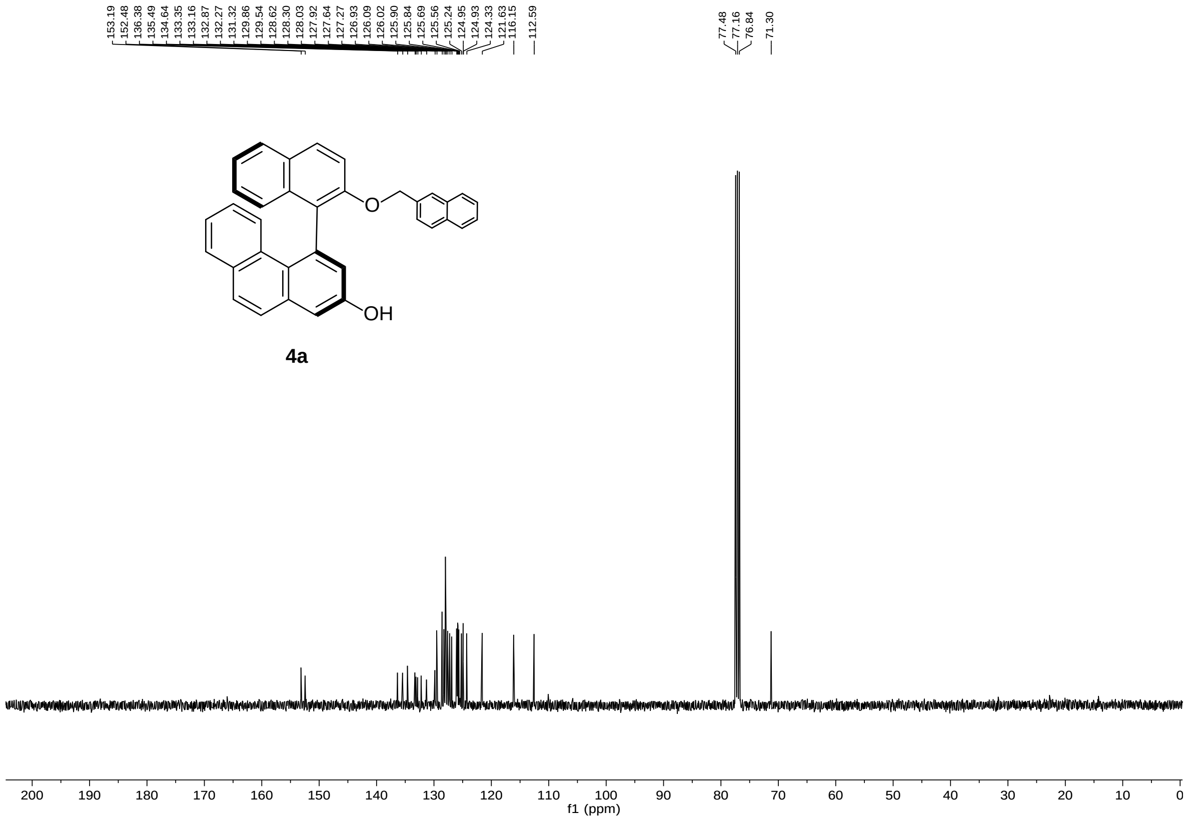

153.19
152.48
136.38
135.49
134.64
133.35
133.16
132.87
132.27
131.32
129.86
129.54
128.62
128.30
128.03
127.92
127.64
127.27
126.93
126.09
126.02
125.90
125.84
125.69
125.56
125.24
124.95
124.93
124.33
121.63
116.15
112.59
77.48
77.16
76.84
71.30
O
OH
4a
f1 (ppm)

Compound 4b

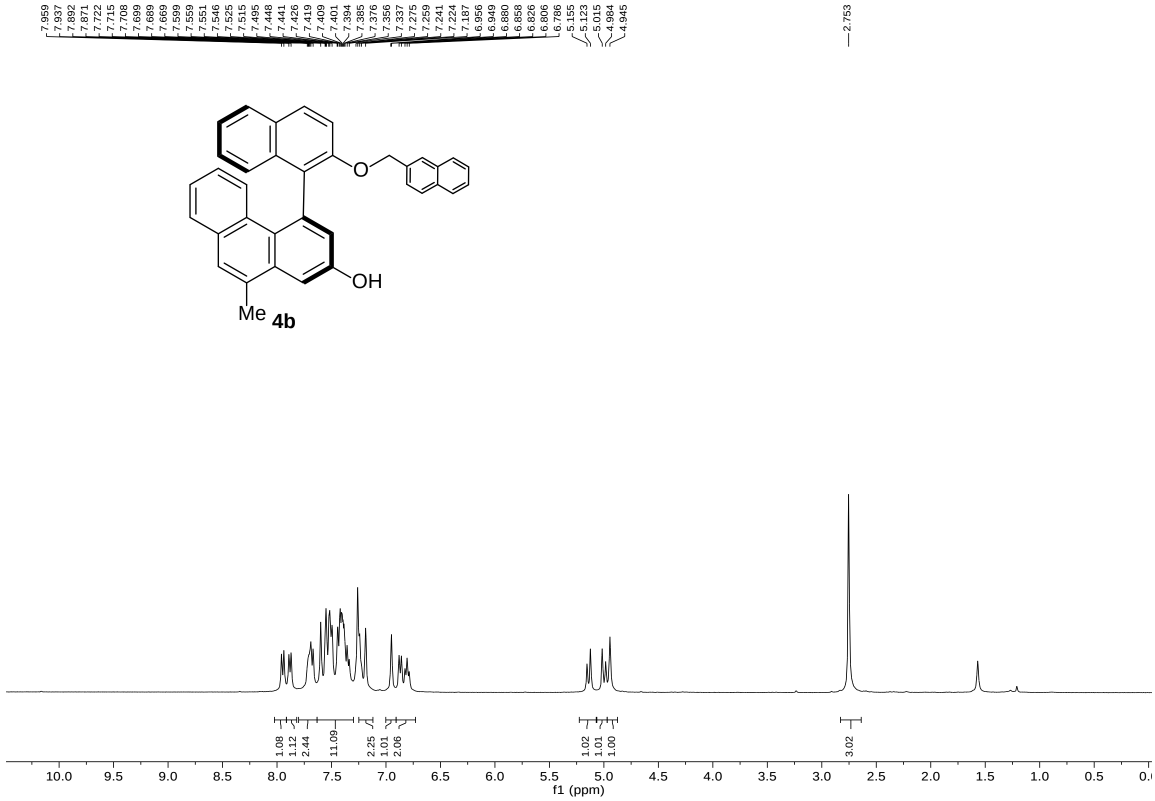
7.959
7.937
7.892
7.871
7.722
7.715
7.708
7.699
7.689
7.669
7.599
7.559
7.551
7.546
7.525
7.515
7.495
7.448
7.441
7.426
7.419
7.409
7.401
7.394
7.385
7.376
7.356
7.337
7.275
7.259
7.241
7.224
7.187
6.956
6.949
6.880
6.858
6.826
6.806
6.786
5.155
5.123
5.015
4.984
4.945
2.753
O
OH
Me
4b
1.08
1.12
2.44
11.09
2.25
1.01
2.06
1.02
1.01
1.00
3.02
f1 (ppm)

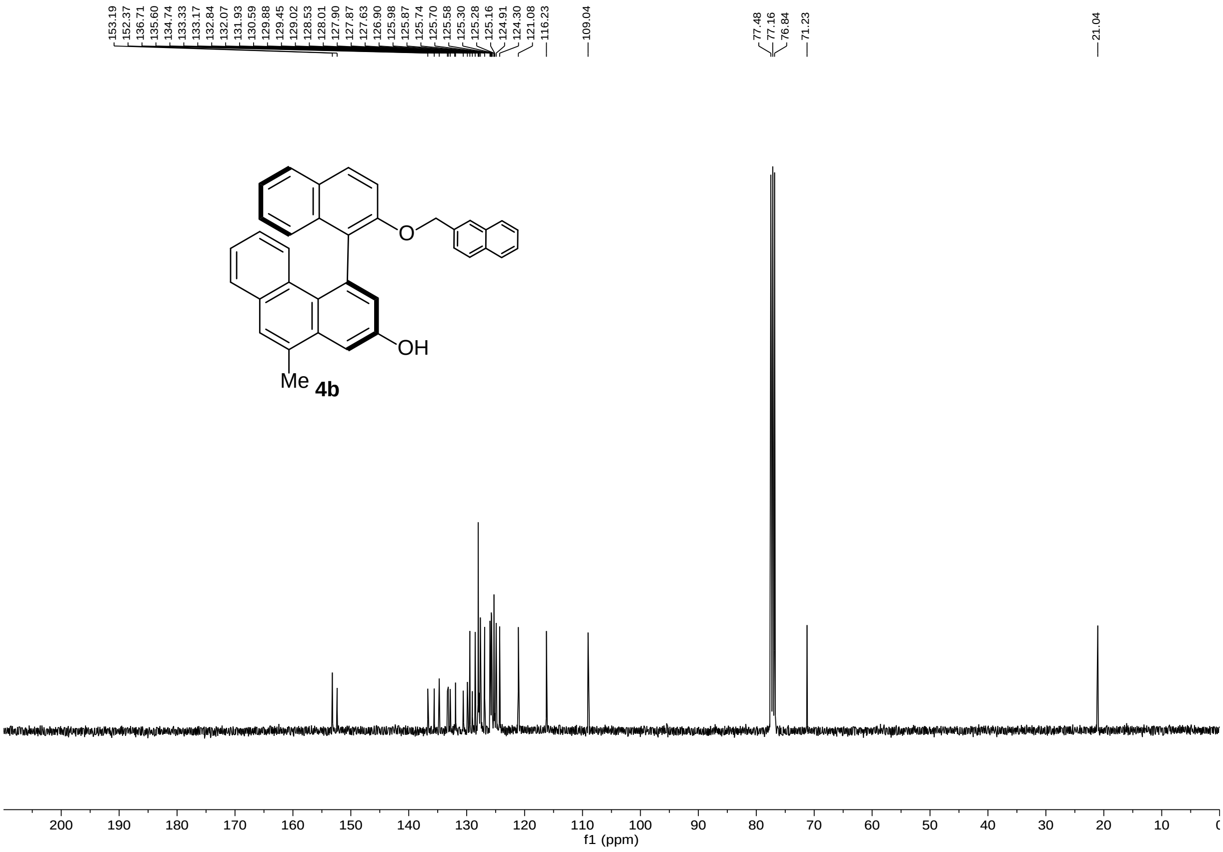
153.19
152.37
136.71
135.60
134.74
133.33
133.17
132.84
132.07
131.93
130.59
129.88
129.45
129.02
128.53
128.01
127.90
127.87
127.63
126.90
125.98
125.87
125.74
125.70
125.58
125.30
125.28
125.16
124.91
124.30
121.08
116.23
109.04
77.48
77.16
76.84
71.23
21.04
O
OH
Me
4b
f1 (ppm)

Compound 4c

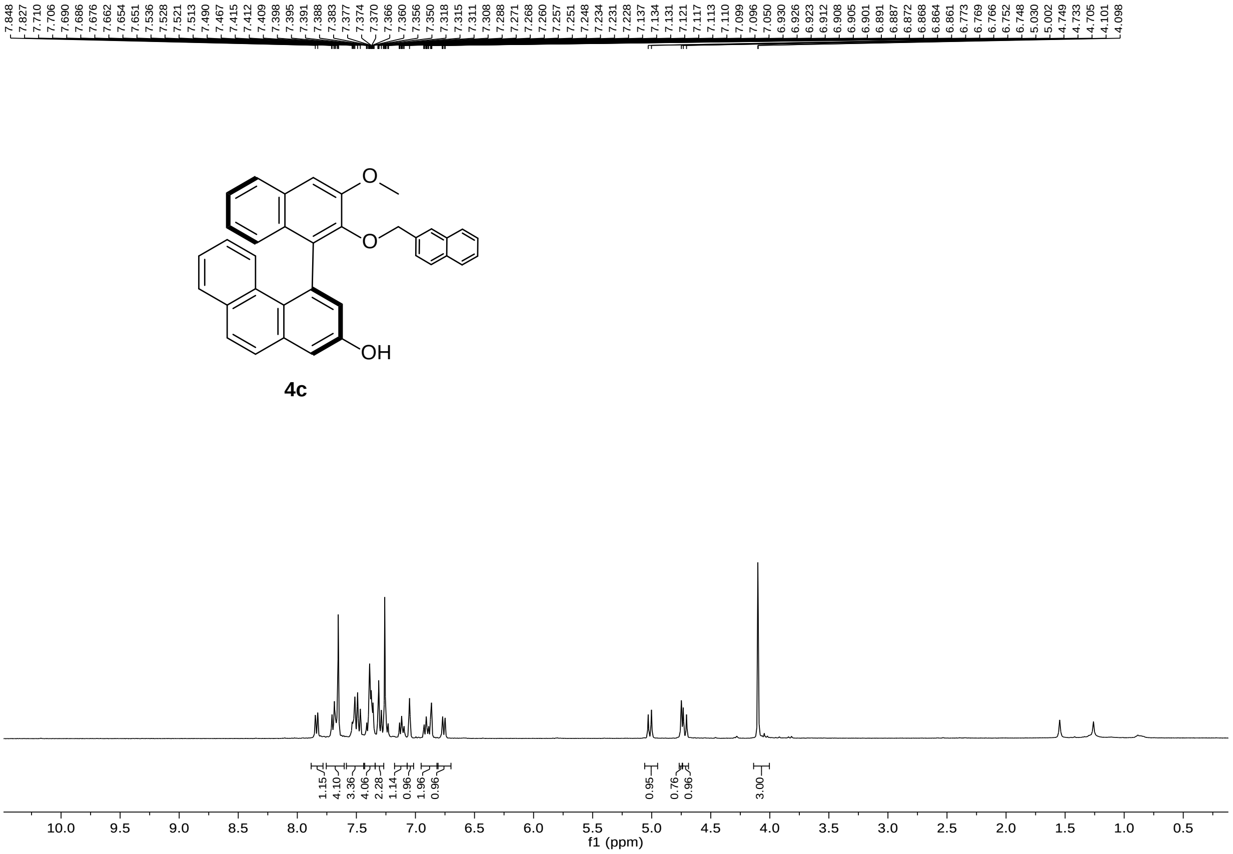
7.848
7.827
7.710
7.706
7.690
7.686
7.676
7.662
7.654
7.651
7.536
7.528
7.521
7.513
7.490
7.467
7.415
7.412
7.409
7.398
7.395
7.391
7.388
7.383
7.377
7.374
7.370
7.366
7.360
7.356
7.350
7.318
7.315
7.311
7.308
7.288
7.271
7.268
7.260
7.257
7.251
7.248
7.234
7.231
7.228
7.137
7.134
7.131
7.121
7.117
7.113
7.110
7.099
7.096
7.050
6.930
6.926
6.923
6.912
6.908
6.905
6.901
6.891
6.887
6.872
6.868
6.864
6.861
6.773
6.769
6.766
6.752
6.748
5.030
5.002
4.749
4.733
4.705
4.101
4.098
O
O
OH
4c
1.15
4.10
3.36
4.06
2.28
1.14
0.96
1.96
0.96
0.95
0.76
0.96
3.00
10.0
9.5
9.0
8.5
8.0
7.5
7.0
6.5
6.0
5.5
5.0
4.5
4.0
3.5
3.0
2.5
2.0
1.5
1.0
0.5
f1 (ppm)

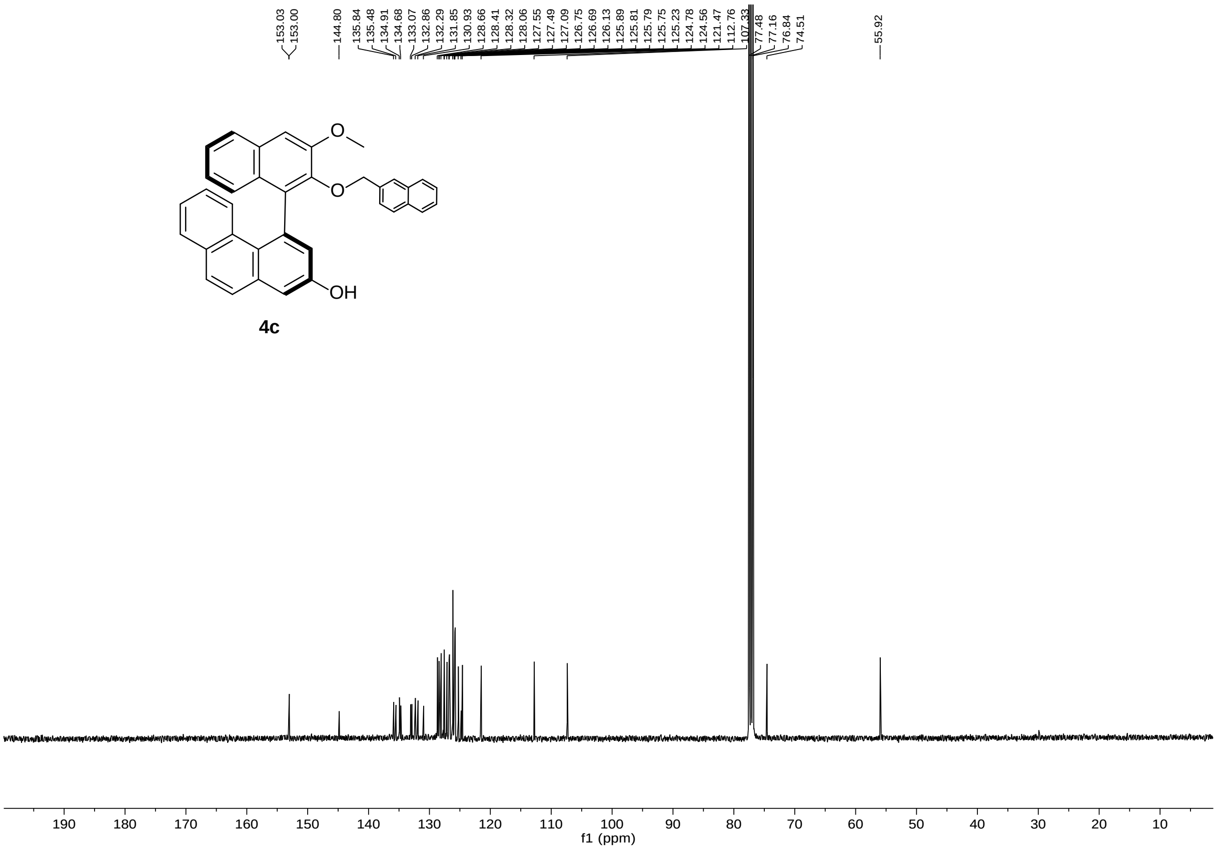
153.03
153.00
144.80
135.84
135.48
134.91
134.68
133.07
132.86
132.29
131.85
130.93
128.66
128.41
128.32
128.06
127.55
127.49
127.09
126.75
126.69
126.13
125.89
125.81
125.79
125.75
125.23
124.78
124.56
121.47
112.76
107.33
77.48
77.16
76.84
74.51
55.92
O
O
OH
4c
190
180
170
160
150
140
130
120
110
100
90
80
70
60
50
40
30
20
10
f1 (ppm)

Compound 4d

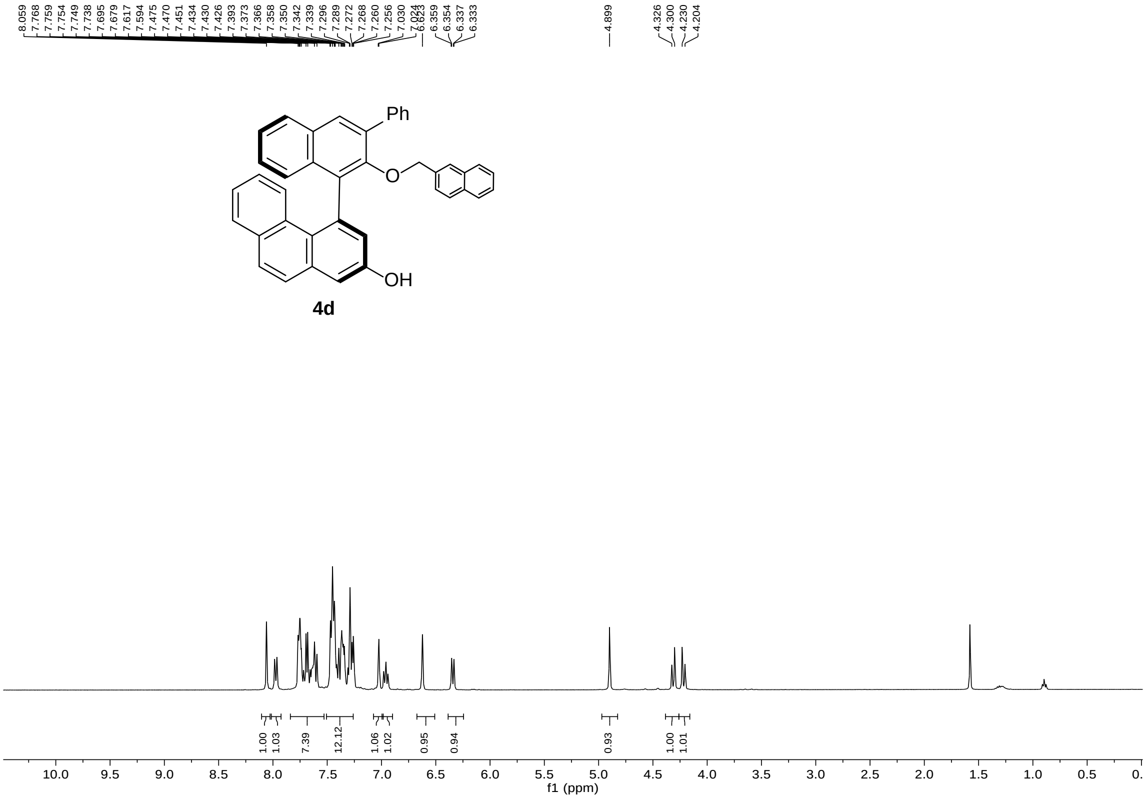

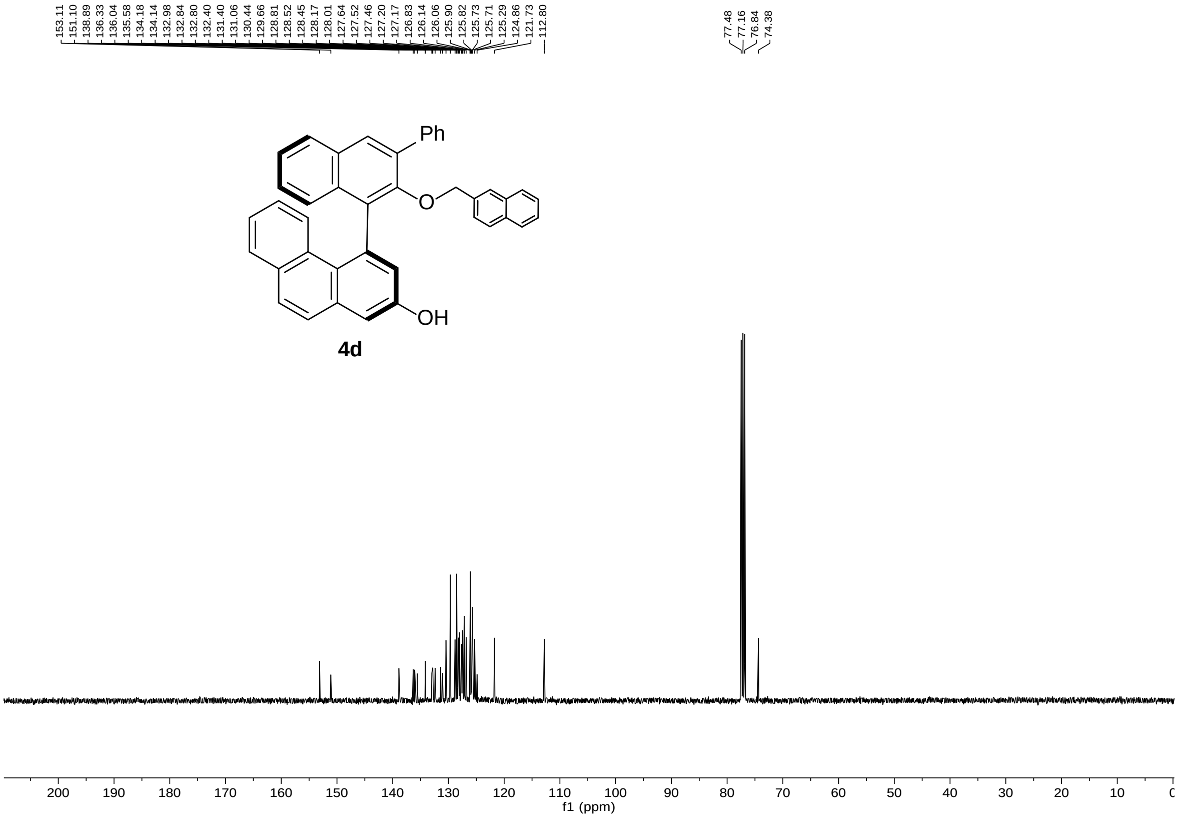

Compound 4e

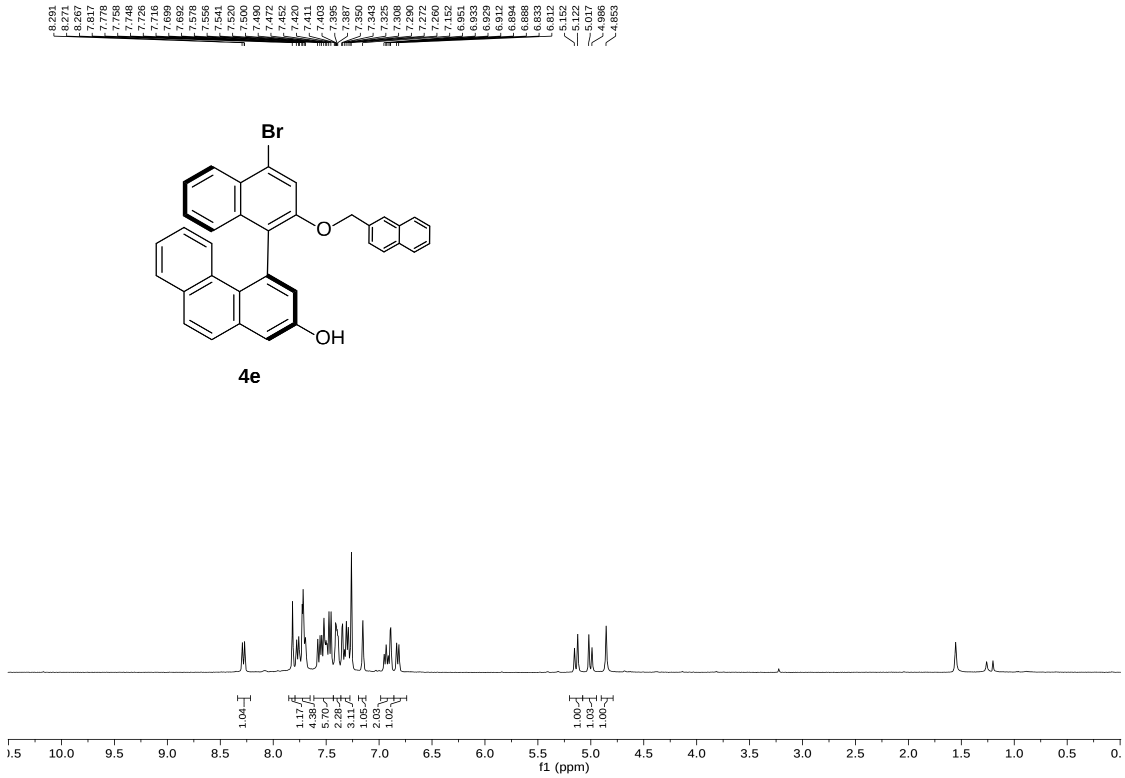

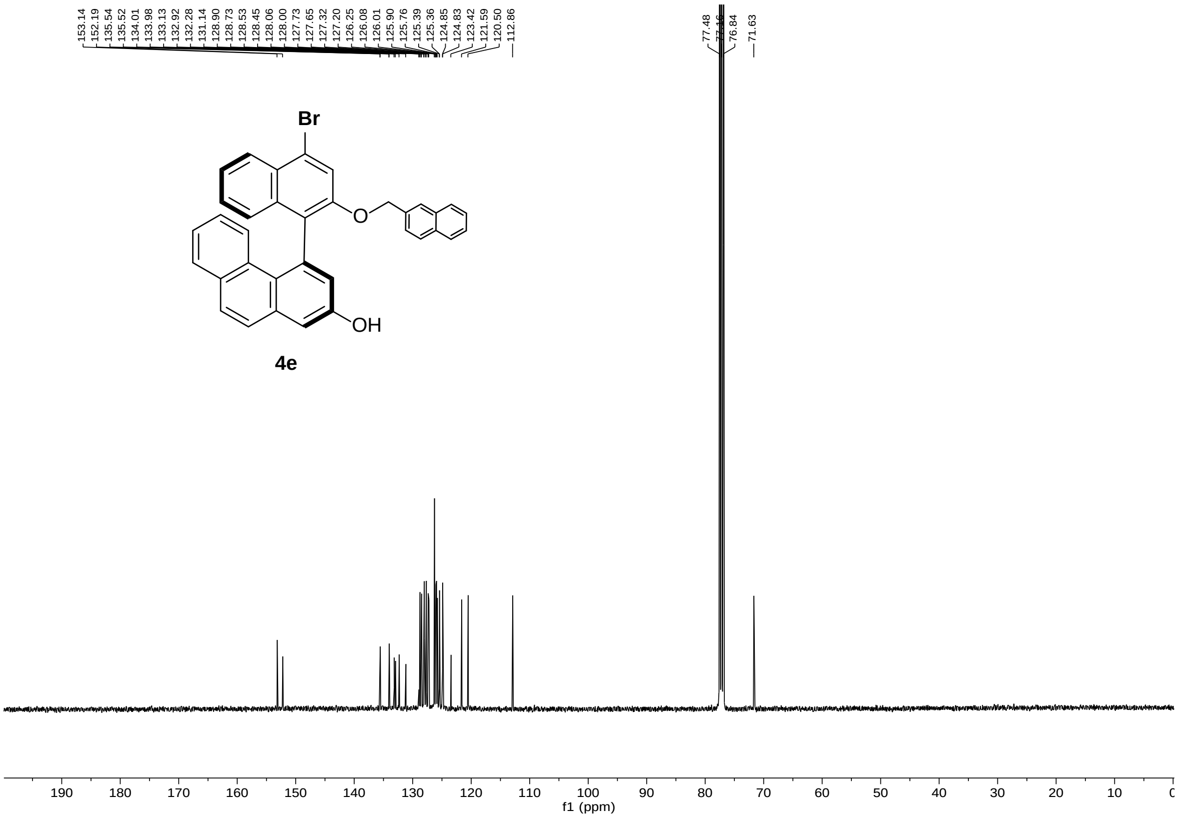

Compound 4f

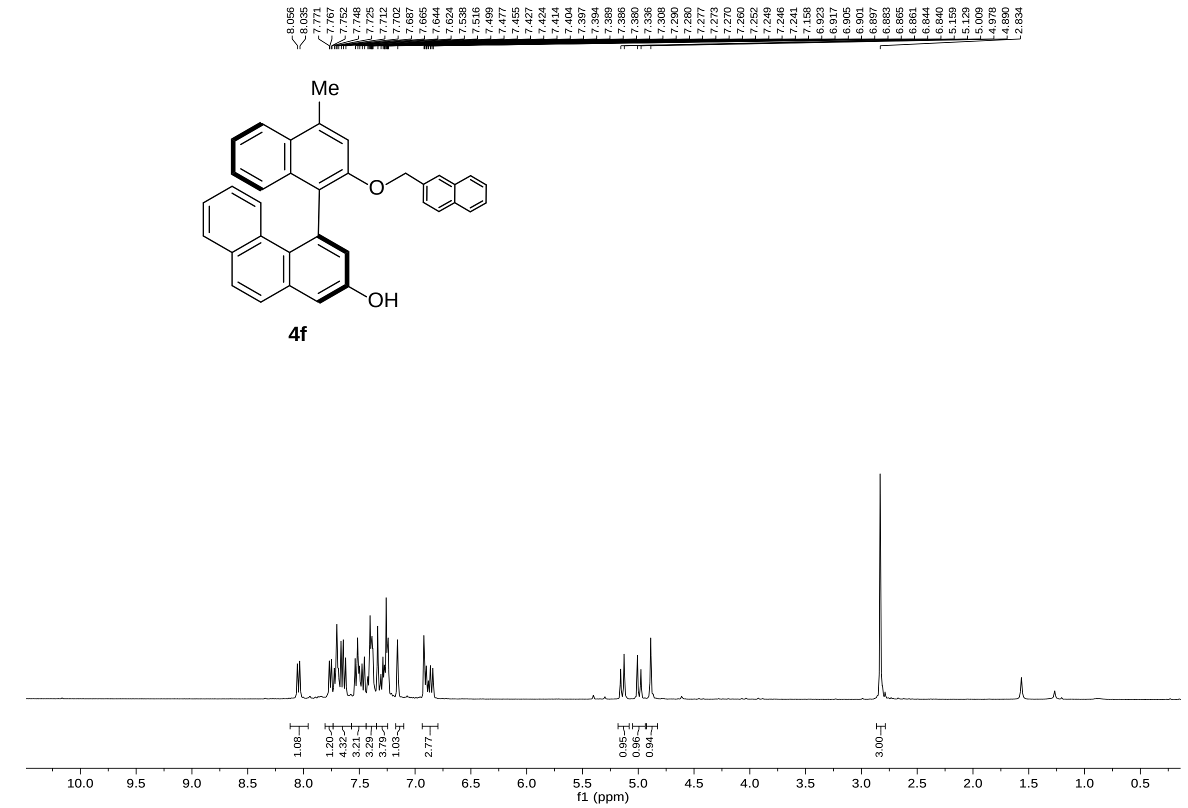

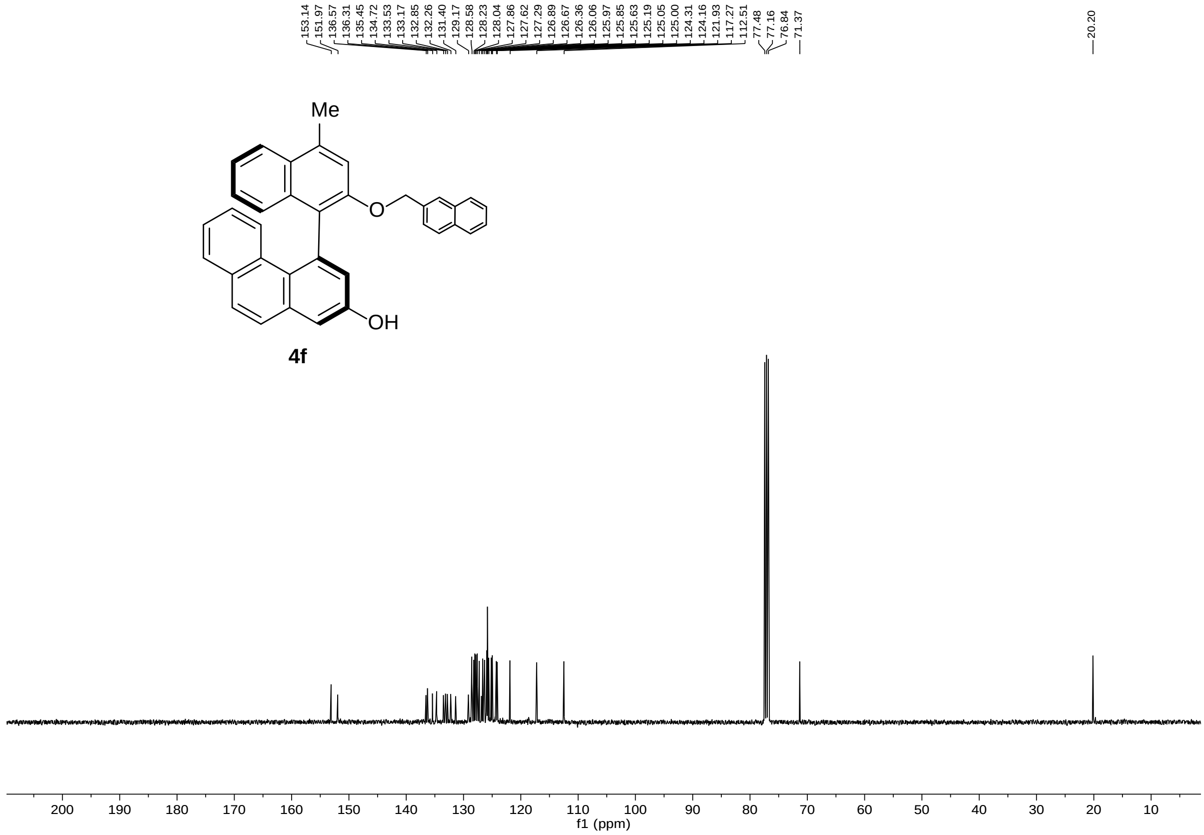

Compound 4g

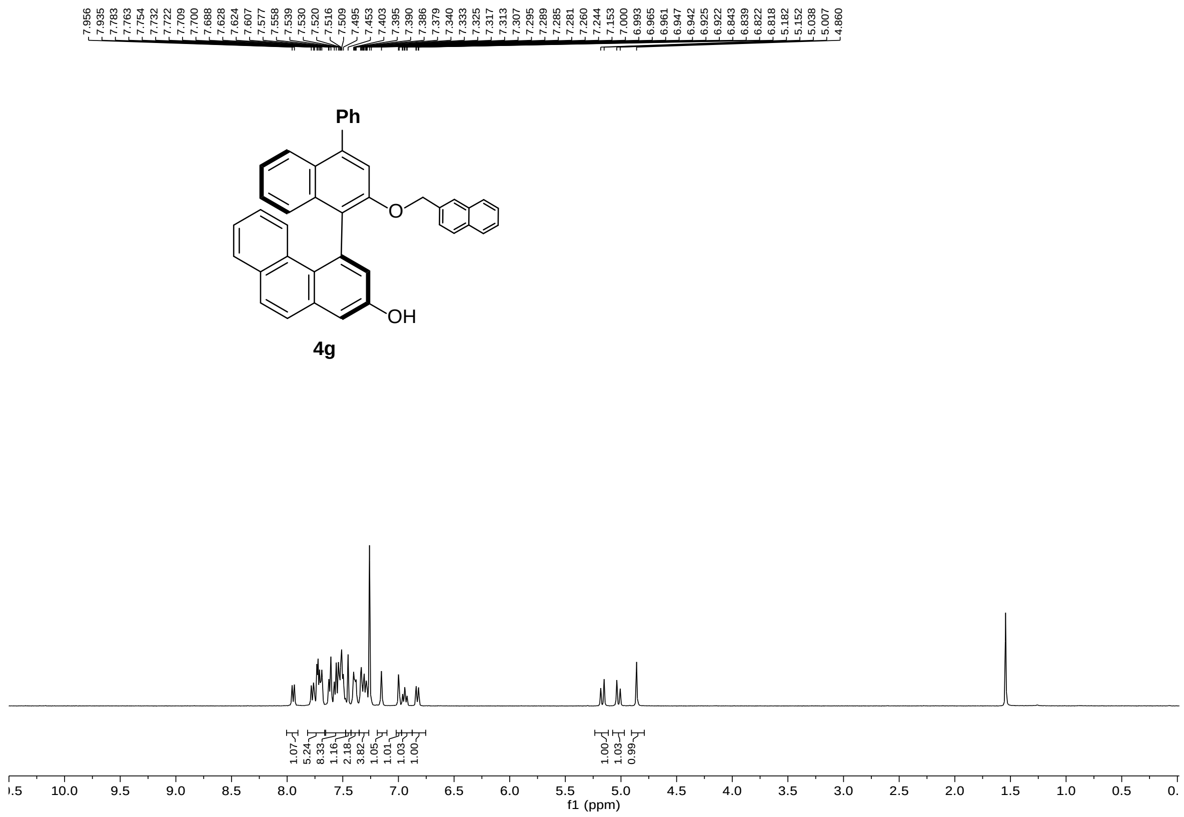

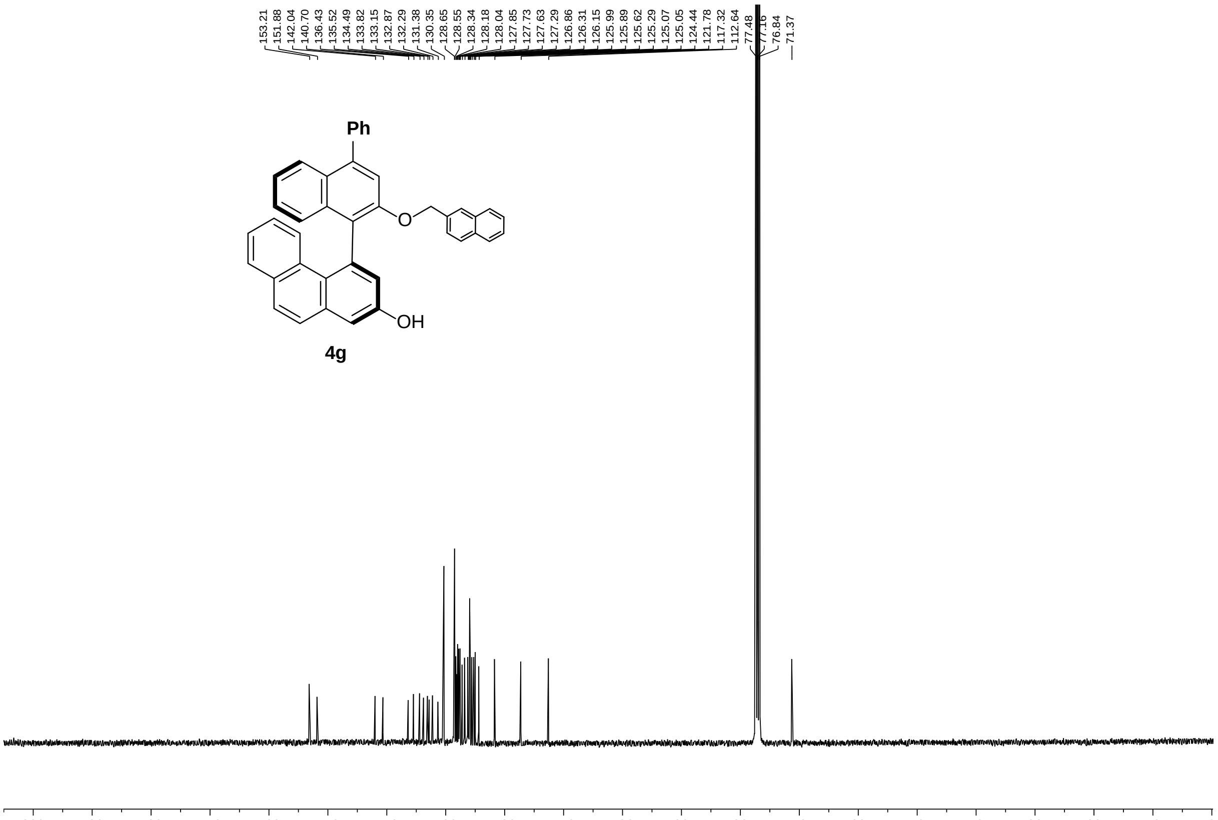

Compound 4h

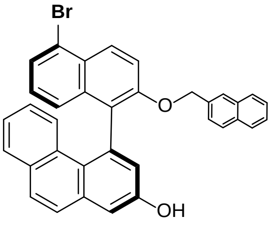

4h

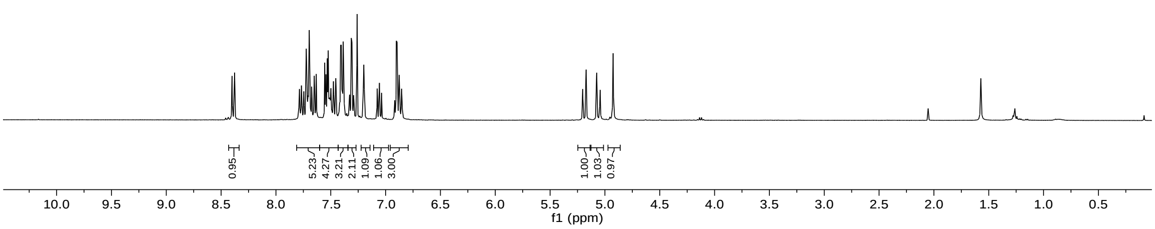

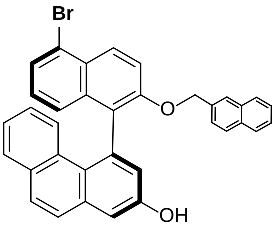

4h

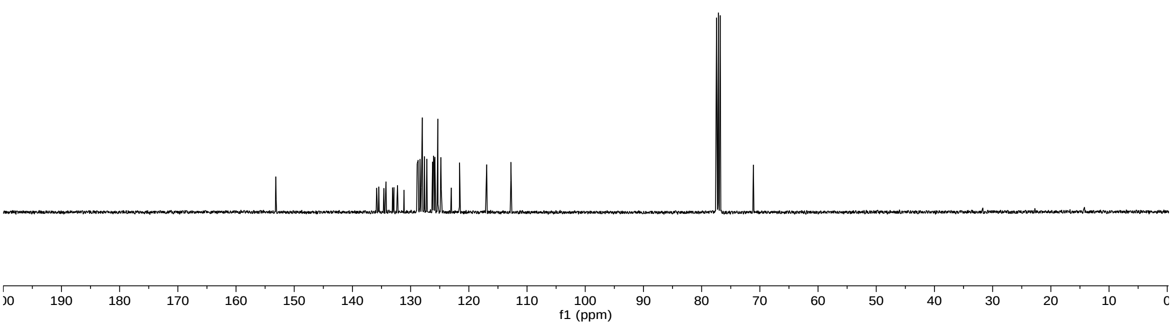

Compound 4i

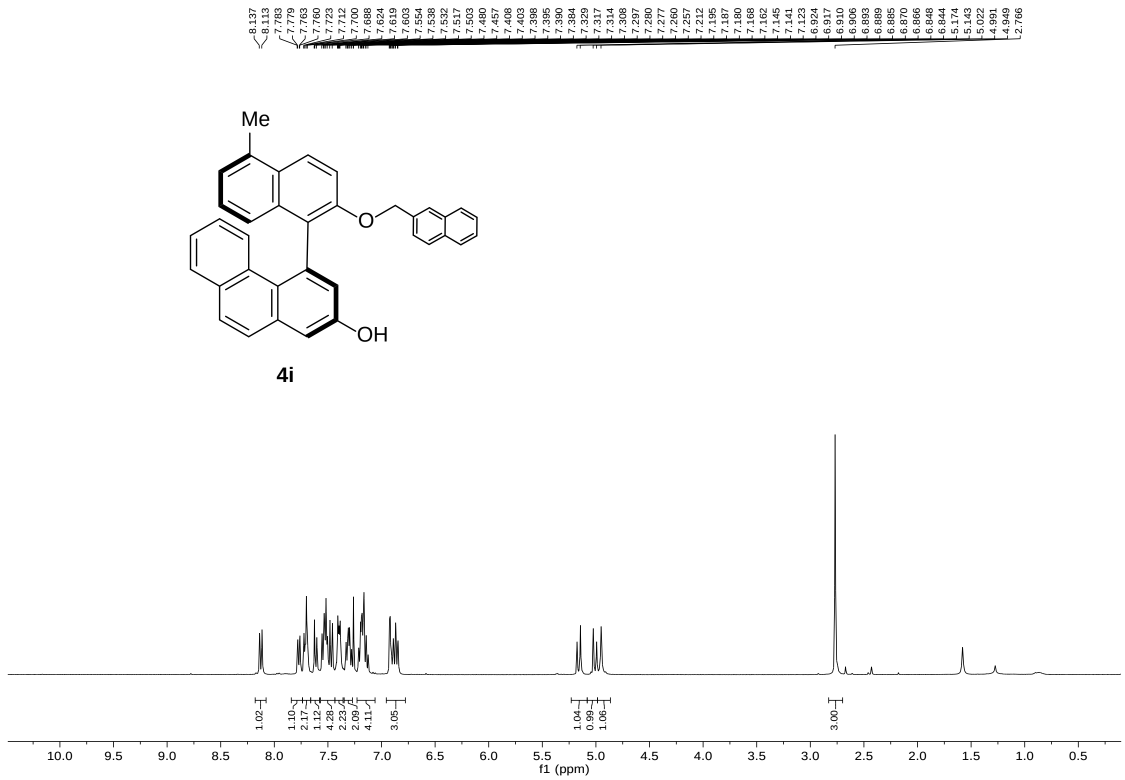

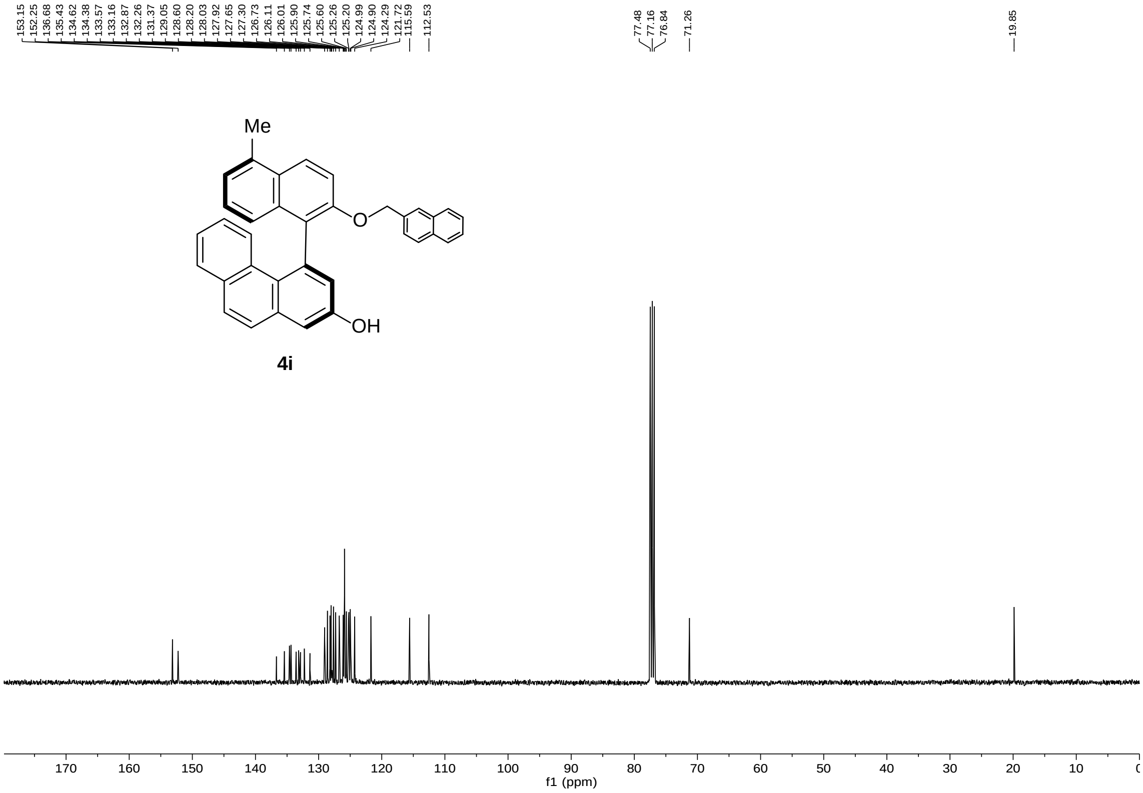

Compound 4j

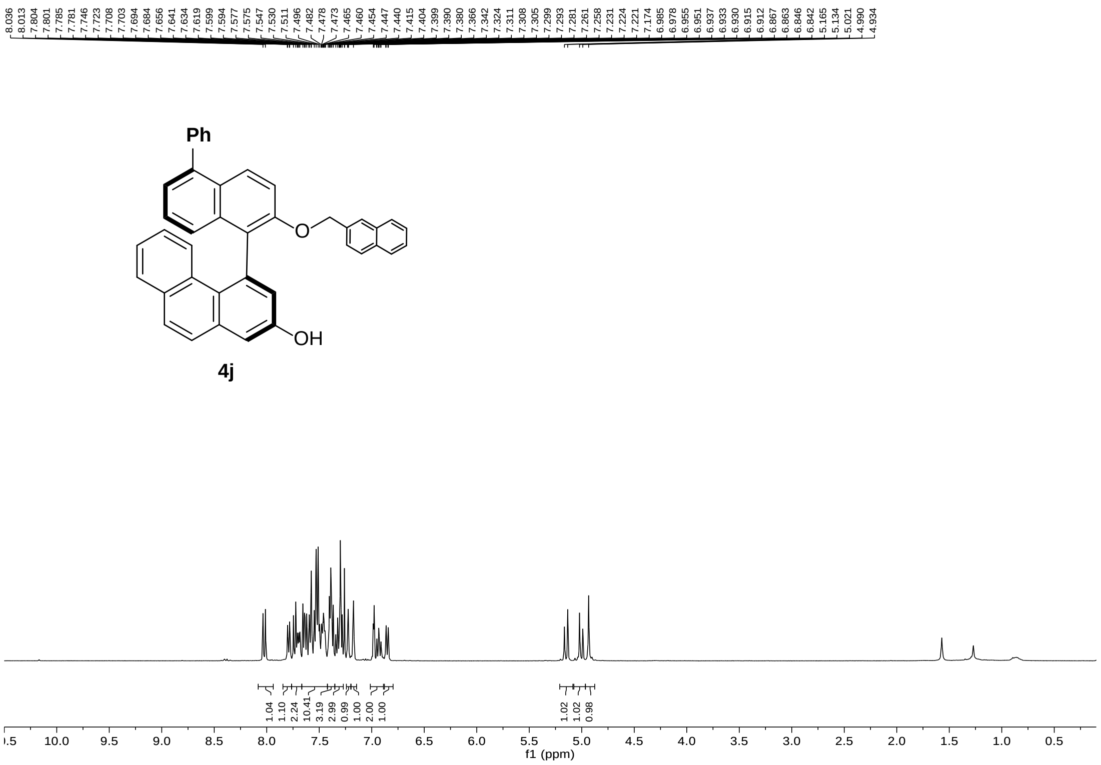

8.036 8.013 7.804 7.801 7.785 7.781 7.746 7.723 7.708 7.703 7.694 7.684 7.656 7.641 7.634 7.619 7.599 7.594 7.577 7.575 7.547 7.530 7.511 7.496 7.482 7.478 7.473 7.465 7.460 7.454 7.447 7.440 7.415 7.404 7.399 7.390 7.380 7.366 7.342 7.324 7.311 7.308 7.305 7.299 7.293 7.281 7.261 7.258 7.231 7.224 7.221 7.174 6.985 6.978 6.955 6.951 6.937 6.933 6.930 6.915 6.912 6.867 6.863 6.846 6.842 5.165 5.134 5.021 4.990 4.934
Ph
O
OH
4j
1.04 1.10 2.24 10.41 3.19 2.99 0.99 1.00 2.00 1.00
1.02 1.02 0.98
10.0 9.5 9.0 8.5 8.0 7.5 7.0 6.5 6.0 5.5 5.0 4.5 4.0 3.5 3.0 2.5 2.0 1.5 1.0 0.5
f1 (ppm)

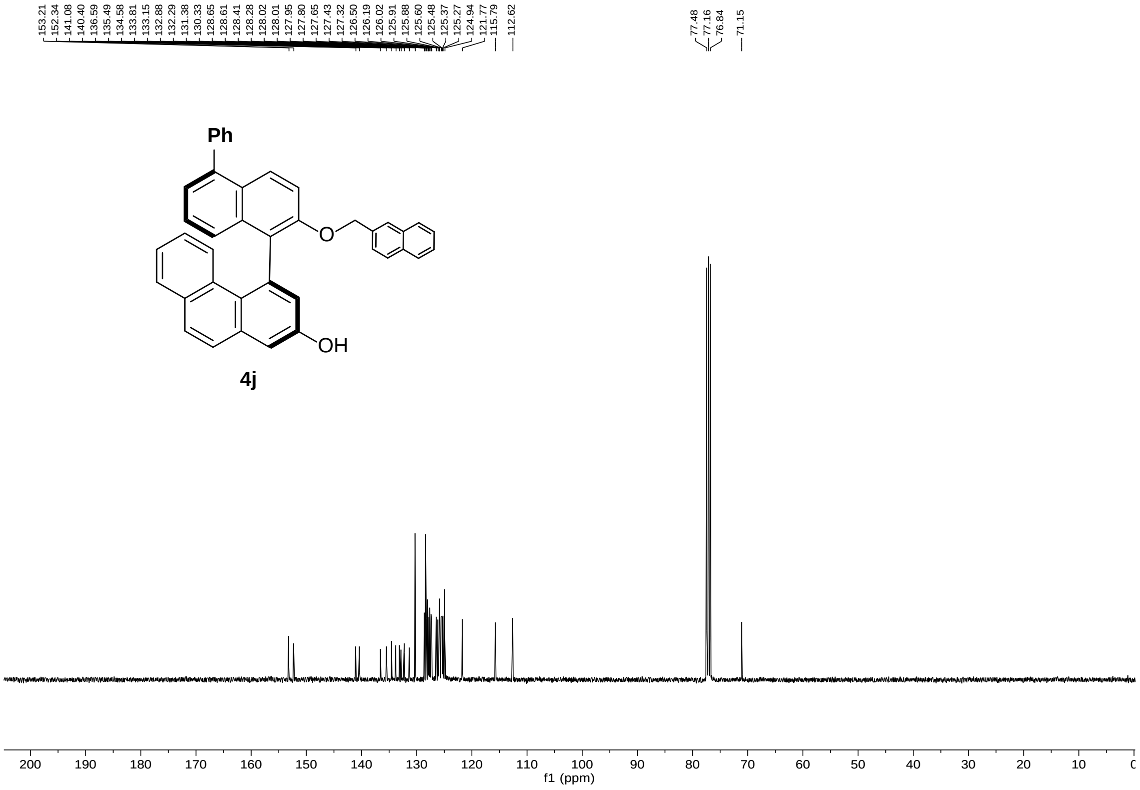

153.21 152.34 141.08 140.40 136.59 135.49 134.58 133.81 133.15 132.88 132.29 131.38 130.33 128.65 128.61 128.41 128.28 128.02 128.01 127.95 127.80 127.65 127.43 127.32 126.50 126.19 126.02 125.91 125.88 125.60 125.48 125.37 125.27 124.94 121.77 115.79 112.62
77.48 77.16 76.84 71.15
Ph
O
OH
4j
200 190 180 170 160 150 140 130 120 110 100 90 80 70 60 50 40 30 20 10
f1 (ppm)

Compound 4k

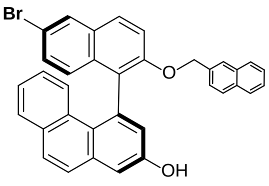

4k

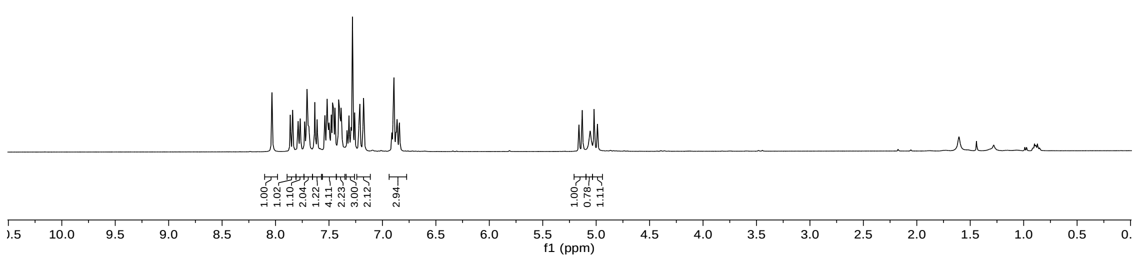

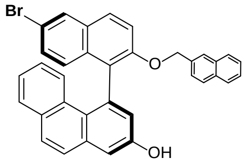

4k

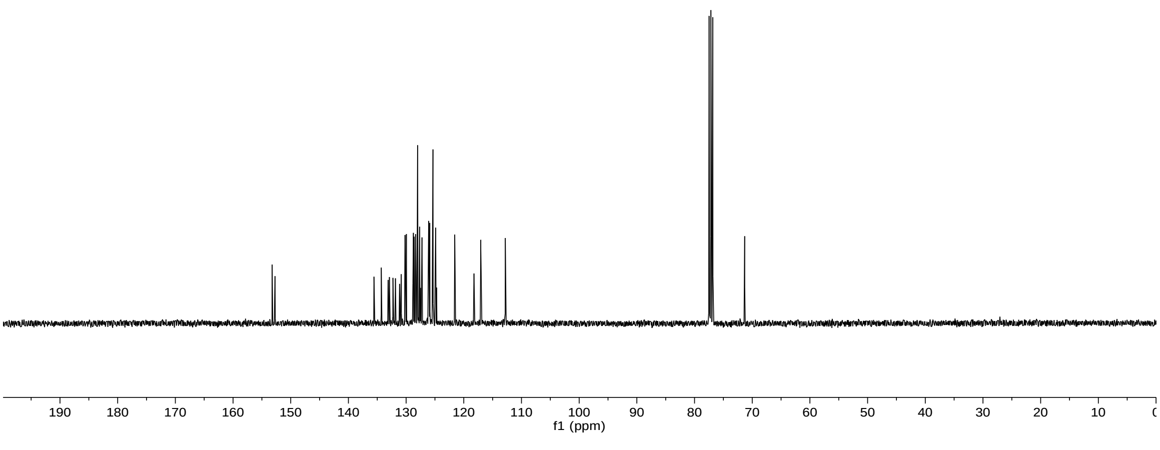

Compound 4l

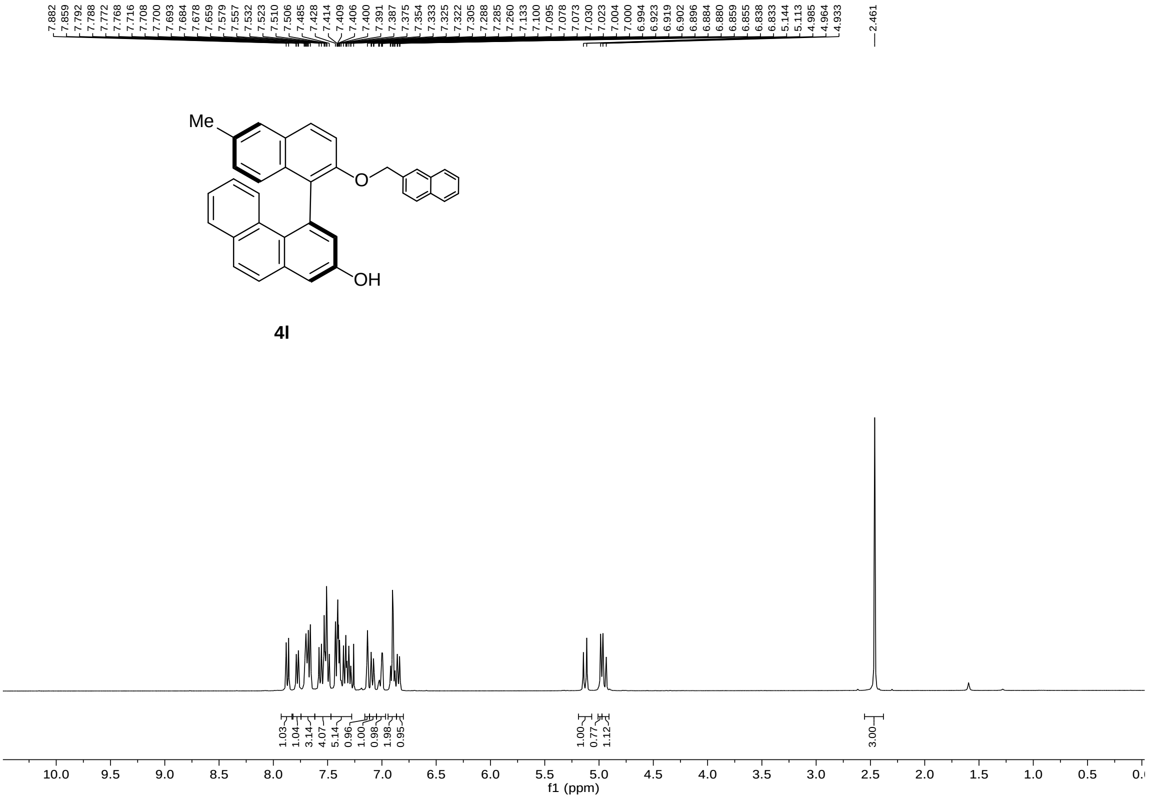

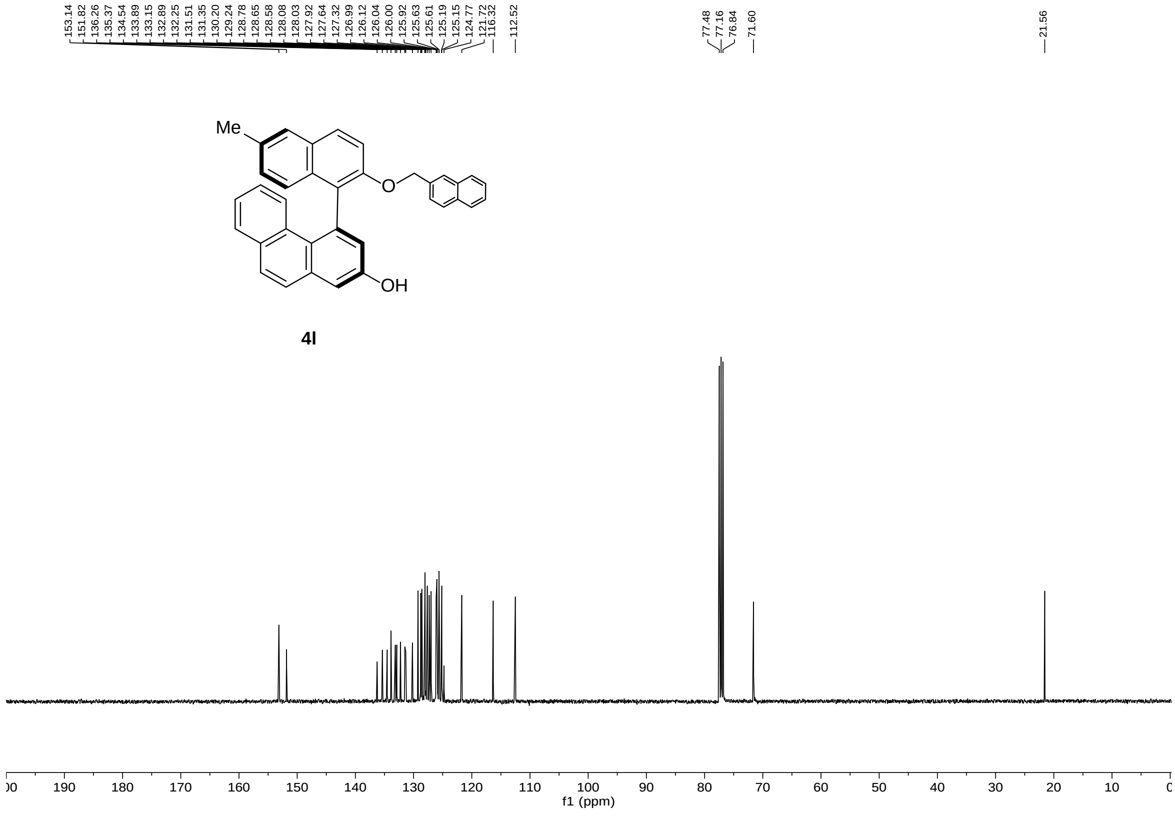

Compound 4m

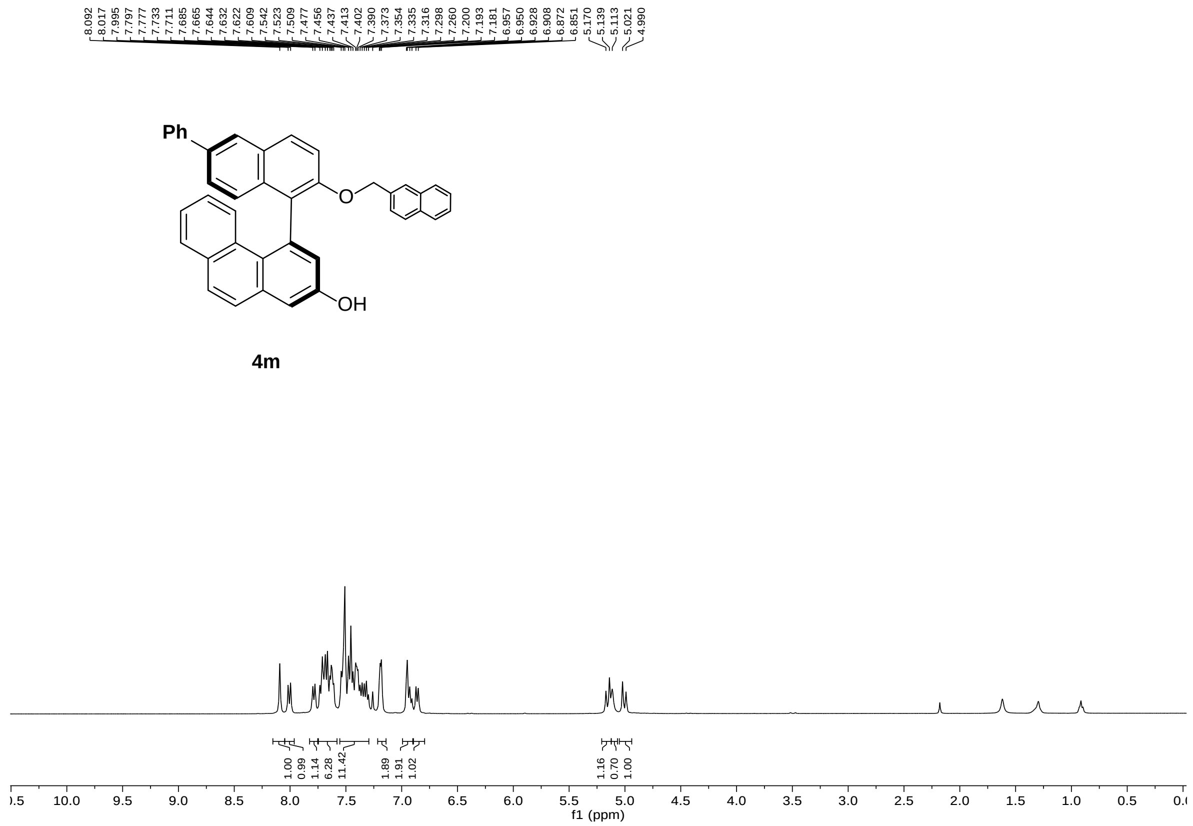

8.092 8.017 7.995 7.797 7.777 7.733 7.711 7.685 7.665 7.644 7.632 7.622 7.609 7.542 7.523 7.509 7.477 7.456 7.437 7.413 7.402 7.390 7.373 7.354 7.335 7.316 7.298 7.260 7.200 7.193 7.181 6.957 6.950 6.928 6.908 6.872 6.851 5.170 5.139 5.113 5.021 4.990
Ph
O
OH
4m
1.00 0.99 1.14 6.28 11.42 1.89 1.91 1.02 1.16 0.70 1.00
10.5 10.0 9.5 9.0 8.5 8.0 7.5 7.0 6.5 6.0 5.5 5.0 4.5 4.0 3.5 3.0 2.5 2.0 1.5 1.0 0.5 0.0
f1 (ppm)

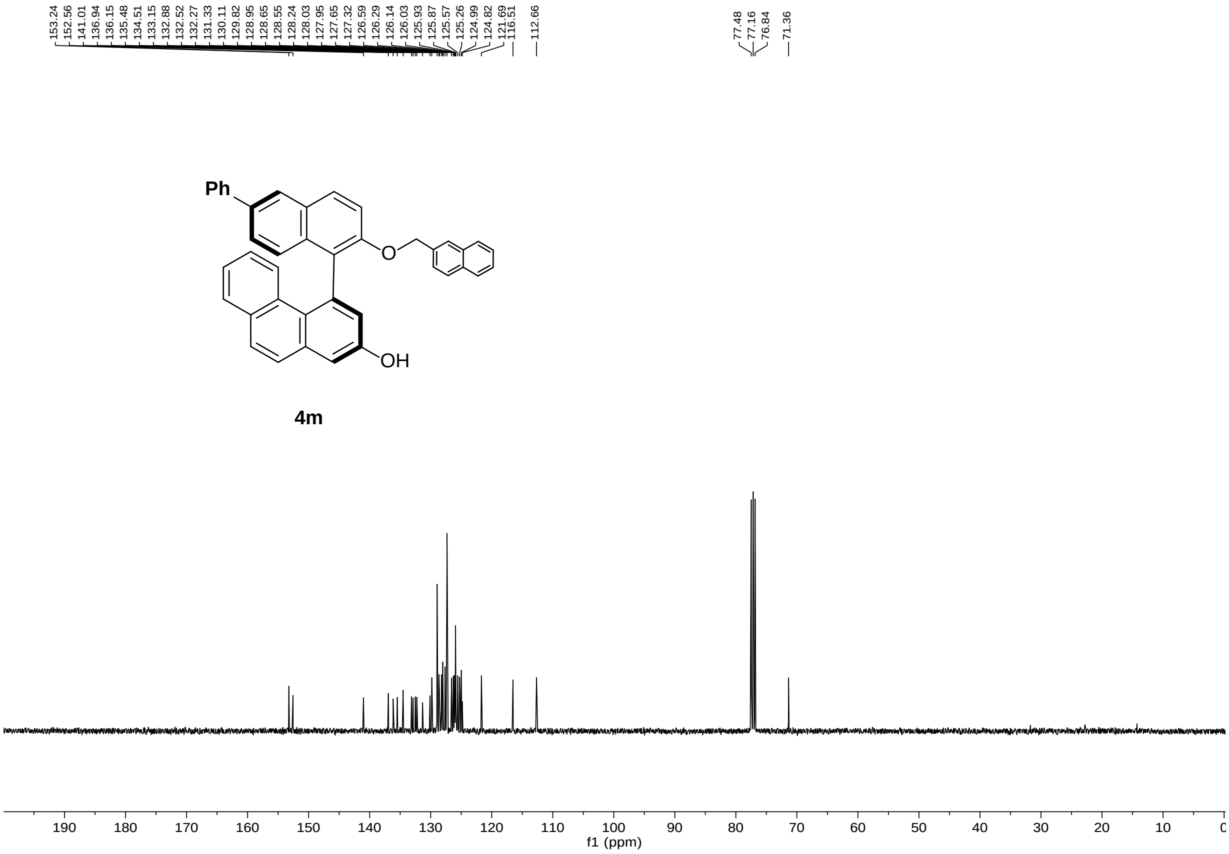

153.24 152.56 141.01 136.94 136.15 135.48 134.51 133.15 132.88 132.52 132.27 131.33 130.11 129.82 128.95 128.65 128.55 128.24 128.03 127.95 127.65 127.32 126.59 126.29 126.14 126.03 125.93 125.87 125.57 125.26 124.99 124.82 121.69 116.51 112.66
77.48 77.16 76.84 71.36
Ph
O
OH
4m
190 180 170 160 150 140 130 120 110 100 90 80 70 60 50 40 30 20 10 0
f1 (ppm)

Compound 4n

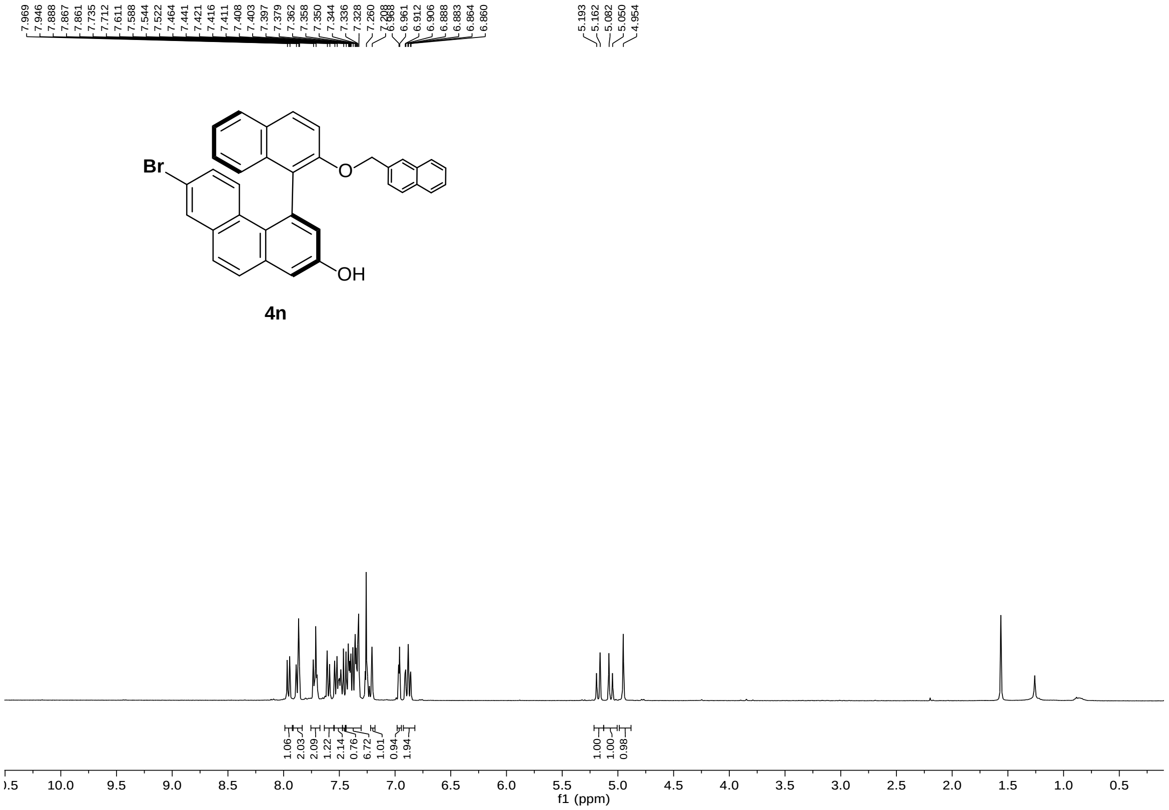

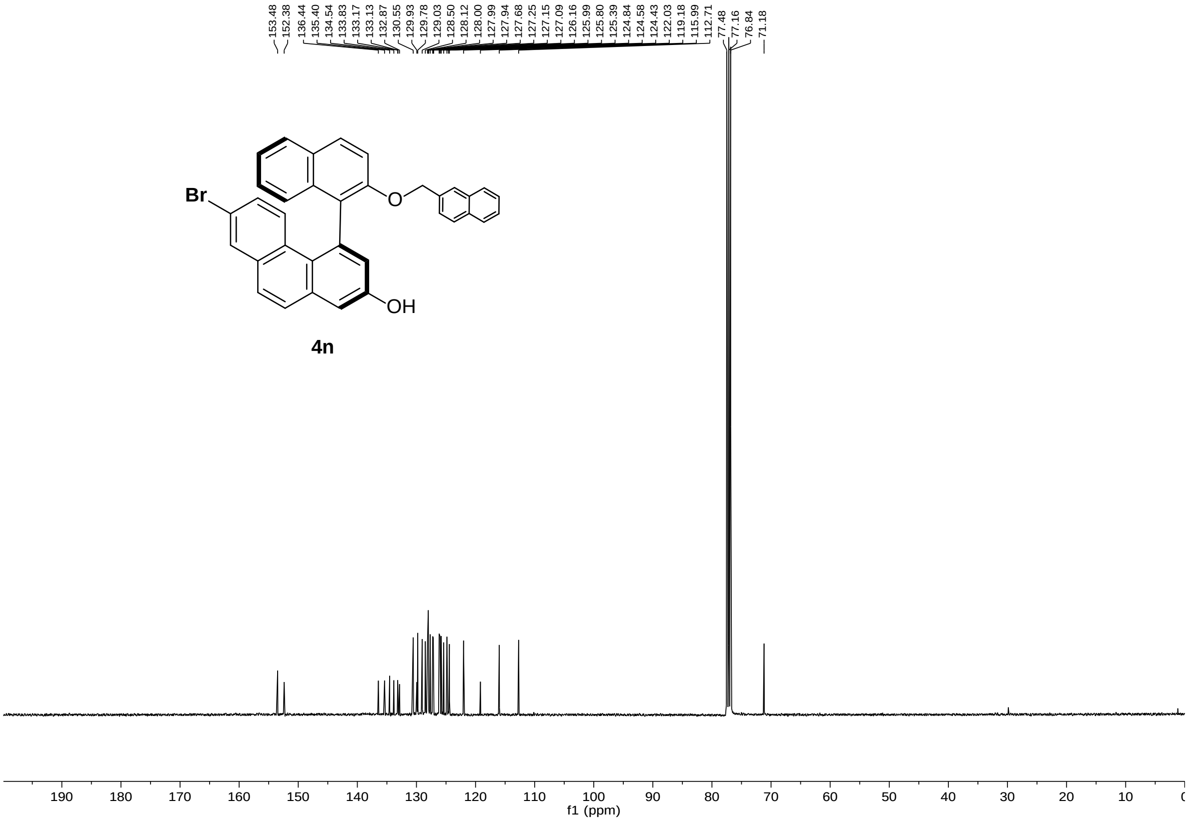

Compound 4o

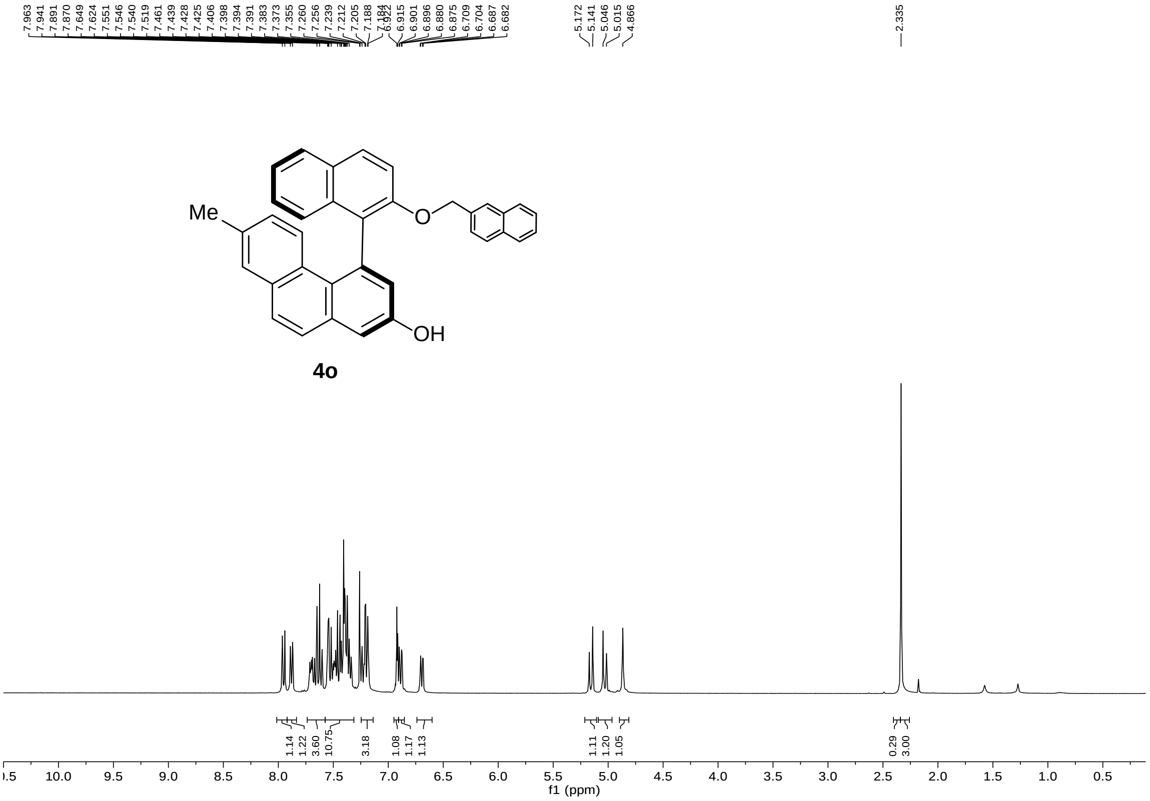

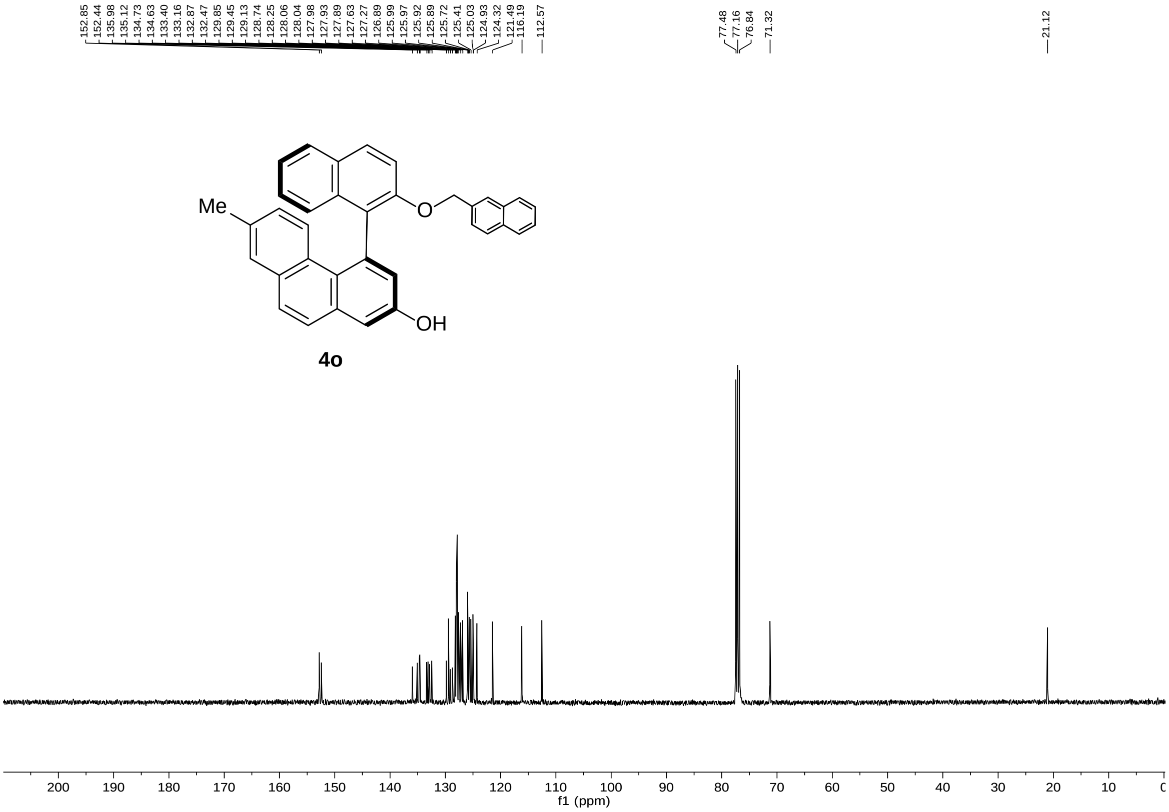

Compound 4p

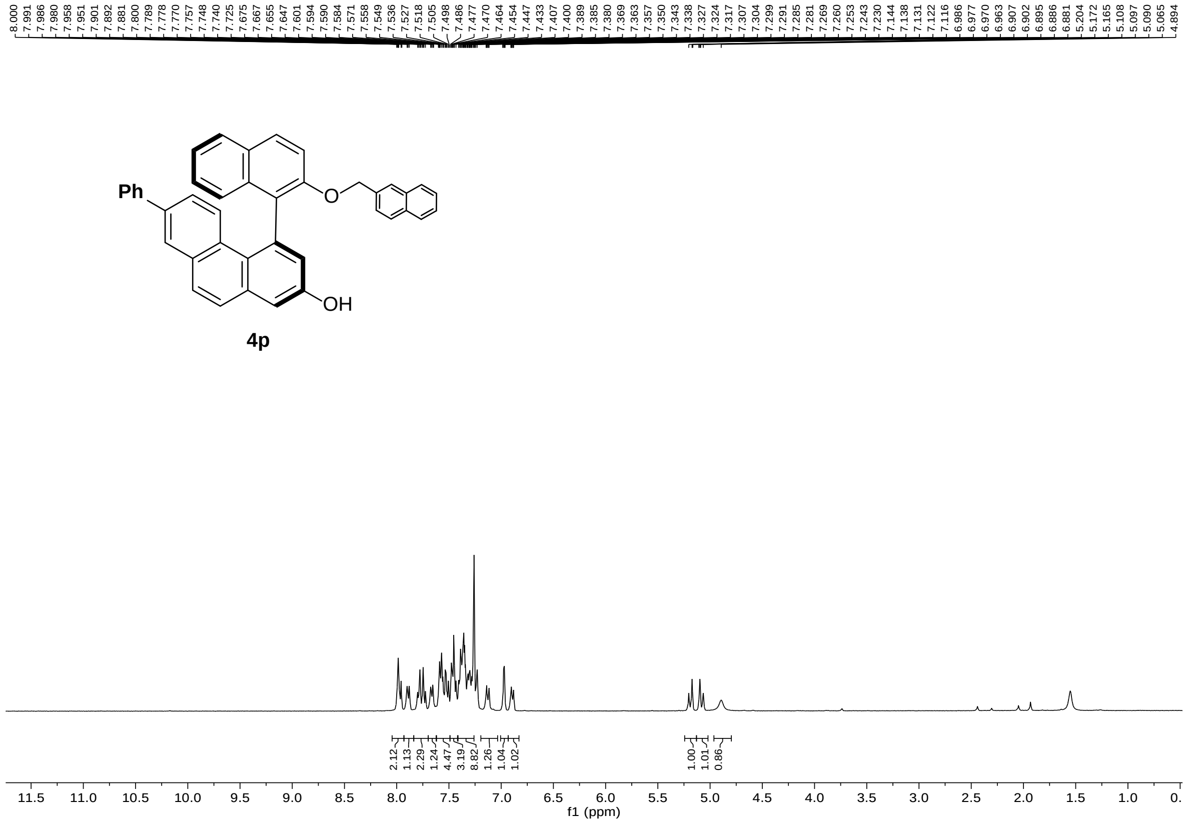
8.000
7.991
7.986
7.980
7.958
7.951
7.901
7.892
7.881
7.800
7.789
7.778
7.770
7.757
7.748
7.740
7.725
7.675
7.667
7.655
7.647
7.601
7.594
7.590
7.584
7.571
7.558
7.549
7.536
7.527
7.518
7.505
7.498
7.486
7.477
7.470
7.464
7.454
7.447
7.433
7.407
7.400
7.389
7.385
7.380
7.369
7.363
7.357
7.350
7.343
7.338
7.327
7.324
7.317
7.307
7.304
7.299
7.291
7.285
7.281
7.269
7.260
7.253
7.243
7.230
7.144
7.138
7.131
7.122
7.116
6.986
6.977
6.970
6.963
6.907
6.902
6.895
6.886
6.881
5.204
5.172
5.165
5.108
5.097
5.090
5.065
4.894
Ph
O
OH
4p
2.12
1.13
2.29
1.24
4.47
3.19
8.82
1.26
1.04
1.02
1.00
1.01
0.86
11.5 11.0 10.5 10.0 9.5 9.0 8.5 8.0 7.5 7.0 6.5 6.0 5.5 5.0 4.5 4.0 3.5 3.0 2.5 2.0 1.5 1.0
f1 (ppm)

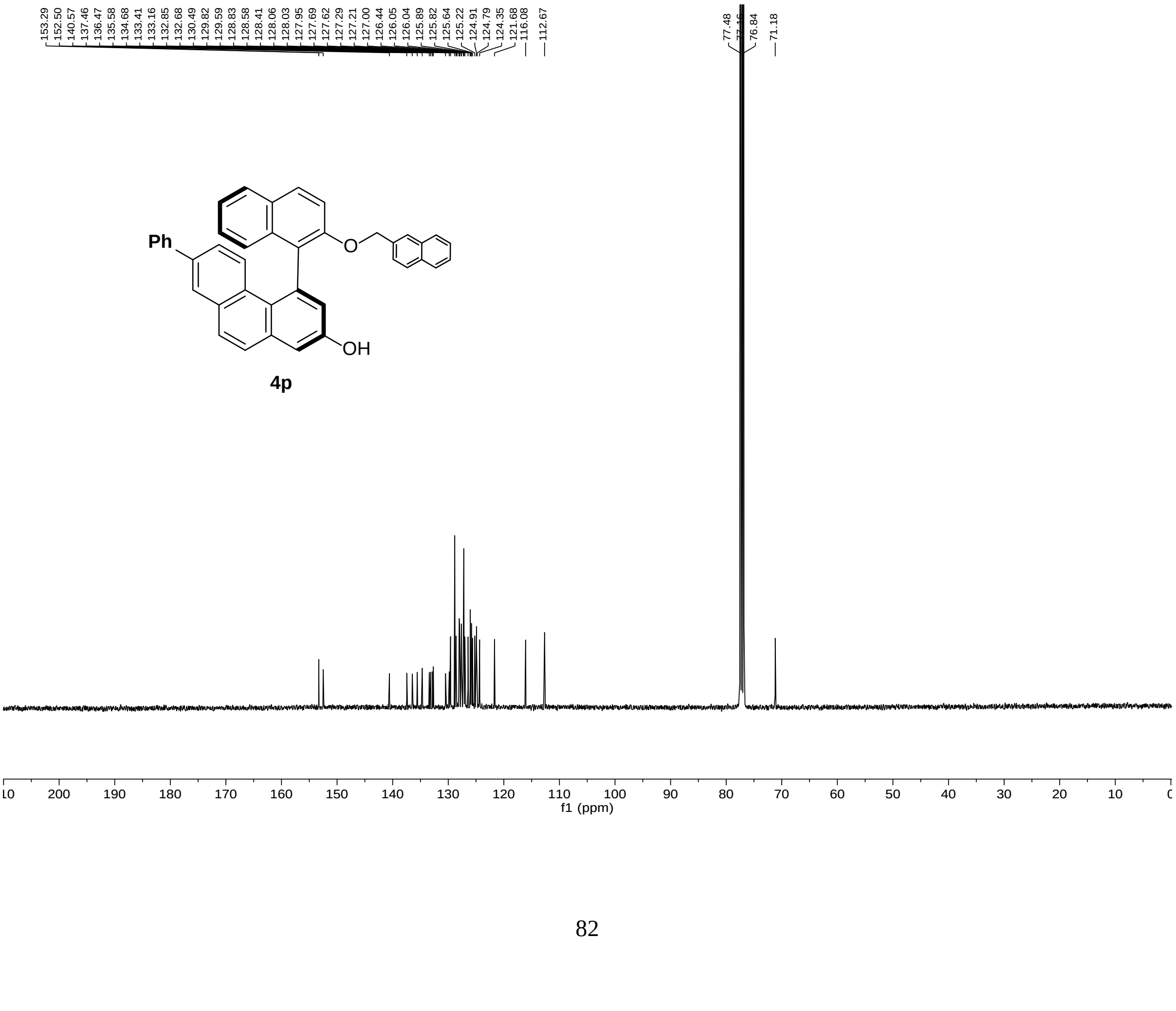
153.29
152.50
140.57
137.46
136.47
135.58
134.68
133.41
133.16
132.85
132.68
130.49
129.82
129.59
128.83
128.58
128.41
128.06
128.03
127.95
127.69
127.62
127.29
127.21
127.00
126.44
126.05
126.04
125.89
125.82
125.64
125.22
124.91
124.79
124.35
121.68
116.08
112.67
77.48
77.16
76.84
71.18
Ph
O
OH
4p
200 190 180 170 160 150 140 130 120 110 100 90 80 70 60 50 40 30 20 10
f1 (ppm)

Compound 4q

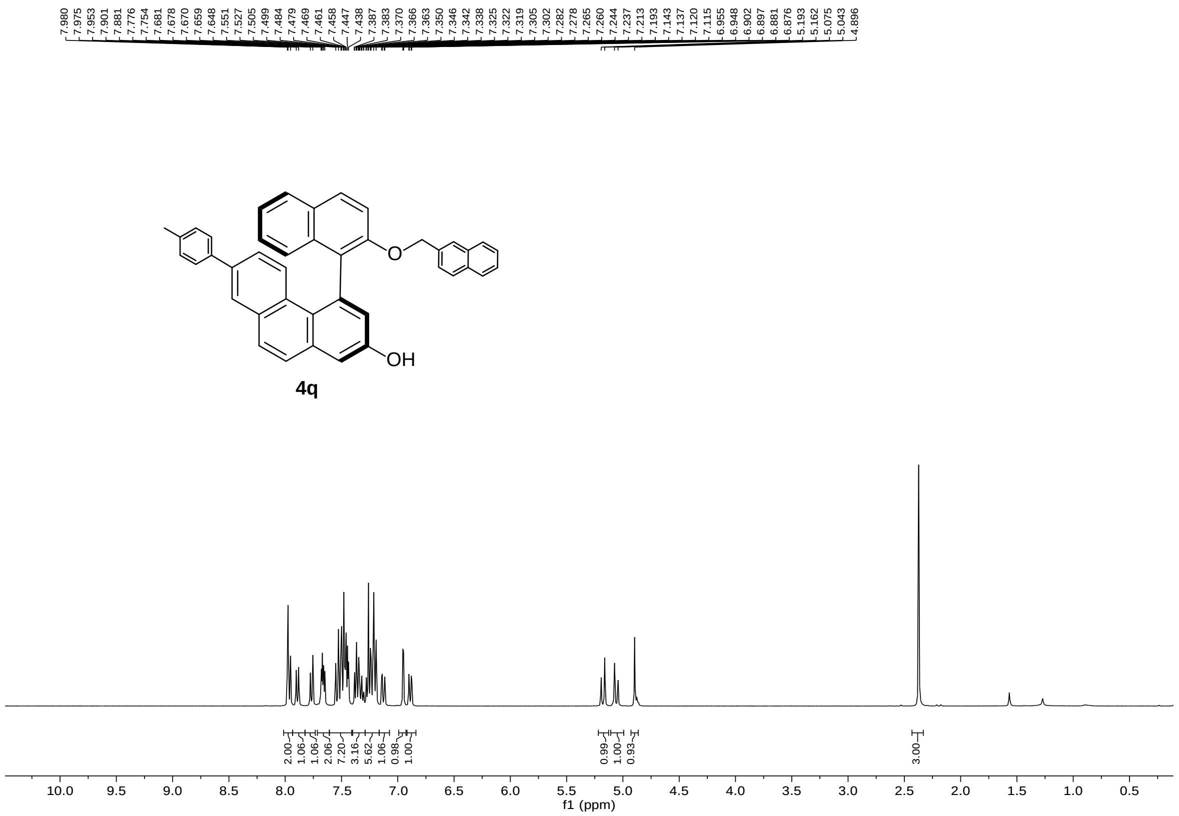

7.980
7.975
7.953
7.901
7.881
7.776
7.754
7.681
7.678
7.670
7.659
7.648
7.551
7.527
7.505
7.499
7.484
7.479
7.469
7.461
7.458
7.447
7.438
7.387
7.383
7.370
7.366
7.363
7.350
7.346
7.342
7.338
7.325
7.322
7.319
7.305
7.302
7.282
7.278
7.265
7.260
7.244
7.237
7.213
7.193
7.143
7.137
7.120
7.115
6.955
6.948
6.902
6.897
6.881
6.876
5.193
5.162
5.075
5.043
4.896
O
OH
4q
2.00
1.06
1.06
2.06
7.20
3.16
5.62
1.06
0.98
1.00
0.99
1.00
0.93
3.00
10.0 9.5 9.0 8.5 8.0 7.5 7.0 6.5 6.0 5.5 5.0 4.5 4.0 3.5 3.0 2.5 2.0 1.5 1.0 0.5
f1 (ppm)

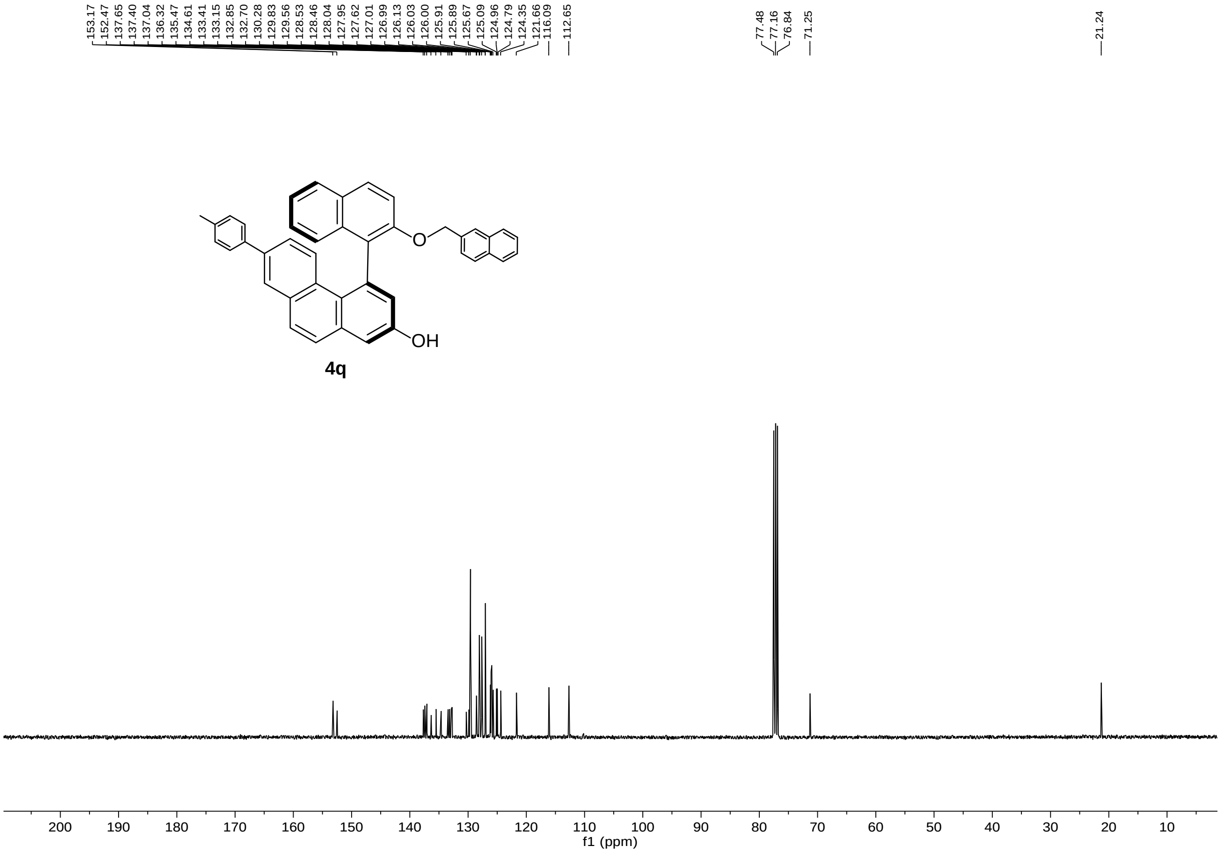

153.17
152.47
137.65
137.40
137.04
136.32
135.47
134.61
133.41
133.15
132.85
132.70
130.28
129.83
129.56
128.53
128.46
128.04
127.95
127.62
127.01
126.99
126.13
126.03
126.00
125.91
125.89
125.67
125.09
124.96
124.79
124.35
121.66
116.09
112.65
77.48
77.16
76.84
71.25
21.24
O
OH
4q
200 190 180 170 160 150 140 130 120 110 100 90 80 70 60 50 40 30 20 10
f1 (ppm)

Compound 4r

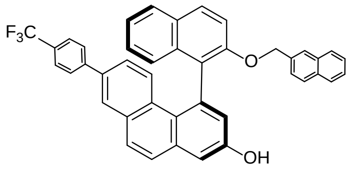

4r

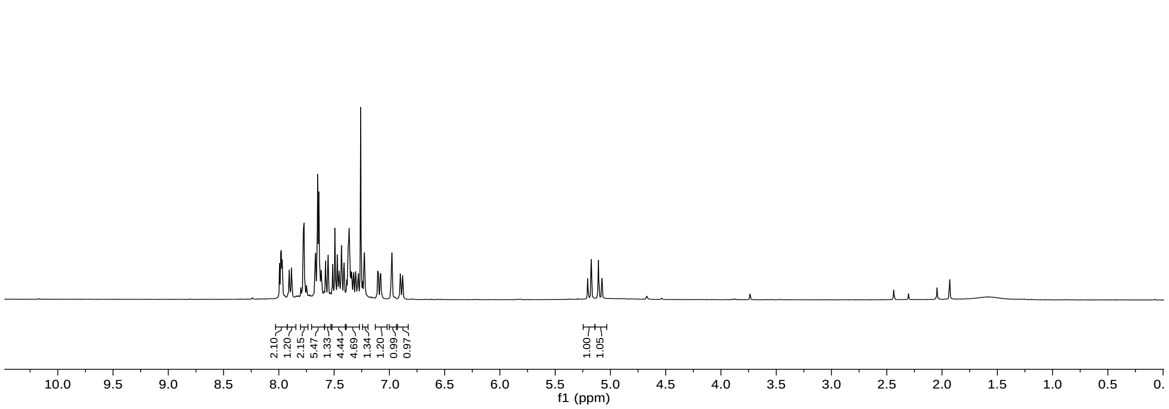

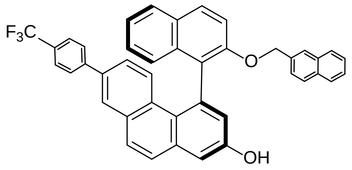

4r

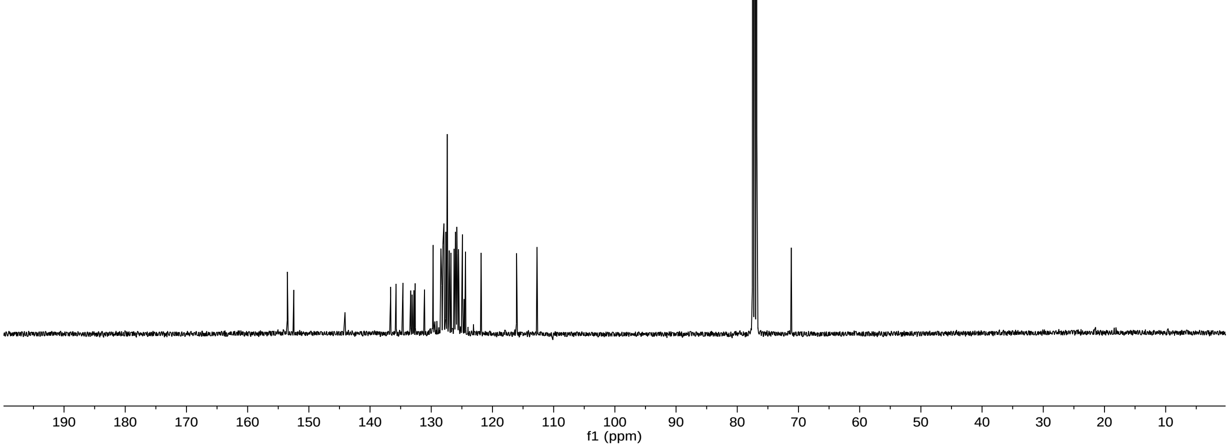

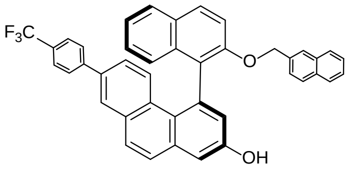
F3C
O
OH

4r

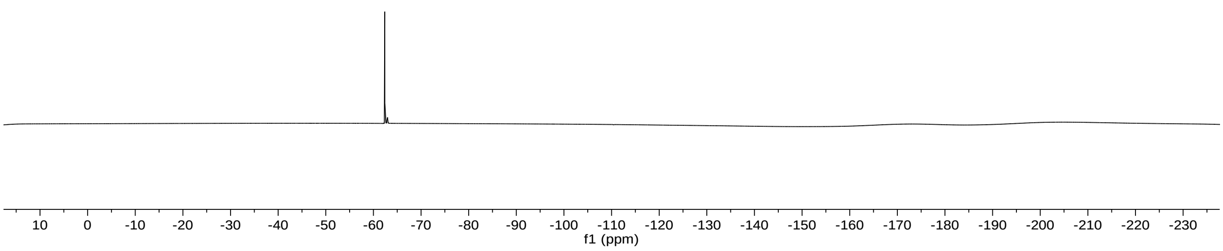
-62.36
10
0
-10
-20
-30
-40
-50
-60
-70
-80
-90
-100
-110
-120
-130
-140
-150
-160
-170
-180
-190
-200
-210
-220
-230
f1 (ppm)

Compound 4s

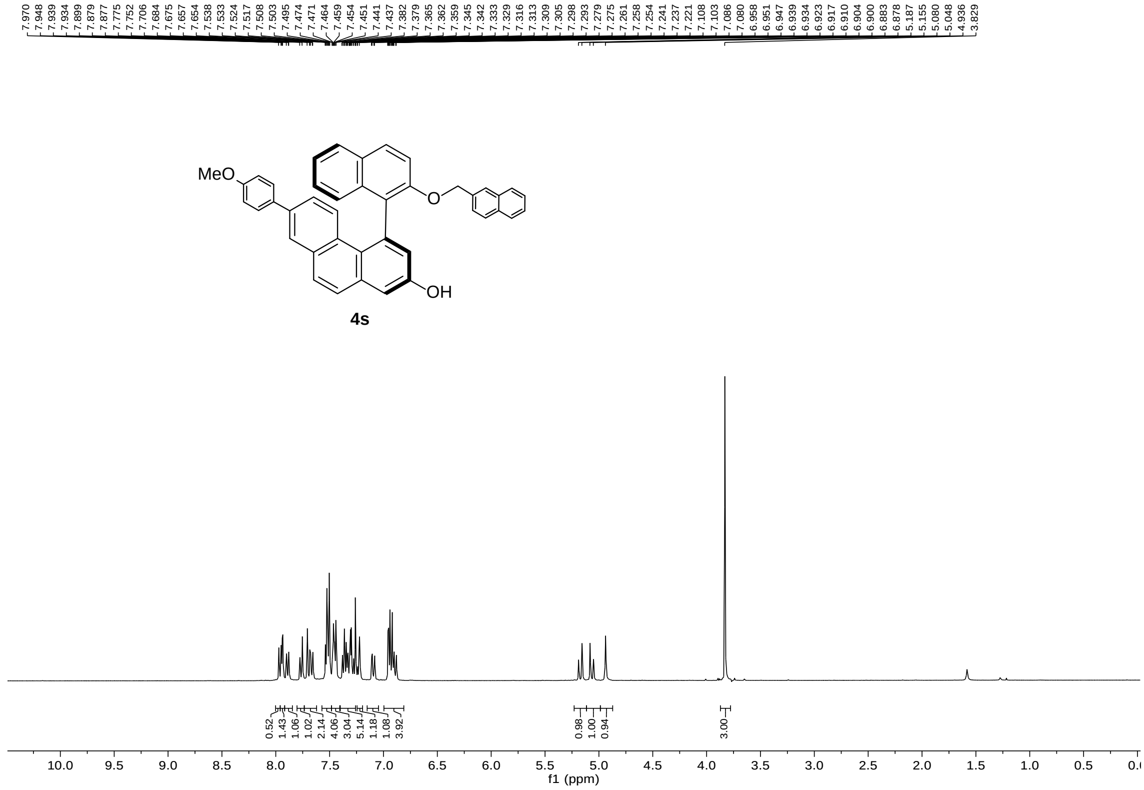

7.970 7.948 7.939 7.934 7.899 7.879 7.877 7.775 7.752 7.706 7.684 7.675 7.657 7.654 7.538 7.533 7.524 7.517 7.508 7.503 7.495 7.474 7.471 7.464 7.459 7.454 7.451 7.441 7.437 7.382 7.379 7.365 7.362 7.359 7.345 7.342 7.333 7.329 7.316 7.313 7.309 7.305 7.298 7.293 7.279 7.275 7.261 7.258 7.254 7.241 7.237 7.221 7.108 7.103 7.086 7.080 6.958 6.951 6.947 6.939 6.934 6.923 6.917 6.910 6.904 6.900 6.883 6.878 5.187 5.155 5.080 5.048 4.936 3.829
MeO
O
OH
4s
0.52 1.43 1.06 1.02 2.14 4.06 3.04 5.14 1.18 1.08 3.92
0.98 1.00 0.94
3.00
f1 (ppm)

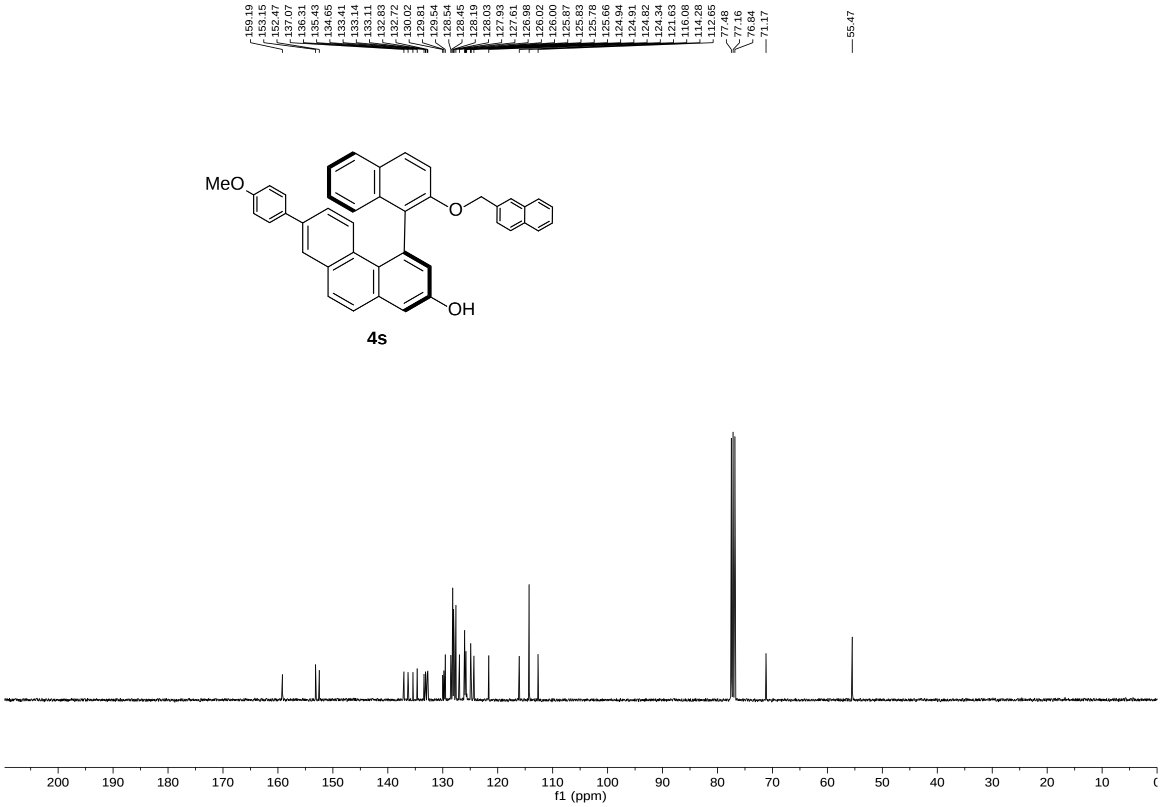

159.19 153.15 152.47 137.07 136.31 135.43 134.65 133.41 133.14 133.11 132.83 132.72 130.02 129.81 129.54 128.54 128.45 128.19 128.03 127.93 127.61 126.98 126.02 126.00 125.87 125.83 125.78 125.66 124.94 124.91 124.82 124.34 121.63 116.08 114.28 112.65 77.48 77.16 76.84 71.17
55.47
MeO
O
OH
4s
f1 (ppm)

Compound 4t

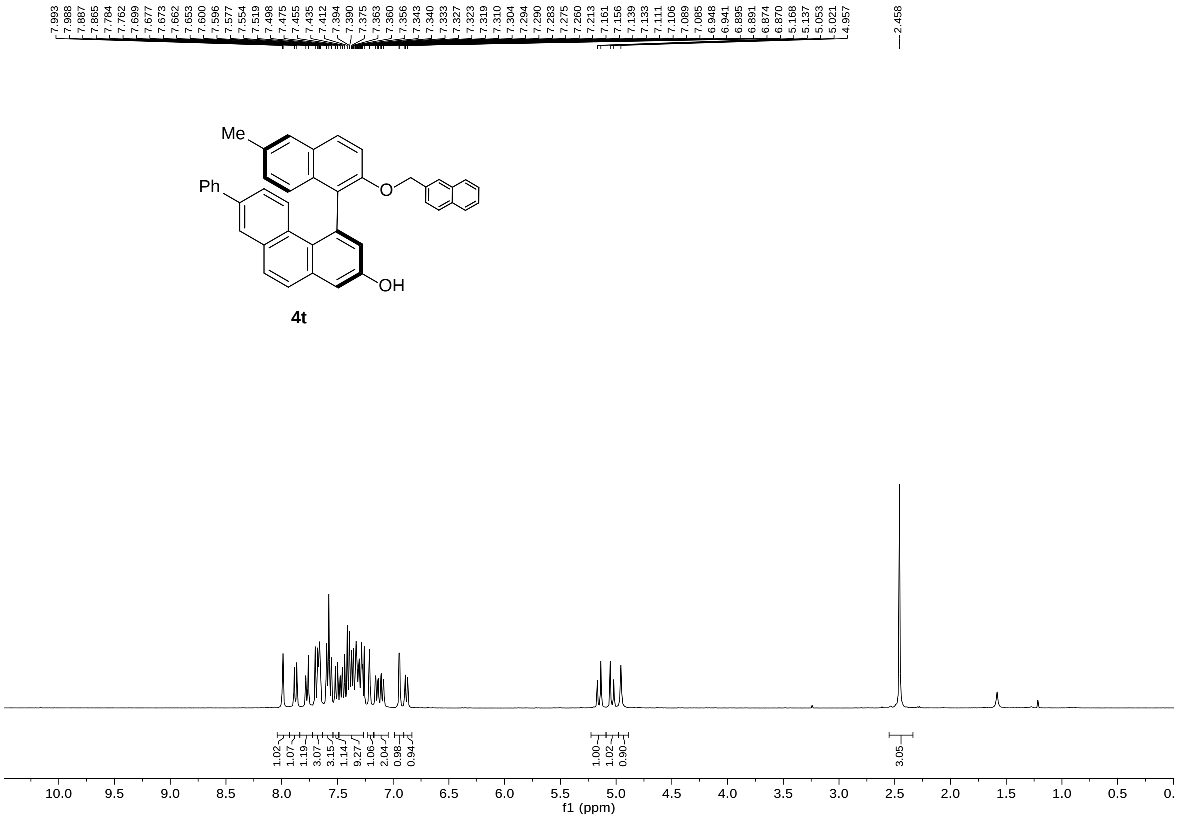

7.993
7.988
7.887
7.865
7.784
7.762
7.699
7.677
7.673
7.662
7.653
7.600
7.596
7.577
7.554
7.519
7.498
7.475
7.455
7.435
7.412
7.394
7.390
7.375
7.363
7.360
7.356
7.343
7.340
7.333
7.327
7.323
7.319
7.310
7.304
7.294
7.290
7.283
7.275
7.260
7.213
7.161
7.156
7.139
7.133
7.111
7.106
7.089
7.085
6.948
6.941
6.895
6.891
6.874
6.870
5.168
5.137
5.053
5.021
4.957
2.458
Me
Ph
O
OH
4t
1.02
1.07
1.19
3.07
3.15
1.14
9.27
1.06
2.04
0.98
0.94
1.00
1.02
0.90
3.05
10.0
9.5
9.0
8.5
8.0
7.5
7.0
6.5
6.0
5.5
5.0
4.5
4.0
3.5
3.0
2.5
2.0
1.5
1.0
0.5
0.
f1 (ppm)

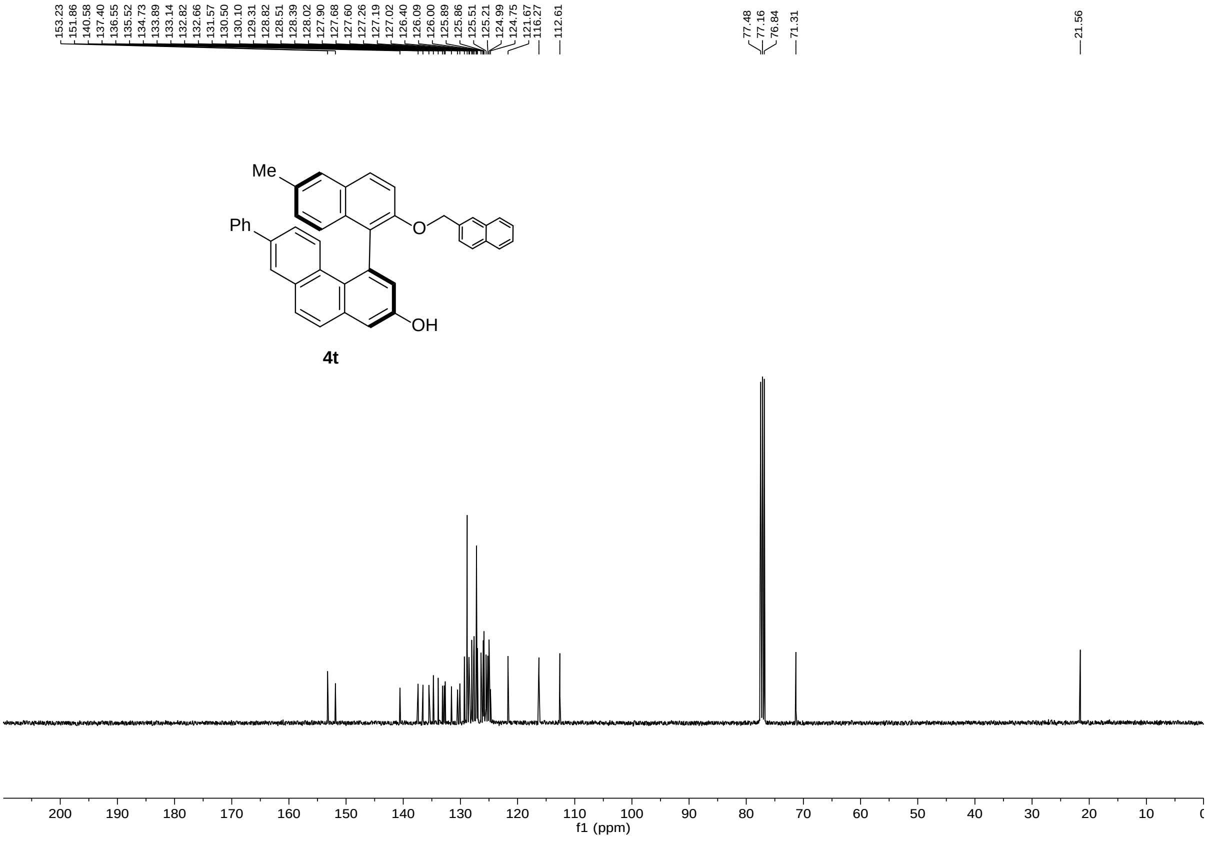

153.23
151.86
140.58
137.40
136.55
135.52
134.73
133.89
133.14
132.82
132.66
131.57
130.50
130.10
129.31
128.82
128.51
128.39
128.02
127.90
127.68
127.60
127.26
127.19
127.02
126.40
126.09
126.00
125.89
125.86
125.51
125.21
124.99
124.75
121.67
116.27
112.61
77.48
77.16
76.84
71.31
21.56
Me
Ph
O
OH
4t
200
190
180
170
160
150
140
130
120
110
100
90
80
70
60
50
40
30
20
10
f1 (ppm)

Compound 4u

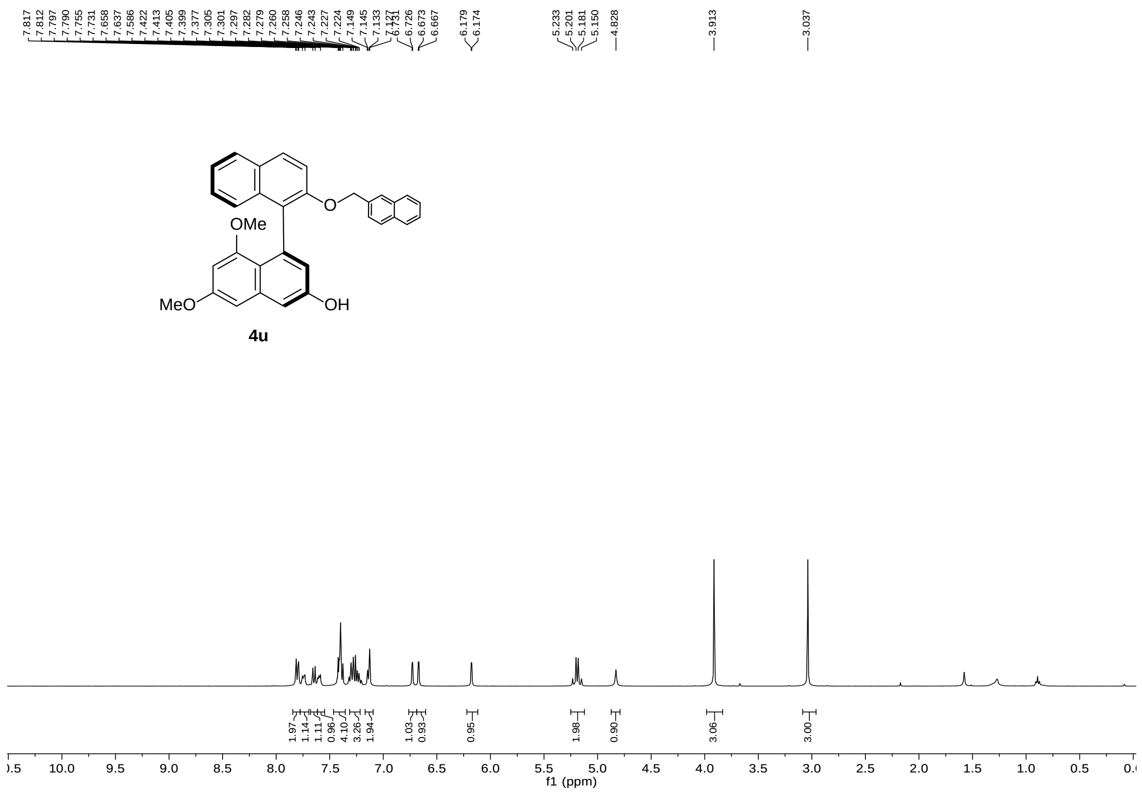

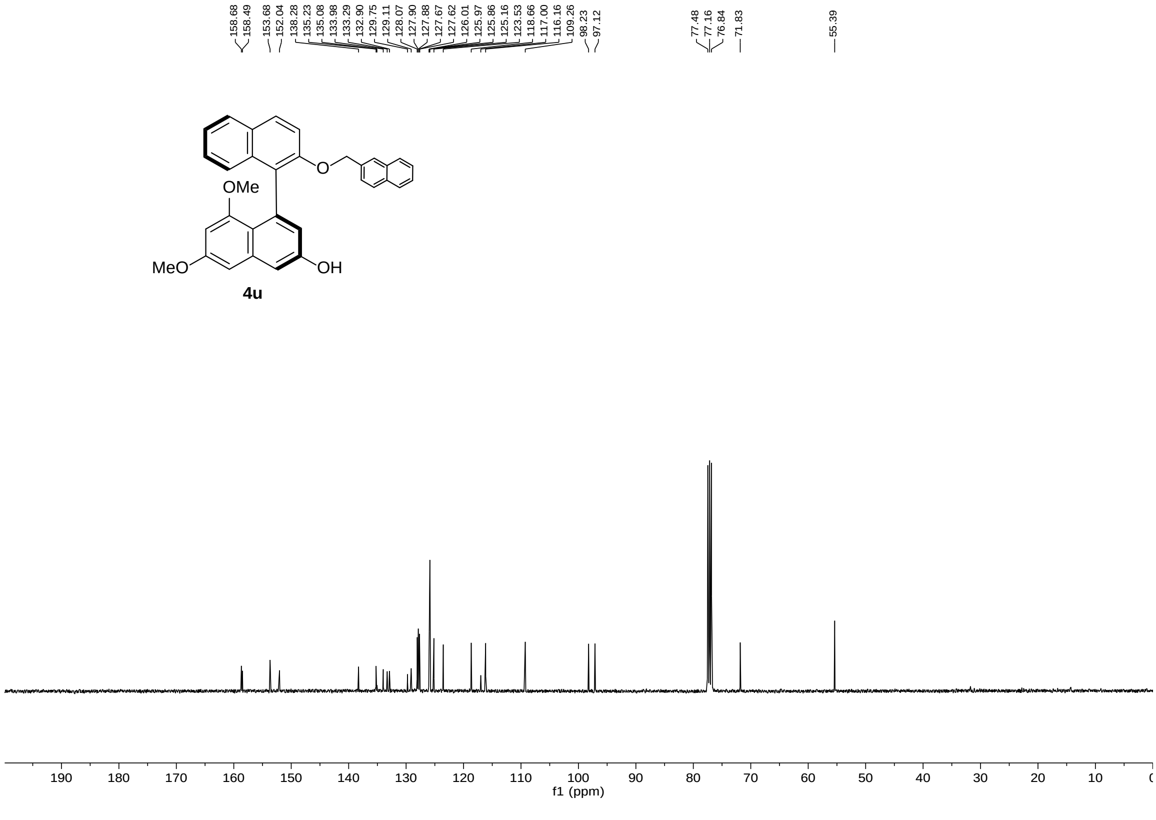

Compound 7

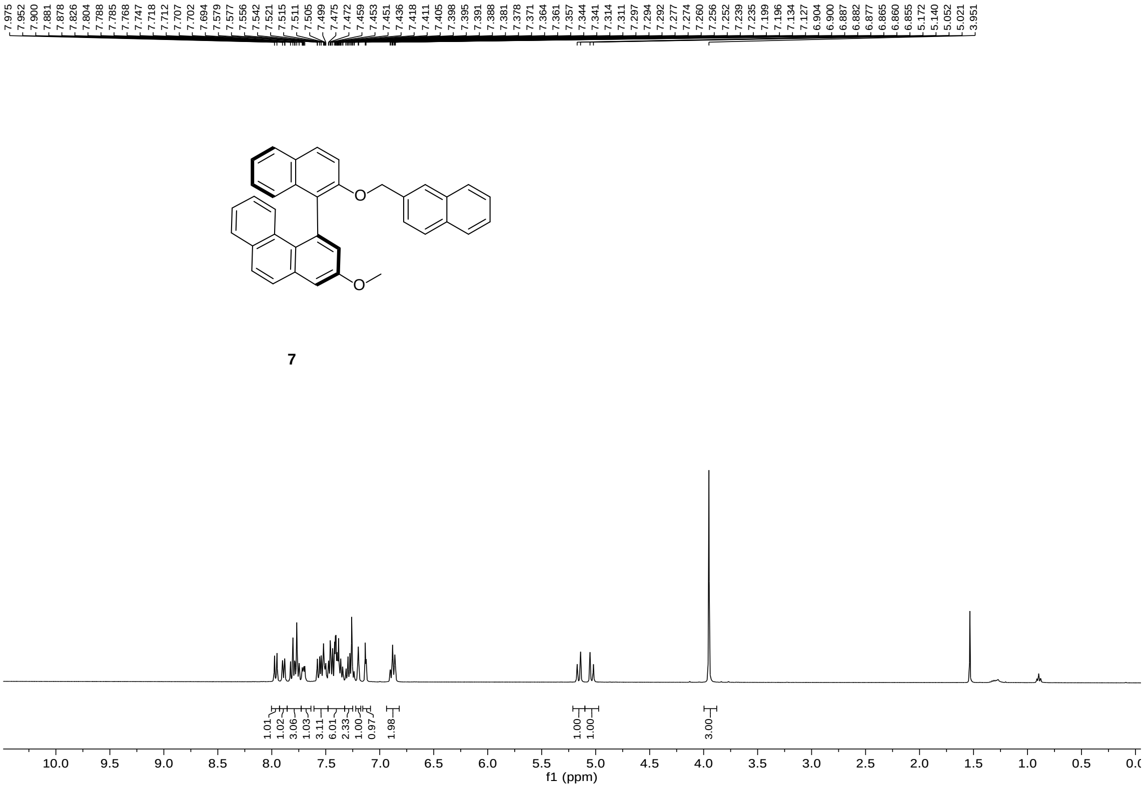
7.975 7.952 7.900 7.881 7.878 7.826 7.804 7.788 7.785 7.768 7.747 7.718 7.712 7.707 7.702 7.694 7.579 7.577 7.556 7.542 7.521 7.515 7.511 7.505 7.499 7.475 7.472 7.459 7.453 7.451 7.436 7.418 7.411 7.405 7.398 7.395 7.391 7.388 7.381 7.378 7.371 7.364 7.361 7.357 7.344 7.341 7.314 7.311 7.297 7.294 7.292 7.277 7.274 7.260 7.256 7.252 7.239 7.235 7.199 7.196 7.134 7.127 6.904 6.900 6.887 6.882 6.877 6.865 6.860 6.855 5.172 5.140 5.052 5.021 3.951
7
1.01 1.02 3.06 1.03 3.11 6.01 2.33 1.00 0.97 1.98 1.00 1.00 3.00
f1 (ppm)

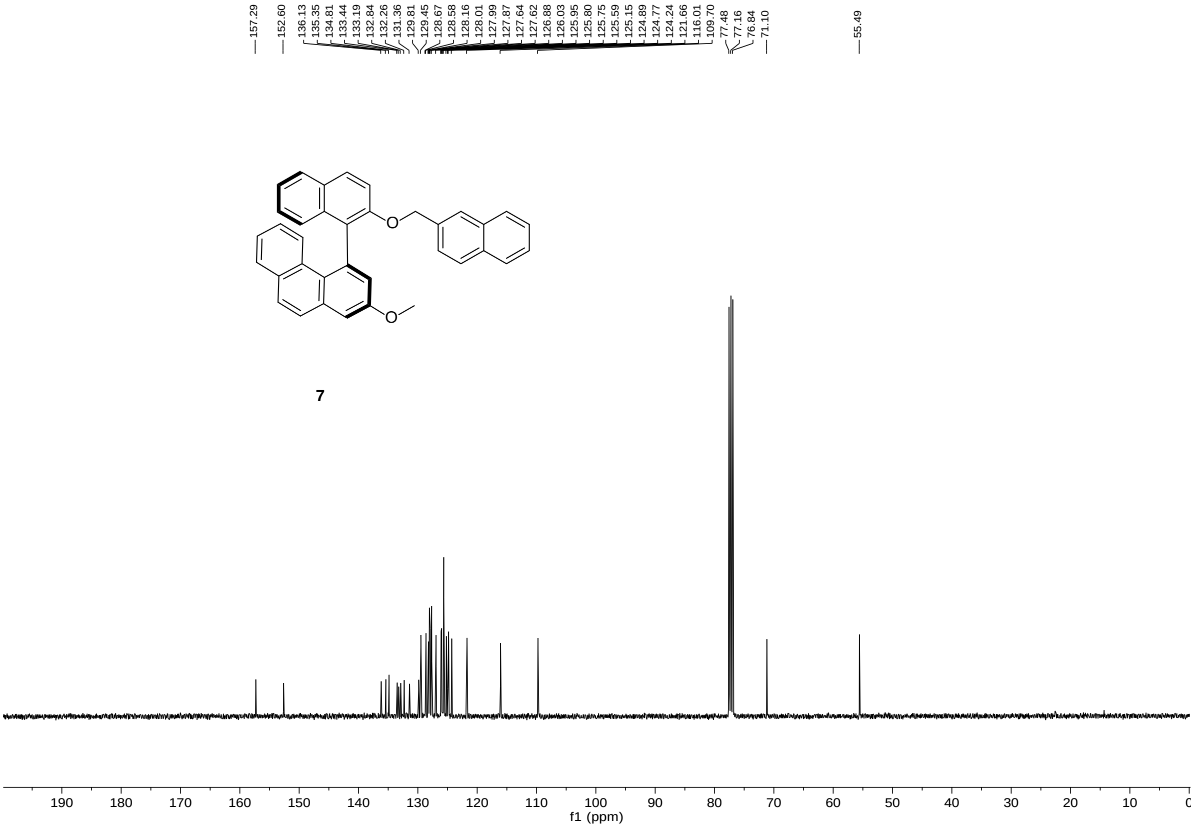
157.29 152.60 136.13 135.35 134.81 133.44 133.19 132.84 132.26 131.36 129.81 129.45 128.67 128.58 128.16 128.01 127.99 127.87 127.64 127.62 126.88 126.03 125.95 125.80 125.75 125.59 125.15 124.89 124.77 124.24 121.66 116.01 109.70 77.48 77.16 76.84 71.10 55.49
7
f1 (ppm)

Compound 8

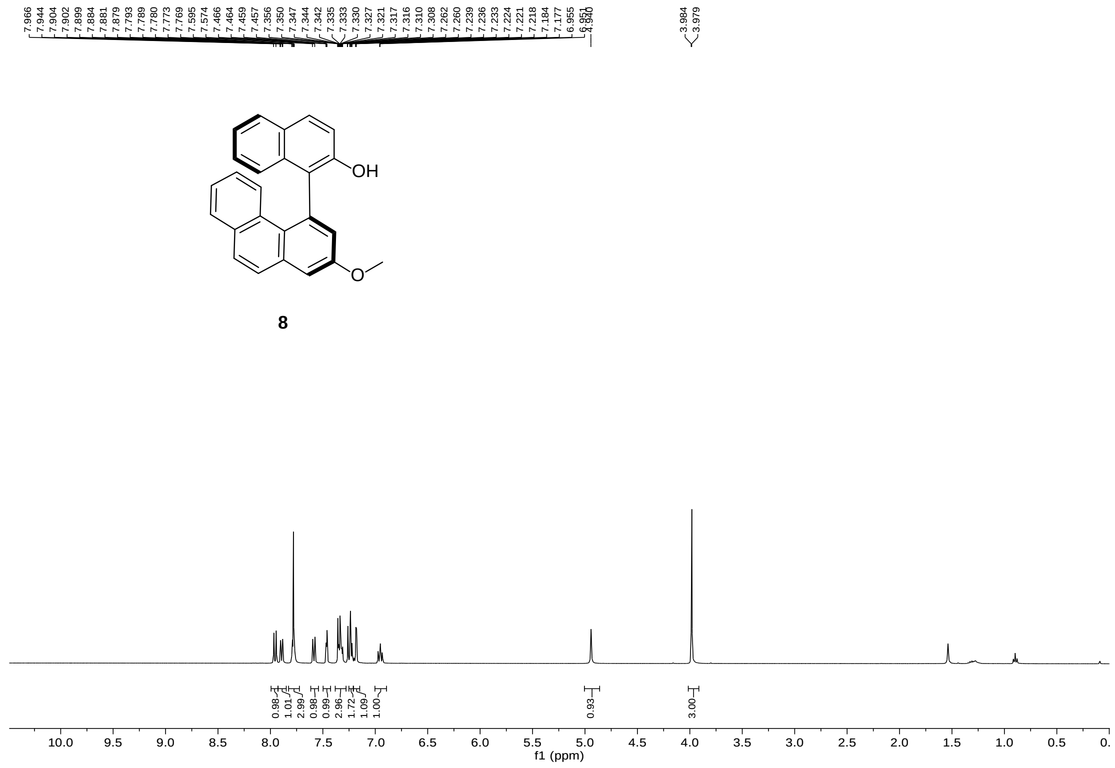

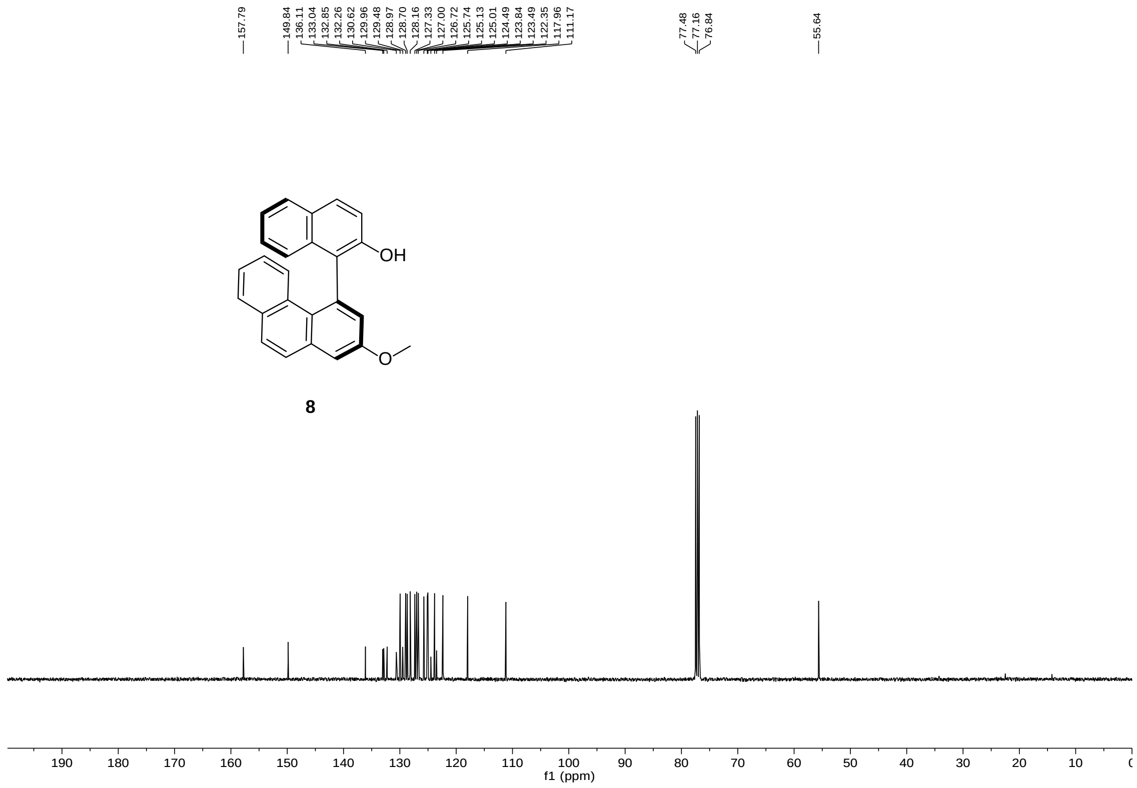

Compound 9

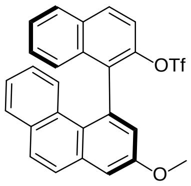

9

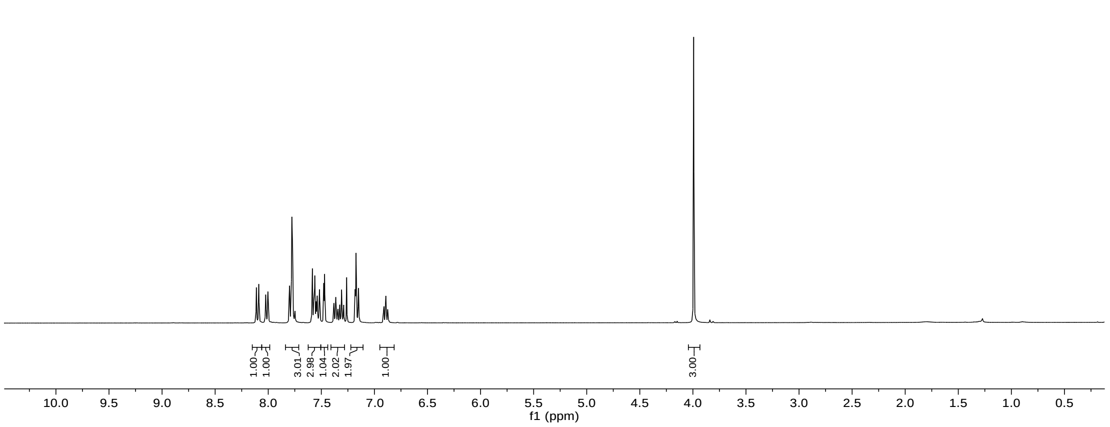

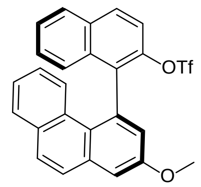

9

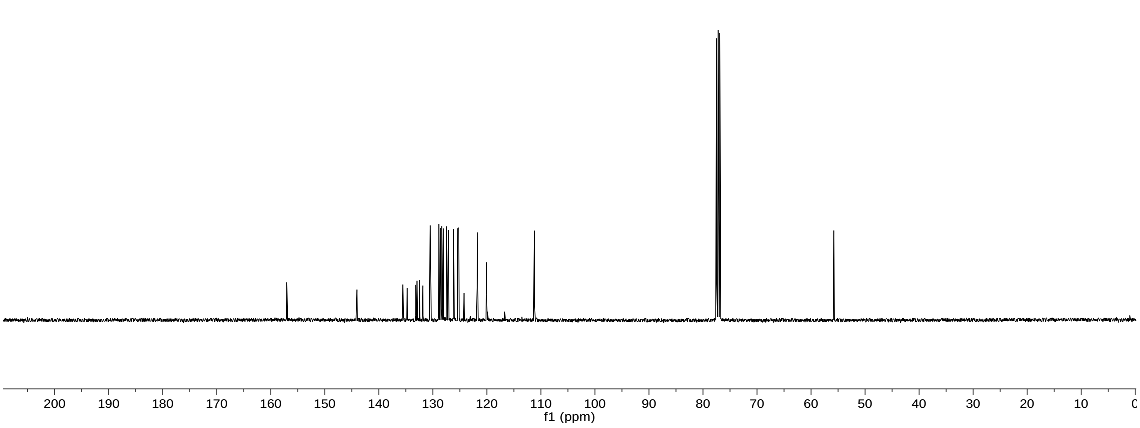

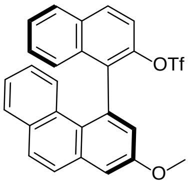
OTf
O

9

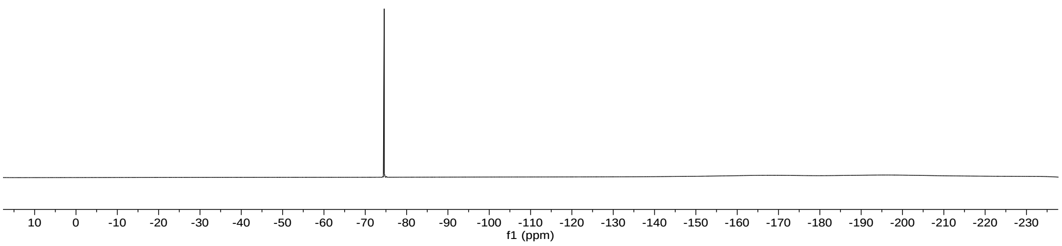
-74.55
10 0 -10 -20 -30 -40 -50 -60 -70 -80 -90 -100 -110 -120 -130 -140 -150 -160 -170 -180 -190 -200 -210 -220 -230
f1 (ppm)

Compound 10

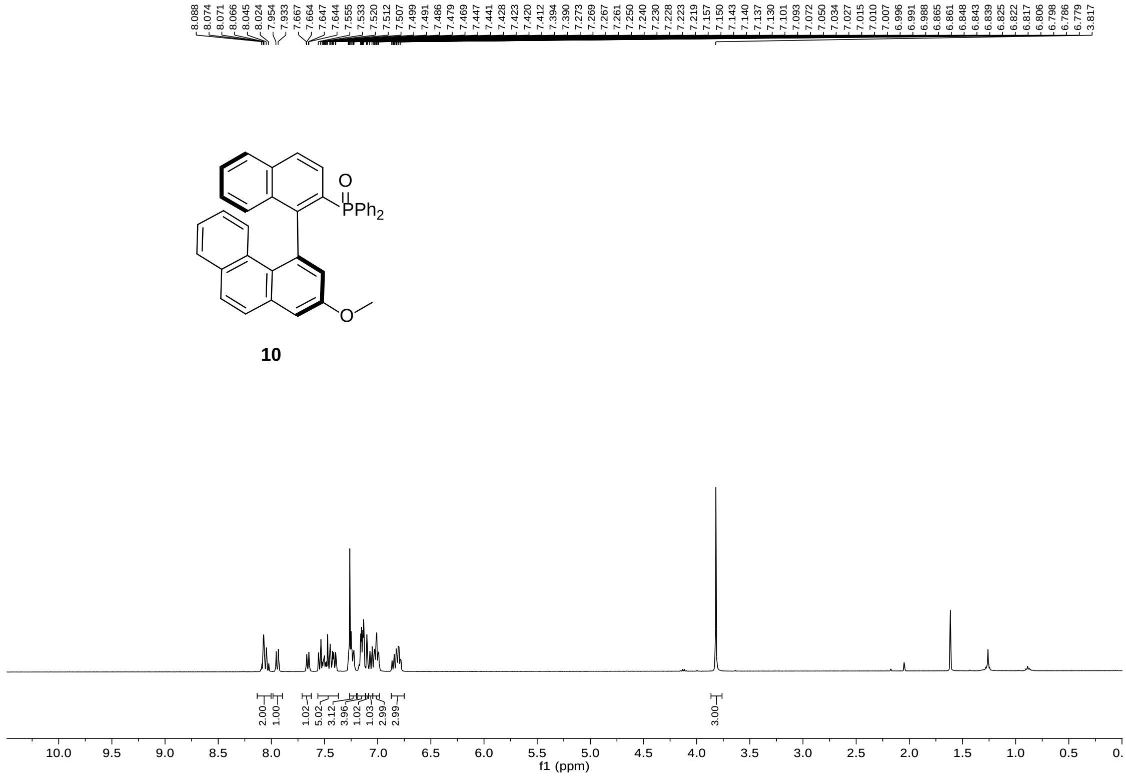

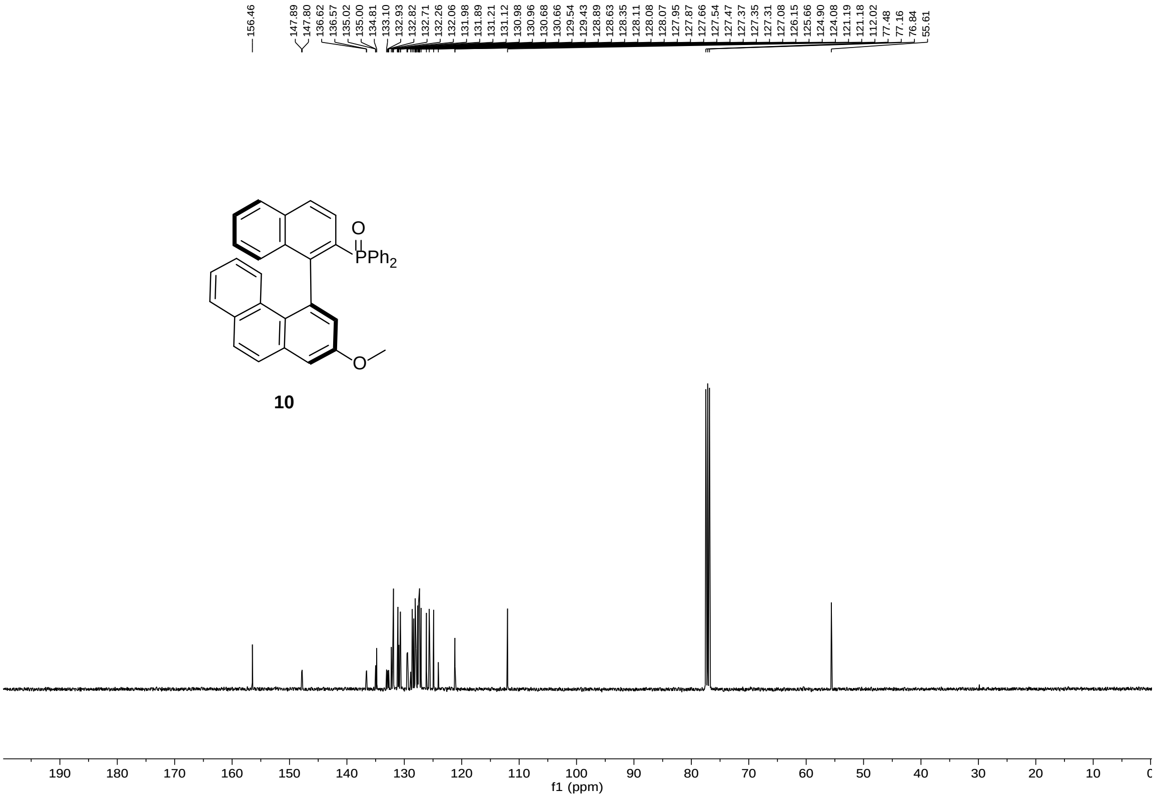

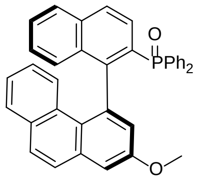
O
PPh2
O

10

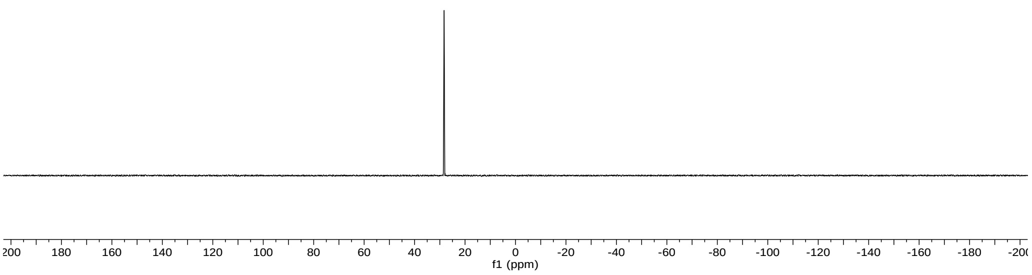
28.29
200 180 160 140 120 100 80 60 40 20 0 -20 -40 -60 -80 -100 -120 -140 -160 -180 -200
f1 (ppm)

Compound 11

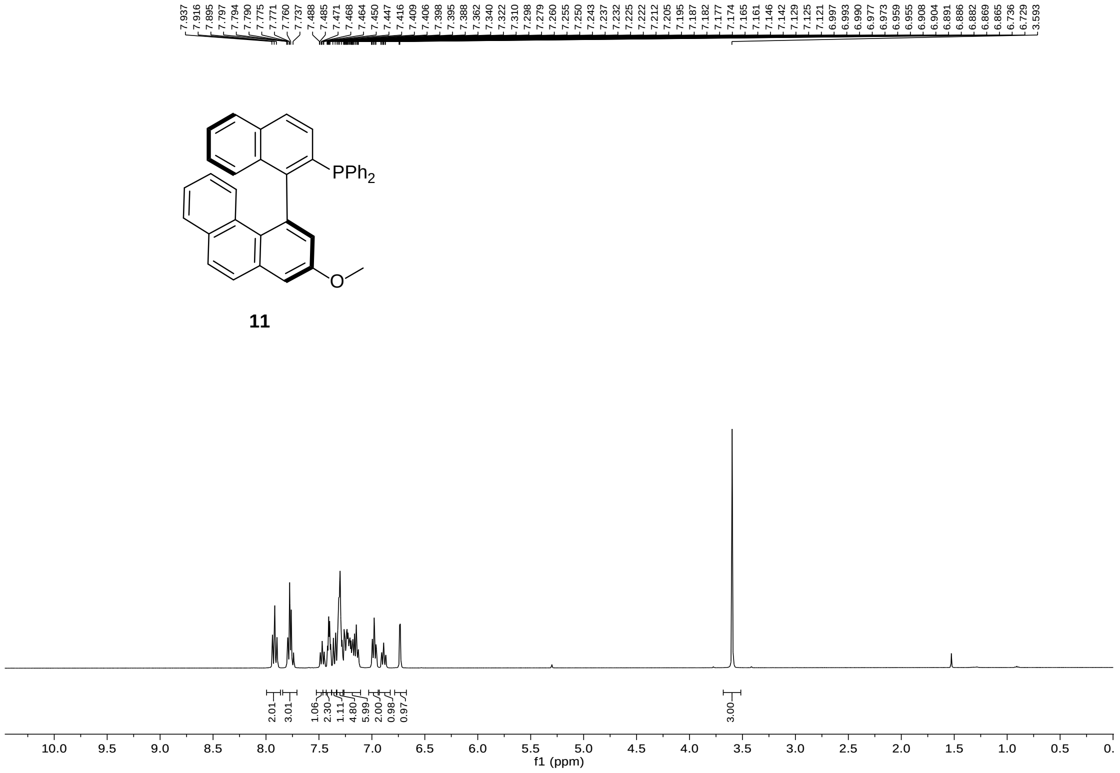

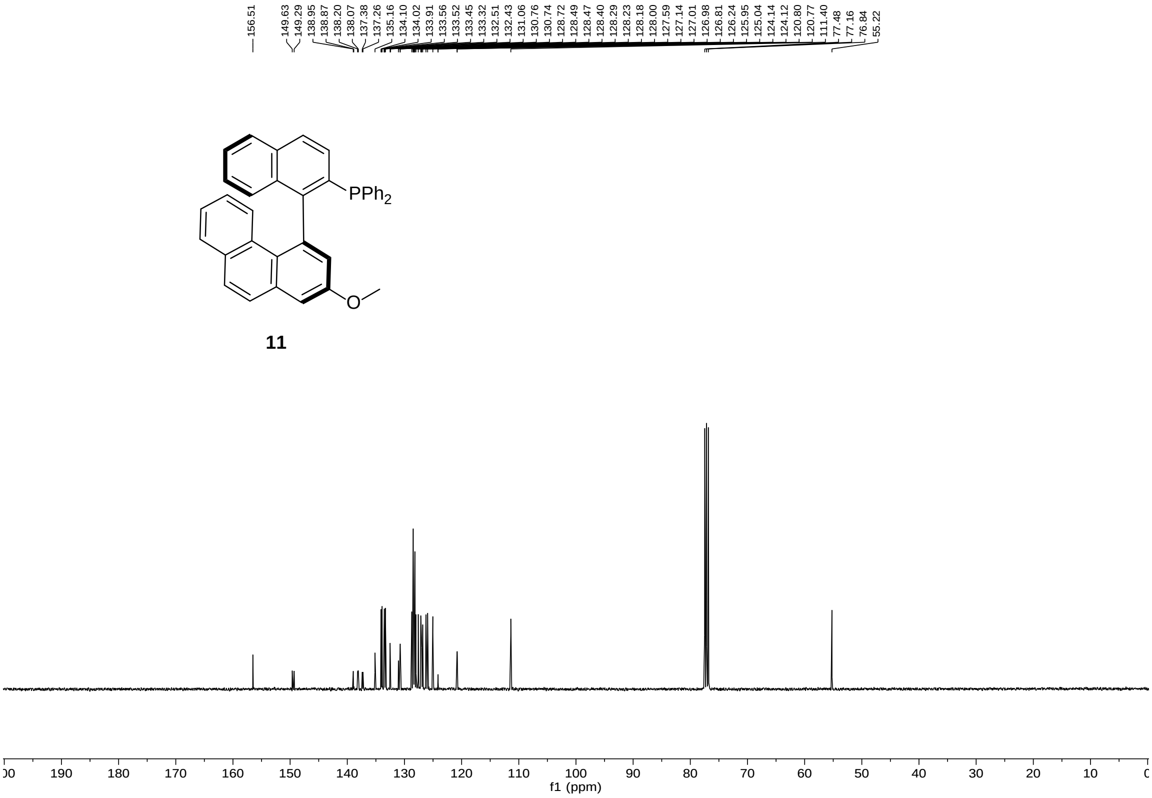

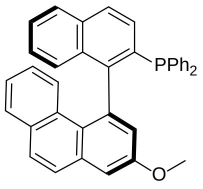
PPh2
O

11

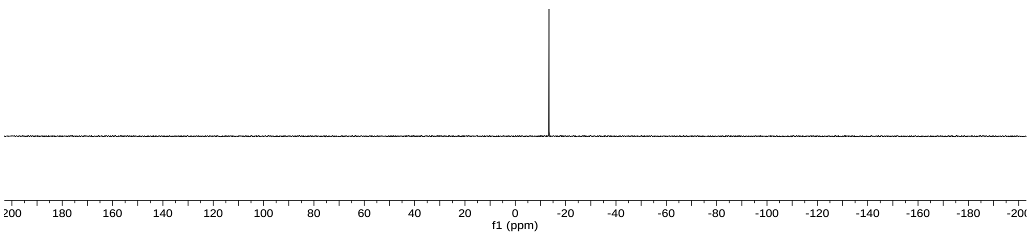
-13.38
200 180 160 140 120 100 80 60 40 20 0 -20 -40 -60 -80 -100 -120 -140 -160 -180 -200
f1 (ppm)

Compound 12

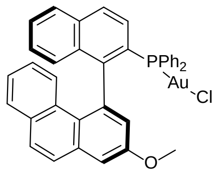

12

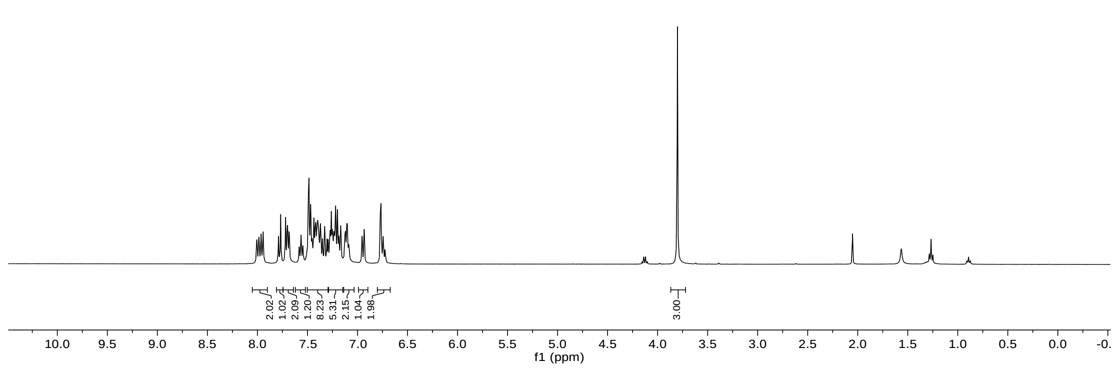

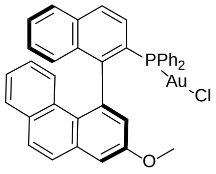

12

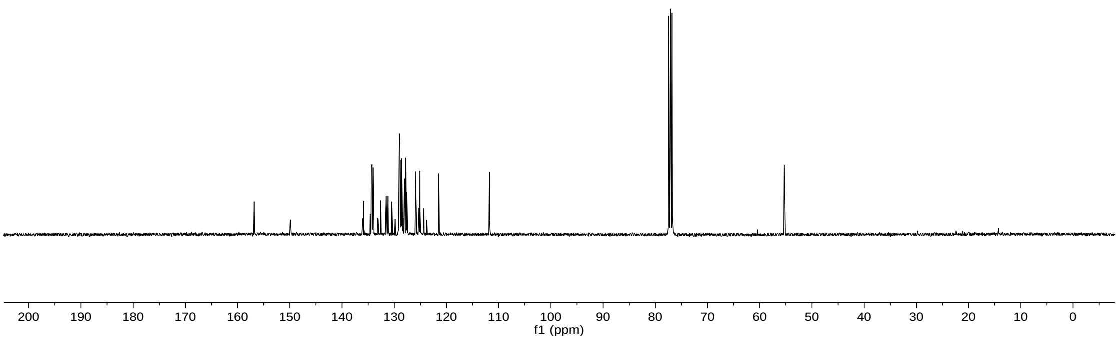

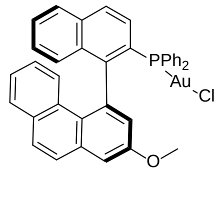

PPh2
Au
Cl
O

12

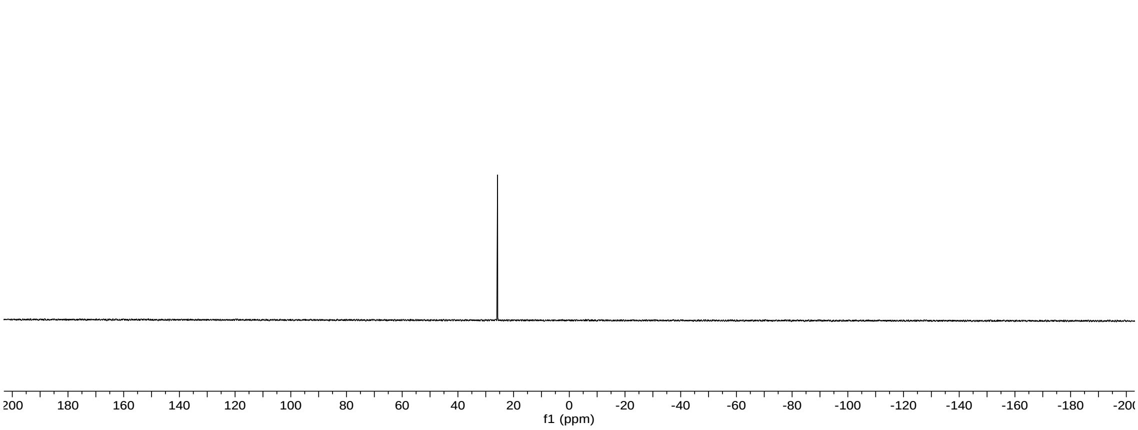

25.81
200
180
160
140
120
100
80
60
40
20
0
-20
-40
-60
-80
-100
-120
-140
-160
-180
f1 (ppm)

9. HPLC spectra

Compound 4a

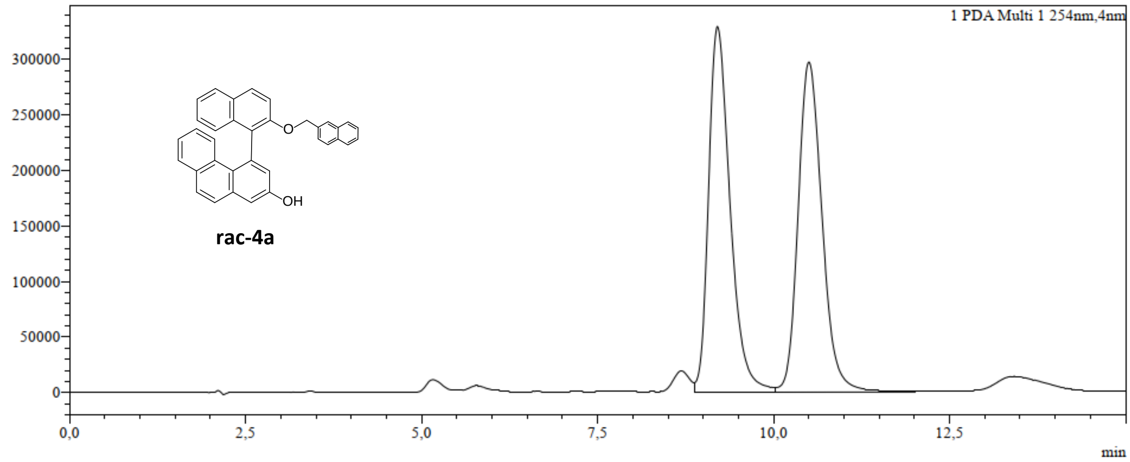

PDA Ch1 254nm

Peak#	Ret. Time	Area	Height	Area%
1	9,204	7008378	329294	49,849
2	10,503	7050869	297180	50,151
Total		14059247	626474	100,000

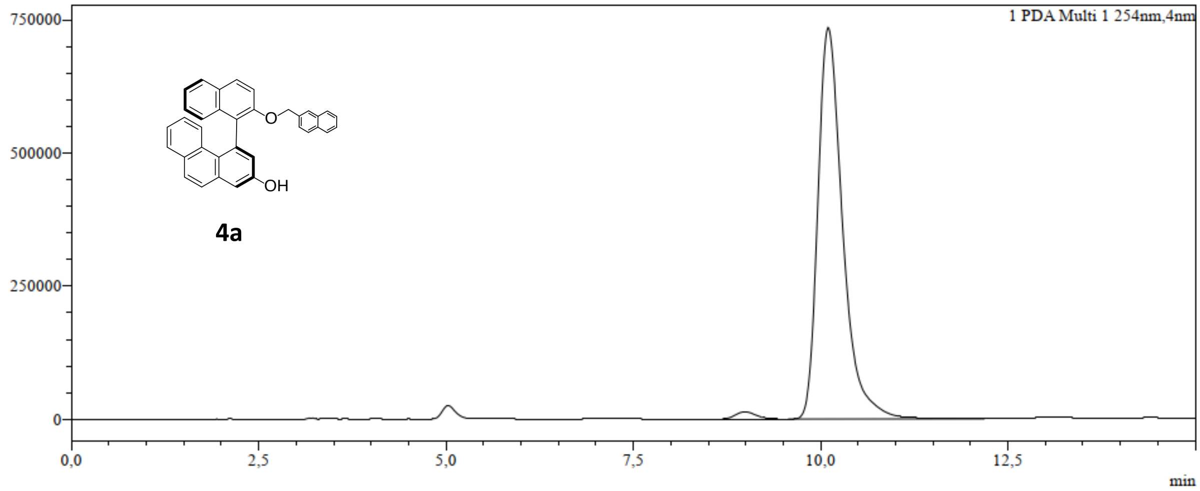

PDA Ch1 254nm

Peak#	Ret. Time	Area	Height	Area%
1	8,990	265630	13803	1,545
2	10,102	16928078	735185	98,455
Total		17193708	748988	100,000

Compound 4b

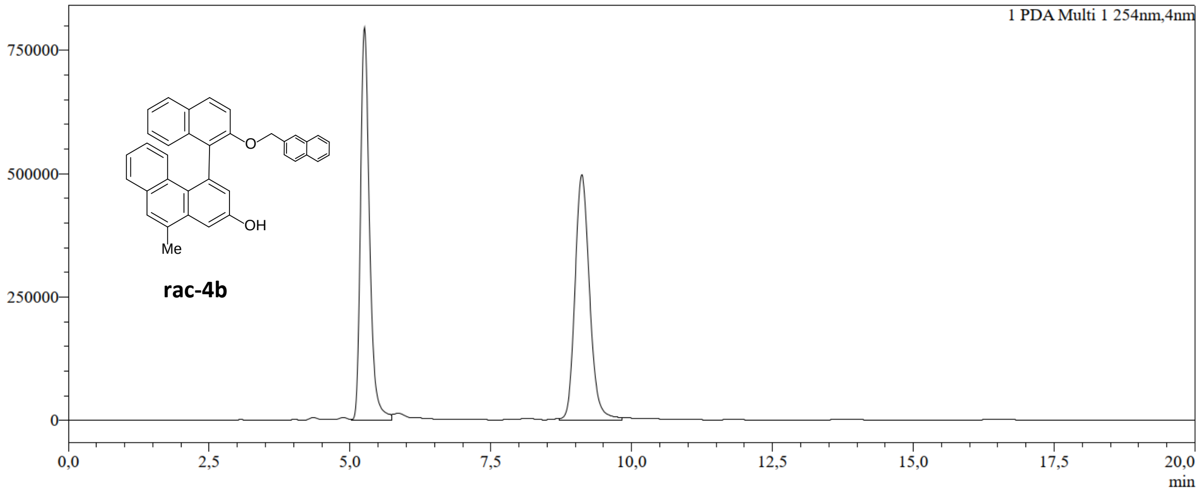

PDA Ch1 254nm

Peak#	Ret. Time	Area	Height	Area%
1	5,256	8914551	796540	49,723
2	9,115	9013744	497831	50,277
Total		17928296	1294371	100,000

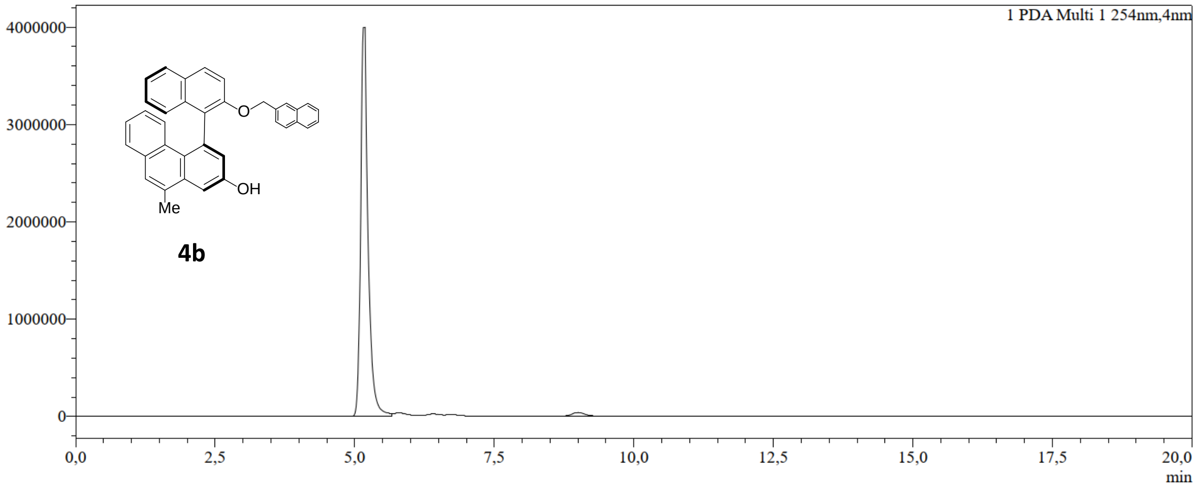

PDA Ch1 254nm

Peak#	Ret. Time	Area	Height	Area%
1	5,168	36856530	3998240	98,209
2	9,004	672187	38305	1,791
Total		37528717	4036546	100,000

Compound 4c

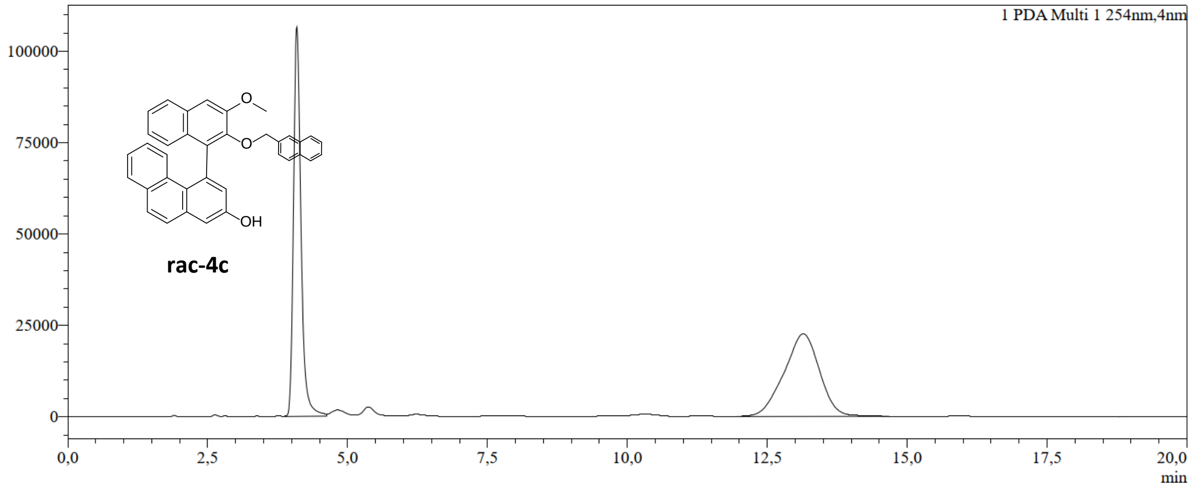

PDA Ch1 254nm

Peak#	Ret. Time	Area	Height	Area%
1	4,092	990111	106453	49,787
2	13,143	998589	22590	50,213
Total		1988700	129042	100,000

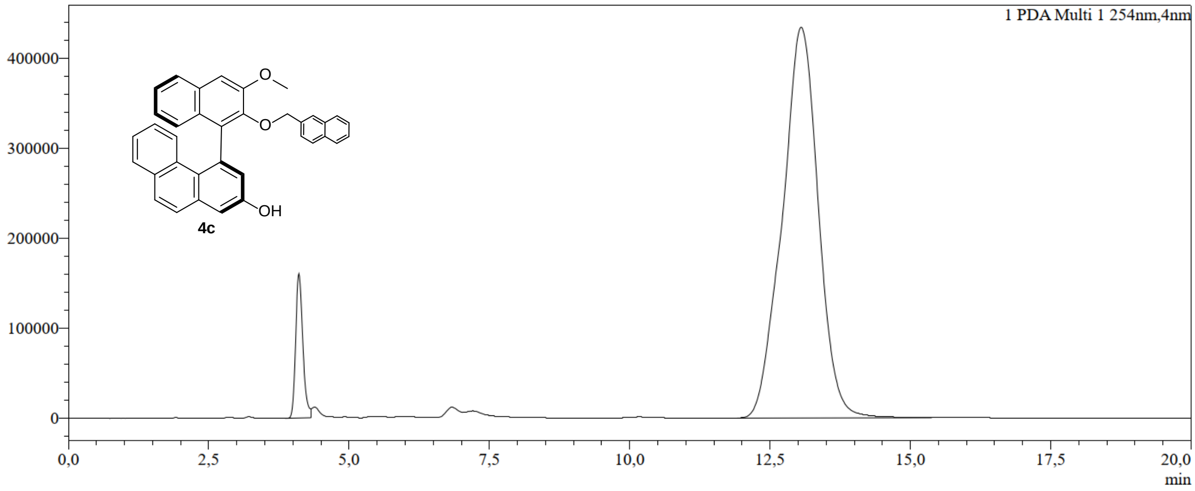

PDA Ch1 254nm

Peak#	Ret. Time	Area	Height	Area%
1	4,106	1429489	160376	6,869
2	13,055	19381015	433636	93,131
Total		20810504	594012	100,000

Compound 4d

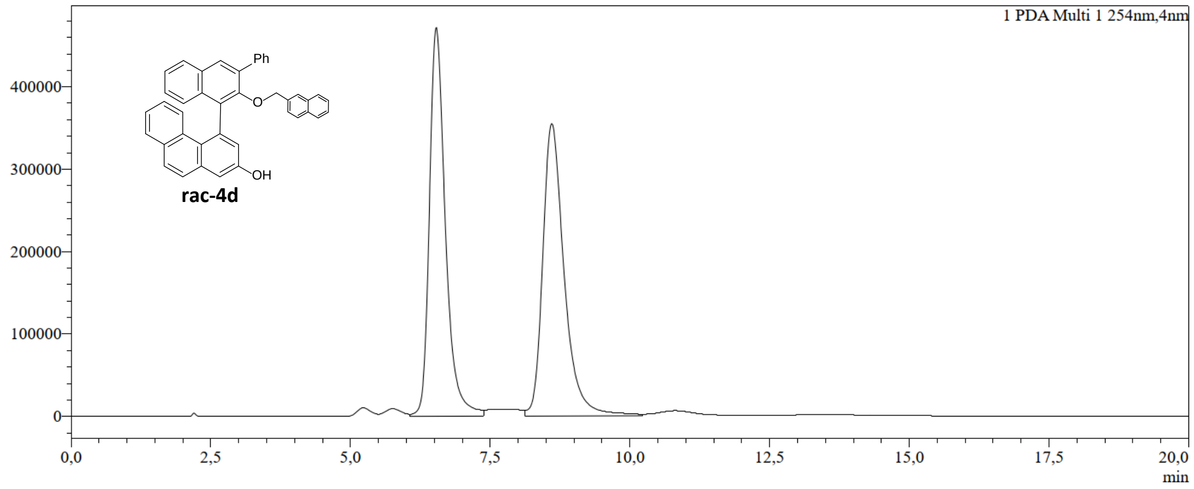

PDA Ch1 254nm

Peak#	Ret. Time	Area	Height	Area%
1	6,534	9394965	471668	49,743
2	8,602	9492233	354739	50,257
Total		18887199	826407	100,000

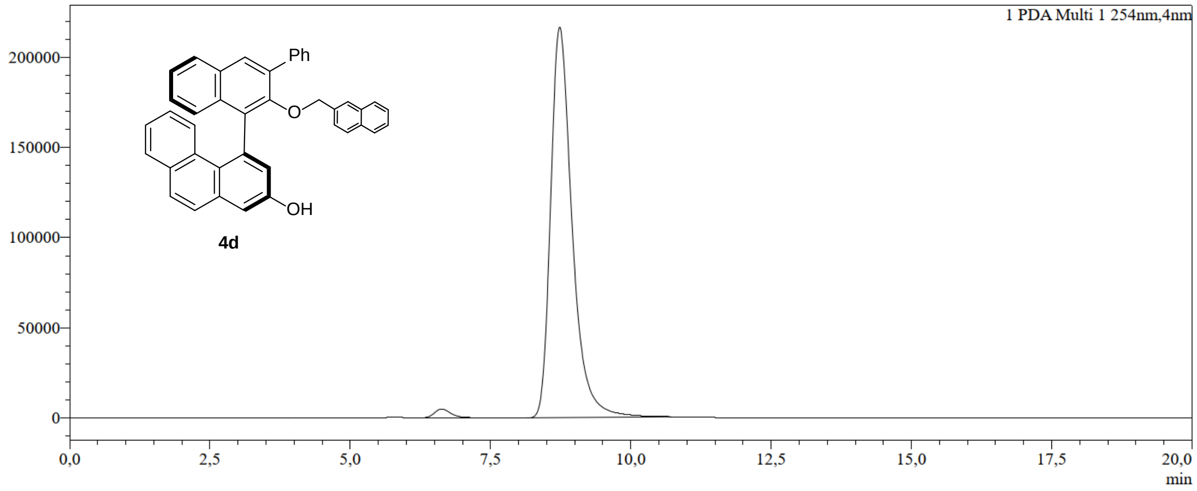

PDA Ch1 254nm

Peak#	Ret. Time	Area	Height	Area%
1	6,628	103213	4963	1,757
2	8,732	5771450	216383	98,243
Total		5874663	221346	100,000

Compound 4e

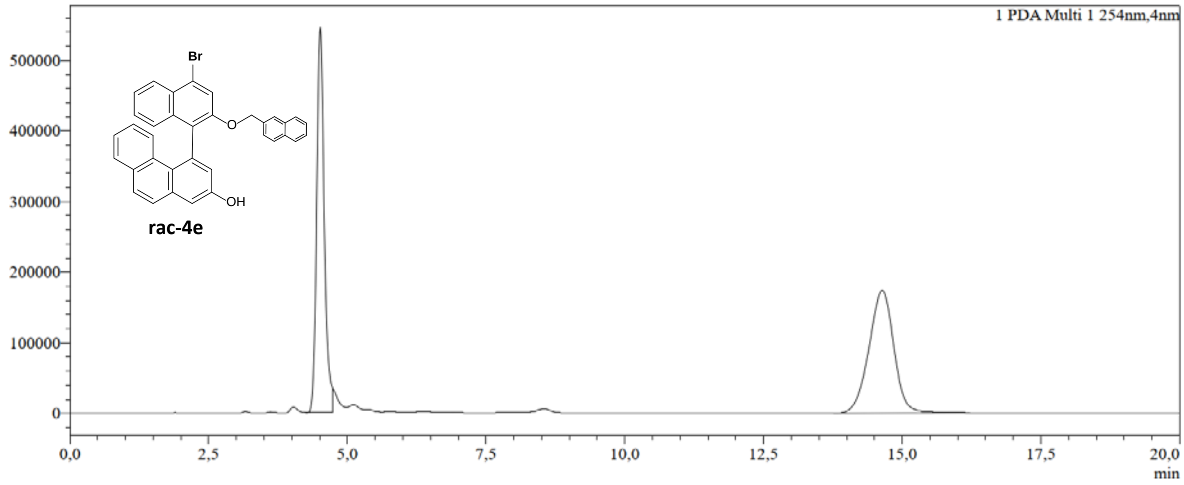

PDA Ch1 254nm

Peak#	Ret. Time	Area	Height	Area%
1	4,511	5196519	545894	48,787
2	14,641	5454918	174097	51,213
Total		10651437	719991	100,000

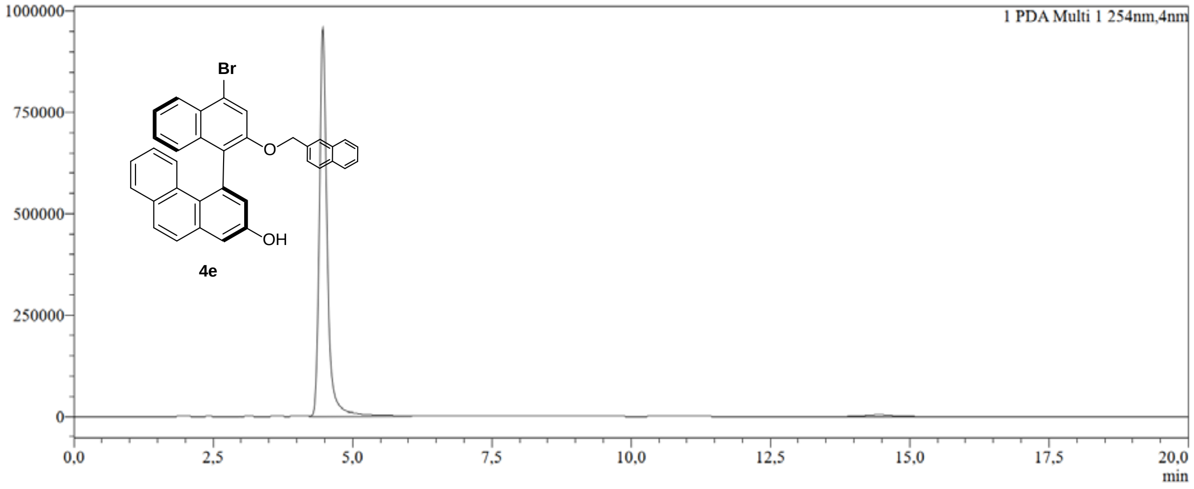

PDA Ch1 254nm

Peak#	Ret. Time	Area	Height	Area%
1	4,467	9441788	957531	98,601
2	14,449	133923	4551	1,399
Total		9575711	962083	100,000

Compound 4f

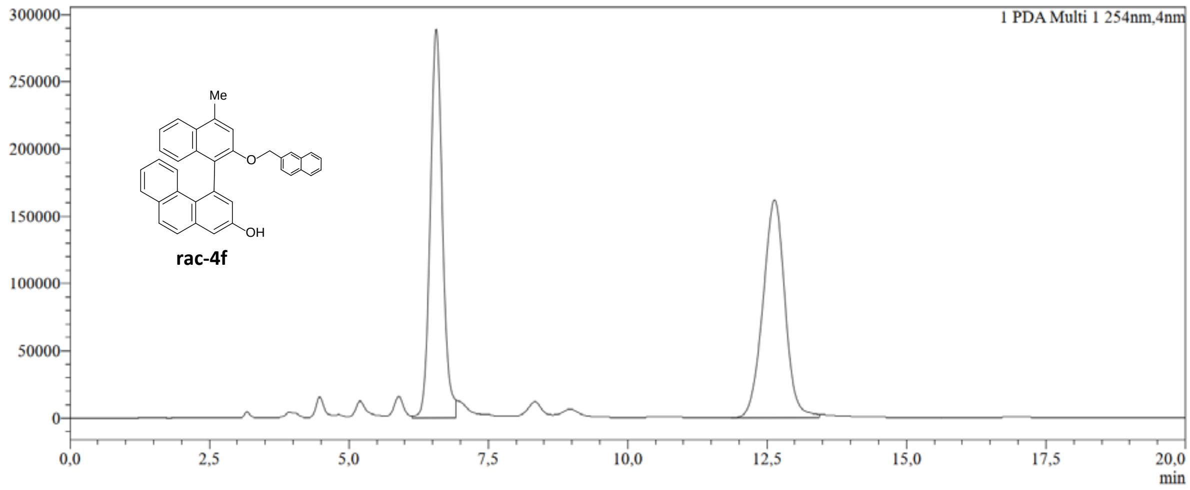

PDA Ch1 254nm

Peak#	Ret. Time	Area	Height	Area%
1	6,567	4378153	289019	49,571
2	12,634	4453915	161843	50,429
Total		8832068	450862	100,000

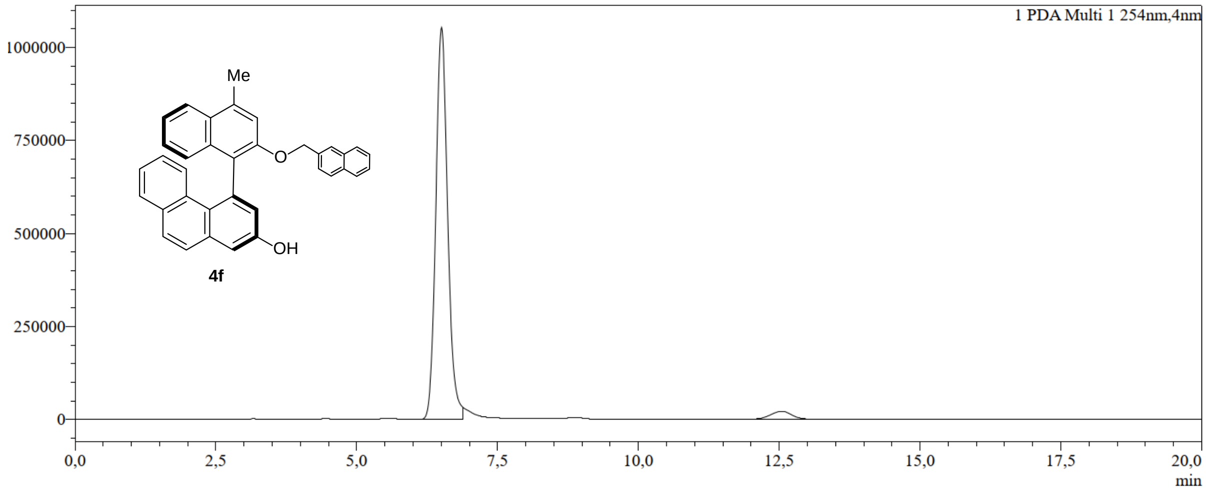

PDA Ch1 254nm

Peak#	Ret. Time	Area	Height	Area%
1	6,505	15462666	1053034	96,308
2	12,541	592783	21936	3,692
Total		16055448	1074971	100,000

Compound 4g

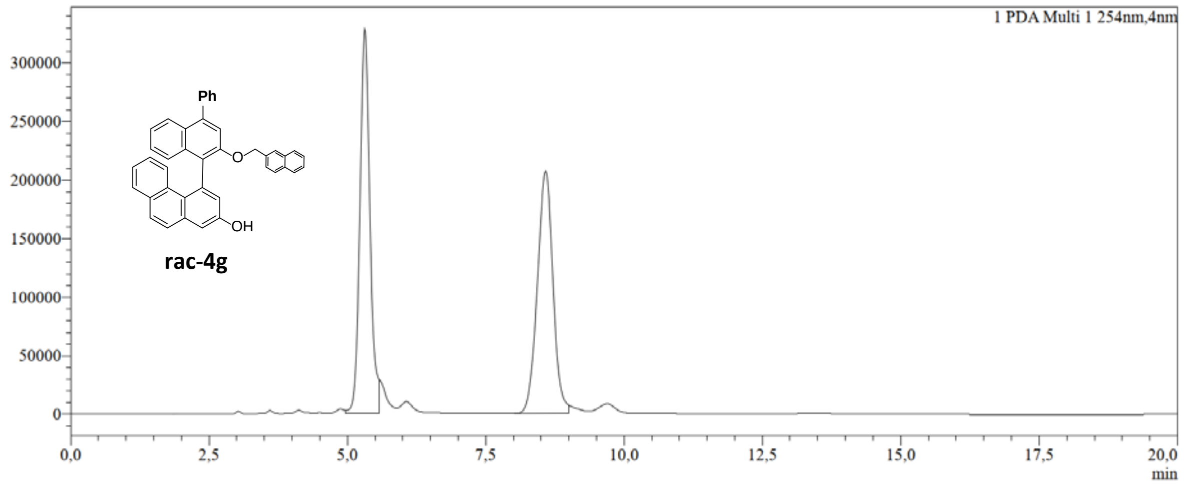

PDA Ch1 254nm

Peak#	Ret. Time	Area	Height	Area%
1	5,314	4159575	328523	50,589
2	8,582	4062749	206850	49,411
Total		8222325	535373	100,000

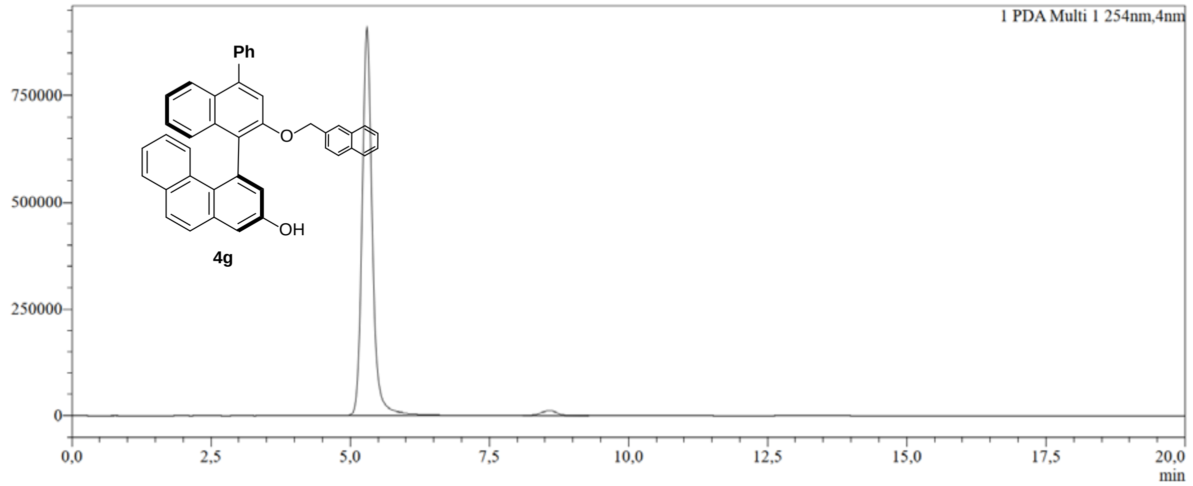

PDA Ch1 254nm

Peak#	Ret. Time	Area	Height	Area%
1	5,301	11509046	907581	97,978
2	8,579	237479	11801	2,022
Total		11746526	919382	100,000

Compound 4h

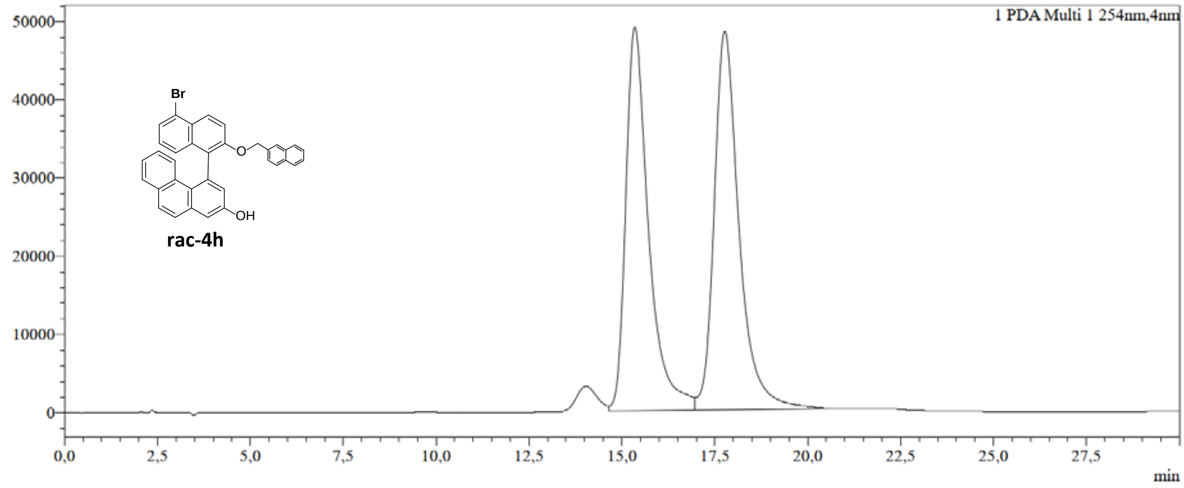

PDA Ch1 254nm

Peak#	Ret. Time	Area	Height	Area%
1	15,348	2099921	49056	48,233
2	17,770	2253750	48423	51,767
Total		4353671	97478	100,000

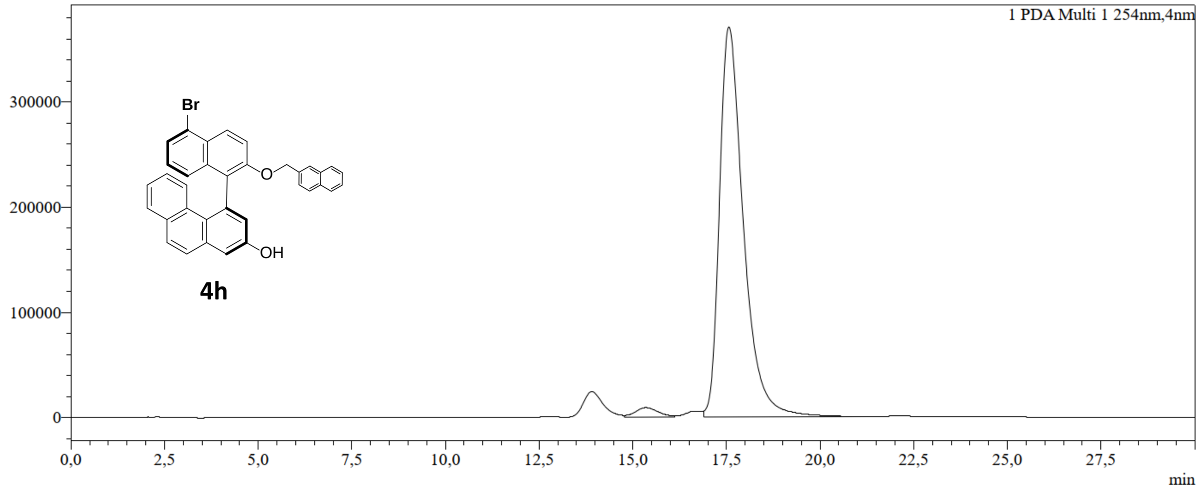

PDA Ch1 254nm

Peak#	Ret. Time	Area	Height	Area%
1	15,350	390070	9071	2,310
2	17,567	16498605	370662	97,690
Total		16888675	379733	100,000

Compound 4i

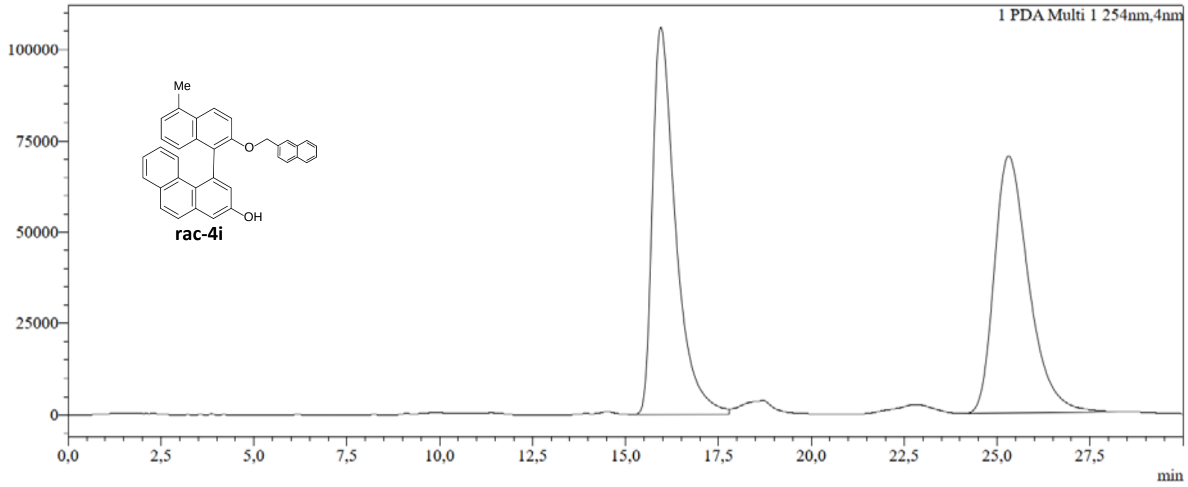

PDA Ch1 254nm

Peak#	Ret. Time	Area	Height	Area%
1	15,952	4595306	105984	50,380
2	25,312	4526071	70380	49,620
Total		9121376	176363	100,000

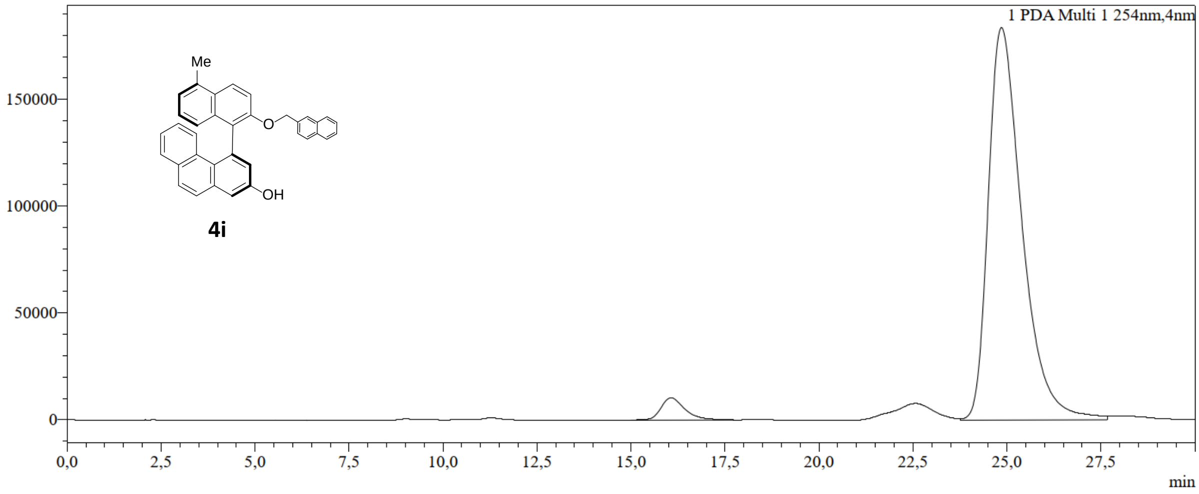

PDA Ch1 254nm

Peak#	Ret. Time	Area	Height	Area%
1	16,064	468247	10437	3,921
2	24,856	11472483	183724	96,079
Total		11940730	194161	100,000

Compound 4j

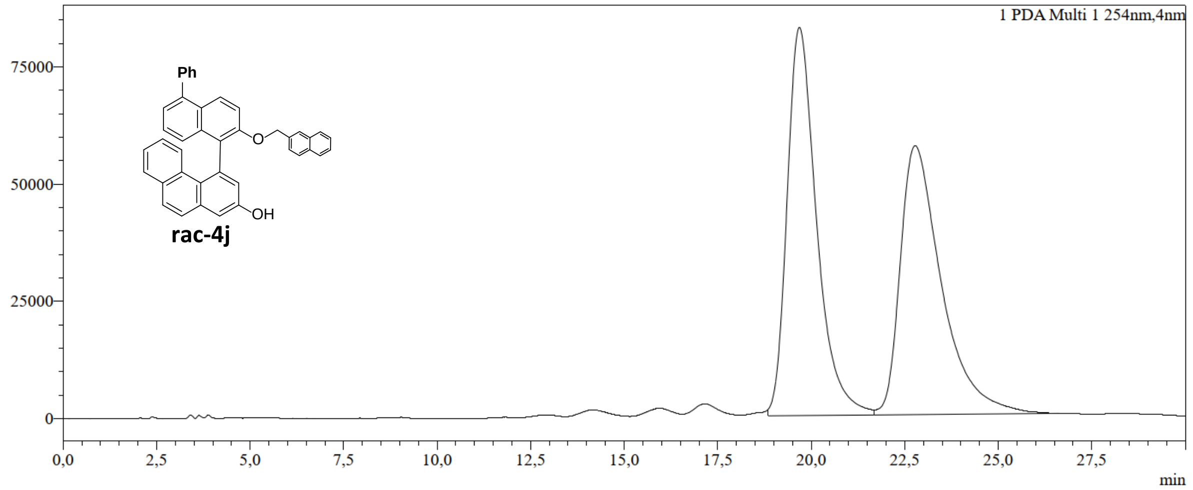

PDA Ch1 254nm

Peak#	Ret. Time	Area	Height	Area%
1	19,685	4541608	82811	50,761
2	22,782	4405521	57385	49,239
Total		8947130	140195	100,000

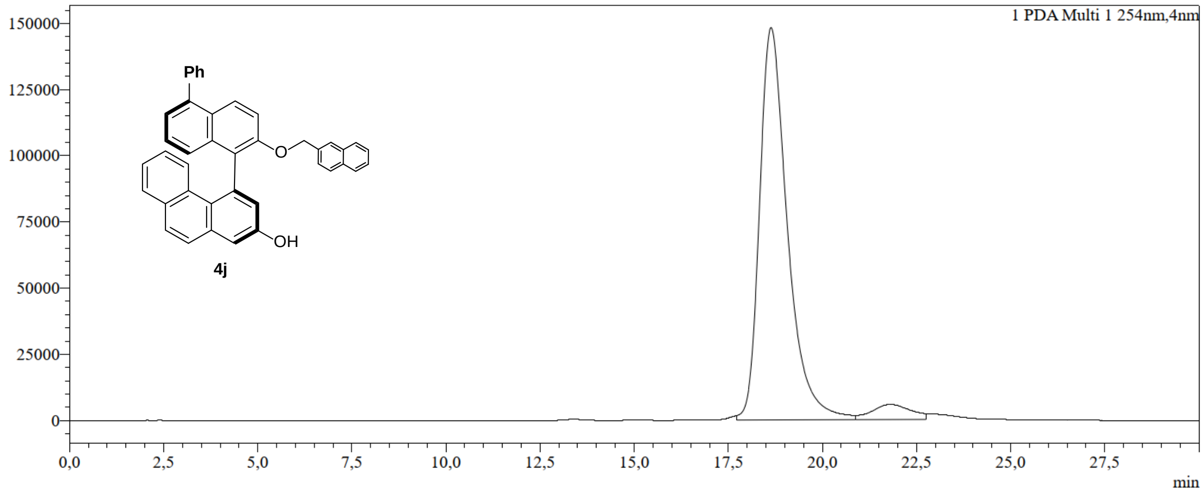

PDA Ch1 254nm

Peak#	Ret. Time	Area	Height	Area%
1	18,636	7632853	148202	94,800
2	21,810	418673	5735	5,200
Total		8051526	153938	100,000

Compound 4k

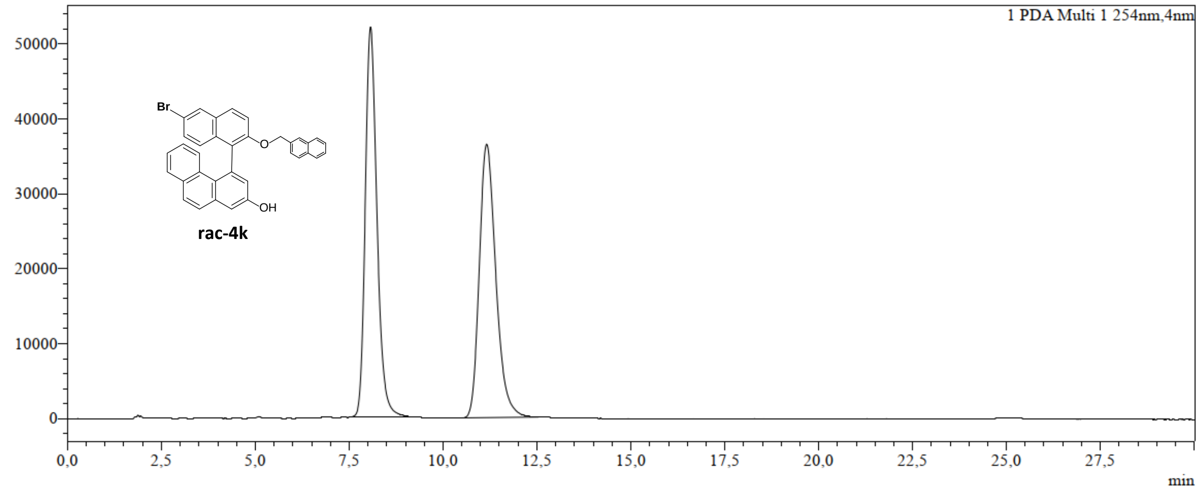

PDA Ch1 254nm

Peak#	Ret. Time	Area	Height	Area%
1	8,069	1131801	52042	50,352
2	11,162	1115958	36468	49,648
Total		2247760	88510	100,000

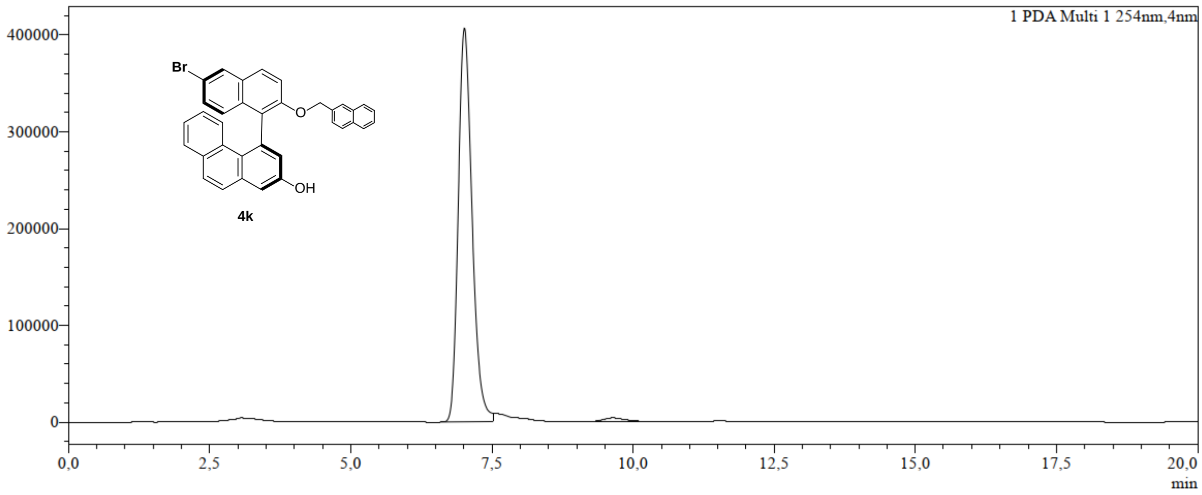

PDA Ch1 254nm

Peak#	Ret. Time	Area	Height	Area%
1	7,012	6933057	406628	98,677
2	9,641	92952	3591	1,323
Total		7026008	410219	100,000

Compound 4l

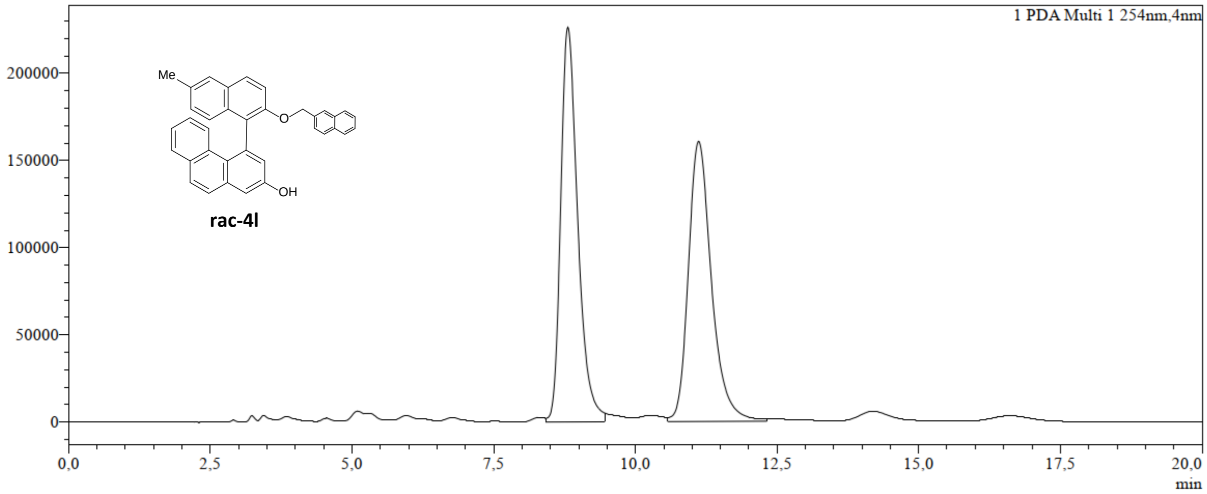

PDA Ch1 254nm

Peak#	Ret. Time	Area	Height	Area%
1	8,810	4735042	226462	50,908
2	11,117	4566185	160775	49,092
Total		9301227	387238	100,000

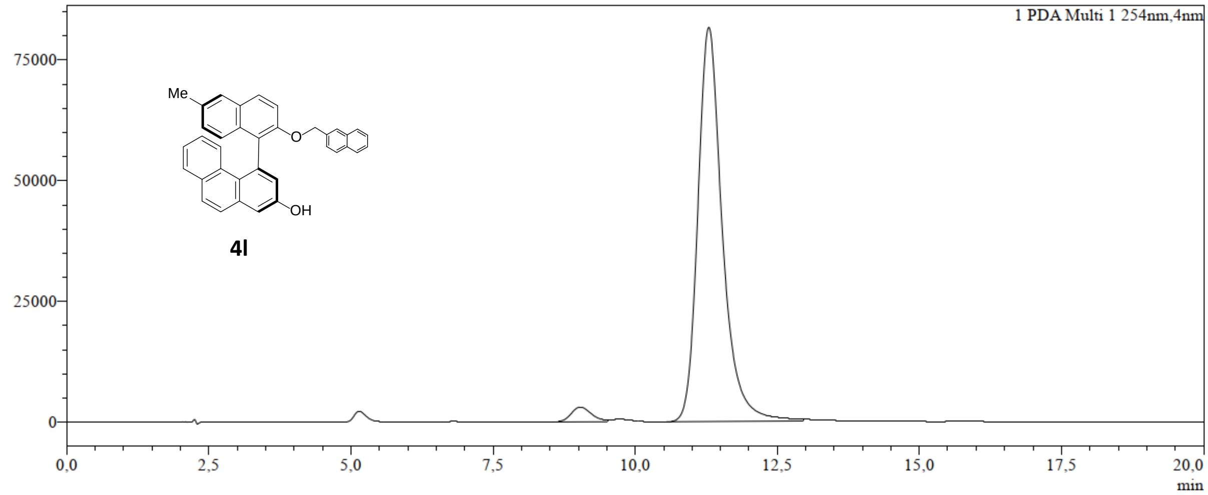

PDA Ch1 254nm

Peak#	Ret. Time	Area	Height	Area%
1	9,036	77472	3083	3,061
2	11,297	2453436	81667	96,939
Total		2530908	84750	100,000

Compound 4m

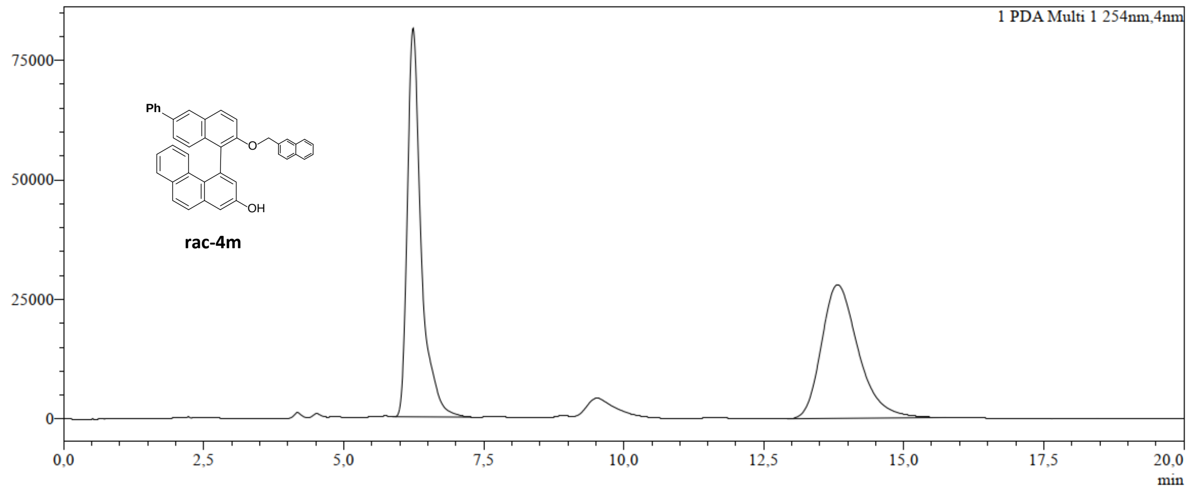

PDA Ch1 254nm

Peak#	Ret. Time	Area	Height	Area%
1	6,238	1390886	81224	52,035
2	13,820	1282090	27971	47,965
Total		2672976	109196	100,000

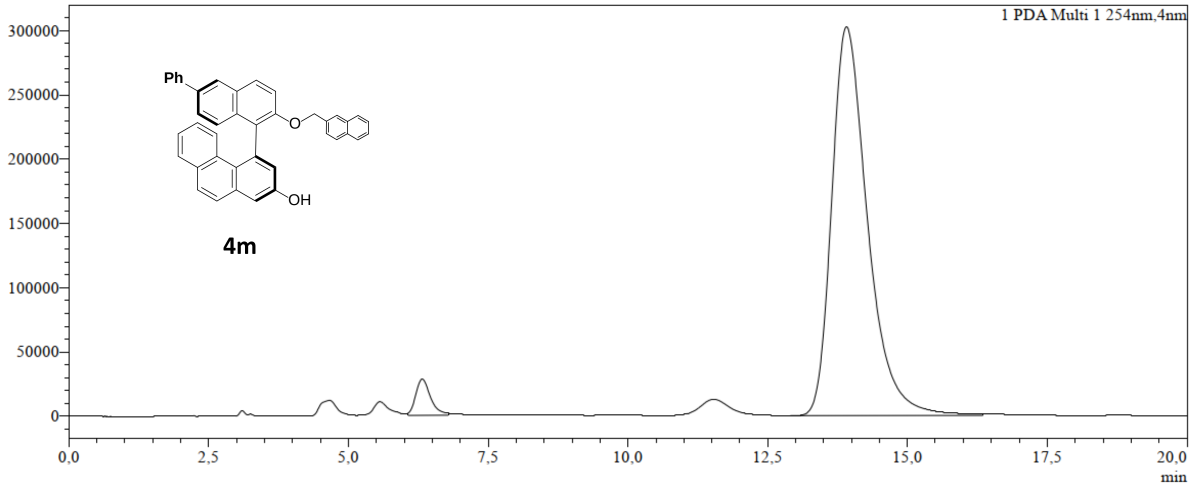

PDA Ch1 254nm

Peak#	Ret. Time	Area	Height	Area%
1	6,319	520968	28211	3,718
2	13,912	13492046	302389	96,282
Total		14013014	330600	100,000

Compound 4n

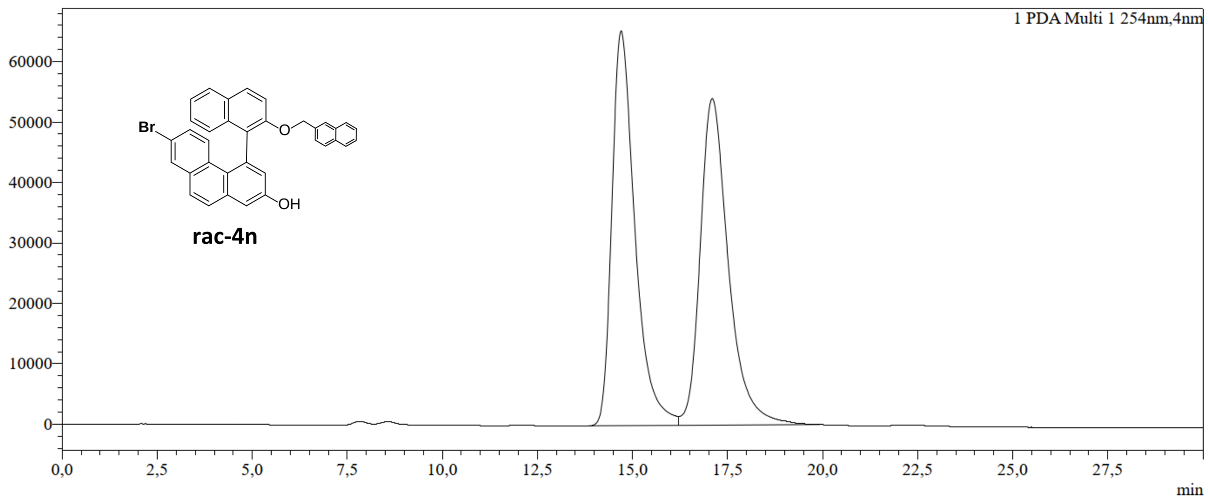

PDA Ch1 254nm

Peak#	Ret. Time	Area	Height	Area%
1	14,701	2760584	65313	49,700
2	17,099	2793962	54040	50,300
Total		5554546	119353	100,000

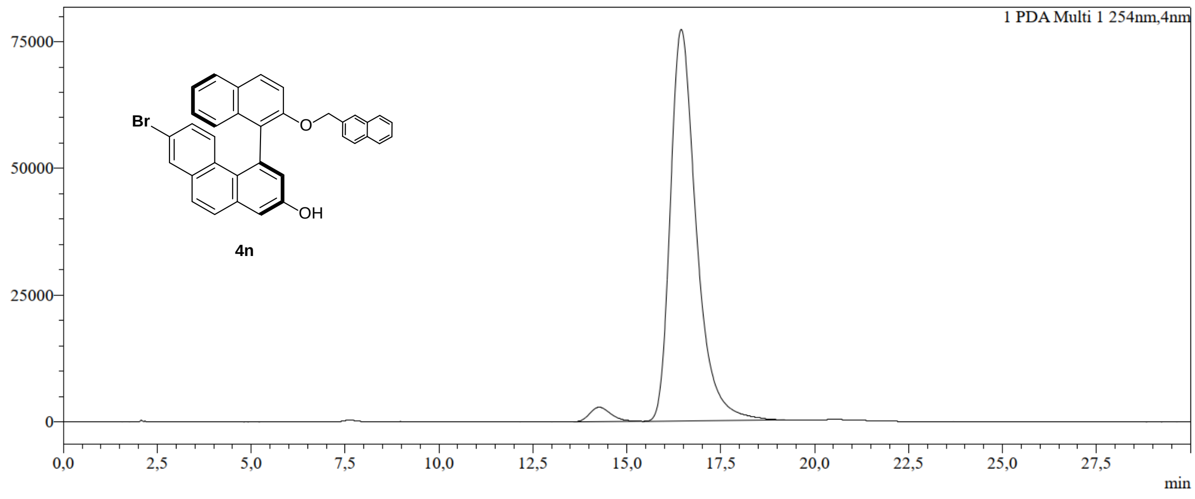

PDA Ch1 254nm

Peak#	Ret. Time	Area	Height	Area%
1	14,264	113163	2851	2,955
2	16,449	3716234	77260	97,045
Total		3829398	80111	100,000

Compound 4o

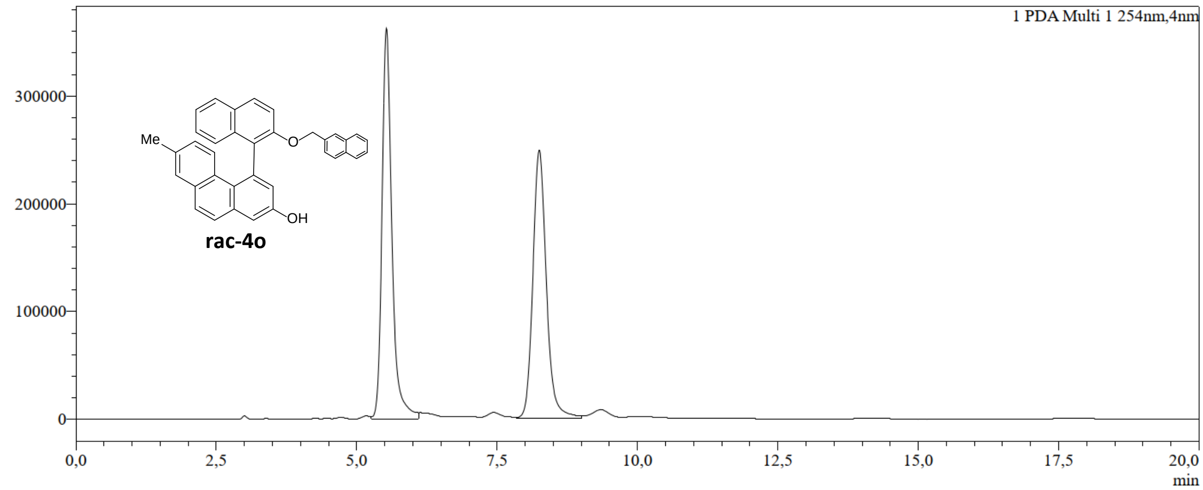

PDA Ch1 254nm

Peak#	Ret. Time	Area	Height	Area%
1	5,529	4447970	362306	51,510
2	8,251	4187161	248977	48,490
Total		8635131	611283	100,000

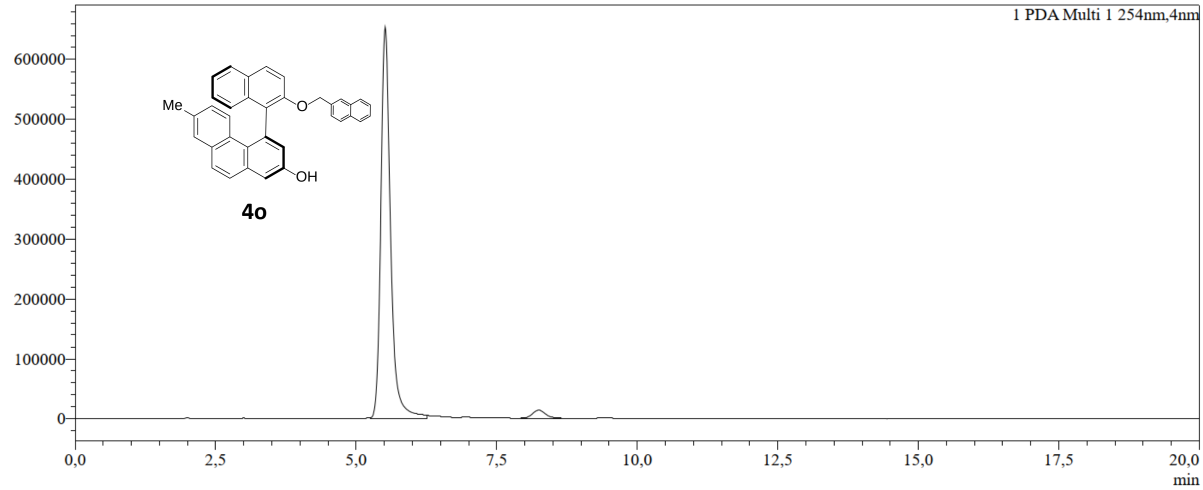

PDA Ch1 254nm

Peak#	Ret. Time	Area	Height	Area%
1	5,515	7911911	653883	96,972
2	8,246	247027	13984	3,028
Total		8158938	667868	100,000

Compound 4p

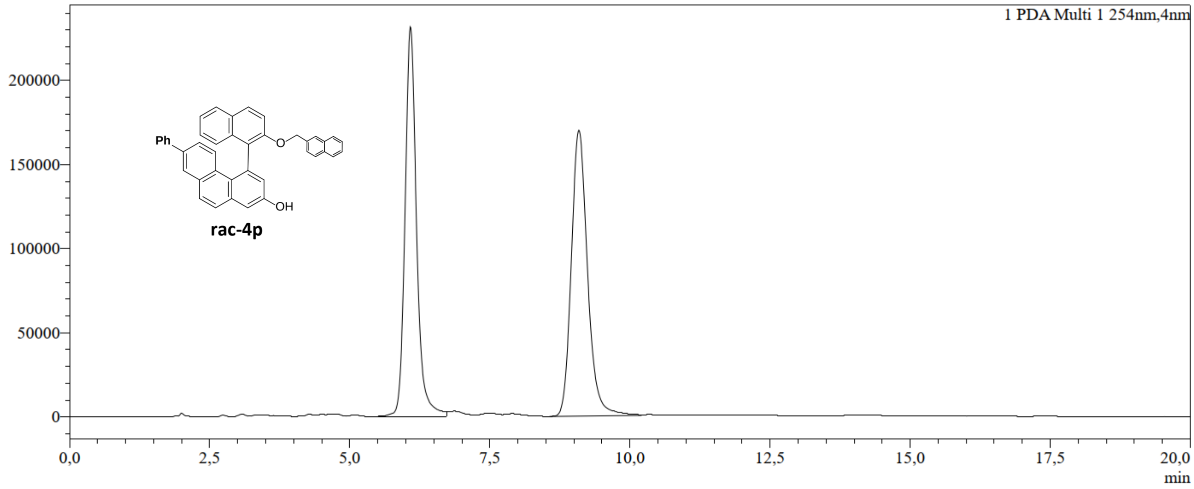

PDA Ch1 254nm

Peak#	Ret. Time	Area	Height	Area%
1	6,083	3316874	231491	49,180
2	9,089	3427456	169840	50,820
Total		6744331	401331	100,000

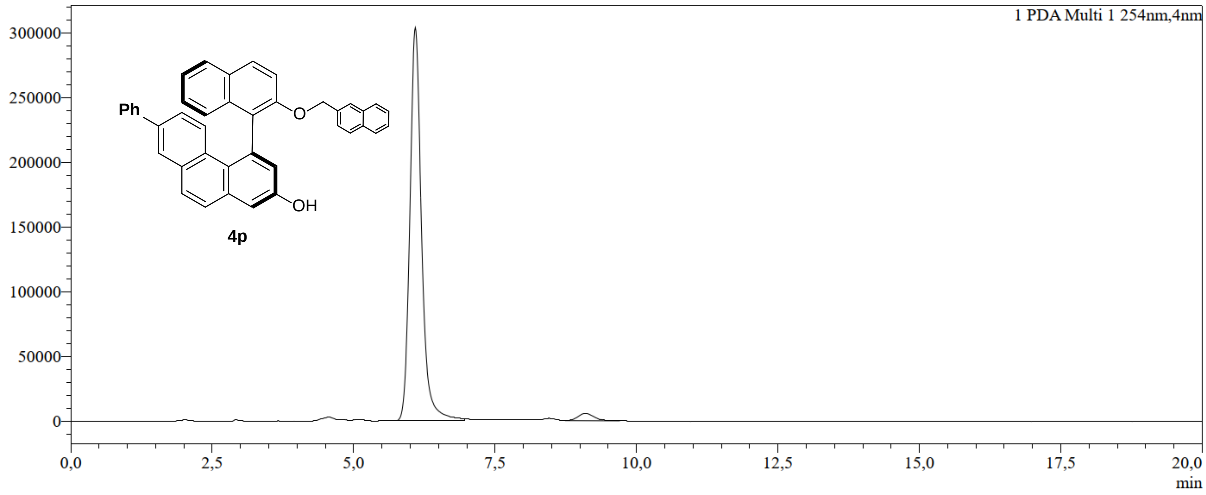

PDA Ch1 254nm

Peak#	Ret. Time	Area	Height	Area%
1	6,090	4349408	303463	97,478
2	9,097	112540	5646	2,522
Total		4461947	309109	100,000

Compound 4q

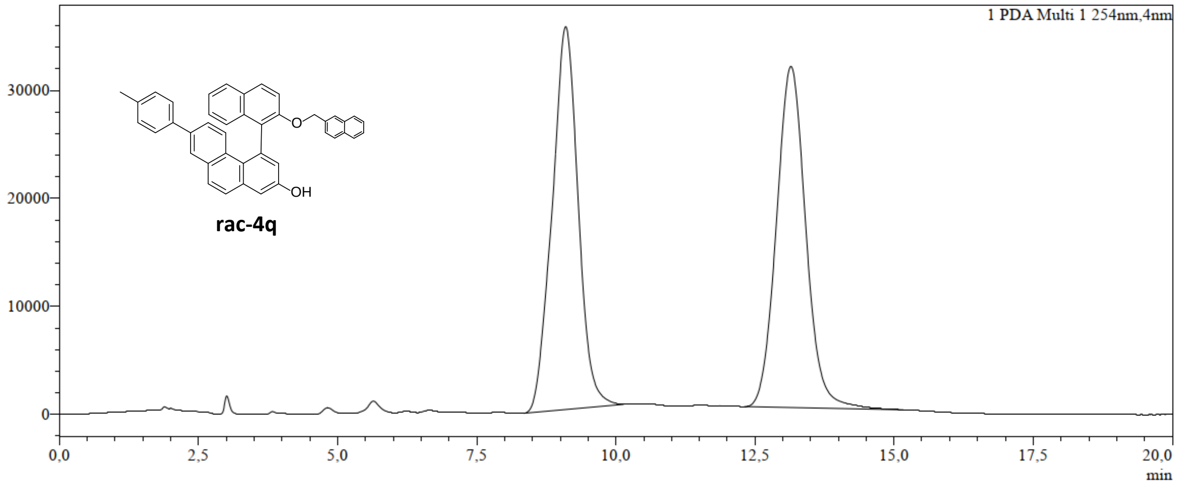

PDA Ch1 254nm

Peak#	Ret. Time	Area	Height	Area%
1	9,099	1154477	35464	49,850
2	13,147	1161404	31581	50,150
Total		2315881	67045	100,000

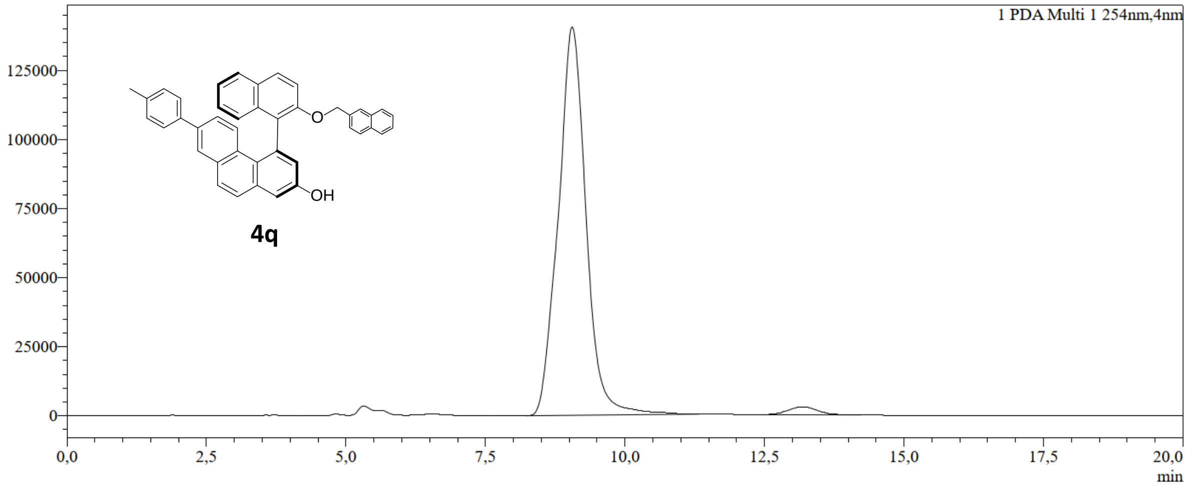

PDA Ch1 254nm

Peak#	Ret. Time	Area	Height	Area%
1	9,053	4862394	140539	97,834
2	13,183	107629	2905	2,166
Total		4970023	143443	100,000

Compound 4r

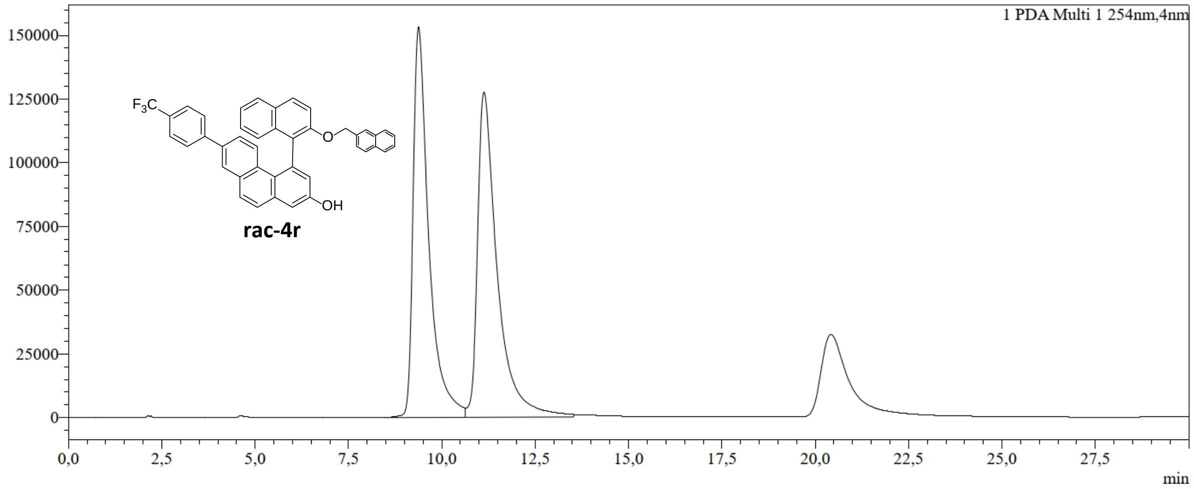

PDA Ch1 254nm

Peak#	Ret. Time	Area	Height	Area%
1	9,377	4476808	153209	48,864
2	11,130	4685003	127476	51,136
Total		9161811	280685	100,000

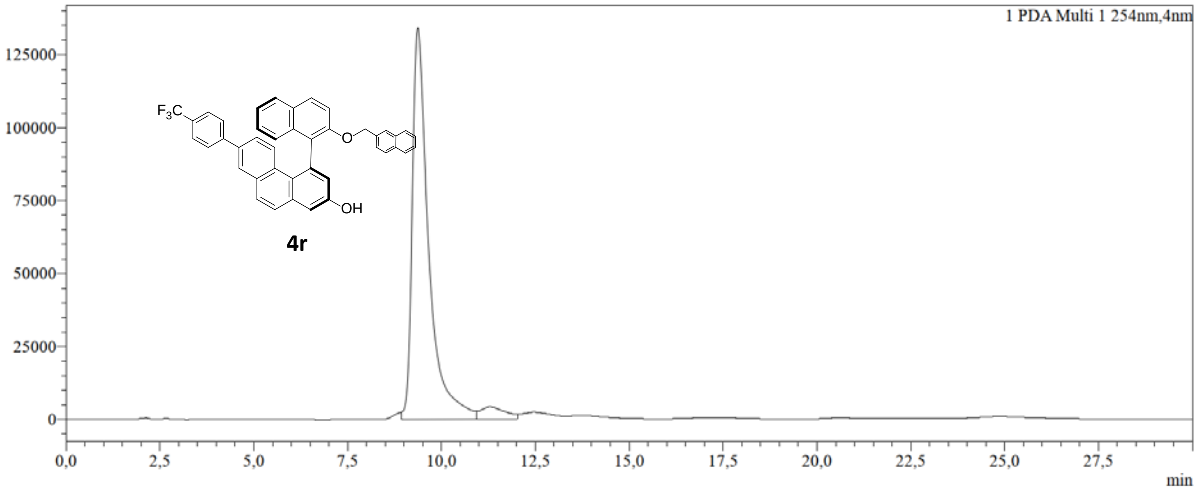

PDA Ch1 254nm

Peak#	Ret. Time	Area	Height	Area%
1	9,371	4074236	134307	95,184
2	11,296	206152	4325	4,816
Total		4280389	138632	100,000

Compound 4s

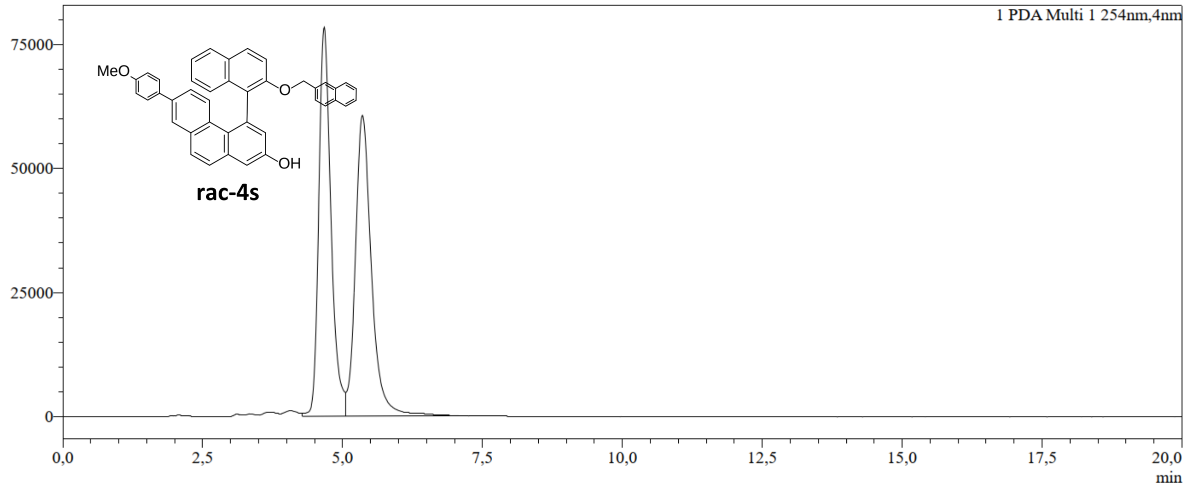

PDA Ch1 254nm

Peak#	Ret. Time	Area	Height	Area%
1	4,670	1192996	78321	49,624
2	5,355	1211090	60566	50,376
Total		2404086	138886	100,000

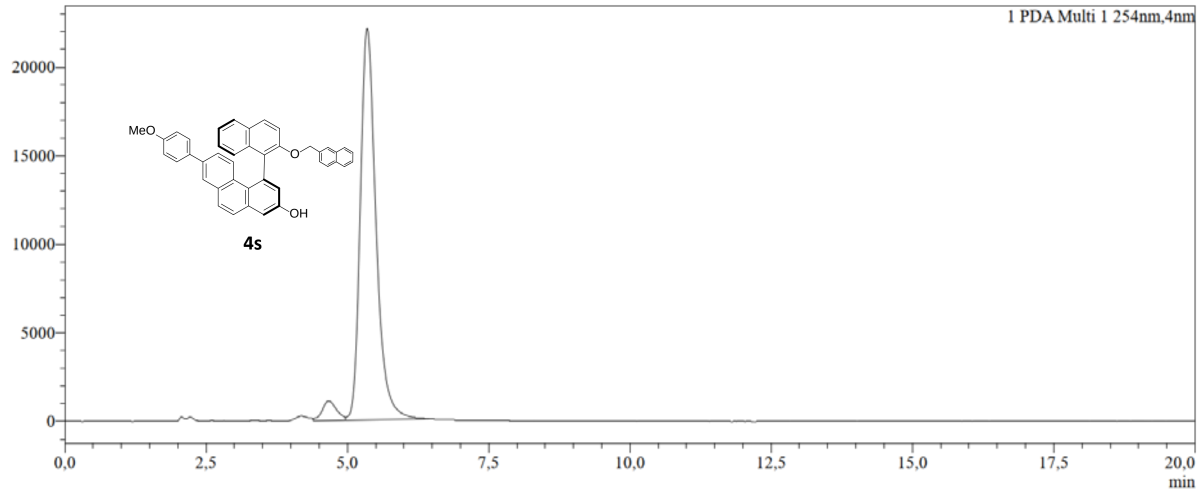

PDA Ch1 254nm

Peak#	Ret. Time	Area	Height	Area%
1	4,670	19898	1124	4,444
2	5,350	427875	22099	95,556
Total		447773	23223	100,000

Compound 4t

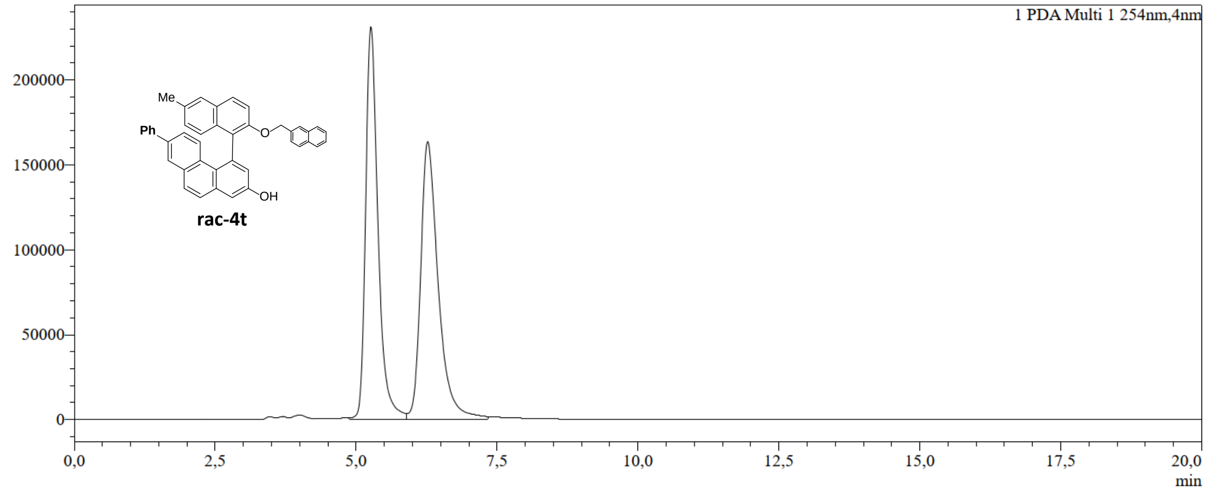

PDA Ch1 254nm

Peak#	Ret. Time	Area	Height	Area%
1	5,262	3538287	230939	49,987
2	6,270	3540128	163477	50,013
Total		7078415	394416	100,000

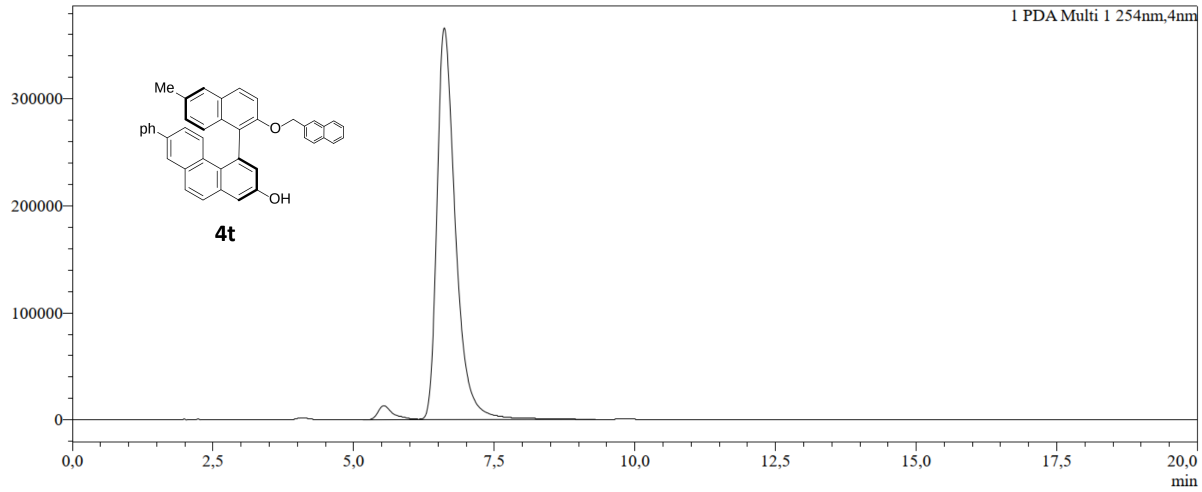

PDA Ch1 254nm

Peak#	Ret. Time	Area	Height	Area%
1	5,537	247151	13152	2,864
2	6,611	8381381	366018	97,136
Total		8628532	379169	100,000

Compound 4u

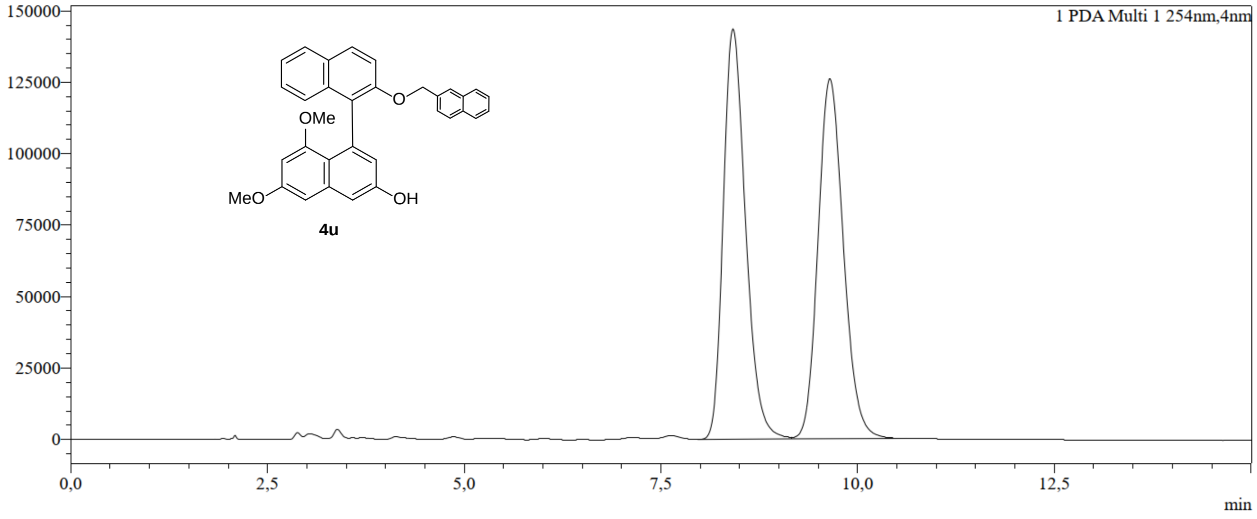

PDA Ch1 254nm

Peak#	Ret. Time	Area	Height	Area%
1	8,417	2833009	143672	49,986
2	9,648	2834559	126002	50,014
Total		5667568	269673	100,000

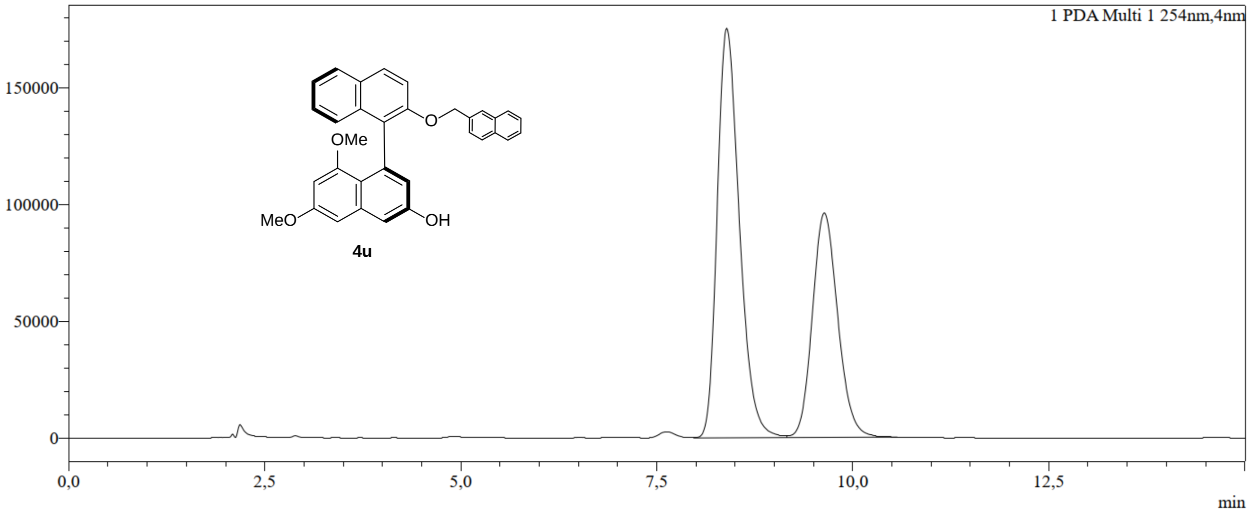

PDA Ch1 254nm

Peak#	Ret. Time	Area	Height	Area%
1	8,394	3453395	175325	61,452
2	9,638	2166304	96115	38,548
Total		5619699	271440	100,000

Compound 7

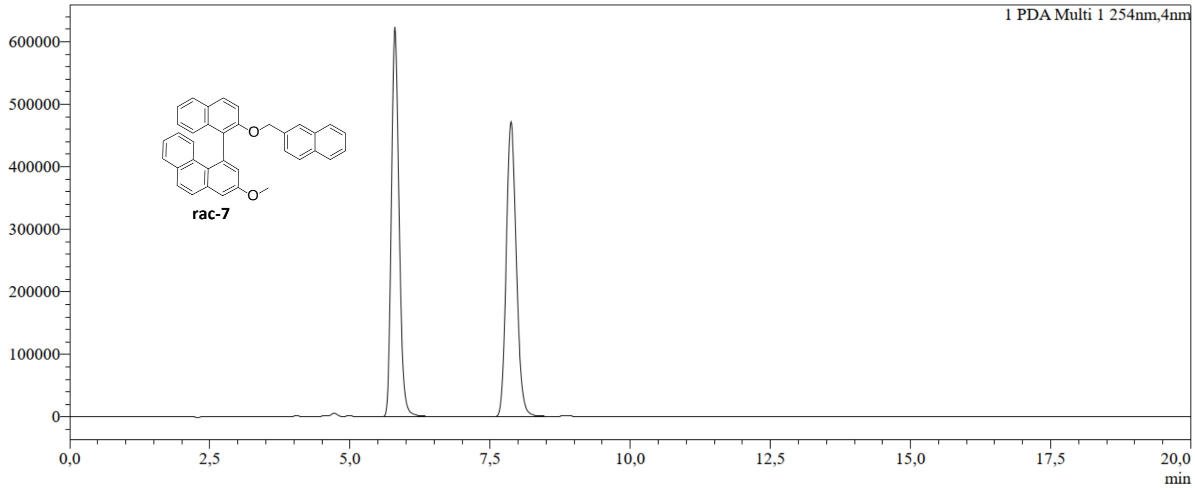

PDA Ch1 254nm

Peak#	Ret. Time	Area	Height	Area%
1	5,801	5969002	623144	50,161
2	7,873	5930711	471587	49,839
Total		11899712	1094731	100,000

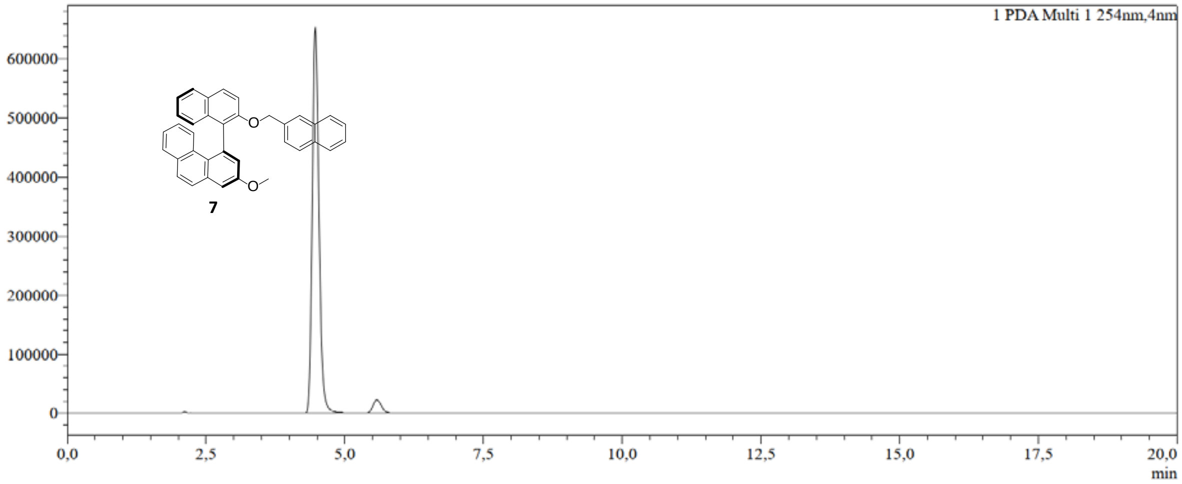

PDA Ch1 254nm

Peak#	Ret. Time	Area	Height	Area%
1	4,468	5513383	653867	95,704
2	5,577	247460	23145	4,296
Total		5760843	677013	100,000

Compound 8

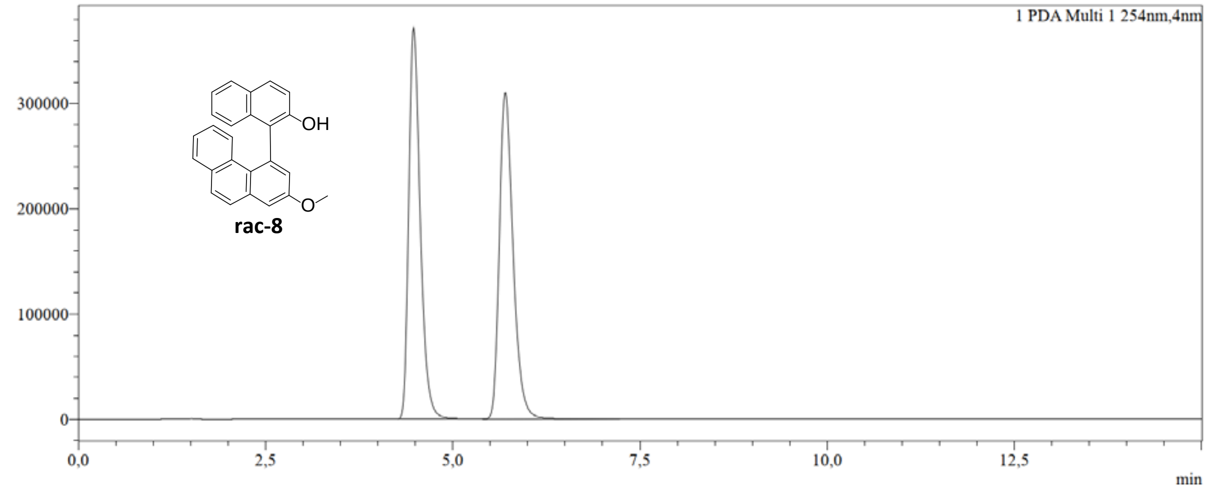

PDA Ch1 254nm

Peak#	Ret. Time	Area	Height	Area%
1	4,479	3990137	372077	50,089
2	5,702	3975878	310275	49,911
Total		7966015	682352	100,000

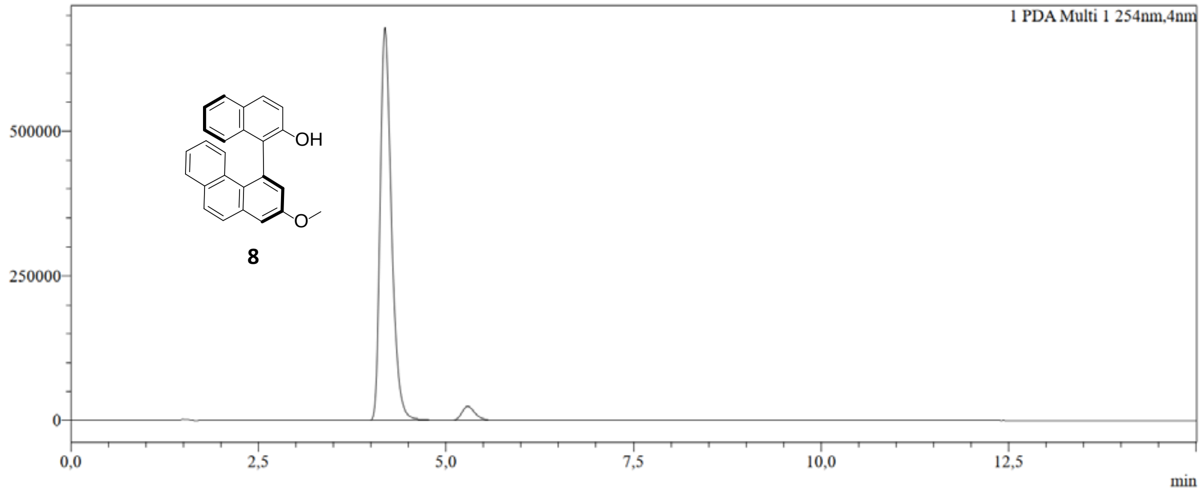

PDA Ch1 254nm

Peak#	Ret. Time	Area	Height	Area%
1	4,187	7134307	678901	95,919
2	5,291	303507	24251	4,081
Total		7437814	703151	100,000

Compound 9

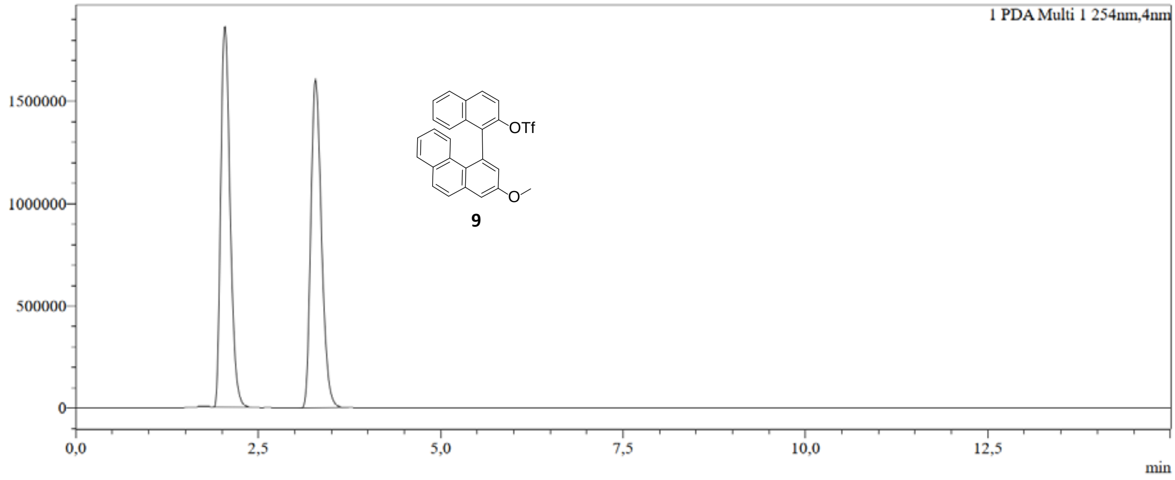

PDA Ch1 254nm

Peak#	Ret. Time	Area	Height	Area%
1	2,043	16631864	1858563	50,487
2	3,286	16311318	1604675	49,513
Total		32943182	3463238	100,000

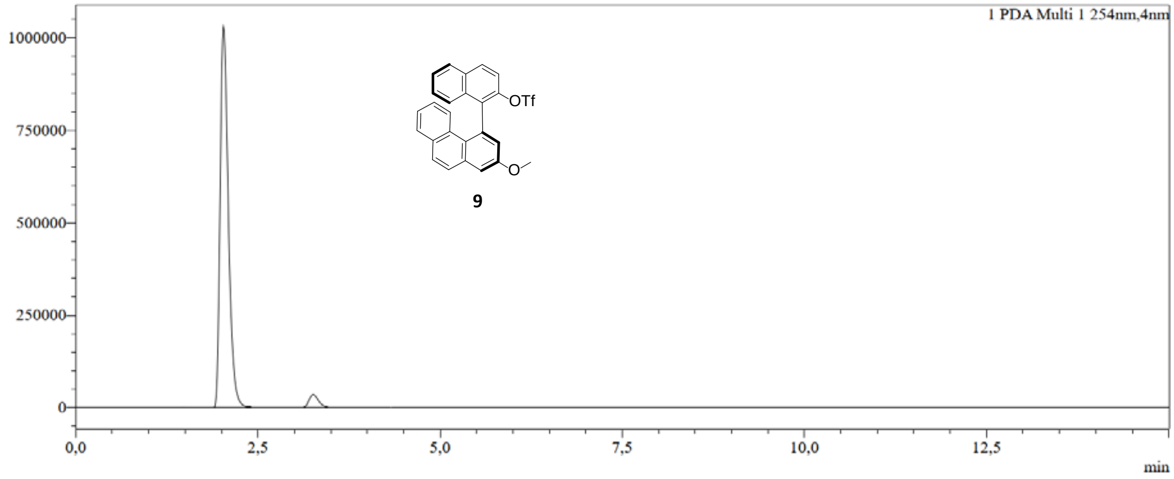

PDA Ch1 254nm

Peak#	Ret. Time	Area	Height	Area%
1	2,030	8094972	1030047	96,141
2	3,260	324888	34557	3,859
Total		8419860	1064604	100,000

Compound 10

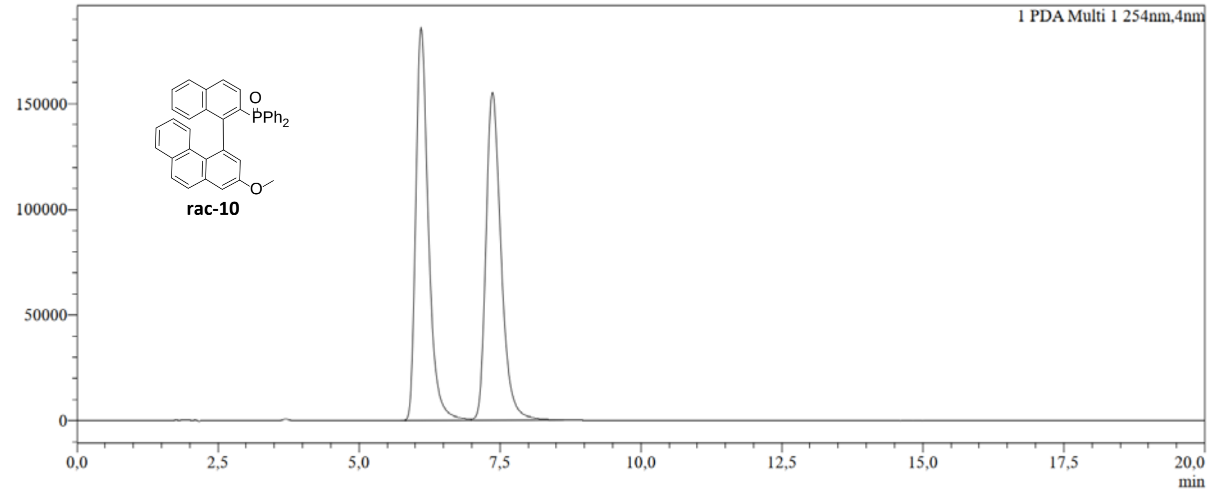

PDA Ch1 254nm

Peak#	Ret. Time	Area	Height	Area%
1	6,102	2902717	185781	50,091
2	7,368	2892182	155105	49,909
Total		5794899	340886	100,000

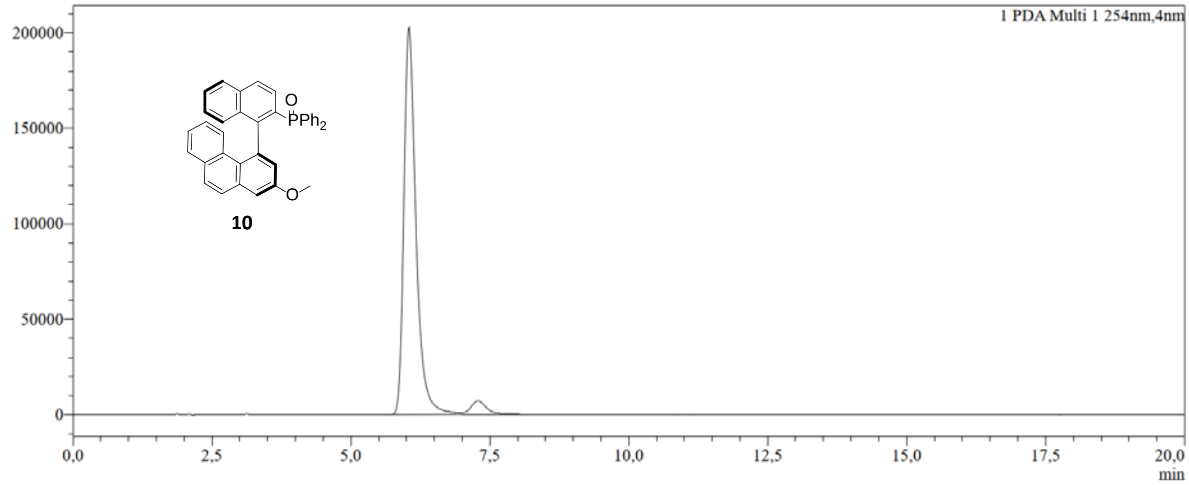

PDA Ch1 254nm

Peak#	Ret. Time	Area	Height	Area%
1	6,044	3064907	202556	95,702
2	7,286	137658	7102	4,298
Total		3202565	209658	100,000

Compound 11

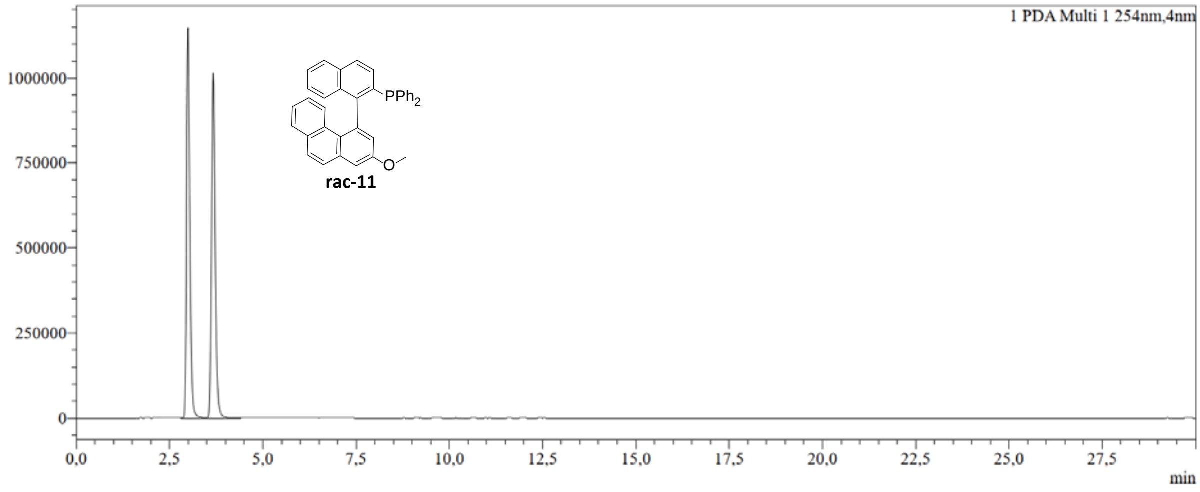

PDA Ch1 254nm

Peak#	Ret. Time	Area	Height	Area%
1	2,993	7091191	1147363	50,271
2	3,669	7014860	1014532	49,729
Total		14106051	2161895	100,000

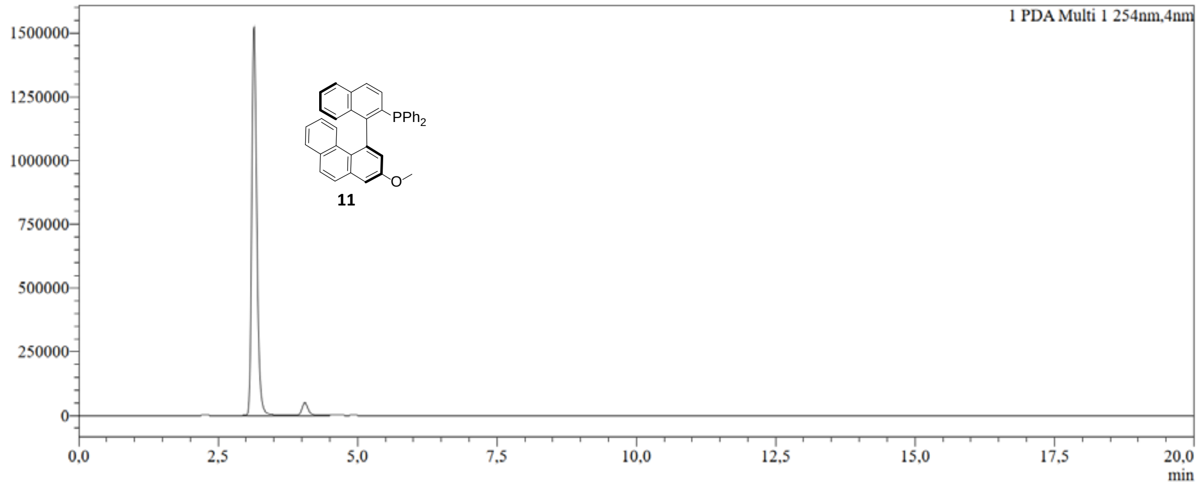

PDA Ch1 254nm

Peak#	Ret. Time	Area	Height	Area%
1	3,142	9608746	1522610	96,032
2	4,054	397049	50881	3,968
Total		10005795	1573491	100,000
